# The Cooperative Double Helicenyl Fragment Model: Efficiently Predicting Stereochemical Stability of Triphenylene-Cored Multiple Helicenes

**DOI:** 10.1021/acs.jpca.6c02107

**Published:** 2026-06-19

**Authors:** Wei-Kai Shao, Mu-Jeng Cheng

**Affiliations:** Department of Chemistry, 34912 National Cheng Kung University, Tainan 701, Taiwan

## Abstract

Predicting the stereochemical stability of complex multiple helicenes (MHs) is conventionally hindered by the combinatorial explosion of stereoisomers and the prohibitive *O*(*N* ^4^) computational scaling of density functional theory (DFT). Herein, we introduce the cooperative double helicenyl fragment (CDHF) model, which deconstructs global relative enthalpies into additive local enthalpic contributions to overcome these limitations. Rigorously validated across seven experimentally synthesized MHs, the CDHF model consistently reproduces the energy distribution of expansive stereoisomeric spaces and correctly pinpoints the experimentally isolated global minima. Exploiting its exceptional parameter transferability, the model functions as a modular design toolkit. We demonstrated this utility by rationally engineering a hypothetical pentadecapole helicene encompassing 10,944 stereoisomers. By strategically manipulating local topologies, we shifted its global minimum to a highly symmetric *C*_3_ configuration, achieving a computational speedup of approximately 3 × 10^5^ over full-molecule DFT. Photophysically, this symmetry-driven architecture effectively eliminates the internal dichroic cancellation, enhancing the long-wavelength chiroptical purity by 82%. Ultimately, the CDHF model provides a precise thermodynamic blueprint for rationally designing complex chiral architectures, establishing itself as a powerful presynthetic strategy for developing advanced chiroptical materials.

## Graphical abstract

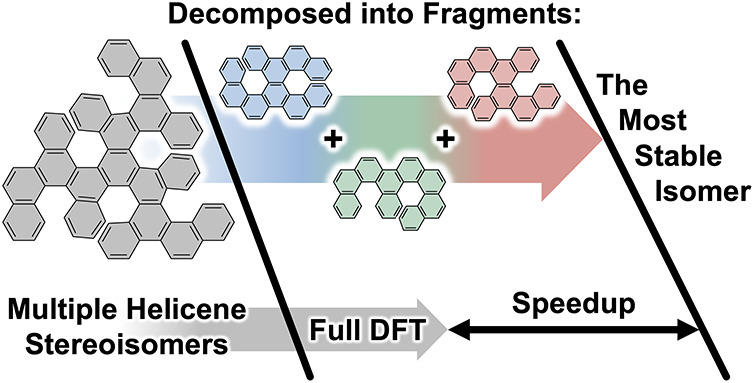

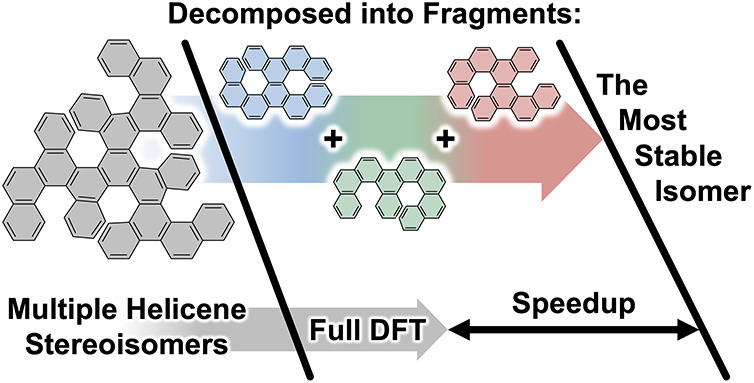

## 1 Introduction

Multiple helicenes (MHs) feature distorted π-systems and rich stereochemistry, offering distinct chiroptical and electronic properties that are heavily dictated by their most stable configurations.^1–5^ Therefore, efficiently identifying the thermodynamically preferred stereoisomers and their symmetries prior to synthesis remains a critical bottleneck in the rational design of these materials. While conventional MH scaffolds generally accommodate no more than six helical substructures,^6^ the triphenylene core has proven uniquely capable of supporting a significantly larger number of helical units. A prime example is the triphenylene-cored dodecapole helicene (MH **7**), which encompasses 1376 stereoisomers.^7^ Because the total number of theoretical candidates grows exponentially with each additional helical unit, exhaustive screening via conventional density functional theory (DFT) becomes computationally prohibitive, despite its high quantitative accuracy.

To avoid the high computational cost of exhaustive full-molecule DFT calculations, we previously introduced the concept of the cooperative double helicenyl fragment (CDHF) in our joint experimental and theoretical study focusing on hexapole helicenes with a triphenylene core.^8^ In that study, we observed that the energy parameters fitted from full-molecule DFT data closely matched the actual enthalpy differences calculated for isolated CDHF subunits. The present paper advances this observation into a fully predictive model. By assuming that helical twisting is primarily driven by short-range steric repulsions, we approximate the total distortion enthalpy of an MH stereoisomer as the sum of local enthalpy differences from adjacent helical substructures. Consequently, the energetics derived solely from small, computationally inexpensive CDHFs can accurately predict the relative enthalpies (Δ*H*_rel_) of full MH stereoisomers, effectively bypassing the need for full-molecule calculations.

## 2 Computational Details

### 2.1 DFT Calculations and Stereoisomeric Space Generation

DFT calculations, including geometry optimizations and vibrational frequency analyses, were performed in the gas phase using Gaussian 16 at the B3LYP-D3­(BJ)/6–31G­(d,p) level.^9–15^ Although relative stability is strictly dictated by Gibbs free energy, relative enthalpy serves as a reliable proxy because these rigid stereoisomers share identical masses, similar dipole moments, and comparable vibrational modes, yielding nearly invariant entropic contributions (see Table S3). This gas-phase enthalpic ordering is robust against environmental perturbations, with continuum solvation evaluations (Table S3) and experimentally isolated structures demonstrating negligible solvent or solid-state packing effects on the stability trends. To parametrize the CDHF model and establish accurate benchmarks, the enthalpies for both full MHs and CDHFs were extracted from fully optimized geometries. To construct the full stereoisomer library for each MH, we generated all possible combinations of local helical configurations. Following standard IUPAC nomenclature, the helical chirality of each local substructure is designated as *P* (plus, right-handed) or *M* (minus, left-handed). Since an MH with *n* helical substructures has 2^*n*^ initial combinations, the final count of theoretical stereoisomers was determined by excluding superimposable duplicates based on molecular symmetry.

Excited-state simplified time-dependent density functional theory (sTDDFT) calculations, including the evaluation of the lowest 50 singlet excited states, were performed using ORCA 6.0.1 at the B3LYP-D3­(BJ)/6–31G­(d,p) level.^10–17^ To evaluate the chiroptical properties, the UV–vis absorption and electronic circular dichroism (ECD) spectra were simulated by applying a frequency-weighted Gaussian broadening with a standard deviation (σ) of 0.15 eV to the calculated rotatory and oscillator strengths. To quantify the degree of molecular dissymmetry, the absorption dissymmetry factor (*g*_CD_) was subsequently derived from the ratio of rotatory and dipole strengths according to standard isotropic definitions.^2,18^ Note that the simulated spectra obtained at this level of theory exhibit a systematic energy shift relative to typical experimental measurements. However, because our analysis focuses exclusively on the relative chiroptical contributions among different stereoisomeric configurations, this systematic shift does not affect the qualitative validity of the results.

### 2.2 Computational Cost Estimation

To quantify the computational advantage of this fragment-based approach, we estimated the computational speedup of the CDHF model over exhaustive DFT screening, based on the *O*(*N* ^4^) scaling of B3LYP-D3­(BJ).^19^ As a conservative estimate, the computational speedup (*S*_comp_) is approximated as

1 $$S_{comp}\approx \frac{\frac{N_{isomers}}{2}\times {\left({Size}_{MH}\right)}^{4}}{\sum_{i}2{\times \left({Size}_{i}\right)}^{4}}$$

Here, *N*_isomers_/2 denotes the number of diastereomers, as enantiomers possess identical energies. The denominator sums the computational costs for all unique CDHF types, accounting for both homochiral and heterochiral forms. Size_MH_ and Size_*i*_ represent the basis function counts for the full MH and the *i*th CDHF, respectively. This estimate is a conservative lower bound because it ignores the typically slower convergence of larger MH scaffolds.

### 2.3 Statistical Confidence Metric

To quantify the reliability of identifying the global minimum, we defined the confidence probability (*P*_conf_) based on the cumulative distribution function (Φ) of the standard normal distribution:^20^

2 $$P_{\mathrm{conf}}=\Phi \left(\frac{{\Delta H}_{\mathrm{gap}}}{\sigma_{\mathrm{error}}}\right)$$

Here, Δ*H*_gap_ denotes the difference in predicted enthalpy between the two most stable stereoisomers, and σ_error_ is the standard deviation of the prediction errors for that specific MH. In simpler terms, this metric indicates the probability that the predicted most stable stereoisomer is genuinely the global minimum, considering the model’s error margin.

## 3 Results and Discussion

### 3.1 Definition and Formulation of the CDHF Model

To implement the proposed additive model, CDHFs were first extracted from the target MHs as minimal structural units encompassing two adjacent helical substructures. Local steric environments and short-range intramolecular π–π interactions were preserved by retaining native substituents and capping truncated bonds with hydrogen atoms. This ensures that individual CDHFs intrinsically capture these local interactions, while the overall stability is accounted for by the summation of these fragment enthalpies. As shown in Figure 1 for MH **1**, this protocol simplifies the nine apparent helical interactions into just two unique CDHF types: the inner-core **CDHF-1** (purple) and the core-wing **CDHF-2** (blue), leveraging its *D*_3_ symmetry to significantly reduce the combinatorial problem. This methodology was applied to define unique CDHFs for MHs **1–9**, as shown in Figure 2. Notably, MHs **1–7** represent previously synthesized architectures with a triphenylene core, providing a robust experimental foundation for subsequent model validation.^7,8,21–24^ Meanwhile, MHs **8** and **9** are hypothetical compounds designed to demonstrate the application of the model for larger, unsynthesized systems. All CDHFs extracted from MHs **1–9** are shown in Figure 3.

**Figure 1:**
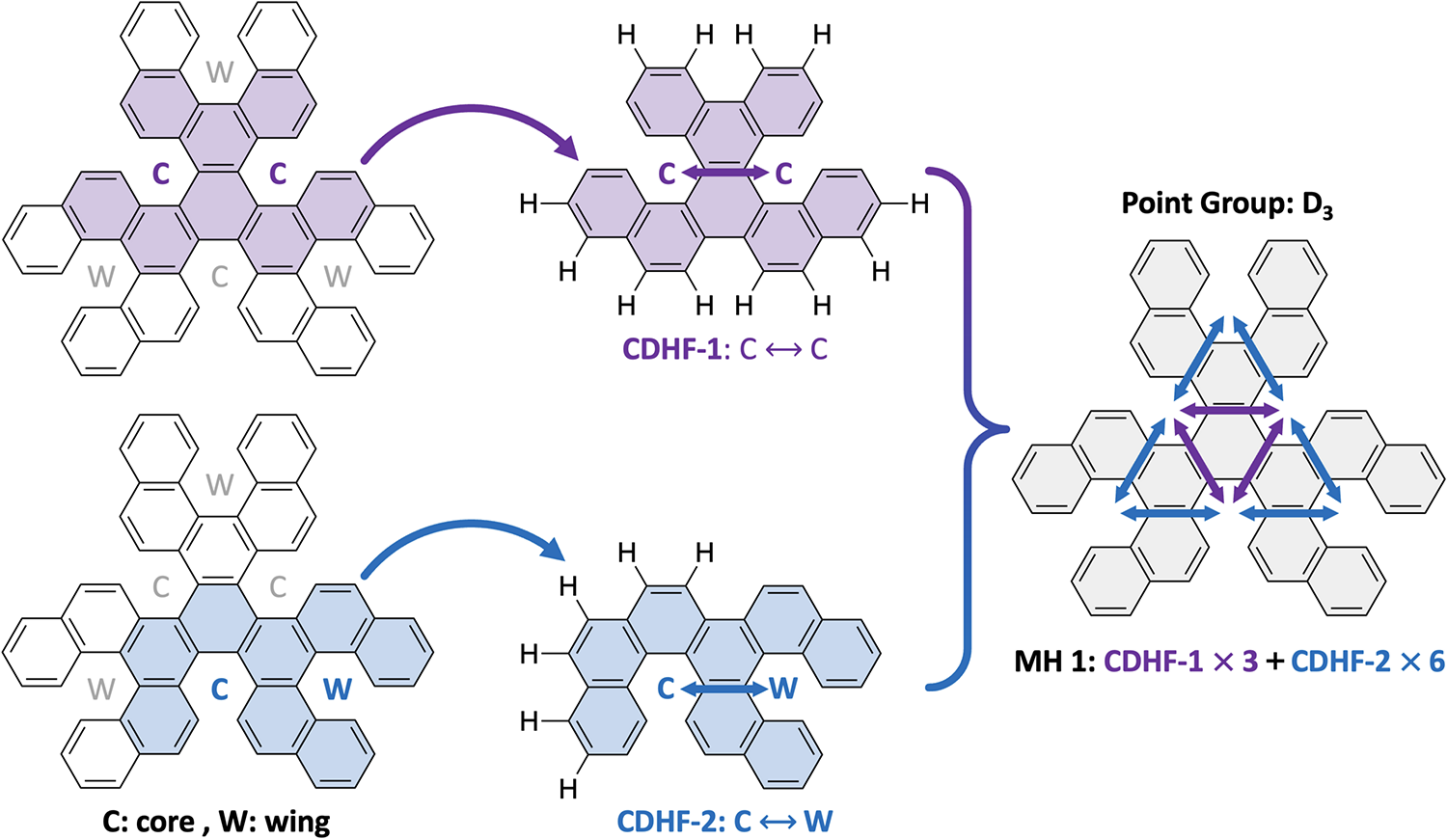
Schematic illustration of the cooperative double helicenyl fragment (CDHF) extraction protocol using the *D*_3_-symmetric multiple helicene (MH) **1** as a template. The abbreviations C and W denote the core and wing helical substructures, respectively. The colored double-headed arrows indicate the specific pairwise helical interactions that define each CDHF type. The explicit hydrogen atoms shown on the extracted CDHF structures represent the capping atoms used to terminate the truncated bonds.

**Figure 2:**
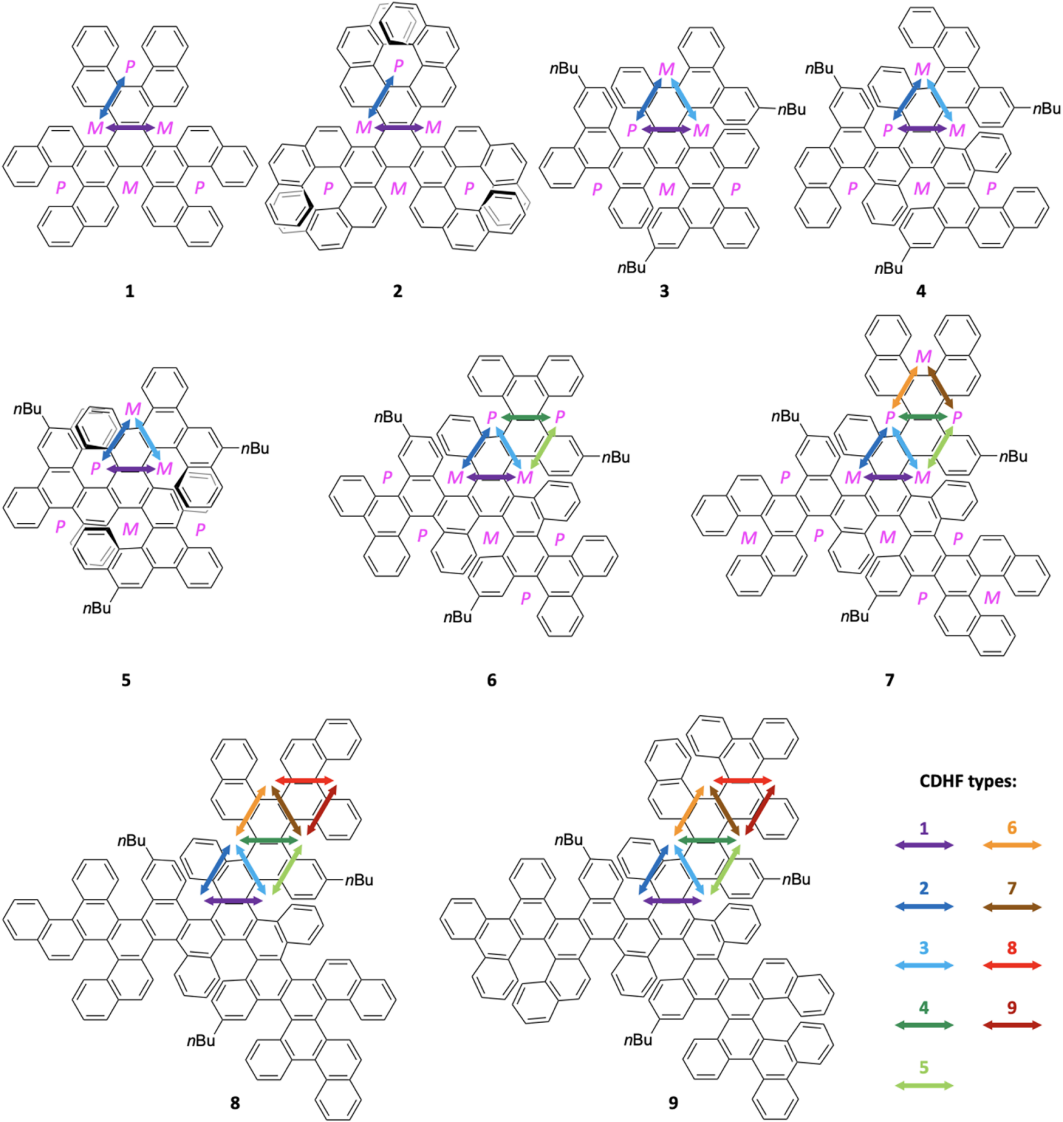
Chemical structures of MHs **1–9** investigated in this study. Colored double-headed arrows denote the unique CDHF types (i) defined within each specific scaffold. For clarity, only unique CDHF types are marked; other equivalent helical interactions can be mapped via molecular symmetry. The pink labels (*P* or *M*) denote the local helicity of the experimentally observed configurations; the corresponding enantiomeric forms are energetically equivalent.

**Figure 3:**
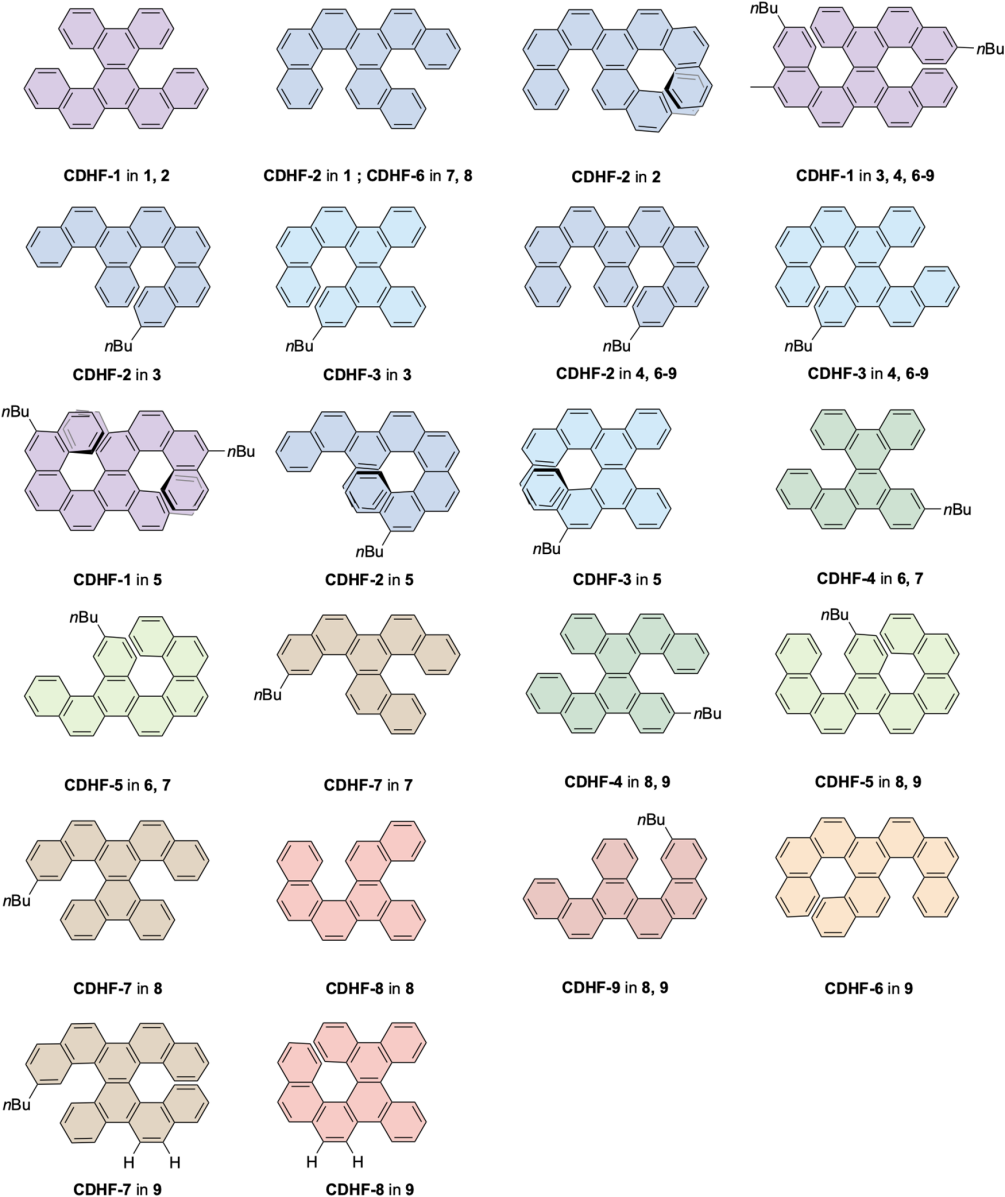
Unique CDHF structures extracted from the parent MHs, which are color-coded as defined in Figure 2.

With the structural building blocks defined, we established the energy relationship between CDHFs and the full molecule. We define the enthalpic contribution (Δ*H*_*i*_) for a specific CDHF type *i* as the enthalpy difference between its heterochiral (*P*,*M* or *M*,*P*) and homochiral (*P*,*P* or *M*,*M*) forms

3 $${\Delta H}_{i}=H_{\mathrm{hetero}}-H_{\mathrm{homo}}$$

By defining the homochiral pair as the local reference (*H*_homo_ ≡ 0), a positive Δ*H*_*i*_ value indicates the homochiral form is more stable, whereas a negative value favors the heterochiral form. Δ*H*_rel_ of any stereoisomer is approximately the sum of its CDHF enthalpic contributions

4 $${\Delta H}_{\mathrm{rel}}\approx \sum_{i}n_{i}{\Delta H}_{i}$$

Here, *n*_*i*_ represents the number of heterochiral CDHFs of type *i* within the full molecule. This formulation naturally establishes the all-homochiral stereoisomer (*n*_*i*_ = 0 for all *i*) as the global reference state (Δ*H*_rel_ = 0).

The computed Δ*H*_*i*_ and the corresponding *S*_comp_ for all investigated MHs are summarized in Table 1. A defining feature of the CDHF model is its high parameter transferability; because identical CDHF structures exhibit the same Δ*H*_*i*_ values, previously established parameters can be directly transferred to newly evaluated MHs, circumventing redundant calculations.

**Table 1:** Computational Speedup (*S*_comp_) for Multiple Helicenes (MHs) and Calculated Enthalpic Contributions (Δ*H*_*i*_) for Cooperative Double Helicenyl Fragments (CDHFs)

		Δ*H*_*i*_ (kcal/mol) for CDHF types (*i*)								
MH	*S*_comp_	1	2	3	4	5	6	7	8	9
**1**	33.83	2.62	–4.45							
**2**	101.00	2.62^a^	–3.58							
**3**	21.08	0.59	–4.06	2.63						
**4**	64.56	0.59^a^	–6.68	3.18						
**5**	22.21	–2.54	–5.64	0.44						
**6**	1330.77	0.59^a^	–6.68^a^	3.18^a^	4.28	–3.54				
**7**	58,383.01	0.59^a^	–6.68^a^	3.18^a^	4.28^a^	–3.54^a^	–4.45^a^	–2.50		
**8**	134,348.34	0.59^a^	–6.68^a^	3.18^a^	4.40	–6.58	–4.45^a^	2.87	–1.21	–5.54
**9**	282,952.81	0.59^a^	–6.68^a^	3.18^a^	4.40^a^	–6.58^a^	–5.65	2.53	2.85	–5.54^a^

a Δ*H*_*i*_ values were transferred from identical CDHF types in smaller MHs.

To demonstrate the practical simplicity of the CDHF model, consider the calculation for the most stable configuration of MH **3** (Figure 2). The *C*_1_-symmetric structure contains two heterochiral **CDHF-1** (*n*_1_ = 2), three heterochiral **CDHF-2** (*n*_2_ = 3), and one heterochiral **CDHF-3** (*n*_3_ = 1). Using the Δ*H*_*i*_ values from Table 1, its relative stability is rapidly estimated using eq 4

$${\Delta H}_{\mathrm{rel}}\approx \sum_{i}n_{i}{\Delta H}_{i}=2\times 0.59+3\times \left(-4.06\right)+1\times 2.63=-8.37\mathrm{kcal}/\mathrm{mol}$$

This simple arithmetic bypasses intensive full-molecule DFT optimization, directly predicting the most stable stereoisomers based purely on local steric environments.

Beyond practical calculations, these local Δ*H*_*i*_ provide valuable physical insights. Focusing on the relationship between CDHF structures and corresponding Δ*H*_*i*_, our analysis reveals that CDHF types adopting a crossed [X]-shaped topology^2^ (e.g., **CDHF-1** in MH **1**, see Figure 1) typically exhibit positive Δ*H*_*i*_, favoring the homochiral configuration. Conversely, fused [F]-shaped topologies^2^ (e.g., **CDHF-2** in MH **1**, see Figure 1) yield negative values, favoring the heterochiral form. This trend holds across most systems. A notable exception is **CDHF-1** in MH **5**, which possesses an [X]-shaped topology yet displays a negative Δ*H*_*i*_ (−2.54 kcal/mol). We attribute this exception to the increased flexibility of the extended π-system, allowing the heterochiral stereoisomer to distribute torsional strain more effectively over the larger scaffold and lower the total energy.

Furthermore, extending the π-system length within similar CDHF scaffolds does not guarantee a consistent trend in Δ*H*_*i*_ for either [X]-shaped or [F]-shaped topologies. For example, appending two benzene rings to each outer wing of MH **1** to form MH **2** increases the Δ*H*_*i*_ of **CDHF-2** by 0.87 kcal/mol. In contrast, appending one benzene ring to each outer wing of MH **3** to form MH **4** decreases the Δ*H*_*i*_ of **CDHF-2** by 2.62 kcal/mol. These changes do not scale linearly with the number of added rings. The only highly predictable shift occurs when an extension changes the fundamental topology. Extending **CDHF-7** from MH **7** to **8** changes its shape from [F] to [X], causing a substantial increase of 5.37 kcal/mol and reversing Δ*H*_*i*_ from negative to positive. These deviations underscore the necessity of explicitly computing Δ*H*_*i*_ values via DFT for each unique CDHF, rather than relying solely on qualitative geometric rules to predict relative stabilities.

### 3.2 Validation of Predictive Accuracy for the CDHF Model

To validate the predictive accuracy of the CDHF model, we explicitly computed the full stereoisomeric spaces for MHs **1–5** to obtain the DFT benchmarks (Δ*H*_DFT_) and compared them against the CDHF-predicted relative enthalpies (Δ*H*_CDHF_). The results demonstrate that the model successfully predicted the most stable configuration for every investigated system, with MHs **1** and **2** adopting *C*_1_-symmetric configurations and MHs **3–5** forming *C*_3_-symmetric structures, as shown in Figure 2.^7,8,21–24^ As illustrated in Figure 4, the correlation between the predicted and benchmark values is highly robust, with coefficients of determination (*R* ^2^) ranging from 0.87 to 0.95 and *P*_conf_ spanning from 97.1 to >99.9%. While some stereoisomers exhibit identical predicted Δ*H*_CDHF_ values due to feature degeneracy, this phenomenon only occurs when stereoisomers share identical local CDHF sets despite differing in global spatial arrangement.

**Figure 4:**
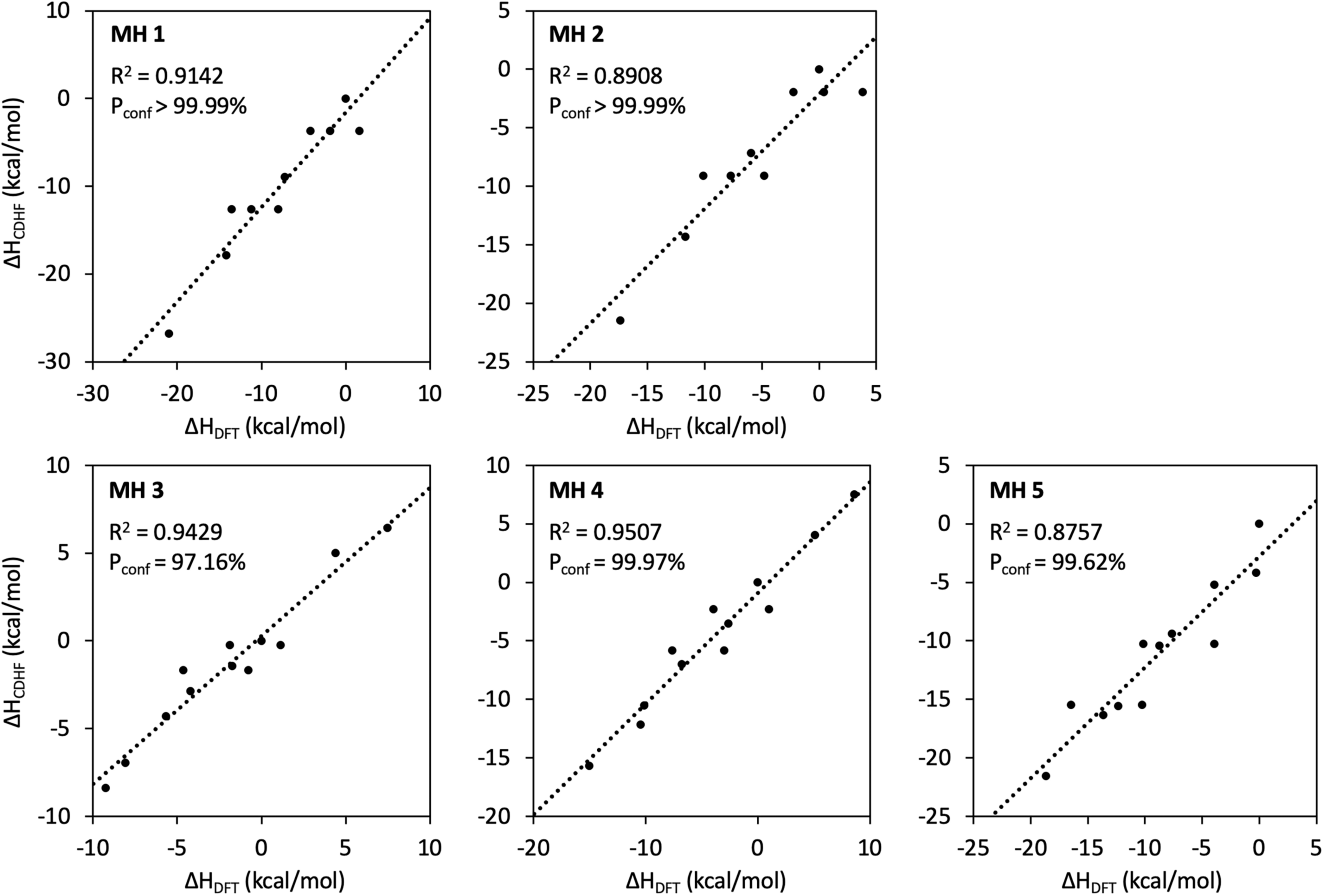
Correlation plots of CDHF-predicted relative enthalpies (Δ*H*_CDHF_) versus DFT benchmarks (Δ*H*_DFT_) for the full stereoisomeric spaces of MHs **1–5**. The coefficient of determination (*R* ^2^) and the confidence probability (*P*_conf_) for predicting the global minimum are provided for each system. Dashed lines represent the linear regression fits.

While the CDHF model excels at predicting the most stable stereoisomer, the predicted Δ*H*_CDHF_ values do not strictly equal the DFT results, as indicated by regression slopes deviating from unity. This discrepancy originates from the CDHF extraction protocol inherently removing the steric constraints imposed by the surrounding scaffold. Consequently, isolated fragments undergo greater relaxation than permitted within the congested full MH. This release of strain typically exaggerates the magnitude of Δ*H*_*i*_, accounting for the observed deviations. Furthermore, neglecting long-range interactions introduces feature degeneracy and leads to minor ranking inversions with a maximum deviation of two positions between nonlowest-energy stereoisomers. Since the total stability is governed by the collective interplay of multiple CDHFs, these systematic deviations are partially mitigated or mutually offset. As a result, relative energy trends are preserved, ensuring the accurate identification of the global minimum without requiring absolute quantitative agreement.

Building upon this validated accuracy, we applied the CDHF model to screen larger, computationally challenging systems: MH **6**, which encompasses 176 stereoisomers, and MH **7**, comprising 1376 stereoisomers. While exhaustive DFT screening for these vast conformational landscapes is intractable, the CDHF model efficiently navigated them, achieving an *S*_comp_ of approximately 1.3 × 10^3^ and 5.8 × 10^4^, respectively. Furthermore, the predicted global minima exactly match the experimentally observed *C*_3_-symmetric architectures,^7,8^ which feature an alternating *M* and *P* sequence from the inner core to the outer wings. The successful identification of these complex configurations confirms the CDHF model’s reliability, establishing its value as a presynthetic screening tool to efficiently navigate the conformational landscapes of MHs.

### 3.3 Rational Design of Multiple Helicenes via the CDHF Model

To demonstrate the predictive power and efficiency of the CDHF model beyond previously validated systems, we extended our analysis to a hypothetical giant MH, pentadecapole helicene **8**. This expanded scaffold comprises 15 helical substructures and encompasses a vast space of 10,944 theoretical stereoisomers. For a system of this size and complexity, identifying the global minimum via full-molecule DFT is computationally prohibitive. In contrast, the CDHF model addresses this challenge with an outstanding *S*_comp_ of approximately 1.35 × 10^5^, rapidly predicting the global minimum of MH **8** to adopt a *C*_1_-symmetric configuration (Figure 5), while the lowest-energy *C*_3_-symmetric stereoisomer ranks only fifth in stability. Subsequent DFT calculations on the five most stable structures predicted by the CDHF model confirmed that the CDHF model correctly identified the global minimum (see Table S5). These DFT results mirror the conclusions drawn from the validation of MHs **1–5**: while the CDHF model fails to quantitatively reproduce Δ*H*_DFT_ values, it reliably identifies the true global minimum.

**Figure 5:**
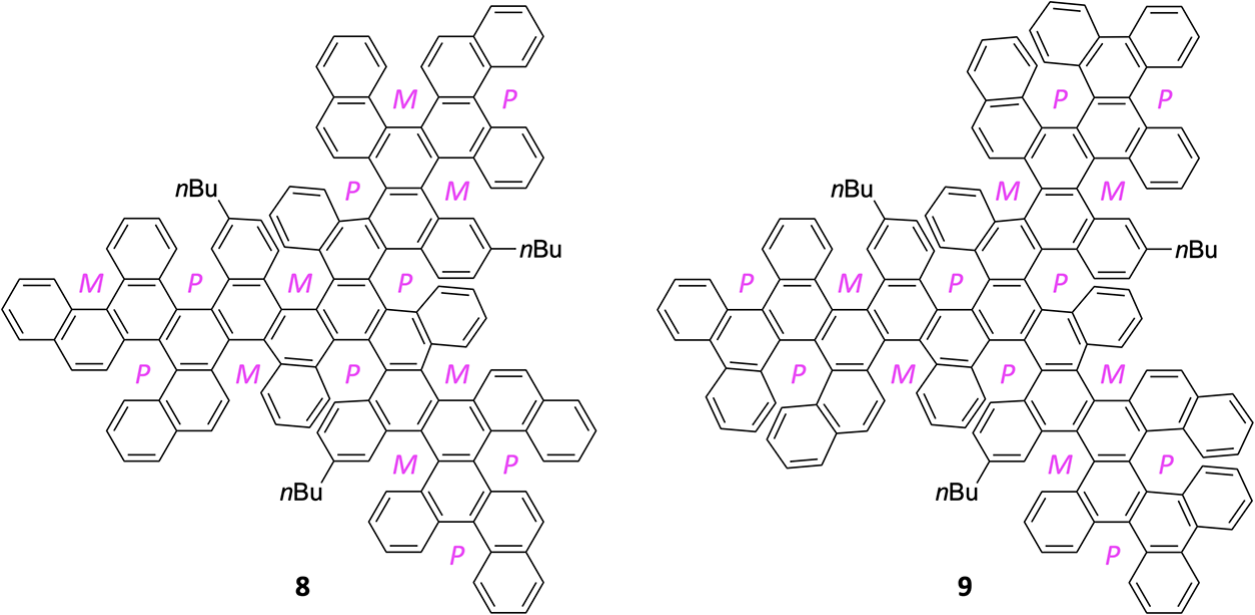
Predicted global minimum structures of the hypothetical pentadecapole helicenes **8** and **9**. MHs 8 and 9 adopt *C*_1_- and *C*_3_-symmetric configurations, respectively. The pink labels (*P* or *M*) denote the local helicity of the depicted global minimum; the corresponding enantiomeric forms are energetically equivalent.

From a photophysical perspective, *C*_1_-symmetric configurations typically yield suboptimal chiroptical properties. Experimental data confirm that highly symmetric architectures (e.g., MHs **6** and **7**) exhibit profoundly enhanced Cotton effects (Δ*ε*) in the low-energy region (>400 nm) compared to *C*_1_-symmetric structures (e.g., MHs **3–5**).^7,8^ This limitation arises because the unsymmetrical spatial arrangement of mixed *P* and *M* helical substructures leads to the destructive interference of local transition dipole moments.^2^ Corroborating this mechanism, sTDDFT simulations of the ECD spectra for the most stable *C*_1_- and *C*_3_-symmetric stereoisomers of MH **8** confirm that low-symmetry helical arrangements induce mutually canceling Δ*ε* signals.^18,25^ This geometric dilution inherent to the *C*_1_ configuration restricts the overall dichroic response of MH **8** in the long-wavelength region (>450 nm) dominated by frontier orbital transitions, yielding a suppressed *g*_CD_ of 2.56 × 10^–3^ at 496.0 nm. In stark contrast, the aforementioned lowest-energy *C*_3_-symmetric stereoisomer of MH **8** exhibits a significantly enhanced *g*_CD_ of 6.16 × 10^–3^ at 498.8 nm, representing a 141% increase compared to its *C*_1_-symmetric counterparts (Figure 6). This pronounced amplification aligns with theoretical expectations, underscoring the critical necessity of rationally designing architectures with highly symmetric global minima to maximize macroscopic optical performance.^2^

**Figure 6:**
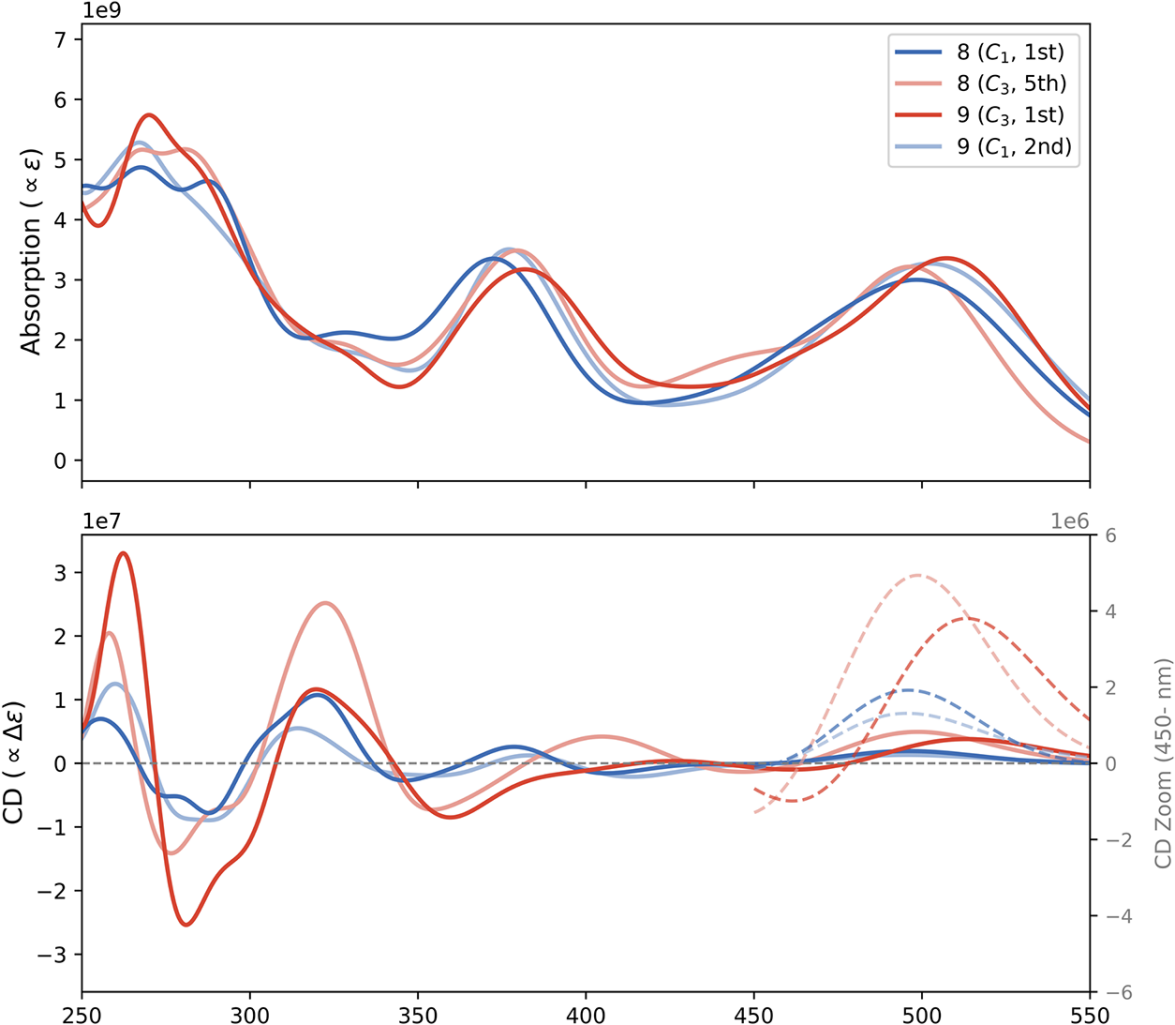
Simulated UV–vis absorption (top) and electronic circular dichroism (ECD, bottom) spectra for the most stable *C*_1_- and *C*_3_-symmetric stereoisomers of MH **8** and **9**. Stereoisomers are color-coded by symmetry: blue tones represent *C*_1_-symmetric and red tones represent *C*_3_-symmetric configurations. The parenthetical labels in the legend denote the point group and the Δ*H*_CDHF_ stability ranking. The dashed lines in the ECD panel, corresponding to the right *Y*-axis, show magnified signals in the low-energy region (>450 nm), illustrating the symmetry-induced enhancement of Δ*ε*.

To overcome the photophysical limitations imposed by this thermodynamically favored *C*_1_-symmetric geometry of MH **8**, we sought to rationally design a highly symmetric variant, designated as MH **9**. Our design strategy was inspired by the relationship between the CDHF topology and the Δ*H*_*i*_ established in Section 3.1, which dictates that [X]-shaped and [F]-shaped topologies typically stabilize distinct local configurations. By introducing an outer-wing benzene ring to the **CDHF-8** domain of MH **8**, we structurally converted this local segment from an [X]-shaped to an [F]-shaped topology (Figure 3). This topological manipulation effectively shifts the Δ*H*_*i*_ from negative to positive, thereby driving a thermodynamic shift in the global minimum. Utilizing the CDHF model, which required only six DFT calculations to parametrize three new CDHFs, we efficiently predicted that the global minimum of MH **9** adopts a *C*_3_-symmetric configuration (Figure 5), while the lowest-energy *C*_1_-symmetric stereoisomer ranks second in stability. This predictive process achieved a remarkable *S*_comp_ of approximately 3 × 10^5^. Subsequent DFT calculations on the five most stable structures confirmed that the CDHF-predicted global minimum matches the DFT results (see Table S5), thereby validating the predictive accuracy of the CDHF model.

Photophysically, this rationally designed symmetric global minimum successfully translates high structural symmetry into chiroptical amplification. As illustrated in the simulated UV–vis absorption spectra (Figure 6), all selected stereoisomers of MHs **8** and MH **9** exhibit nearly identical absorption profiles, as they share comparable extended π-conjugated frameworks and corresponding transition dipole moments. In striking contrast, the corresponding ECD spectra reveal profound differences. For both MH **8** and MH **9**, the *C*_3_-symmetric stereoisomers display substantially larger Δ*ε* compared to their *C*_1_-symmetric counterparts. This symmetry-driven enhancement is particularly prominent in the low-energy transition regime (>450 nm), which is highly relevant for advanced chiroptical applications. Specifically, the *C*_3_-symmetric global minimum of MH **9** achieves a *g*_CD_ of 4.65 × 10^–3^ at 513.4 nm, representing an approximate 82% enhancement in chiroptical purity compared to the most stable stereoisomer of MH **8**. Furthermore, the structural expansion in MH **9** induces a bathochromic shift of approximately 17 nm in the primary CD peak, highlighting the spectral tunability of the molecular framework. Consequently, the CDHF model functions not merely as a rapid computational screening tool but as a precise design blueprint for engineering molecular materials. By strategically manipulating local substructures, complex architectures can be rationally refined to ensure that their most stable configurations inherently possess the highest symmetry-driven optical performance.

## 4 Conclusions

In this work, we introduce the CDHF model, which deconstructs the global Δ*H*_rel_ into additive local Δ*H*_*i*_. Rigorously validated across seven experimentally synthesized MHs, this model accurately reproduces stereoisomeric energy distributions and identifies the true global minima. Furthermore, its inherent parameter transferability across diverse molecular scaffolds advances the CDHF model from a rapid screening method to a modular design toolkit. To demonstrate this utility, we navigated the 10,944-stereoisomer conformational landscape of a hypothetical pentadecapole helicene, MH **8**. By strategically manipulating local CDHF topologies, we rationally engineered MH **9**, successfully shifting its thermodynamic global minimum to a highly symmetric *C*_3_ configuration while achieving a *S*_comp_ of approximately 3 × 10^5^ over exhaustive DFT. Photophysically, this *C*_3_-symmetric MH **9** effectively eliminates the internal cancellation of dichroic signals, yielding an 82% enhancement in long-wavelength chiroptical purity compared to the *C*_1_-symmetric MH **8**. Ultimately, this work provides a precise thermodynamic blueprint for describing complex chiral architectures through cooperative double helicenyl interactions, establishing the CDHF model as a powerful presynthetic strategy for the development of advanced chiroptical materials. Looking forward, this additive framework can be systematically extended by defining higher-order helicenyl fragments to capture long-range interactions in giant architectures.

## Supplementary Material



## Notes

Detailed protocols for stereoisomeric space generation, along with optimized geometries, absolute energetics, and relative enthalpy tables for the cooperative double helicenyl fragments of multiple helicenes (MHs) **1–9** and the stereoisomers of MHs **1–5** and predicted low-energy stereoisomers for MHs **6–9** (PDF)
