# Supplementary material for: The Cooperative Double Helicenyl Fragment Model: Efficiently Predicting Stereochemical Stability of Triphenylene-Cored Multiple Helicenes
Source: J Phys Chem A. 2026 Jun 19;130(26):5088–96. doi: 10.1021/acs.jpca.6c02107 (PMC13339626; doi:10.1021/acs.jpca.6c02107)
Supplement: Supplementary file 1 [file jp6c02107_si_001.pdf]

Supporting Information for

The Cooperative Double Helicenyl Fragment Model: Efficiently Predicting Stereochemical Stability of Triphenylene-Cored Multiple Helicenes

Wei-Kai Shao, Mu-Jeng Cheng*

Department of Chemistry, National Cheng Kung University, Tainan, 701, Taiwan

Submitted to
J. Phys. Chem. A

June 1st, 2026

Corresponding Author
*Email: mjcheng@mail.ncku.edu.tw

Table of Contents

S1. Detailed Protocols for Stereoisomeric Space Generation S2

- **Figure S1.** Schematic representations of the molecular graphs for MHs **1–9**.

S2. Energetics and Parameterization of CDHFs for MHs 1–9 .... S3

Table S1. H and ΔH_i of the CDHFs for MHs **1–9**.

S3. Relative Energies and Environmental Validations for MHs 1–5 .... S5

- **Table S2.** ΔH_{CDHF} and ΔH_{DFT} for all stereoisomers of MHs **1–5**.
- **Table S3.** ΔH_{DFT}, ΔG_{DFT}, $T\Delta S$, and ΔH_{DMSO} for all stereoisomers of MHs **1–5**.

S4. Predicted Low-Energy Stereoisomers for MHs 6–9 .... S9

- **Table S4.** ΔH_{CDHF} for the top 10-ranked stereoisomers of MHs **6–9**.

S5. DFT and sTDDFT Calculations of MHs 8 and 9 .... S11

- **Table S5.** ΔH_{CDHF} and ΔH_{DFT} for the five most stable stereoisomers of MHs **8** and **9**.
- **Figure S2.** All simulated spectra for the selected stereoisomers of MHs **8** and **9**.

S6. Cartesian Coordinates of Optimized Geometries S14

S1. Detailed Protocols for Stereoisomeric Space Generation

We employed a graph-theoretical approach to systematically represent and evaluate the stereoisomeric spaces of multiple helicenes (MHs). In this model, the helical substructures are treated as nodes, and their apparent helical interactions are treated as edges (visually represented by the double-headed arrows in **Figures 1** and **2** of the main text). As illustrated in **Figure S1**, the C_{3h} and C_3 architectures are represented as extended planar triangular networks.

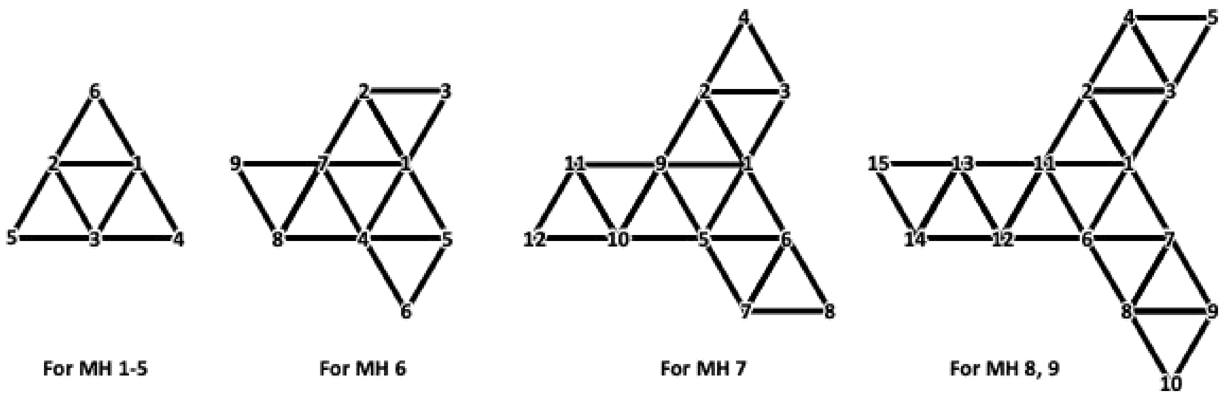

Figure S1. Schematic representations of the molecular graphs for multiple helicenes (MHs) **1–9**.

Because each node can adopt either a P or M configuration, an MH with n nodes yields 2^n initial permutations. To determine the exact number of unique stereoisomers, we generated all combinations and applied point-group symmetry operations (rotations and mirror reflections for the C_3 and C_{3h} groups) to the 2D molecular graphs. Configurations that perfectly overlap under these operations were excluded as duplicates. Importantly, while these mirror reflections only move the topological node positions on the graph, enantiomers possess fully inverted P/M sequences and are considered stereochemically distinct. Consequently, we retained both

enantiomers in the total stereoisomer count, despite their thermodynamic degeneracy. For instance, MHs **8** and **9** possess 15 nodes, generating 2^{15} initial permutations that are ultimately reduced to the 10,944 unique stereoisomers discussed in **Section 3.3** of the main text.

Within the graph-theoretical framework, extracting cooperative double helicenyl fragments (CDHFs) from an MH corresponds to evaluating the edges within its molecular graph. Unique CDHF types are defined as edges that are non-superimposable under the point-group symmetry operations of the parent scaffold. A CDHF is classified as homochiral if its connected nodes possess identical helicities (P,P or M,M), and heterochiral if they differ (P,M or M,P). Consequently, distinct global stereoisomers can yield the exact same set of local CDHFs, leading to feature degeneracy.

S2. Energetics and Parameterization of CDHFs for MHs 1–9

To determine the enthalpic contribution (ΔH_i, as defined by **Equation 3** in the main text) for each unique CDHF type, we extracted the corresponding fragments from the parent MHs **1–9**. All CDHFs were fully optimized using density functional theory (DFT) at the B3LYP-D3(BJ)/6-31G(d,p) level. The optimized 3D structures for both the homochiral and heterochiral forms of each CDHF are shown in **Section S6**. The CDHF configurations, minimum vibration frequencies (ν_{min}), calculated absolute enthalpies (H), and ΔH_i are summarized in **Table S1** (corresponding to the parameters summarized in **Table 1** of the main text).

Table S1. Calculated absolute enthalpies (H) and enthalpic contributions (ΔH_i) of the cooperative double helicenyl fragments (CDHFs) for multiple helicenes (MHs) **1–9**.

Parent MH	CDHF	Configuration	$\boldsymbol{\nu_{min}}$ (cm^{-1})	$\boldsymbol{H}$ (Hartree)	$\Delta\boldsymbol{H_i}$ (kcal/mol)
**1**	**1**	Homochiral	36.97	-1307.454500	0
**1**	**1**	Heterochiral	29.20	-1307.450331	2.62
**1**	**2**	Homochiral	31.42	-1307.449719	0
**1**	**2**	Heterochiral	35.69	-1307.456809	-4.45
**2**	**2**	Homochiral	27.62	-1614.678882	0
**2**	**2**	Heterochiral	28.50	-1614.684589	-3.58
**3**	**1**	Homochiral	11.21	-1929.023982	0
**3**	**1**	Heterochiral	11.81	-1929.023047	0.59
**3**	**2**	Homochiral	14.04	-1464.624789	0
**3**	**2**	Heterochiral	14.38	-1464.631264	-4.06
**3**	**3**	Homochiral	12.10	-1464.629756	0
**3**	**3**	Heterochiral	11.79	-1464.625562	2.63
**4**	**2**	Homochiral	12.85	-1618.234324	0
**4**	**2**	Heterochiral	13.53	-1618.244973	-6.68
**4**	**3**	Homochiral	12.49	-1618.240253	0
**4**	**3**	Heterochiral	11.84	-1618.235192	3.18
**5**	**1**	Homochiral	14.60	-2236.254224	0
**5**	**1**	Heterochiral	14.99	-2236.258264	-2.54
**5**	**2**	Homochiral	18.96	-1618.239938	0
**5**	**2**	Heterochiral	17.26	-1618.248924	-5.64
**5**	**3**	Homochiral	14.83	-1618.242708	0
**5**	**3**	Heterochiral	18.42	-1618.242001	0.44
**6**	**4**	Homochiral	16.20	-1311.018402	0
**6**	**4**	Heterochiral	10.40	-1311.011586	4.28
**6**	**5**	Homochiral	20.86	-1464.627652	0
**6**	**5**	Heterochiral	13.20	-1464.633289	-3.54
**7**	**7**	Homochiral	11.93	-1311.012254	0
**7**	**7**	Heterochiral	15.27	-1311.016232	-2.50
**8**	**4**	Homochiral	17.22	-1464.629601	0
**8**	**4**	Heterochiral	23.66	-1464.622588	4.40
**8**	**5**	Homochiral	17.89	-1618.236367	0
**8**	**5**	Heterochiral	16.05	-1618.246845	-6.58
**8**	**7**	Homochiral	21.57	-1464.628236	0
**8**	**7**	Heterochiral	20.14	-1464.623657	2.87

**8**	**8**	Homochiral	34.23	-1153.843094	0
**8**	**8**	Heterochiral	38.49	-1153.845017	-1.21
**8**	**9**	Homochiral	24.15	-1311.012042	0
**8**	**9**	Heterochiral	23.94	-1311.020873	-5.54
**9**	**6**	Homochiral	27.01	-1461.062434	0
**9**	**6**	Heterochiral	28.99	-1461.071437	-5.65
**9**	**7**	Homochiral	19.22	-1618.240726	0
**9**	**7**	Heterochiral	20.35	-1618.236697	2.53
**9**	**8**	Homochiral	34.64	-1307.459111	0
**9**	**8**	Heterochiral	29.35	-1307.454574	2.85

S3. Relative Enthalpy Comparisons for MHs 1–5

To validate the predictive accuracy of the CDHF model, the full stereoisomeric spaces for MHs **1–5** were exhaustively evaluated. All MHs were fully optimized using DFT at the B3LYP-D3(BJ)/6-31G(d,p) level. The optimized 3D structures for all diastereomers of each MH are shown in **Section S6**. MH configurations, ν_{min}, H, CDHF-predicted relative enthalpies (ΔH_{CDHF}), and DFT benchmarks (ΔH_{DFT}) are detailed in **Table S2** (corresponding directly to the data points plotted in **Figure 4** of the main text).

Table S2. CDHF-predicted relative enthalpies (ΔH_{CDHF}) and DFT benchmarks (ΔH_{DFT}) for all stereoisomers of MHs **1–5**.

**MH**	**Configuration**	$\boldsymbol{\nu_{min}}$ (cm^{-1})	$\boldsymbol{H}$ (Hartree)	$\Delta\boldsymbol{H}$ (kcal/mol)	
				$\boldsymbol{DFT}^{b}$	$\boldsymbol{CDHF}$
**1**	MMMMMM	19.34	-2536.306854	0	0
**1**	MMMMMP	18.29	-2536.318389	-7.24	-8.90
**1**	MMMMPP	19.01	-2536.329511	-14.22	-17.80
**1**	MMMPPP	20.58	-2536.340344	-21.02	-26.70
**1**	MMPMMM	18.03	-2536.313521	-4.18	-3.66
**1**	MMPMMP	17.03	-2536.328426	-13.54	-12.56
**1**	MMPMPM	17.41	-2536.309759	-1.82	-3.66

**1**	MMPPMP	17.34	-2536.324771	-11.24	-12.56
**1**	MMPPPM	16.89	-2536.304286	1.61	-3.66
**1**	MMPPPP	16.96	-2536.319634	-8.02	-12.56
**2**	MMMMMM	15.90	-3457.995622	0	0
**2**	MMMMMP	15.00	-3458.005131	-5.97	-7.16
**2**	MMMMPP	15.22	-3458.014305	-11.72	-14.32
**2**	MMMPPP	16.42	-3458.023337	-17.39	-21.48
**2**	MMPMMM	14.54	-3457.999183	-2.23	-1.92
**2**	MMPMMP	14.10	-3458.011739	-10.11	-9.08
**2**	MMPMPM	14.14	-3457.994965	0.41	-1.92
**2**	MMPPMP	14.52	-3458.007912	-7.71	-9.08
**2**	MMPPPM	14.39	-3457.989513	3.83	-1.92
**2**	MMPPPP	14.69	-3458.003261	-4.79	-9.08
**3**	MMMMMM	7.75	-3007.843138	0	0
**3**	MMMMMP	7.80	-3007.845918	-1.74	-1.43
**3**	MMMMPP	8.92	-3007.849884	-4.23	-2.86
**3**	MMMPPP	9.83	-3007.852158	-5.66	-4.29
**3**	MMPMMM	8.54	-3007.846143	-1.89	-0.25
**3**	MMPMMP	7.78	-3007.850565	-4.66	-1.68
**3**	MMPMPM	8.77	-3007.85601	-8.08	-6.94
**3**	MMPMPP	8.87	-3007.857913	-9.27	-8.37
**3**	MMPPMM	8.43	-3007.831236	7.47	6.44
**3**	MMPPMP	7.86	-3007.836148	4.39	5.01
**3**	MMPPPM	8.63	-3007.841304	1.15	-0.25
**3**	MMPPPP	8.48	-3007.844374	-0.78	-1.68
**4**	MMMMMM	8.18	-3468.671728	0	0
**4**	MMMMMP	7.60	-3468.675913	-2.63	-3.50
**4**	MMMMPP	8.03	-3468.682556	-6.79	-7.00
**4**	MMMPPP	8.92	-3468.687845	-10.11	-10.50
**4**	MMPMMM	8.56	-3468.677989	-3.93	-2.32
**4**	MMPMMP	9.01	-3468.683846	-7.60	-5.82
**4**	MMPMPM	8.49	-3468.688392	-10.46	-12.18
**4**	MMPMPP	8.65	-3468.695656	-15.02	-15.68
**4**	MMPPMM	8.79	-3468.658028	8.60	7.54
**4**	MMPPMP	8.21	-3468.663604	5.10	4.04
**4**	MMPPPM	8.12	-3468.670173	0.98	-2.32
**4**	MMPPPP	8.26	-3468.676498	-2.99	-5.82
**5**	MMMMMM	11.82	-3468.685884	0	0
**5**	MMMMMP	10.46	-3468.692114	-3.91	-5.20
**5**	MMMMPP	12.26	-3468.699851	-8.76	-10.40

**5**	MMMPPP	12.78	-3468.705558	-12.35	-15.60
**5**	MMPMMM	10.07	-3468.702057	-10.15	-10.28
**5**	MMPMMP	12.89	-3468.712124	-16.47	-15.48
**5**	MMPMPM	10.14	-3468.707584	-13.62	-16.36
**5**	MMPMPP	12.23	-3468.715603	-18.65	-21.56
**5**	MMPPMM	7.81	-3468.686319	-0.27	-4.20
**5**	MMPPMP	12.53	-3468.698053	-7.64	-9.40
**5**	MMPPPM	8.86	-3468.692173	-3.95	-10.28
**5**	MMPPPP	12.56	-3468.702226	-10.25	-15.48

[a] The configurations are represented by their helical sequence (P or M), corresponding to the numbered nodes defined in **Figure S1**.
[b] ΔH_{DFT} is defined with the all-homochiral configuration as the global reference state.

Furthermore, to justify the use of gas-phase relative enthalpies as the primary thermodynamic metric, we evaluated the entropic and environmental effects for MHs **1–5**. As detailed in **Table S3**, the entropic contributions to the relative free energies ($T\Delta S$) are negligible due to the highly rigid nature of these stereoisomers. Consequently, the relative energy rankings based on gas-phase ΔH_{DFT} and ΔG_{DFT} are strictly identical. Additionally, single-point energy evaluations using the SMD continuum solvation model (in DMSO) demonstrate that solvation introduces only marginal energetic perturbations. These minor solvent effects strictly preserve the predicted stability ordering and global minima across all investigated systems.

Table S3. Comparisons of gas-phase relative enthalpies (ΔH_{DFT}), relative Gibbs free energies (ΔG_{DFT}), entropic contributions ($T\Delta S$), and SMD-solvated relative enthalpies (ΔH_{DMSO}) for all stereoisomers of MHs **1–5**.

MH	Configuration[a]	ΔH_{DFT}[b]	ΔG_{DFT}[b]	$T\Delta S$[b]	ΔH_{DMSO}[b]
**1**	MMMMMM	0	0	0	0
**1**	MMMMMP	-7.24	-7.35	0.11	-7.18

**1**	MMMMPP	-14.22	-14.44	0.22	-14.14
**1**	MMMPPP	-21.02	-21.31	0.29	-20.91
**1**	MMPMMM	-4.18	-4.53	0.35	-4.28
**1**	MMPMMP	-13.54	-13.96	0.42	-13.58
**1**	MMPMPM	-1.82	-2.20	0.38	-1.97
**1**	MMPPMP	-11.24	-11.67	0.43	-11.34
**1**	MMPPPM	1.61	1.20	0.41	1.45
**1**	MMPPPP	-8.02	-8.46	0.44	-8.14
**2**	MMMMMM	0	0	0	0
**2**	MMMMMP	-5.97	-6.39	0.42	-6.03
**2**	MMMMPP	-11.72	-12.51	0.79	-11.86
**2**	MMMPPP	-17.39	-18.41	1.02	-17.65
**2**	MMPMMM	-2.23	-2.72	0.49	-2.42
**2**	MMPMMP	-10.11	-10.97	0.86	-10.45
**2**	MMPMPM	0.41	-0.12	0.53	0.28
**2**	MMPPMP	-7.71	-8.55	0.84	-8.02
**2**	MMPPPM	3.83	3.48	0.35	3.73
**2**	MMPPPP	-4.79	-5.52	0.73	-5.10
**3**	MMMMMM	0	0	0	0
**3**	MMMMMP	-1.74	-1.97	0.23	-1.76
**3**	MMMMPP	-4.23	-4.54	0.31	-4.22
**3**	MMMPPP	-5.66	-6.00	0.34	-5.67
**3**	MMPMMM	-1.89	-1.91	0.02	-2.27
**3**	MMPMMP	-4.66	-4.78	0.12	-5.07
**3**	MMPMPM	-8.08	-7.83	-0.25	-8.29
**3**	MMPMPP	-9.27	-9.05	-0.22	-9.64
**3**	MMPPMM	7.47	8.02	-0.55	6.90
**3**	MMPPMP	4.39	4.57	-0.18	3.96
**3**	MMPPPM	1.15	1.61	-0.46	0.72
**3**	MMPPPP	-0.78	-0.71	-0.07	-1.28
**4**	MMMMMM	0	0	0	0
**4**	MMMMMP	-2.63	-2.36	-0.27	-2.61
**4**	MMMMPP	-6.79	-6.49	-0.3	-6.78
**4**	MMMPPP	-10.11	-9.31	-0.8	-10.25
**4**	MMPMMM	-3.93	-3.41	-0.52	-4.10
**4**	MMPMMP	-7.60	-6.25	-1.35	-7.65
**4**	MMPMPM	-10.46	-10.05	-0.41	-10.73
**4**	MMPMPP	-15.02	-14.18	-0.84	-15.40
**4**	MMPPMM	8.60	9.90	-1.3	8.28
**4**	MMPPMP	5.10	6.57	-1.47	5.07

**4**	MMPPPM	0.98	1.62	-0.64	0.60
**4**	MMPPPP	-2.99	-1.83	-1.16	-3.38
**5**	MMMMMM	0	0	0	0
**5**	MMMMMP	-3.91	-3.95	0.04	-3.84
**5**	MMMMPP	-8.76	-8.77	0.01	-8.39
**5**	MMMPPP	-12.35	-12.20	-0.15	-11.68
**5**	MMPMMM	-10.15	-10.03	-0.12	-10.00
**5**	MMPMMP	-16.47	-15.82	-0.65	-15.92
**5**	MMPMPM	-13.62	-13.71	0.09	-13.66
**5**	MMPMPP	-18.65	-18.59	-0.06	-18.29
**5**	MMPPMM	-0.27	-0.45	0.18	-0.24
**5**	MMPPMP	-7.64	-7.25	-0.39	-7.24
**5**	MMPPPM	-3.95	-4.35	0.4	-4.15
**5**	MMPPPP	-10.25	-10.31	0.06	-10.00

[a] The configurations are represented by their helical sequence (P or M), corresponding to the numbered nodes defined in **Figure S1**.
[b] All energy terms are reported in kcal/mol and defined relative to the all-homochiral configuration as the global reference state.

S4. Predicted Low-Energy Stereoisomers for MHs 6–9

To evaluate the thermodynamic stabilities of the computationally demanding giant MHs **6–9**, the CDHF model was applied across their expansive stereoisomeric spaces. Due to the vast number of possible configurations, the stereoisomers are sorted in ascending order of ΔH_{CDHF} (from most to least stable). The configurations and corresponding ΔH_{CDHF} for only the top 10-ranked stereoisomers of each MH are detailed in **Table S4** (featuring the predicted global minima discussed in **Sections 3.2** and **3.3** of the main text).

Table S4. ΔH_{CDHF} for the top 10-ranked stereoisomers of MHs **6–9**.

MH	Configuration[a]	Point Groups	$\Delta \boldsymbol{H_{CDHF}}$ (kcal/mol)
**6**	PMMPMMPMM	C_3	-21.12

	PPPPMMMMM	C_1	-19.22
	PPPPMMMMP	C_1	-18.48
	PPPMMPMPP	C_1	-18.48
	PPMPMMMMP	C_1	-17.74
	PPPPMMPMM	C_1	-14.08
	PPMPMMPMM	C_1	-13.34
	PMPPMMPMM	C_1	-13.30
	PPPPMMMPP	C_1	-12.90
	PPPMPPMPP	C_1	-12.90
**7**	PMMPPMMPPMMP	C_3	-41.97
	PPPMPMMPMMMP	C_1	-40.07
	PPPMPMMPMMPP	C_1	-36.83
	PPPMMMPPMPPM	C_1	-36.83
	PMMPPMMPPMMM	C_1	-35.02
	PPPMPMMPPMMP	C_1	-34.93
	PPPMPMMPMMPM	C_1	-34.88
	PPPMMMPMMPPM	C_1	-34.88
	PPPMPMMPMPPM	C_1	-33.75
	PPPMMPPMMPPM	C_1	-33.75
**8**	PPMMPPMMPPMMPPM	C_1	-56.14
	PPMMPPMMMPMMPPM	C_1	-55.77
	PPMMPPPMMPMMPPM	C_1	-52.32
	PPMMPPMMPMMMPPM	C_1	-51.81
	PMMPPPMMPPPMMPP	C_3	-51.60
	PMMPPPMMPPPMMMP	C_1	-51.23
	PMMPPPMMMPPMMMP	C_1	-50.86
	PMMMPPMMMPPMMMP	C_3	-50.49
	PPPMMPMMPPMMPPM	C_1	-49.88
	PPPMMMMPPMMPPMM	C_1	-49.88
**9**	PMMPPPMMPPPMMPP	C_3	-56.22
	PPMMPPMMPPMMPPM	C_1	-51.96
	PMMPPPMMPPPMMMP	C_1	-50.25
	PPPMMPMMPPMMPPM	C_1	-50.10
	PPPMMMMPPMMPPMM	C_1	-50.10
	PPMMPPMMPPMMPPP	C_1	-49.27
	PPMMPMMPPPMPPMM	C_1	-49.27
	PPPMMPMMPPMMMPP	C_1	-48.24
	PPMMPPMMPPPMMPP	C_1	-48.00
	PMMPPPMMPPPMMPM	C_1	-47.83

[a] The configurations are represented by their helical sequence (P or M), corresponding to the numbered nodes defined in **Figure S1**.

S5. DFT and sTDDFT Calculations of MHs 8 and 9

To validate the predictive accuracy of the CDHF model for larger systems, the five most stable stereoisomers of MHs **8** and **9** were evaluated. All selected MHs were fully optimized using DFT at the B3LYP-D3(BJ)/6-31G(d,p) level. The optimized 3D structures for the selected diastereomers of each MH are shown in **Section S6**. The MH configurations, ν_{min}, H, ΔH_{CDHF}, and ΔH_{DFT} are detailed in **Table S5** (corresponding to the energetically validated stereoisomers presented in **Section 3.3** and the predicted global minimum structures depicted in **Figure 5** of the main text).

Table S5. ΔH_{CDHF} and ΔH_{DFT} for the five most stable stereoisomers of MHs **8** and **9**.

**MH**	**Configuration**[a]	**Point Groups and Ranking**[b]	$\boldsymbol{\nu_{min}}$ (cm^{-1})	$\boldsymbol{H}$ (Hartree)	$\Delta\boldsymbol{H}$ (kcal/mol) $\boldsymbol{DFT}$[c]	$\boldsymbol{CDHF}$[c]
**8**	PPMMPPMMPPMMPPM	C_1, 1st	5.65	-5772.862118	0	0
**8**	PPMMPPMMMPMMPPM	C_1, 2nd	6.67	-5772.859220	1.82	0.37
**8**	PPMMPPPMMPMMPPM	C_1, 3rd	6.01	-5772.852617	5.96	3.82
**8**	PPMMPPMMPMMMPPM	C_1, 4th	6.33	-5772.856711	3.39	4.33
**8**	PMMPPPMMPPPMMPP	C_3, 5th	6.19	-5772.857257	3.05	4.54
**9**	PMMPPPMMPPPMMPP	C_3, 1st	6.09	-6233.707008	0	0
**9**	PPMMPPMMPPMMPPM	C_1, 2nd	6.10	-6233.701487	3.46	4.26
**9**	PMMPPPMMPPPMMMP	C_1, 3rd	4.19	-6233.695451	7.25	5.97
**9**	PPPMMPMMPPMMPPM	C_1, 4th	6.74	-6233.699362	4.80	6.12
**9**	PPPMMMMPPMMPPMM	C_1, 4th	7.04	-6233.700703	3.96	6.12

[a] The configurations are represented by their helical sequence (P or M), corresponding to the numbered nodes defined in **Figure S1**.
[b] The stereoisomers are sorted in ascending order of ΔH_{CDHF} (from most to least stable).
[c] The relative enthalpy is defined with the most stable stereoisomer as the global reference state.

To elucidate the relationship between geometric symmetry and chiroptical properties, we evaluated the lowest-energy C_1- and C_3-symmetric stereoisomers of MHs **8** and **9**. The optimized 3D structures for these selected diastereomers are provided in **Section S6**. Spectral simulations were performed using the simplified time-dependent density functional theory (sTDDFT) approach at the B3LYP-D3(BJ)/6-31G(d,p) level to calculate the excitation energies, oscillator strengths (f), and rotatory strengths (R). The continuous UV-vis absorption and electronic circular dichroism (ECD) spectral curves were generated by applying a frequency-weighted Gaussian broadening with a standard deviation (σ) of 0.15 eV to the calculated electronic transitions. Furthermore, to ensure numerical stability and avoid artifacts from the small-denominator effect, the absorption dissymmetry factor (g_{CD}) was strictly evaluated only in spectral regions where the UV-vis absorption intensity exceeded 1% of the global maximum absorption. The resulting simulated UV-vis (top), ECD (middle), and g_{CD} (bottom), alongside the underlying electronic transitions (f and R) that elucidate their physical origins, are collectively presented in **Figure S2** (corresponding directly to the spectral data presented in **Figure 6** and the g_{CD} data discussed in **Section 3.3** of the main text).

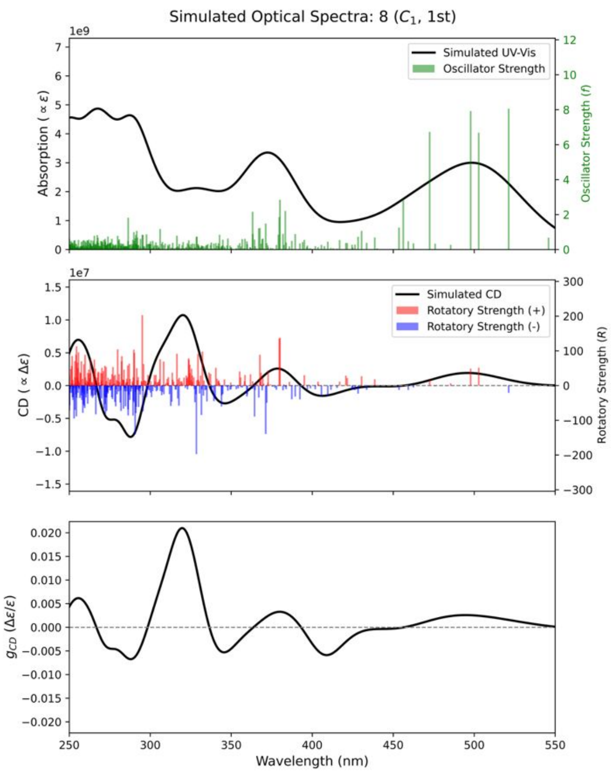
Simulated Optical Spectra: 8 (C_1, 1st)
Simulated UV-Vis
Oscillator Strength
Absorption (∝ ε)
Oscillator Strength (f)
Simulated CD
Rotatory Strength (+)
Rotatory Strength (-)
CD (∝ Δε)
Rotatory Strength (R)
g_{CD} (Δε/ε)
Wavelength (nm)

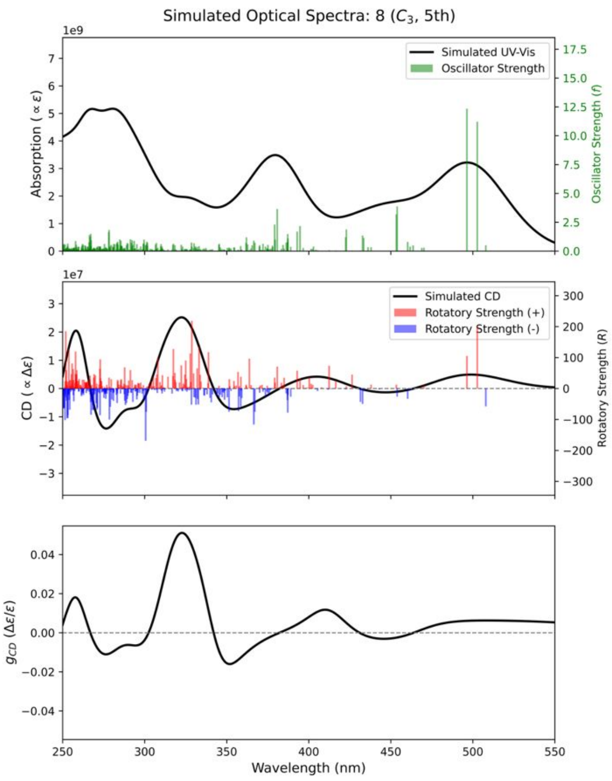
Simulated Optical Spectra: 8 (C_3, 5th)
Simulated UV-Vis
Oscillator Strength
Absorption (∝ ε)
Oscillator Strength (f)
Simulated CD
Rotatory Strength (+)
Rotatory Strength (-)
CD (∝ Δε)
Rotatory Strength (R)
g_{CD} (Δε/ε)
Wavelength (nm)

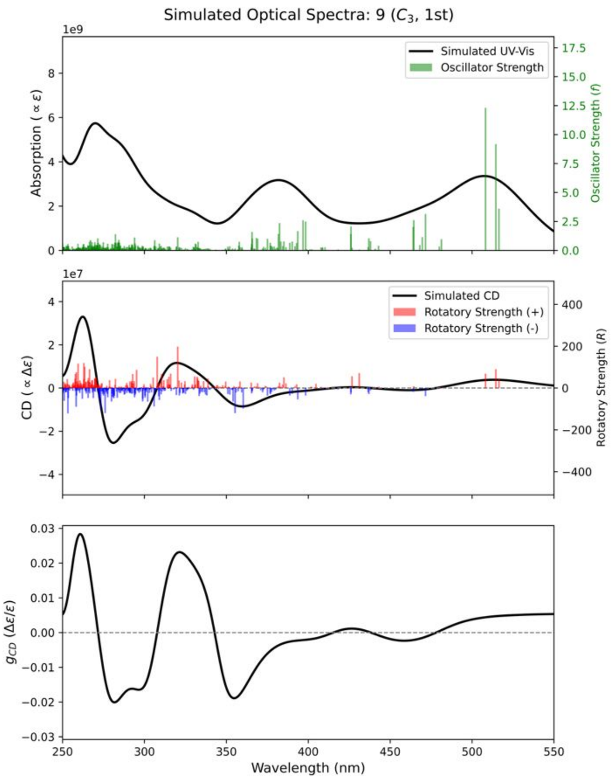
Simulated Optical Spectra: 9 (C_3, 1st)
Simulated UV-Vis
Oscillator Strength
Absorption (∝ ε)
Oscillator Strength (f)
Simulated CD
Rotatory Strength (+)
Rotatory Strength (-)
CD (∝ Δε)
Rotatory Strength (R)
g_{CD} (Δε/ε)
Wavelength (nm)

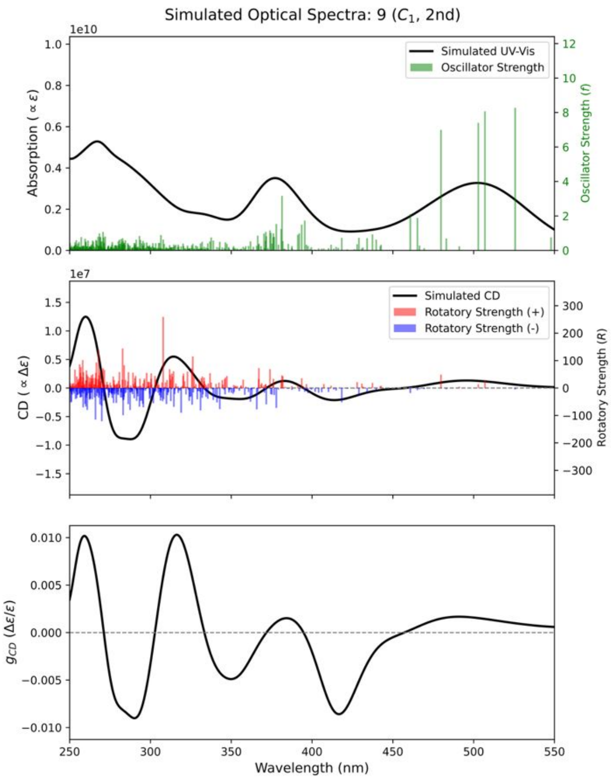
Simulated Optical Spectra: 9 (C_1, 2nd)
Simulated UV-Vis
Oscillator Strength
Absorption (∝ ε)
Oscillator Strength (f)
Simulated CD
Rotatory Strength (+)
Rotatory Strength (-)
CD (∝ Δε)
Rotatory Strength (R)
g_{CD} (Δε/ε)
Wavelength (nm)

Figure S2. Simulated UV-vis absorption (top), electronic circular dichroism (ECD, middle), and absorption dissymmetry factor (g_{CD}, bottom) spectra for the lowest-energy C_1- and C_3-symmetric stereoisomers of MHs **8** and **9**. The vertical bars in the background represent the calculated oscillator strengths (f, green) and rotatory strengths (R, red for positive, blue for negative) corresponding to individual electronic transitions.

S6. Cartesian Coordinates of Optimized Geometries

All geometry optimizations were performed at the B3LYP-D3(BJ)/6-31G(d,p) level of theory. All investigated species are neutral ground-state singlets (charge = 0, multiplicity = 1). Vibrational frequency analyses confirmed that all optimized structures are true minima with no imaginary frequencies. The optimized Cartesian coordinates for all CDHFs and the stereoisomers of MHs are listed below in standard XYZ format.

```
CDHF-1 in MH 1 (Homochiral)
54
C     -0.362433     1.292335    -0.462526
C      0.791330     1.944708     0.122132
C      2.013447     1.242547     0.251380
C      2.100493    -0.092892    -0.290655
C      0.916474    -0.803819    -0.544545
C     -0.291890    -0.070277    -0.838883
C      3.350109    -0.739331    -0.700889
C      0.982893    -2.259198    -0.385964
C      2.230156    -2.928068    -0.484640
C      3.404272    -2.149023    -0.851265
C      3.076005     1.837690     1.051719
C      0.738105     3.316596     0.524412
C      1.808995     3.943677     1.085611
C      2.986870     3.214943     1.415399
C     -1.377327    -0.628820    -1.634918
C     -1.603191     1.987453    -0.617522
C     -2.695227     1.394341    -1.174877
C     -2.602811     0.094063    -1.747521
C     -1.250316    -1.808588    -2.413478
C     -3.680902    -0.447038    -2.488810
C     -3.545286    -1.634985    -3.171891
C     -2.303356    -2.300732    -3.156266
C     -0.142787    -2.994648     0.048981
C     -0.056083    -4.348073     0.318162
C      1.168718    -5.016597     0.172404
C      2.293883    -4.308949    -0.212231
C      4.456655     0.026683    -1.132122
C      5.594662    -0.575881    -1.635681
C      5.663493    -1.973459    -1.737507
C      4.576564    -2.742178    -1.360189
C      4.153177     1.093958     1.600102
C      4.024929     3.820279     2.164125
C      5.095397     3.081691     2.616653
C      5.136700     1.697417     2.355984
H     -0.303340    -2.327432    -2.444376
H     -4.610231     0.114017    -2.532893
H     -4.373724    -2.037400    -3.746420
H     -2.165618    -3.202920    -3.744067
H     -1.084404    -2.480438     0.191557
H     -0.934394    -4.885604     0.661400
H      1.242079    -6.077653     0.389355
H      3.249638    -4.817928    -0.267130
```

H	4.400198	1.106429	-1.083740
H	6.425307	0.037768	-1.969807
H	6.552653	-2.449676	-2.138631
H	4.612716	-3.817370	-1.495091
H	4.199204	0.026434	1.441305
H	3.941515	4.877167	2.401632
H	5.881684	3.551662	3.199073
H	5.941673	1.093850	2.763598
H	-0.163270	3.889575	0.354680
H	1.753905	4.995980	1.348956
H	-1.696345	3.003024	-0.257614
H	-3.635804	1.932567	-1.248025

CDHF-1 in MH 1 (Heterochiral)
54

C	-0.617233	1.133020	-0.293354
C	0.597603	1.766549	0.181929
C	1.805410	1.038543	0.128979
C	1.710744	-0.412546	0.168483
C	0.535879	-1.025219	-0.291011
C	-0.542744	-0.186032	-0.789520
C	2.754426	-1.235010	0.777294
C	0.382384	-2.471917	-0.150274
C	1.523339	-3.279793	0.108150
C	2.743317	-2.643629	0.585177
C	3.042223	1.781640	0.015341
C	0.638729	3.151847	0.528954
C	1.823934	3.814315	0.666557
C	3.052282	3.172888	0.336218
C	-1.523375	-0.599385	-1.770493
C	-1.838171	1.860229	-0.440360
C	-2.876861	1.362971	-1.172720
C	-2.721811	0.161713	-1.922596
C	-1.303243	-1.671354	-2.674402
C	-3.699127	-0.248640	-2.862380
C	-3.477727	-1.329636	-3.686314
C	-2.252488	-2.025781	-3.608408
C	-0.881245	-3.108122	-0.201076
C	-1.002509	-4.482832	-0.177084
C	0.147493	-5.283212	-0.092221
C	1.381608	-4.685874	0.072215
H	-0.364181	-2.207760	-2.636505
H	-4.615773	0.329075	-2.942434
H	-4.226743	-1.627545	-4.413349
H	-2.050912	-2.842587	-4.294385
H	-1.774348	-2.503122	-0.260636
H	-1.986548	-4.938451	-0.222207
H	0.065827	-6.365600	-0.106044
H	2.249494	-5.317082	0.217249
C	4.234116	1.216619	-0.508770
C	4.262978	3.903569	0.252247
C	5.389354	1.959702	-0.619694
C	5.417106	3.309139	-0.207202
C	3.849467	-3.399010	1.037143
C	3.758117	-0.688954	1.613068
C	4.875171	-2.817989	1.756335
C	4.800153	-1.457071	2.091874
H	4.226832	0.186821	-0.841137
H	4.254045	4.954562	0.527458
H	6.282396	1.503613	-1.035417
H	6.336550	3.881358	-0.281652
H	3.881518	-4.466064	0.855340
H	3.708447	0.355848	1.883472
H	5.707996	-3.423528	2.100065
H	5.555642	-1.005631	2.727011
H	-0.287427	3.693374	0.675977
H	1.839210	4.863320	0.947685
H	-1.941398	2.830995	0.028239
H	-3.805069	1.920039	-1.260932

CDHF-2 in MH 1 (Homochiral)
54

C	-0.645437	0.882013	0.075872
C	0.547785	1.429133	0.631131
C	1.758449	0.833023	0.418614
C	1.838472	-0.451816	-0.180112
C	0.650190	-1.096515	-0.610320
C	-0.560836	-0.305975	-0.688238
C	3.103785	-1.103217	-0.318719
C	0.723419	-2.532165	-0.840717
C	2.003655	-3.150911	-0.987127
C	3.189638	-2.382077	-0.776128
C	-1.688228	-0.654576	-1.530489
C	-1.902154	1.598246	0.174254
C	-3.011639	1.214297	-0.612386
C	-2.945598	-0.037852	-1.337071
C	-1.542262	-1.542803	-2.648030
C	-4.109054	-0.742973	-1.857686
C	-3.938203	-1.698038	-2.903267
C	-2.616186	-1.989219	-3.355379
C	-2.040714	2.693167	1.084637
C	-3.177830	3.441316	1.137749
C	-4.205202	3.256000	0.169152
C	-4.097989	2.176273	-0.759369
C	-0.409184	-3.384582	-0.797159
C	-0.299324	-4.742854	-1.012145
C	0.955393	-5.326357	-1.276688
C	2.086658	-4.540002	-1.246465
H	-0.543837	-1.839368	-2.939590
H	-2.480185	-2.630073	-4.221264
H	-1.244200	2.898373	1.788362
H	-3.283764	4.234751	1.871767
H	-1.378789	-2.964509	-0.568331
H	-1.187439	-5.365137	-0.964163
C	-5.065153	-2.372639	-3.428395
C	-5.399935	-0.615023	-1.287400
C	-6.477565	-1.318347	-1.786097
C	-6.319411	-2.182197	-2.888159
C	-5.277119	4.175397	0.067396
C	-4.993238	2.183033	-1.860367

C	-6.177073	4.101747	-0.972376
C	-6.004165	3.115823	-1.964320
H	-4.915633	-3.073077	-4.245332
H	-5.537502	0.035429	-0.433656
H	-7.451190	-1.209398	-1.318678
H	-7.175226	-2.717645	-3.287317
H	-5.351340	4.966021	0.808867
H	-4.869836	1.456263	-2.649945
H	-6.987641	4.819507	-1.050681
H	-6.662896	3.094237	-2.826874
H	0.514243	2.382113	1.142525
H	2.674235	1.310934	0.753831
H	3.997755	-0.546372	-0.053075
H	4.153971	-2.864381	-0.906893
H	1.032878	-6.392912	-1.463270
H	3.069549	-4.981648	-1.385356

CDHF-2 in MH 1 (Heterochiral)

54

C	-0.534978	1.234889	-0.169616
C	0.678660	1.826745	0.332664
C	1.805617	0.994793	0.537059
C	1.843971	-0.280951	-0.085230
C	0.787117	-0.724104	-0.834133
C	-0.435723	-0.005081	-0.856635
C	2.841689	1.447614	1.451467
C	0.899897	3.262763	0.478920
C	2.024454	3.737035	1.187687
C	2.811758	2.769275	1.939611
C	-1.568421	-0.533684	-1.552201
C	-1.866253	1.778149	0.051883
C	-2.974004	1.225609	-0.660756
C	-2.777684	0.088004	-1.504486
C	-2.143746	2.763351	1.032468
C	-3.420918	3.249179	1.224801
C	-4.493463	2.766495	0.449609
C	-4.269232	1.761582	-0.466877
C	3.811777	0.538021	1.970488
C	3.524808	3.073427	3.158940
C	4.479103	2.137604	3.662842
C	4.638225	0.888855	2.999325
C	0.100119	4.189905	-0.260303
C	0.378546	5.523644	-0.272226
C	1.555522	6.028420	0.346790
C	2.428453	5.121039	1.021954
H	-1.338109	3.132460	1.652995
H	-3.599094	4.000248	1.988018
H	3.875823	-0.463234	1.562986
H	5.375776	0.186081	3.376018
H	-0.733132	3.816624	-0.837839
H	-0.253451	6.208931	-0.829789
C	3.708317	5.612232	1.394970
C	1.918115	7.390969	0.209368
C	4.051602	6.935958	1.222244
C	3.137990	7.847895	0.653931
C	3.246617	4.221432	3.947240
C	5.196799	2.436289	4.847724
C	4.940229	3.586599	5.559039
C	3.933949	4.471669	5.114578
H	4.438342	4.931989	1.809308
H	1.221783	8.062674	-0.284998
H	5.040661	7.273702	1.515889
H	3.410841	8.892003	0.536444
H	2.470180	4.903830	3.626907
H	5.935704	1.721703	5.199596
H	5.487128	3.800406	6.472036
H	3.692545	5.352709	5.701021
H	2.736702	-0.889776	-0.011949
H	0.848853	-1.665799	-1.371482
H	-1.436832	-1.447944	-2.123764
H	-3.629128	-0.307832	-2.050410
H	-5.493680	3.161890	0.596483
H	-5.093060	1.342044	-1.037588

CDHF-2 in MH 2 (Homochiral)

66

C	-0.469997	0.872754	0.040673
C	0.723249	1.428884	0.582800
C	1.931779	0.821267	0.387476
C	2.005275	-0.478339	-0.177492
C	0.813472	-1.130624	-0.589250
C	-0.394803	-0.339919	-0.684619
C	3.268746	-1.136503	-0.302994
C	0.883450	-2.572748	-0.783567
C	2.162012	-3.197512	-0.916980
C	3.350571	-2.426277	-0.728499
C	-1.533068	-0.717789	-1.506336
C	-1.722572	1.603689	0.118922
C	-2.824560	1.213635	-0.677573
C	-2.790317	-0.095405	-1.311574
C	-1.403778	-1.619115	-2.608148
C	-3.962994	-0.782398	-1.811756
C	-3.804110	-1.655137	-2.914655
C	-2.490960	-2.000040	-3.341479
C	-1.883329	2.691891	1.026152
C	-3.074393	3.356593	1.122723
C	-4.106145	3.127969	0.172969
C	-3.911427	2.156336	-0.838491
C	-0.250096	-3.422237	-0.715443
C	-0.143059	-4.786009	-0.894737
C	1.109932	-5.378416	-1.146773
C	2.242250	-4.593034	-1.140138
H	-0.416445	-1.959623	-2.888285
H	-2.369180	-2.617144	-4.226554
H	-1.084342	2.936099	1.714639
H	-3.226612	4.107846	1.892105
H	-1.219125	-2.995042	-0.497253
H	-1.032194	-5.405143	-0.828488
C	-4.953237	-2.139939	-3.612273
C	-5.288611	-0.630486	-1.246562

C -6.415981 -1.082681 -1.994099
C -6.211279 -1.808657 -3.207788
C -5.333823 3.860365 0.232039
C -4.804255 2.191726 -1.981251
C -6.302085 3.681801 -0.708659
C -6.043920 2.886945 -1.869052
H -4.797162 -2.759108 -4.491062
C -5.522332 -0.099284 0.044614
C -7.720367 -0.851711 -1.496662
H -7.080749 -2.141431 -3.767284
H -5.483449 4.544753 1.062229
C -4.474501 1.629808 -3.237372
H -7.253327 4.200729 -0.634241
C -6.967972 2.840762 -2.939383
H 0.691509 2.395921 1.067936
H 2.849903 1.303875 0.709282
H 4.164641 -0.575222 -0.053629
H 4.313408 -2.914100 -0.849399
H 1.185336 -6.449596 -1.305644
H 3.224201 -5.039513 -1.269970
C -5.378373 1.625405 -4.279575
C -6.654121 2.200949 -4.119428
C -7.916738 -0.253172 -0.270872
C -6.802875 0.095145 0.518709
H -4.679285 0.154740 0.672599
H -8.567490 -1.175613 -2.094848
H -3.490779 1.205666 -3.385164
H -7.922716 3.345708 -2.821966
H -5.099842 1.178750 -5.228520
H -7.369692 2.174150 -4.935332
H -8.922256 -0.081037 0.100285
H -6.950193 0.513622 1.509208

CDHF-2 in MH 2 (Heterochiral)
66
C -0.598708 1.131868 -0.236523
C 0.607483 1.689481 0.319932
C 1.691067 0.818651 0.593671
C 1.718506 -0.462522 -0.016218
C 0.689750 -0.874871 -0.820641
C -0.503871 -0.114300 -0.913894
C 2.696642 1.242680 1.555929
C 0.870792 3.119349 0.461744
C 2.003442 3.554543 1.191067
C 2.679179 2.572611 2.034948
C -1.613942 -0.606176 -1.670559
C -1.923466 1.713137 -0.081298
C -3.008645 1.196455 -0.852718
C -2.802964 0.054830 -1.688999
C -2.223507 2.696596 0.894254
C -3.494474 3.217277 1.026227
C -4.538670 2.772926 0.191462
C -4.296167 1.769020 -0.721453
C 3.638711 0.329936 2.103264
C 3.366612 2.891373 3.256473
C 4.375743 1.999286 3.702644
C 4.501404 0.724988 3.090976
C 0.107689 4.086341 -0.254420
C 0.378745 5.420974 -0.135330
C 1.541870 5.872180 0.541548
C 2.453557 4.917368 1.057162
H -1.440198 3.036303 1.558549
H -3.690803 3.965608 1.787718
H 3.681727 -0.688183 1.736668
H 5.262138 0.043206 3.460071
H -0.712836 3.759824 -0.876665
H -0.262113 6.154571 -0.616024
C 3.782118 5.387125 1.415250
C 1.804260 7.270263 0.694252
C 3.984891 6.782580 1.629452
C 2.943923 7.706072 1.297561
C 3.108748 4.080746 4.050426
C 5.278490 2.399436 4.738006
C 5.172671 3.621924 5.325960
C 4.066955 4.479478 5.028218
C 4.912504 4.540541 1.500248
H 1.053686 7.973947 0.345716
C 5.239724 7.243450 2.091700
H 3.115676 8.765354 1.465499
C 1.925443 4.847822 3.938766
H 6.072653 1.713873 5.019644
H 5.890835 3.942757 6.074989
C 3.882023 5.690499 5.734662
H 2.583499 -1.101594 0.112430
H 0.748122 -1.822097 -1.348527
H -1.481370 -1.525159 -2.234291
H -3.635721 -0.313368 -2.281106
H -5.533458 3.195945 0.290430
H -5.101270 1.377361 -1.336939
C 1.754619 6.014365 4.656437
C 2.755905 6.461644 5.539161
C 6.137575 5.017278 1.921076
C 6.296369 6.374195 2.261267
H 4.819268 3.501345 1.217984
H 5.362437 8.305702 2.283924
H 1.135708 4.511591 3.281729
H 4.639016 5.991574 6.453438
H 0.838748 6.585152 4.540846
H 2.625097 7.389896 6.086589
H 6.982638 4.339260 1.983836
H 7.255664 6.739152 2.614842

CDHF-1 in MH 4 (Homochiral)
90
C -1.125072 2.091854 1.705731
C -2.395145 1.473674 1.619328
C -2.524193 0.150500 1.040750
C -1.366158 -0.543713 0.604665
C -0.074796 0.021181 0.926604
C 0.021188 1.406025 1.146458

C	-1.526247	-1.751180	-0.177363
C	-3.780049	-0.518434	0.977766
C	-3.875682	-1.800320	0.512941
C	-2.764608	-2.437147	-0.098922
C	-3.547979	2.194161	2.063594
C	-3.450124	3.434980	2.616412
C	-2.176472	4.009934	2.890670
C	-0.997314	3.311825	2.492130
C	1.291013	2.031588	0.778263
C	2.472305	1.270588	0.864269
C	2.387847	-0.133369	1.235726
C	1.122520	-0.775364	1.171393
C	-2.898864	-3.746291	-0.659206
C	-1.872487	-4.327251	-1.338352
C	-0.684241	-3.585612	-1.626222
C	-0.532768	-2.265064	-1.106415
C	1.351507	3.354906	0.190911
C	2.624917	3.949200	-0.059239
C	3.804634	3.188872	0.175467
C	3.725317	1.885359	0.572567
C	0.206035	4.088757	-0.211413
C	0.313230	5.353146	-0.743286
C	1.570799	5.979645	-0.919182
C	2.700557	5.260781	-0.587874
C	-2.073316	5.227187	3.605419
C	0.241501	3.817476	2.960301
C	3.517830	-0.889010	1.611252
H	-0.774280	3.645307	-0.108786
H	-0.587761	5.884150	-1.038789
C	1.655879	7.357068	-1.528106
C	0.550558	-1.498621	-1.602244
C	1.483566	-2.038832	-2.459249
C	1.399802	-3.383369	-2.888256
C	0.307797	-4.123675	-2.478792
C	1.038860	-2.144068	1.509376
H	3.684887	5.694322	-0.749464
H	0.641716	-0.459002	-1.319584
H	2.300232	-1.417282	-2.816367
H	0.181890	-5.143049	-2.836559
C	-0.846077	5.725584	3.982521
C	0.317367	4.991600	3.680214
C	2.161875	-2.864433	1.867611
C	3.414730	-2.234707	1.912602
H	-2.988623	5.747421	3.873864
H	1.149931	3.266012	2.768342
H	4.483949	-0.404895	1.690977
H	0.072039	-2.628939	1.512801
H	-0.776483	6.657005	4.535725
H	1.284871	5.344840	4.022883
H	2.067000	-3.912829	2.131990
H	4.298153	-2.794267	2.203907
C	1.473173	7.339786	-3.055653
C	1.536166	8.733467	-3.685020
C	1.356502	8.709193	-5.204213
H	2.624947	7.809109	-1.285863
H	0.888216	8.004256	-1.084379
H	0.511335	6.869176	-3.298332
H	2.244793	6.696569	-3.498412
H	2.498410	9.200027	-3.434775
H	0.763769	9.369455	-3.232097
H	2.135189	8.105373	-5.683244
H	1.405056	9.715949	-5.630953
H	0.388424	8.276095	-5.479394
C	2.428692	-3.946484	-3.836829
C	2.204353	-3.493379	-5.289903
C	3.253906	-4.041549	-6.259626
C	3.024743	-3.589013	-7.703067
H	2.410482	-5.041921	-3.793759
H	3.432194	-3.636793	-3.517498
H	2.203903	-2.396162	-5.329144
H	1.202336	-3.807888	-5.609626
H	3.251824	-5.138807	-6.212310
H	4.252023	-3.725498	-5.927794
H	2.046339	-3.919714	-8.068831
H	3.786905	-3.992903	-8.376882
H	3.053651	-2.496625	-7.782516
H	-4.670023	-0.036874	1.359967
H	-4.824095	-2.328338	0.549586
H	-4.531208	1.764130	1.927474
H	-4.343612	3.979545	2.907793
H	-3.840504	-4.269432	-0.519300
H	-1.966066	-5.334288	-1.734588
H	4.769472	3.646255	-0.024089
H	4.633541	1.300924	0.654376

CDHF-1 in MH 4 (Heterochiral)

90

C	-0.366206	2.078331	1.388334
C	-1.676137	1.558718	1.383672
C	-1.911738	0.254203	0.796057
C	-0.787710	-0.498585	0.365373
C	0.368777	0.260151	-0.088617
C	0.552742	1.571249	0.380847
C	-0.905035	-1.935777	0.350981
C	-3.196894	-0.349843	0.765134
C	-3.342488	-1.664264	0.409257
C	-2.205218	-2.498407	0.251646
C	-2.699641	2.258192	2.094647
C	-2.408314	3.345570	2.866777
C	-1.057071	3.744075	3.083167
C	-0.015152	3.067166	2.382234
C	1.639112	2.378641	-0.156634
C	2.743764	1.714338	-0.726317
C	2.558858	0.368927	-1.242372
C	1.291915	-0.260080	-1.089308
C	-2.360392	-3.901430	0.015310
C	-1.281305	-4.728823	-0.042611
C	0.030083	-4.233877	0.245176
C	0.217188	-2.844540	0.503426
C	1.492863	-2.431927	0.956536

C	2.543379	-3.317878	1.047986
C	2.389438	-4.680348	0.703051
C	1.132884	-5.115078	0.326186
C	1.643118	3.826971	-0.152360
C	2.876563	4.518993	-0.332222
C	4.048151	3.769912	-0.642743
C	3.966594	2.431673	-0.899811
C	0.468871	4.611868	-0.033433
C	0.528391	5.985704	0.014010
C	1.763205	6.675261	-0.053942
C	2.910086	5.931208	-0.244234
C	-0.730129	4.763818	4.009434
C	1.324380	3.366591	2.735190
C	3.533472	-0.258498	-2.051652
C	0.992382	-1.369286	-1.910422
H	1.646262	-1.399352	1.239174
H	3.511424	-2.964341	1.392545
C	3.551524	-5.633799	0.829593
H	0.970915	-6.166835	0.101255
H	-0.494643	4.122344	0.012407
H	-0.391649	6.556320	0.107606
C	1.795152	8.182541	-0.004101
H	3.868933	6.432933	-0.353297
C	1.946536	-1.939097	-2.729046
C	3.244760	-1.406310	-2.763964
C	1.617504	4.344659	3.660201
C	0.583428	5.073171	4.284948
H	-1.538757	5.283823	4.515698
H	2.129290	2.810902	2.272306
H	4.512231	0.192002	-2.161931
H	-0.004333	-1.786964	-1.889496
H	1.689972	-2.798326	-3.340223
H	4.007849	-1.864240	-3.385688
H	2.652798	4.552519	3.911372
H	0.824388	5.853981	4.999620
C	3.805594	-6.067007	2.283832
C	4.993926	-7.020651	2.428319
C	5.240398	-7.449711	3.876066
C	1.384980	8.828769	-1.338604
C	1.394549	10.358606	-1.293993
C	0.989023	10.995830	-2.624513
H	4.459993	-5.161249	0.434219
H	3.368348	-6.523363	0.215304
H	2.802233	8.521684	0.266040
H	1.122022	8.538589	0.786629
H	2.897456	-6.542289	2.676945
H	3.971692	-5.174709	2.901633
H	5.895367	-6.537560	2.027957
H	4.822995	-7.908291	1.804538
H	5.444792	-6.582897	4.514277
H	6.094164	-8.130189	3.953960
H	4.365102	-7.963236	4.289041
H	0.384010	8.474638	-1.618196
H	2.061934	8.475445	-2.127293
H	2.396902	10.705644	-1.009222
H	0.717994	10.700914	-0.499400
H	1.668942	10.694229	-3.428969
H	1.003139	12.088827	-2.567780
H	-0.021777	10.689519	-2.915969
H	-4.075160	0.239507	0.998530
H	-4.330894	-2.105703	0.321526
H	-3.723376	1.909262	2.038454
H	-3.199577	3.877436	3.387315
H	-3.365629	-4.289644	-0.121861
H	-1.403625	-5.788607	-0.247073
H	4.994135	4.294097	-0.744196
H	4.852463	1.901374	-1.227242

CDHF-2 in MH 4 (Homochiral)
72

C	-0.860489	2.104050	0.975271
C	-2.062808	1.483602	1.168875
C	-2.204445	0.091715	0.926937
C	-1.073494	-0.662461	0.497073
C	0.233951	-0.065313	0.690461
C	0.321461	1.340130	0.752958
C	-1.335284	-1.950375	-0.115584
C	-3.461892	-0.551806	1.110230
C	-3.604094	-1.882263	0.842774
C	-2.561704	-2.597723	0.185879
C	1.615230	2.002622	0.673949
C	2.807599	1.273125	0.872464
C	2.726259	-0.184034	0.828874
C	1.459450	-0.835193	0.864139
C	-2.771191	-3.954970	-0.214894
C	-1.845918	-4.621594	-0.958534
C	-0.698000	-3.937234	-1.465545
C	-0.472442	-2.576526	-1.101802
C	1.688531	3.378009	0.316214
C	2.898580	3.993212	0.153596
C	4.094192	3.341676	0.551534
C	4.036153	2.015444	1.057871
C	3.875570	-0.994677	0.657598
C	0.561423	-1.887558	-1.781413
C	1.384965	-2.529171	-2.679039
C	1.232659	-3.905613	-2.965055
C	0.183613	-4.577326	-2.367733
C	1.440313	-2.232403	1.091717
H	0.705209	-0.832067	-1.597055
H	2.170693	-1.967577	-3.177007
H	0.006286	-5.623406	-2.607122
C	2.591557	-2.989760	1.052093
C	3.814608	-2.369862	0.747887
H	4.826755	-0.519180	0.464716
H	0.493606	-2.718421	1.278685
H	2.540488	-4.061353	1.215544
H	4.714649	-2.961205	0.612536
C	5.189619	1.551885	1.818405
C	6.426315	2.259465	1.715152
C	6.485770	3.477319	0.968821

C	5.346327	4.029128	0.471170
C	5.115756	0.508677	2.775711
C	7.558821	1.802837	2.431558
H	7.438545	3.991847	0.884425
H	5.362451	5.007082	-0.001354
C	2.142633	-4.582104	-3.959927
C	1.764763	-4.266604	-5.417536
C	2.692786	-4.931617	-6.436821
C	2.310670	-4.616401	-7.884391
H	2.115166	-5.667541	-3.807280
H	3.179204	-4.266711	-3.783912
H	1.774830	-3.178642	-5.564799
H	0.729324	-4.586146	-5.593397
H	2.681461	-6.018696	-6.280725
H	3.725660	-4.608919	-6.249101
H	1.293407	-4.957474	-8.106356
H	2.987859	-5.102236	-8.593912
H	2.345037	-3.537965	-8.074782
H	-0.784739	3.175656	1.111828
H	-2.936503	2.052090	1.473621
H	-4.290883	0.032827	1.498136
H	-4.538870	-2.396023	1.047111
H	-3.690736	-4.443465	0.094630
H	-1.997534	-5.661074	-1.234899
H	0.780016	3.913063	0.068826
H	2.956857	5.007888	-0.229015
C	7.469240	0.734636	3.297323
C	6.223030	0.107290	3.493907
H	4.165466	0.025617	2.957026
H	8.496164	2.339068	2.311925
H	8.341293	0.402310	3.851827
H	6.127068	-0.690980	4.223247

CDHF-2 in MH 4 (Heterochiral)
72

C	-0.725570	2.452709	0.491682
C	-1.982896	1.923560	0.360662
C	-2.169768	0.547119	0.068977
C	-1.027469	-0.292363	-0.070079
C	0.232363	0.216011	0.398104
C	0.419781	1.609999	0.519252
C	-1.209326	-1.601169	-0.660364
C	-3.474334	-0.011707	-0.064393
C	-3.634496	-1.341604	-0.327870
C	-2.512391	-2.159241	-0.657339
C	1.779022	2.139287	0.595590
C	2.873640	1.243044	0.562514
C	2.609261	-0.120630	1.013056
C	1.294084	-0.636476	0.921495
C	-2.697850	-3.529382	-1.020287
C	-1.650135	-4.297177	-1.434098
C	-0.362310	-3.714937	-1.644516
C	-0.155417	-2.341689	-1.325891
C	2.042138	3.534310	0.622495
C	3.327676	4.006649	0.582917
C	4.417305	3.130919	0.345812
C	4.179263	1.735171	0.223823
C	3.594585	-0.882220	1.681206
C	5.744475	3.647517	0.209597
C	5.247992	0.909303	-0.324030
C	1.059963	-1.754919	-1.762063
C	2.046294	-2.505820	-2.357585
C	1.897288	-3.900112	-2.562110
C	0.687226	-4.471938	-2.223552
C	1.019575	-1.908708	1.469499
H	1.206313	-0.689389	-1.649236
H	2.958292	-2.017112	-2.691439
C	3.050111	-4.673051	-3.166063
H	0.502280	-5.523384	-2.417829
C	2.008490	-2.652622	2.081749
C	3.307882	-2.131774	2.194242
H	4.585213	-0.464371	1.806519
H	0.007688	-2.290372	1.431595
H	1.769760	-3.623393	2.504626
H	4.080155	-2.694124	2.709698
C	6.559286	1.461017	-0.446114
C	6.785465	2.835027	-0.117339
H	5.900586	4.709490	0.376364
C	5.031196	-0.385357	-0.859730
C	7.609280	0.666607	-0.965720
H	7.793857	3.229805	-0.201541
C	2.808803	-6.158364	-3.434107
C	4.030018	-6.854436	-4.041295
C	3.794924	-8.341893	-4.310208
H	3.919655	-4.567987	-2.500857
H	3.345449	-4.182129	-4.104117
H	1.951637	-6.276598	-4.110118
H	2.534565	-6.662449	-2.497997
H	4.887777	-6.733066	-3.366381
H	4.305817	-6.349855	-4.976815
H	3.548813	-8.875389	-3.385352
H	4.680778	-8.816629	-4.743361
H	2.962600	-8.489483	-5.007322
H	-0.615526	3.521649	0.625188
H	-2.857812	2.562612	0.436714
H	-4.334218	0.633720	0.089340
H	-4.625113	-1.786016	-0.358234
H	-3.697220	-3.949033	-0.948095
H	-1.792668	-5.346719	-1.675278
H	1.223762	4.238965	0.701590
H	3.524035	5.071401	0.668428
C	7.375790	-0.613375	-1.420884
C	6.065326	-1.128899	-1.390844
H	4.033783	-0.801298	-0.862823
H	8.603758	1.100056	-1.026043
H	8.188033	-1.208419	-1.826603
H	5.860199	-2.115690	-1.795251

CDHF-3 in MH 4 (Homochiral)
72

C	-0.955251	2.126033	1.034487
C	-2.171945	1.531636	0.837218
C	-2.267879	0.150651	0.520785
C	-1.081134	-0.615823	0.434236
C	0.153409	-0.057959	0.932600
C	0.228300	1.349442	1.104928
C	-1.171817	-1.953639	-0.135886
C	-3.548070	-0.484794	0.248281
C	-3.603889	-1.899598	0.092522
C	-2.365647	-2.665009	0.025959
C	1.485908	1.977516	1.374346
C	2.616917	1.240916	1.538780
C	2.551335	-0.187717	1.579107
C	1.306714	-0.847896	1.344481
C	-2.299837	-4.120077	-0.031072
C	-1.271707	-4.720639	-0.786044
C	-0.244454	-3.886059	-1.389412
C	-0.142443	-2.529545	-0.986272
C	-4.744754	0.257651	0.140624
C	-4.850546	-2.493036	-0.221842
C	3.695311	-0.945595	1.922282
C	-3.164295	-4.967264	0.768453
C	-1.251757	-6.139656	-0.933296
C	0.875210	-1.736658	-1.566494
C	1.764455	-2.264462	-2.475299
C	1.688352	-3.616497	-2.871355
C	0.679497	-4.394421	-2.329713
C	1.248033	-2.237599	1.609995
H	0.938581	-0.687371	-1.313094
H	2.528222	-1.624536	-2.909421
C	2.692180	-4.144404	-3.875149
H	0.583650	-5.421252	-2.654452
C	2.370798	-2.952155	1.975366
C	3.616903	-2.310198	2.101633
C	-5.955298	-0.353034	-0.124334
C	-6.004236	-1.741583	-0.321845
H	-4.719340	1.335076	0.246015
H	-4.893356	-3.555327	-0.417820
H	4.636200	-0.422756	2.070309
H	0.299337	-2.751838	1.547322
H	2.285216	-4.015889	2.173562
H	4.500328	-2.880350	2.371714
H	-6.858021	0.244448	-0.204092
H	-6.942220	-2.226240	-0.573874
C	-3.130227	-6.382392	0.574650
C	-2.177033	-6.936170	-0.324173
C	-3.976642	-4.473743	1.824066
H	-0.476407	-6.600326	-1.532228
C	-3.985917	-7.219646	1.332871
H	-2.154435	-8.013061	-0.464440
C	2.538881	-5.607891	-4.289667
C	3.599606	-6.048930	-5.302069
C	3.451095	-7.513100	-5.719558
H	3.701143	-3.994290	-3.465288
H	2.650877	-3.512875	-4.773847
H	1.541368	-5.767877	-4.720075
H	2.598402	-6.251584	-3.401908
H	4.597621	-5.887124	-4.873567
H	3.541995	-5.404944	-6.189619
H	3.536907	-8.180158	-4.854523
H	4.219230	-7.804434	-6.442612
H	2.473466	-7.694348	-6.179915
H	-0.886729	3.196844	1.202642
H	-3.068212	2.135779	0.896015
H	1.514122	3.061800	1.432733
H	3.575262	1.722168	1.711395
C	-4.778790	-5.312184	2.567623
C	-4.809041	-6.698037	2.304768
H	-3.956351	-3.418643	2.059542
H	-3.956984	-8.289961	1.148233
H	-5.382703	-4.901237	3.370682
H	-5.453893	-7.349067	2.886739

CDHF-3 in MH 4 (Heterochiral)
72

C	-0.858197	2.354719	0.498638
C	-2.098777	1.793366	0.649250
C	-2.273692	0.388137	0.553872
C	-1.163842	-0.424354	0.216024
C	0.154496	0.122010	0.433661
C	0.298390	1.534583	0.446706
C	-1.442960	-1.726639	-0.380092
C	-3.517579	-0.271570	0.913623
C	-3.640775	-1.673217	0.711378
C	-2.688907	-2.335432	-0.174814
C	1.604796	2.120152	0.423410
C	2.723265	1.346417	0.465933
C	2.620537	-0.067984	0.658314
C	1.333660	-0.680694	0.711736
C	-2.960764	-3.632878	-0.776076
C	-1.861881	-4.433988	-1.154121
C	-0.596101	-3.786119	-1.451670
C	-0.481322	-2.387294	-1.250347
C	-4.523827	0.403228	1.640965
C	-4.658618	-2.363602	1.405948
C	3.778045	-0.857883	0.852031
C	0.580806	-1.711899	-1.891106
C	1.571124	-2.400972	-2.553867
C	1.556711	-3.810825	-2.618986
C	0.460075	-4.473129	-2.094974
C	1.266421	-2.040699	1.097169
H	0.622915	-0.632024	-1.849220
H	2.381927	-1.853441	-3.026616
C	2.711359	-4.524389	-3.290087
H	0.386969	-5.544104	-2.224827
C	2.408219	-2.786661	1.302845
C	3.679814	-2.200705	1.149716
C	-5.562823	-0.287631	2.235496
C	-5.605493	-1.688563	2.152207
H	-4.446618	1.472644	1.796999

H	-4.696990	-3.442865	1.347794
H	4.750387	-0.377812	0.783397
H	0.298680	-2.503406	1.234755
H	2.322522	-3.829818	1.590118
H	4.575519	-2.795912	1.298291
H	-6.313050	0.249431	2.807484
H	-6.376422	-2.244413	2.676294
C	-4.290798	-4.148807	-1.040547
C	-2.062030	-5.831393	-1.366601
C	-3.310969	-6.381491	-1.354336
C	-4.462632	-5.547283	-1.275600
C	-5.428696	-3.314595	-1.193971
H	-1.203337	-6.471287	-1.529350
H	-3.445083	-7.450987	-1.488371
C	-5.760305	-6.068897	-1.500404
C	2.594785	-6.043306	-3.417070
C	3.810883	-6.673997	-4.101038
C	3.699963	-8.194427	-4.228110
H	3.629634	-4.283207	-2.735620
H	2.856868	-4.091230	-4.289557
H	1.689235	-6.298648	-3.983394
H	2.471079	-6.488735	-2.420937
H	4.716959	-6.416264	-3.536692
H	3.936606	-6.229095	-5.097043
H	3.605520	-8.666277	-3.243687
H	4.579514	-8.621720	-4.719568
H	2.819160	-8.477471	-4.815142
H	-0.736260	3.433854	0.493950
H	-2.961641	2.431292	0.799399
H	1.679794	3.202173	0.363616
H	3.711705	1.794400	0.419497
C	-6.854048	-5.235835	-1.578666
C	-6.674898	-3.841791	-1.457800
H	-5.310331	-2.241911	-1.123314
H	-5.869316	-7.140060	-1.646151
H	-7.842451	-5.643446	-1.766649
H	-7.522586	-3.175154	-1.582179

CDHF-6 in MH 7 (Homochiral)
54

C	-2.247145	2.796127	1.061827
C	-0.861414	3.198787	0.939024
C	0.144893	2.205781	0.932094
C	-0.183841	0.827962	0.623086
C	-1.544392	0.442436	0.674713
C	-2.581953	1.445308	0.815810
C	-0.422419	4.578470	0.779015
C	0.922828	4.926322	1.101373
C	1.821173	3.891187	1.499405
C	1.464827	2.586289	1.346882
C	-3.300399	3.669944	1.566132
C	-4.665054	3.275230	1.419786
C	-4.960441	1.982082	0.901604
C	-3.959989	1.082492	0.688462
C	-1.239758	5.579102	0.197022
C	-0.784605	6.871654	0.033237
C	0.512124	7.231458	0.451398
C	1.351928	6.267591	0.968096
C	0.808956	-0.172548	0.290098
C	0.472381	-1.535987	0.491383
C	-0.880417	-1.893085	0.732421
C	-1.864562	-0.945691	0.720166
C	2.093703	0.105684	-0.335601
C	3.085611	-0.922641	-0.379575
C	2.760045	-2.239728	0.069307
C	1.481483	-2.546550	0.420390
H	2.813654	4.155736	1.851465
H	2.179292	1.803607	1.563133
H	-5.998330	1.701134	0.748602
H	-4.214433	0.082931	0.360018
H	-2.234415	5.318009	-0.139734
H	-1.429685	7.612392	-0.428806
H	0.856667	8.254535	0.337098
H	2.371781	6.515720	1.248608
H	-1.126671	-2.941358	0.873578
H	-2.893288	-1.255722	0.848265
H	3.530500	-3.004839	0.043524
H	1.200380	-3.568084	0.659732
C	-3.047761	4.844559	2.321290
C	-4.074489	5.640341	2.785433
C	-5.418071	5.305025	2.524153
C	-5.702443	4.129705	1.865212
H	-2.029307	5.113592	2.560491
H	-3.840980	6.525950	3.368157
H	-6.219782	5.947310	2.874961
H	-6.731288	3.817503	1.709059
C	2.375112	1.310210	-1.029326
C	3.602531	1.534673	-1.617753
C	4.617524	0.559972	-1.550240
C	4.351083	-0.653498	-0.953957
H	1.603008	2.061760	-1.118893
H	3.779484	2.466041	-2.146461
H	5.587390	0.749967	-1.999269
H	5.099392	-1.441156	-0.947236

CDHF-6 in MH 7 (Heterochiral)
54

C	-2.082382	2.867293	-0.014865
C	-0.731768	3.322848	0.285272
C	0.289084	2.351330	0.362240
C	-0.050008	0.930975	0.360435
C	-1.383454	0.604382	0.705611
C	-2.430345	1.568048	0.405724
C	-0.426433	4.694693	0.647172
C	0.935737	5.081082	0.837967
C	1.947777	4.081922	0.810902
C	1.627775	2.767906	0.645123
C	-3.060537	3.645809	-0.739296
C	-4.425980	3.226357	-0.740917
C	-4.772909	2.002289	-0.103523

C	-3.804357	1.181024	0.398507
C	-1.420888	5.666248	0.939724
C	-1.087928	6.960475	1.277451
C	0.262800	7.359034	1.354789
C	1.253596	6.424833	1.154512
C	0.906156	-0.136221	0.211058
C	0.620986	-1.377933	0.840519
C	-0.665663	-1.605554	1.393317
C	-1.661851	-0.677729	1.248669
C	2.103916	-0.056415	-0.610810
C	3.081082	-1.094685	-0.524569
C	2.826602	-2.241587	0.290547
C	1.620636	-2.399379	0.900329
H	2.975043	4.373349	1.010173
H	2.397763	2.017114	0.748279
H	-5.814401	1.694136	-0.093361
H	-4.084988	0.203288	0.769934
H	-2.463446	5.383856	0.913714
H	-1.875658	7.674168	1.498035
H	0.515660	8.384491	1.605612
H	2.300046	6.694595	1.266908
H	-0.864469	-2.556511	1.878603
H	-2.659640	-0.908821	1.600849
H	3.588575	-3.012835	0.355693
H	1.391300	-3.308098	1.449426
C	-5.395336	3.994608	-1.432555
C	-2.712168	4.762009	-1.545207
C	-3.669070	5.471768	-2.236804
C	-5.030269	5.104627	-2.160286
H	-6.432009	3.671075	-1.399957
H	-1.671851	5.049497	-1.624156
H	-3.371250	6.314906	-2.852306
H	-5.778864	5.679580	-2.696390
C	2.303336	0.951372	-1.587428
C	3.446835	0.991384	-2.358785
C	4.450791	0.016180	-2.199656
C	4.258715	-1.014839	-1.305134
H	1.535411	1.697833	-1.740561
H	3.565170	1.774565	-3.101061
H	5.353953	0.060347	-2.800231
H	4.999455	-1.803365	-1.205398

CDHF-7 in MH 7 (Homochiral)
60

C	-2.308200	2.818449	1.120498
C	-0.961381	3.276418	0.858939
C	0.091122	2.330292	0.834322
C	-0.196850	0.928330	0.613537
C	-1.536478	0.491588	0.712897
C	-2.602660	1.448316	0.936719
C	-0.617159	4.660900	0.572976
C	0.730003	5.098359	0.740257
C	1.711660	4.141777	1.132832
C	1.415129	2.811559	1.113957
C	-3.346364	3.668347	1.690682
C	-4.699613	3.214365	1.688729
C	-4.984489	1.893909	1.238372
C	-3.971588	1.030194	0.949224
C	-1.542705	5.579211	0.016189
C	-1.179091	6.878706	-0.270532
C	0.130242	7.330553	-0.007895
C	1.068767	6.447382	0.481004
C	0.822415	-0.033989	0.221756
C	0.509622	-1.427840	0.256041
C	-0.824154	-1.834575	0.538628
C	-1.814289	-0.911818	0.691007
C	2.090928	0.323015	-0.304083
C	3.049259	-0.615574	-0.648409
C	2.755772	-1.987471	-0.483154
C	1.505012	-2.380059	-0.064238
C	4.401090	-0.179931	-1.158897
C	5.440366	-0.055107	-0.031174
C	6.821070	0.378043	-0.529458
C	7.847485	0.506606	0.597782
H	2.709952	4.481333	1.392764
H	2.178348	2.092411	1.377944
H	-6.018575	1.564221	1.197948
H	-4.218921	0.012767	0.677095
H	-2.548657	5.246499	-0.203443
H	-1.906577	7.554073	-0.709815
H	0.403346	8.359742	-0.219118
H	2.094816	6.765059	0.644468
H	-1.049979	-2.896250	0.579873
H	-2.824303	-1.258917	0.861938
H	2.312848	1.363544	-0.498494
H	3.507727	-2.729923	-0.736358
H	1.249696	-3.434537	-0.005387
H	4.308958	0.785993	-1.669760
H	4.766484	-0.896654	-1.904965
H	5.520875	-1.017013	0.492378
H	5.071990	0.663920	0.712583
H	6.729895	1.337016	-1.056848
H	7.179425	-0.344985	-1.274373
H	7.527407	1.246902	1.339400
H	8.825554	0.817100	0.217138
H	7.979977	-0.447029	1.120723
C	-3.074094	4.882539	2.372973
C	-4.085784	5.653816	2.906022
C	-5.431977	5.252485	2.791172
C	-5.726244	4.041761	2.204679
H	-2.050018	5.203183	2.498654
H	-3.837508	6.571961	3.429271
H	-6.223878	5.874714	3.196260
H	-6.750627	3.682534	2.160006

CDHF-7 in MH 7 (Heterochiral)
60

C	-2.138729	2.864827	0.011279
C	-0.804945	3.361728	0.307537
C	0.243554	2.419084	0.432260

C	-0.061216	0.996501	0.471750
C	-1.392470	0.626437	0.756486
C	-2.459398	1.563005	0.449531
C	-0.537537	4.755505	0.609117
C	0.813357	5.192307	0.766733
C	1.852481	4.223299	0.782912
C	1.568855	2.892364	0.689159
C	-3.131387	3.605094	-0.737613
C	-4.488251	3.160948	-0.729626
C	-4.812217	1.946142	-0.062109
C	-3.827985	1.152504	0.451657
C	-1.558546	5.707557	0.876214
C	-1.262691	7.024451	1.153200
C	0.075960	7.469014	1.192086
C	1.092998	6.558766	1.018951
C	0.933022	-0.046815	0.336927
C	0.631334	-1.356765	0.822395
C	-0.673016	-1.629480	1.322592
C	-1.661301	-0.692923	1.231887
C	2.166990	0.129363	-0.340136
C	3.106401	-0.881262	-0.446816
C	2.828440	-2.138307	0.138817
C	1.612933	-2.372425	0.740214
C	4.424726	-0.639761	-1.140434
C	5.538936	-0.241812	-0.156628
C	6.888038	-0.003229	-0.838710
C	7.989329	0.397873	0.144676
H	2.870936	4.553337	0.967371
H	2.358361	2.175704	0.862205
H	-5.848428	1.621263	-0.039296
H	-4.093617	0.181044	0.848571
H	-2.591688	5.391340	0.878693
H	-2.069658	7.721624	1.356356
H	0.298814	8.511976	1.394495
H	2.131761	6.865213	1.105842
H	-0.885169	-2.617675	1.720374
H	-2.662794	-0.951932	1.551597
H	2.365813	1.069804	-0.839346
H	3.567576	-2.932066	0.072161
H	1.375115	-3.356294	1.135245
H	4.308326	0.151855	-1.890134
H	4.731609	-1.543228	-1.682247
H	5.643306	-1.025526	0.605420
H	5.230079	0.664043	0.381757
H	6.773271	0.777167	-1.602813
H	7.186682	-0.912974	-1.376548
H	7.728835	1.322206	0.672130
H	8.943344	0.562609	-0.365818
H	8.144887	-0.379813	0.900620
C	-5.471241	3.891905	-1.441588
C	-2.802539	4.701802	-1.577147
C	-3.771495	5.374441	-2.289342
C	-5.126010	4.987312	-2.200839
H	-6.501772	3.550256	-1.400011
H	-1.767438	5.004064	-1.666977

H	-3.487559	6.203162	-2.930458
H	-5.884574	5.533414	-2.752886

CDHF-4 in MH 8 (Homochiral)

66

C	-1.561417	1.936319	-0.212025
C	-0.121427	1.851721	-0.393890
C	-2.344656	0.754223	-0.340182
C	-1.722573	-0.474027	-0.817478
C	-0.457294	-0.370053	-1.408927
C	0.437845	0.680597	-0.942072
C	-2.393538	-1.766895	-0.855495
C	-2.085234	-2.659213	-1.902813
C	-1.036162	-2.315821	-2.846324
C	-0.175232	-1.225238	-2.552928
C	-3.265542	-2.219980	0.211867
C	-3.991189	-3.440248	0.051191
C	-3.764694	-4.234934	-1.106845
C	-2.809757	-3.883067	-2.015304
C	-3.360746	-1.566810	1.469590
C	-4.196780	-2.032449	2.461409
C	-4.981647	-3.186791	2.257094
C	-4.865336	-3.882228	1.075092
C	0.735377	2.937212	-0.042443
C	2.088569	2.861132	-0.200318
C	2.703249	1.645144	-0.609102
C	1.879927	0.520208	-0.919700
C	4.114504	1.517322	-0.641032
C	4.710583	0.304131	-0.900117
C	3.902614	-0.835907	-1.099343
C	2.528243	-0.730376	-1.102112
H	-4.318757	-5.162189	-1.221199
H	-2.592072	-4.555242	-2.835807
H	-2.754335	-0.692219	1.660133
H	-4.240291	-1.510882	3.412505
H	-5.647185	-3.538777	3.039091
H	-5.423047	-4.801090	0.916544
H	0.305302	3.844614	0.362322
H	2.720367	3.709486	0.046470
H	4.717455	2.396119	-0.430045
H	5.792317	0.214872	-0.915489
H	4.364795	-1.808255	-1.238848
H	1.931253	-1.621130	-1.240159
C	0.825407	-0.878266	-3.490238
C	1.049592	-1.604993	-4.644620
C	0.209819	-2.708497	-4.904796
C	-0.812595	-3.039319	-4.039711
H	1.425658	-0.003611	-3.295847
C	2.153291	-1.245428	-5.618586
H	0.359807	-3.292842	-5.808954
H	-1.462479	-3.867052	-4.296804
C	3.181927	-0.236654	-5.100620
H	1.707598	-0.865955	-6.549455
H	2.671820	-2.171491	-5.902994
C	4.361538	-0.032539	-6.053455

C	5.372156	0.984210	-5.518180
H	3.558381	-0.568090	-4.124641
H	2.696276	0.731867	-4.924346
H	3.990896	0.294186	-7.034315
H	4.861271	-0.995852	-6.222409
H	4.908169	1.968271	-5.388896
H	6.223980	1.102199	-6.195235
H	5.758112	0.674638	-4.540506
C	-3.750573	0.860899	-0.209298
C	-4.358534	2.063756	0.089147
C	-3.580837	3.220204	0.256580
C	-2.210434	3.152500	0.095145
H	-4.362674	-0.014383	-0.377246
H	-5.439648	2.114620	0.171654
H	-4.053686	4.170173	0.485146
H	-1.630820	4.063502	0.178862

CDHF-4 in MH 8 (Heterochiral)
66

C	-0.743051	0.990221	1.518946
C	0.339304	1.326854	0.611187
C	-1.405461	-0.259453	1.369592
C	-1.256649	-0.985455	0.113178
C	-0.130115	-0.712465	-0.679477
C	0.798132	0.335275	-0.279574
C	-2.188146	-2.026746	-0.294536
C	-1.734784	-3.009438	-1.200851
C	-0.641018	-2.678115	-2.093265
C	0.024245	-1.435259	-1.935019
C	-3.571560	-2.106283	0.138795
C	-4.278316	-3.339104	-0.006747
C	-3.645002	-4.425206	-0.676963
C	-2.452314	-4.238712	-1.312821
C	-4.306145	-0.981498	0.595973
C	-5.623688	-1.094469	0.986188
C	-6.280251	-2.342151	0.945382
C	-5.617195	-3.440279	0.444213
C	1.040713	2.567760	0.692246
C	2.222005	2.760171	0.035615
C	2.865689	1.669038	-0.616491
C	2.178748	0.421459	-0.718825
C	4.194933	1.780076	-1.094570
C	4.869927	0.677930	-1.571098
C	4.236551	-0.583499	-1.558646
C	2.925766	-0.705910	-1.148697
H	-4.165527	-5.376063	-0.745048
H	-2.032604	-5.043856	-1.903941
H	-3.827752	-0.011813	0.618134
H	-6.160737	-0.211605	1.318719
H	-7.311527	-2.425932	1.273765
H	-6.123369	-4.397195	0.351507
H	0.614673	3.379661	1.269508
H	2.725874	3.721539	0.073691
H	4.685459	2.747957	-1.040907
H	5.894347	0.769250	-1.918396
H	4.785494	-1.466917	-1.869484
H	2.459698	-1.682005	-1.137966
C	0.786355	-0.934003	-3.013420
C	1.046625	-1.679701	-4.148521
C	0.495474	-2.977341	-4.226804
C	-0.345446	-3.450754	-3.241750
H	1.188584	0.063802	-2.933939
C	1.905112	-1.156089	-5.282094
H	0.699978	-3.591782	-5.099905
H	-0.825354	-4.411790	-3.382816
C	2.621291	0.168229	-5.009143
H	1.288797	-1.059322	-6.187514
H	2.654128	-1.923448	-5.523750
C	3.587495	0.575428	-6.123387
C	4.306403	1.890413	-5.813777
H	3.176586	0.093286	-4.067049
H	1.881774	0.967199	-4.866923
H	3.043630	0.662982	-7.073501
H	4.327632	-0.223051	-6.268562
H	3.591679	2.714932	-5.714506
H	5.018168	2.157859	-6.601083
H	4.856644	1.819838	-4.868822
C	-2.144850	-0.759793	2.465141
C	-2.352991	-0.001061	3.600201
C	-1.818256	1.294889	3.683634
C	-1.011139	1.769669	2.667933
H	-2.557176	-1.757896	2.409813
H	-2.924800	-0.411268	4.426456
H	-1.993844	1.903441	4.565271
H	-0.524304	2.729490	2.791062

CDHF-5 in MH 8 (Homochiral)
72

C	-0.898133	0.513035	1.438125
C	-0.119559	1.269662	0.524687
C	-1.811355	-0.397153	0.982307
C	-1.848915	-0.764563	-0.392264
C	-0.942528	-0.163576	-1.293325
C	-0.252537	1.035653	-0.869849
C	-2.754293	-1.811136	-0.844261
C	-2.534731	-2.461572	-2.076671
C	-1.589131	-1.863112	-3.007007
C	-0.721956	-0.822937	-2.576049
C	-3.915380	-2.133292	-0.084063
C	-4.830503	-3.031566	-0.557381
C	-4.538996	-3.827113	-1.696859
C	-3.286486	-3.668829	-2.358986
C	-5.485043	-4.775434	-2.180981
C	-2.866900	-4.740018	-3.241508
C	0.397275	-0.515781	-3.387208
C	0.550722	-1.010651	-4.667958
C	-0.475708	-1.835572	-5.177811
C	-1.497705	-2.264578	-4.362822
H	1.146445	0.148731	-2.986920
C	1.742528	-0.666797	-5.536302

H	-0.436571	-2.167541	-6.211699
H	-2.244705	-2.935912	-4.762133
C	2.675778	0.412124	-4.983790
H	1.369024	-0.348577	-6.519920
H	2.317659	-1.583892	-5.729757
C	3.788738	0.806177	-5.956947
C	4.696503	1.899047	-5.388338
H	3.127313	0.065639	-4.044794
H	2.091284	1.304357	-4.730316
H	3.339733	1.153122	-6.897402
H	4.387062	-0.078334	-6.213656
H	4.116605	2.792212	-5.130042
H	5.471393	2.194039	-6.102885
H	5.195990	1.558798	-4.474409
C	-3.865205	-5.591549	-3.781321
C	-5.189649	-5.561208	-3.256699
C	-1.479862	-5.068620	-3.527581
C	-1.183149	-5.980619	-4.585068
C	-2.249246	-6.659735	-5.252557
C	-3.532171	-6.516350	-4.821087
C	-0.393831	-4.576841	-2.763465
C	0.912019	-4.876234	-3.090448
C	1.200837	-5.693818	-4.200309
C	0.165132	-6.249203	-4.920637
H	-0.792844	0.705849	2.501666
H	-2.440168	-0.921189	1.691440
H	-4.133804	-1.575475	0.818109
H	-5.778175	-3.177393	-0.047489
H	-6.455198	-4.832528	-1.696127
H	-5.930834	-6.234981	-3.676366
H	-2.006811	-7.341456	-6.062682
H	-4.334793	-7.100210	-5.262492
H	-0.592544	-3.958685	-1.899253
H	1.719845	-4.479810	-2.483554
H	2.231165	-5.910891	-4.464579
H	0.367672	-6.926212	-5.746013
C	0.239576	2.079035	-1.760147
C	1.147838	3.058481	-1.255124
C	1.447903	3.090016	0.142529
C	0.777738	2.276471	1.003109
C	-0.241662	2.257993	-3.081574
C	0.243356	3.259857	-3.897133
C	1.238771	4.142560	-3.431066
C	1.667105	4.048240	-2.124521
H	2.151751	3.831659	0.508820
H	0.916288	2.368592	2.076554
H	-1.013119	1.598459	-3.454909
H	-0.152874	3.371395	-4.901624
H	1.632728	4.915013	-4.084139
H	2.385359	4.759866	-1.726966

CDHF-5 in MH 8 (Heterochiral)

72

C	-0.876999	1.296469	0.677693
C	0.445402	1.204843	0.174025
C	-1.853100	0.434839	0.251793
C	-1.613875	-0.463346	-0.822225
C	-0.350560	-0.437248	-1.459627
C	0.746018	0.244637	-0.829085
C	-2.605637	-1.450864	-1.238458
C	-2.276221	-2.371898	-2.257935
C	-1.240005	-1.967743	-3.198775
C	-0.289631	-0.997811	-2.805939
C	-3.878628	-1.557316	-0.612989
C	-4.771231	-2.522363	-0.997900
C	-4.390447	-3.551705	-1.897870
C	-3.063052	-3.565861	-2.415420
C	1.473217	2.064401	0.675977
C	2.761998	1.939194	0.258117
C	3.145380	0.860054	-0.599453
C	2.150872	-0.033369	-1.100183
C	4.510274	0.627003	-0.894401
C	4.905978	-0.490791	-1.597172
C	3.938020	-1.433164	-1.998109
C	2.598413	-1.210721	-1.750866
C	-5.317904	-4.553410	-2.306328
C	-2.601636	-4.770346	-3.072994
H	1.194955	2.830089	1.394499
H	3.532576	2.613061	0.621252
H	5.246627	1.337220	-0.528646
H	5.956530	-0.662483	-1.809906
H	4.244423	-2.346687	-2.498614
H	1.877403	-1.953905	-2.061561
C	0.612740	-0.493649	-3.768091
C	0.653007	-0.968952	-5.066085
C	-0.293053	-1.946908	-5.440436
C	-1.225092	-2.414849	-4.539511
H	1.291382	0.290924	-3.468412
C	1.660693	-0.463548	-6.078148
H	-0.302590	-2.317036	-6.462301
H	-1.970605	-3.128113	-4.865925
C	2.849137	0.302610	-5.490571
H	1.150159	0.167994	-6.819756
H	2.036167	-1.327547	-6.643331
C	3.928769	0.629689	-6.524213
C	5.104879	1.395827	-5.914733
H	3.292740	-0.284025	-4.676419
H	2.500438	1.237956	-5.034000
H	3.489929	1.214464	-7.343799
H	4.290999	-0.303402	-6.976358
H	4.776130	2.355220	-5.500092
H	5.882230	1.601164	-6.657424
H	5.560840	0.826244	-5.097181
C	-3.575084	-5.675793	-3.564894
C	-4.942065	-5.527953	-3.184863
C	-1.204292	-5.151477	-3.184193
C	-0.843374	-6.218409	-4.061659
C	-1.867626	-6.979526	-4.705252
C	-3.179220	-6.756963	-4.413252
C	-0.175069	-4.572767	-2.400257

C	1.144813	-4.941519	-2.556934
C	1.505708	-5.917355	-3.507448
C	0.521328	-6.555495	-4.230473
H	-1.089845	2.026939	1.452736
H	-2.826248	0.467247	0.725369
H	-4.174167	-0.838368	0.140732
H	-5.775906	-2.537338	-0.585635
H	-6.337616	-4.498921	-1.936413
H	-5.663511	-6.249979	-3.556074
H	-1.575917	-7.781889	-5.376775
H	-3.955318	-7.393211	-4.828963
H	-0.431738	-3.842669	-1.645353
H	1.905662	-4.485523	-1.930554
H	2.548010	-6.190318	-3.639938
H	0.775326	-7.351691	-4.924780

CDHF-7 in MH 8 (Homochiral)
66

C	-1.181200	0.345981	0.899880
C	0.134707	0.494543	0.321442
C	-2.228086	-0.141255	0.080673
C	-1.953703	-0.552967	-1.281743
C	-0.622367	-0.558507	-1.761939
C	0.442855	-0.215684	-0.848884
C	-2.999791	-1.004907	-2.146068
C	-2.756236	-1.387092	-3.432229
C	-1.462890	-1.229896	-4.003404
C	-0.397688	-0.754242	-3.186617
C	1.155575	1.429048	0.804136
C	2.512300	1.262334	0.420316
C	2.872574	0.082068	-0.352630
C	1.838606	-0.629997	-1.011765
C	4.199790	-0.381316	-0.456654
C	4.504269	-1.533589	-1.159749
C	3.473607	-2.274565	-1.759600
C	2.166433	-1.830801	-1.681011
C	0.791569	2.610942	1.488541
C	3.456298	2.242208	0.785938
H	4.991692	0.152570	0.056314
H	5.531506	-1.879808	-1.216627
H	3.696635	-3.205266	-2.271720
H	1.373270	-2.415282	-2.129363
C	0.809984	-0.393743	-3.835045
C	1.029328	-0.612090	-5.180946
C	-0.010505	-1.187432	-5.952371
C	-1.230097	-1.465284	-5.381236
H	1.585909	0.076631	-3.254851
C	2.345519	-0.257180	-5.844063
H	0.152962	-1.377769	-7.009999
H	-2.045357	-1.854197	-5.985219
C	3.466717	0.167893	-4.893109
H	2.174358	0.535992	-6.586141
H	2.680215	-1.128177	-6.425166
C	4.811265	0.379558	-5.591561
C	5.909868	0.799886	-4.612427
H	3.587073	-0.590495	-4.110163
H	3.183637	1.094856	-4.377489
H	4.705225	1.137674	-6.379079
H	5.105396	-0.548645	-6.099755
H	5.663239	1.753313	-4.132166
H	6.875784	0.917386	-5.113604
H	6.028627	0.055850	-3.816854
C	-1.439226	0.533040	2.321284
C	-3.558889	-0.165862	0.604151
H	-4.012262	-1.073312	-1.771937
H	-3.563528	-1.759645	-4.056334
C	3.081675	3.371969	1.491468
C	1.734143	3.562349	1.832043
H	4.488850	2.127262	0.475928
H	3.823389	4.120090	1.753078
C	-3.836138	0.209895	1.883775
C	-2.786082	0.519737	2.793696
C	-0.413268	0.613136	3.298810
C	-0.701843	0.787679	4.636261
C	-2.037066	0.882053	5.077339
C	-3.059011	0.732815	4.166657
H	-4.379024	-0.465818	-0.033866
H	-4.860221	0.214416	2.245716
H	0.619422	0.517213	2.996450
H	0.110238	0.838706	5.354853
H	-2.255278	1.034735	6.129675
H	-4.095826	0.744261	4.491137
H	-0.250087	2.780669	1.727887
H	1.423273	4.465472	2.347727

CDHF-7 in MH 8 (Heterochiral)
66

C	-1.088667	-0.233990	0.765558
C	0.266463	-0.618614	0.402488
C	-2.144640	-0.791559	0.012365
C	-1.882683	-1.207495	-1.352290
C	-0.576795	-1.055934	-1.866128
C	0.519981	-1.038056	-0.911112
C	-2.935600	-1.588239	-2.239465
C	-2.732145	-1.674785	-3.587423
C	-1.494712	-1.264394	-4.158448
C	-0.421009	-0.900154	-3.295511
C	1.341568	-0.687107	1.391130
C	2.689999	-0.814558	0.957659
C	2.952536	-1.244161	-0.409016
C	1.850256	-1.535152	-1.257361
C	4.255787	-1.557712	-0.858659
C	4.465552	-2.249421	-2.035706
C	3.362393	-2.698933	-2.779929
C	2.084654	-2.347436	-2.392496
H	5.112378	-1.288938	-0.252950
H	5.476045	-2.487450	-2.353107
H	3.508920	-3.319205	-3.658458
H	1.242595	-2.694941	-2.973853
C	0.730103	-0.309788	-3.876281

C	0.886201	-0.182765	-5.241501
C	-0.156953	-0.638988	-6.088202
C	-1.319842	-1.148133	-5.560356
H	1.506672	0.049396	-3.217947
C	2.134790	0.415058	-5.858056
H	-0.038226	-0.558232	-7.165652
H	-2.133388	-1.453122	-6.212903
C	3.304004	0.637775	-4.896326
H	1.877441	1.365619	-6.347478
H	2.462701	-0.247883	-6.671110
C	4.584581	1.096031	-5.597343
C	5.741805	1.300042	-4.617116
H	3.506778	-0.290922	-4.349270
H	3.025590	1.382767	-4.139567
H	4.393209	2.028332	-6.145401
H	4.869017	0.351562	-6.353146
H	5.500982	2.074608	-3.880597
H	6.659511	1.601563	-5.131755
H	5.951783	0.377678	-4.064042
C	-1.427127	0.695543	1.822711
C	-3.467634	-0.765432	0.551200
H	-3.920413	-1.798730	-1.841541
H	-3.538827	-1.981935	-4.246876
C	-3.753426	-0.087568	1.700610
C	-2.764145	0.727166	2.322743
C	-0.515263	1.664266	2.317241
C	-0.877635	2.545720	3.312746
C	-2.172446	2.503376	3.872456
C	-3.099235	1.614591	3.374778
H	-4.263948	-1.287210	0.035158
H	-4.760391	-0.092876	2.107657
H	0.477526	1.719273	1.890345
H	-0.162000	3.283357	3.662253
H	-2.443119	3.189618	4.668809
H	-4.115403	1.602250	3.759069
C	1.091418	-0.689372	2.784096
C	2.118429	-0.641081	3.705354
C	3.450153	-0.597109	3.264244
C	3.724182	-0.707634	1.915103
H	4.263771	-0.527452	3.979504
H	4.758061	-0.757425	1.596557
H	0.070747	-0.728773	3.136288
H	1.891367	-0.639663	4.766679

CDHF-8 in MH 8 (Homochiral)
48

C	-1.310771	0.608925	0.959330
C	-0.041634	0.853621	0.312353
C	-2.408503	0.220802	0.148003
C	-2.178055	-0.164881	-1.197105
C	-0.909541	-0.208955	-1.704948
C	0.193435	0.307586	-0.968947
C	0.991913	1.692338	0.902493
C	2.329640	1.587628	0.476142
C	2.598069	0.975426	-0.818185
C	1.532364	0.356673	-1.538846
C	0.645559	2.725715	1.832898
C	3.354734	2.177696	1.321876
C	-1.525926	0.639075	2.399864
C	-3.732424	0.192305	0.688426
C	2.977886	3.173622	2.276388
C	1.596738	3.487220	2.439533
C	4.707731	1.751026	1.332148
C	3.965931	3.795396	3.074160
C	-3.959622	0.453340	2.004041
C	-2.862830	0.624160	2.905006
C	-0.472793	0.559521	3.346059
C	-0.718049	0.586212	4.703734
C	-2.035489	0.687467	5.191265
C	-3.087411	0.689286	4.300811
H	-4.554606	-0.037445	0.016798
H	-4.968847	0.458202	2.405484
H	0.545314	0.452990	2.998238
H	0.113223	0.516591	5.398322
H	-2.220670	0.725620	6.260203
H	-4.113133	0.706152	4.658647
C	5.286066	3.403528	3.006162
C	5.648054	2.345084	2.151895
H	-3.023675	-0.478149	-1.802379
H	-0.764255	-0.580318	-2.710592
H	5.001686	0.900312	0.732185
H	3.655488	4.570178	3.769685
H	6.033433	3.878675	3.633793
H	6.669490	1.977350	2.148156
C	3.841993	1.124662	-1.481720
C	1.805294	-0.197129	-2.810411
C	3.052609	-0.106081	-3.395149
C	4.072099	0.594227	-2.735828
H	4.619449	1.717007	-1.023317
H	1.021302	-0.711480	-3.350839
H	3.228356	-0.546525	-4.371507
H	5.036692	0.738072	-3.212499
H	-0.402399	2.919956	2.018512
H	1.314580	4.301291	3.100394

CDHF-8 in MH 8 (Heterochiral)
48

C	-1.175578	0.446331	0.983124
C	0.207558	0.428597	0.573860
C	-2.166232	0.208467	-0.007868
C	-1.779766	-0.263379	-1.288489
C	-0.472905	-0.579412	-1.547236
C	0.545510	-0.262312	-0.611324
C	1.265918	1.215679	1.209416
C	2.619785	0.987447	0.888440
C	2.938367	-0.171304	0.061213
C	1.925601	-0.688559	-0.793370
C	4.187485	-0.829305	0.088327
C	4.478399	-1.866713	-0.780900
C	3.516399	-2.292193	-1.707050

C 2.255152 -1.723665 -1.693612
C 0.924742 2.341677 2.022239
C 3.625181 1.934683 1.327942
H 4.919046 -0.542655 0.832163
H 5.444131 -2.359638 -0.729451
H 3.739293 -3.100306 -2.396612
H 1.490035 -2.129649 -2.344662
C -1.625522 0.599253 2.358323
C -3.548563 0.424533 0.291754
C 3.252624 2.975193 2.236913
C 1.879777 3.148378 2.563178
C 4.959165 1.962286 0.837110
C 4.228713 3.877027 2.724685
C -3.952573 0.771638 1.543498
C -3.013274 0.811785 2.620939
C -0.766493 0.430206 3.473207
C -1.227670 0.569201 4.766261
C -2.579915 0.876927 5.013457
C -3.455872 0.981599 3.954296
H -4.270436 0.303925 -0.510891
H -5.001336 0.952679 1.760228
H 0.270907 0.172759 3.306206
H -0.543459 0.428106 5.597097
H -2.934100 0.998288 6.032366
H -4.512092 1.167128 4.128646
C 5.533113 3.816793 2.288305
C 5.886521 2.869316 1.307067
H -2.547471 -0.429079 -2.038482
H -0.205535 -1.018681 -2.500878
H 5.250255 1.289471 0.043481
H 3.917051 4.639641 3.433036
H 6.272846 4.516856 2.663795
H 6.893023 2.862758 0.900054
H -0.117416 2.558946 2.204815
H 1.595359 3.982201 3.198576

CDHF-9 in MH 8 (Homochiral)
60
C -0.928335 0.446832 1.025032
C 0.403034 0.455152 0.516668
C -1.901132 -0.306252 0.429577
C -1.667052 -0.925179 -0.826510
C -0.383479 -0.824077 -1.425146
C 0.709299 -0.336663 -0.609903
C -2.724484 -1.613428 -1.499604
C -2.556877 -2.098679 -2.761007
C -1.363029 -1.807823 -3.488641
C -0.294309 -1.114881 -2.848441
C 1.448883 1.205460 1.194069
C 2.810033 0.962658 0.920242
C 3.141589 0.085203 -0.193968
C 2.110700 -0.651281 -0.851914
C 4.458473 -0.050751 -0.708604
C 4.789118 -1.034100 -1.619296
C 3.807559 -1.947395 -2.039957
C 2.493526 -1.736185 -1.678452
H 5.219326 0.650995 -0.400573
H 5.807283 -1.109214 -1.988332
H 4.068250 -2.786884 -2.676471
H 1.726716 -2.401758 -2.049842
C 0.756018 -0.627135 -3.665122
C 0.843604 -0.910004 -5.014193
C -0.172815 -1.699696 -5.606519
C -1.254446 -2.117525 -4.867030
H 1.510523 -0.005071 -3.209581
C 1.987498 -0.407912 -5.872516
H -0.108199 -1.944514 -6.663616
H -2.060648 -2.673105 -5.338314
C 3.104309 0.325362 -5.127844
H 1.584756 0.244153 -6.660883
H 2.418186 -1.268850 -6.403654
C 4.282603 0.705398 -6.027032
C 5.392647 1.423451 -5.256378
H 3.469848 -0.303418 -4.307437
H 2.702919 1.234015 -4.660194
H 3.930235 1.341678 -6.849919
H 4.687137 -0.201897 -6.495681
H 5.025539 2.357861 -4.817730
H 6.241203 1.667960 -5.902992
H 5.762619 0.800670 -4.434172
H -1.151088 0.963654 1.949741
H -2.885553 -0.392078 0.880172
H -3.667962 -1.739058 -0.976257
H -3.354475 -2.641356 -3.260255
C 1.088582 2.247853 2.103394
C 2.031987 2.981995 2.756451
C 3.406381 2.624648 2.673476
C 3.800831 1.556329 1.805750
C 5.129506 1.075715 1.958263
C 6.036474 1.693524 2.796152
C 5.668660 2.829578 3.541938
C 4.364162 3.267975 3.493141
H 0.044457 2.509706 2.218095
H 1.743276 3.814216 3.391777
H 5.429261 0.166757 1.458063
H 7.036394 1.282217 2.894577
H 6.392874 3.322531 4.182744
H 4.036383 4.097680 4.113287

CDHF-9 in MH 8 (Heterochiral)
60
C -0.745683 0.669253 1.161089
C 0.526092 0.819391 0.542573
C -1.690637 -0.168338 0.632567
C -1.496196 -0.777573 -0.633739
C -0.269217 -0.578782 -1.321246
C 0.811557 0.053982 -0.611181
C -2.531568 -1.568369 -1.224314
C -2.396196 -2.084213 -2.477407
C -1.259167 -1.753102 -3.277028

C	-0.215657	-0.954629	-2.725826
C	1.509545	1.785977	1.020087
C	2.760455	1.892979	0.380291
C	3.195910	0.760791	-0.427373
C	2.224365	-0.155701	-0.916267
C	4.565405	0.446810	-0.603305
C	4.968401	-0.702405	-1.256851
C	4.007600	-1.611750	-1.725058
C	2.665354	-1.347540	-1.535522
C	1.194146	2.697524	2.072889
C	3.572247	3.065576	0.608557
H	5.314745	1.086220	-0.156888
H	6.026595	-0.922009	-1.359177
H	4.314226	-2.540418	-2.196031
H	1.929989	-2.077784	-1.845690
C	0.785289	-0.478590	-3.605606
C	0.856953	-0.859888	-4.931473
C	-0.138138	-1.731722	-5.436537
C	-1.177517	-2.146800	-4.635314
H	1.521509	0.207336	-3.218729
C	1.969459	-0.377876	-5.841532
H	-0.089980	-2.050976	-6.474475
H	-1.965214	-2.775954	-5.040872
C	3.117859	0.351205	-5.139398
H	1.545780	0.271572	-6.621172
H	2.372622	-1.248133	-6.378230
C	4.287727	0.686876	-6.066190
C	5.415689	1.413476	-5.329670
H	3.485113	-0.264119	-4.308724
H	2.745440	1.279975	-4.687830
H	3.933068	1.303091	-6.903207
H	4.674633	-0.239364	-6.511836
H	5.065785	2.368156	-4.921055
H	6.262308	1.623050	-5.990905
H	5.782863	0.813596	-4.489307
H	-0.964155	1.189184	2.084970
H	-2.632223	-0.328756	1.149595
H	-3.431260	-1.750559	-0.643580
H	-3.177037	-2.702063	-2.911662
C	3.229800	3.951674	1.677509
C	2.044544	3.705591	2.424473
C	4.036991	5.084952	1.939412
C	5.117437	5.386406	1.140374
C	5.404623	4.571858	0.025758
C	4.652683	3.444600	-0.232613
H	0.259390	2.594729	2.608717
H	1.795460	4.373008	3.244421
H	3.768942	5.729468	2.772007
H	5.723868	6.263231	1.344528
H	6.213564	4.841921	-0.646059
H	4.860826	2.864824	-1.122268

CDHF-6 in MH 9 (Homochiral)

60

C	-0.323832	0.355954	1.688413
C	0.631255	0.989247	0.799862
C	-1.081473	-0.741215	1.220684
C	-0.652171	-1.488013	0.052189
C	0.350183	-0.919205	-0.768645
C	1.072374	0.260464	-0.332456
C	1.133490	2.339547	0.966409
C	2.377104	2.666945	0.368544
C	2.944196	1.786373	-0.590015
C	2.255799	0.680264	-1.004240
C	0.405009	3.415194	1.619872
C	3.035899	3.892043	0.704738
C	-0.521795	0.762526	3.070930
C	-2.334056	-1.008796	1.869363
C	2.446148	4.803817	1.524810
C	1.095320	4.618396	1.956371
C	-0.988792	3.370327	1.865220
C	0.413224	5.647112	2.648604
C	-1.640015	4.399055	2.512723
C	-0.928868	5.536657	2.941892
C	-2.677386	-0.408350	3.042102
C	-1.732876	0.408755	3.733845
C	0.464128	1.449487	3.817806
C	0.232713	1.840404	5.120727
C	-1.002683	1.567741	5.739643
C	-1.963015	0.851312	5.056568
H	-3.025808	-1.687673	1.389950
H	-1.558320	2.515725	1.526920
H	0.965518	6.542772	2.919349
H	-2.709292	4.330633	2.685790
H	-1.443130	6.334890	3.467980
H	-3.647658	-0.593983	3.492971
H	1.421762	1.661159	3.360981
H	1.011204	2.359490	5.670761
H	-1.185235	1.896109	6.758135
H	-2.904253	0.593160	5.533966
C	-1.203142	-2.773366	-0.325352
C	0.554296	-1.442825	-2.077411
C	-1.093922	-3.170650	-1.683575
C	-0.219632	-2.464528	-2.550951
H	3.899534	2.043172	-1.038001
H	2.665146	0.066242	-1.796582
H	4.023396	4.072820	0.289983
H	2.960478	5.719641	1.801424
H	1.256131	-0.957831	-2.743780
H	-0.137349	-2.785609	-3.585129
C	-1.833198	-4.295061	-2.167672
C	-1.768957	-3.743261	0.601285
C	-2.510928	-4.851027	0.086142
C	-2.575836	-5.064012	-1.325557
C	-1.509372	-3.726754	1.995391
C	-2.054108	-4.669572	2.842552
C	-2.886191	-5.688547	2.338521
C	-3.093665	-5.781809	0.979277
H	-1.788833	-4.517213	-3.230000
H	-3.157858	-5.900701	-1.700843

H	-0.851680	-2.970726	2.401657
H	-1.827541	-4.629574	3.903377
H	-3.326922	-6.416535	3.012436
H	-3.679974	-6.597453	0.565289

CDHF-6 in MH 9 (Heterochiral)

60

C	-1.236962	0.391839	1.244602
C	-0.056335	0.831082	0.506537
C	-2.270020	-0.240923	0.525650
C	-2.067319	-0.610160	-0.872392
C	-0.721578	-0.744976	-1.289641
C	0.299342	0.046181	-0.618899
C	0.782870	1.941751	0.870687
C	2.122698	1.950263	0.396853
C	2.527047	1.003566	-0.577129
C	1.621680	0.136069	-1.127305
C	0.326129	3.095535	1.634056
C	3.053561	2.921902	0.884726
C	-1.310706	0.440483	2.693289
C	-3.432075	-0.696969	1.225213
C	2.663605	3.895829	1.749619
C	1.286346	4.038052	2.111139
C	-1.039736	3.374870	1.875618
C	0.861782	5.139112	2.890587
C	-1.432595	4.469970	2.618341
C	-0.474707	5.349769	3.155761
C	-3.576329	-0.506534	2.566089
C	-2.521778	0.051859	3.342284
C	-0.214162	0.790242	3.522285
C	-0.337248	0.850888	4.893889
C	-1.565217	0.554650	5.519198
C	-2.631654	0.144811	4.750872
H	-4.201112	-1.219659	0.674565
H	-1.795155	2.722298	1.460964
H	1.613059	5.832235	3.258915
H	-2.489746	4.653558	2.782648
H	-0.788669	6.201294	3.751341
H	-4.473855	-0.848148	3.073724
H	0.745752	1.001380	3.074196
H	0.524531	1.122437	5.495489
H	-1.657621	0.619845	6.598852
H	-3.572265	-0.138640	5.215011
C	-3.118582	-1.015270	-1.768076
C	-0.426177	-1.582855	-2.397184
C	-2.789559	-1.916473	-2.816432
C	-1.428775	-2.214654	-3.082961
C	-4.479115	-0.501471	-1.721163
C	-5.486975	-1.119136	-2.523006
C	-5.129197	-2.165346	-3.429876
C	-3.824471	-2.507463	-3.607526
C	-4.840293	0.662313	-0.997498
C	-6.139163	1.128288	-0.982603
C	-7.147435	0.456871	-1.701206
C	-6.817327	-0.640272	-2.467750
H	3.552152	1.028615	-0.934897
H	1.925642	-0.487355	-1.958663
H	4.084222	2.857455	0.547937
H	3.378476	4.617435	2.134374
H	0.603402	-1.761236	-2.681189
H	-1.194192	-2.908740	-3.884581
H	-5.912395	-2.632230	-4.020049
H	-3.540511	-3.242375	-4.355284
H	-4.077345	1.205612	-0.456360
H	-6.379994	2.025822	-0.421517
H	-8.169878	0.820566	-1.675347
H	-7.572173	-1.140568	-3.068017

CDHF-7 in MH 9 (Homochiral)

72

C	-1.196068	0.364805	0.869783
C	0.100500	0.589523	0.262968
C	-2.229575	-0.183477	0.074041
C	-1.967986	-0.536696	-1.308139
C	-0.640865	-0.513298	-1.796143
C	0.423898	-0.156968	-0.883838
C	-3.018813	-0.953753	-2.182012
C	-2.780256	-1.296517	-3.480829
C	-1.485486	-1.139679	-4.047481
C	-0.416253	-0.687608	-3.221739
C	1.113939	1.527791	0.739976
C	2.472472	1.243002	0.500949
C	2.824398	0.052553	-0.255655
C	1.808390	-0.599578	-1.004816
C	4.136290	-0.467868	-0.289231
C	4.441306	-1.606167	-1.011945
C	3.425867	-2.282341	-1.708121
C	2.135646	-1.789291	-1.697225
C	0.758712	2.810515	1.318444
C	3.476189	2.119872	1.007823
H	4.917780	0.011813	0.287689
H	5.455946	-1.991865	-1.013672
H	3.646410	-3.203374	-2.238447
H	1.351756	-2.330172	-2.210761
C	0.797753	-0.331821	-3.861891
C	1.017474	-0.532528	-5.210056
C	-0.029370	-1.079427	-5.993472
C	-1.253194	-1.351798	-5.429543
H	1.579396	0.116115	-3.271190
C	2.340728	-0.188494	-5.864401
H	0.133885	-1.253758	-7.053872
H	-2.071684	-1.721049	-6.041443
C	3.473315	0.179670	-4.903078
H	2.187907	0.631672	-6.580751
H	2.653488	-1.048457	-6.473330
C	4.821896	0.376330	-5.598277
C	5.932639	0.742980	-4.611390
H	3.573990	-0.603513	-4.141600
H	3.215347	1.098701	-4.360827
H	4.733080	1.157907	-6.364702

H	5.091786	-0.544745	-6.132214
H	5.710679	1.688722	-4.104708
H	6.899825	0.850498	-5.112338
H	6.035868	-0.025177	-3.836791
C	-1.425253	0.535349	2.297361
C	-3.532741	-0.338498	0.640749
H	-4.033695	-1.009136	-1.811808
H	-3.592628	-1.637131	-4.116498
C	3.150865	3.251673	1.696462
C	1.791472	3.645574	1.844808
C	-0.564785	3.320329	1.337373
C	1.459088	4.880519	2.453964
H	4.519662	1.889310	0.831842
H	3.928342	3.898553	2.092533
C	-0.860258	4.535318	1.917001
C	0.154721	5.316820	2.505940
C	-3.796999	-0.004384	1.934700
C	-2.751247	0.405989	2.809240
C	-0.387476	0.751423	3.238955
C	-0.654417	0.931258	4.579994
C	-1.978064	0.894879	5.060733
C	-3.004675	0.618641	4.185683
H	-4.338982	-0.722945	0.030693
H	-1.360322	2.752737	0.876287
H	2.262505	5.487415	2.862243
H	-1.884523	4.894672	1.911214
H	-0.089327	6.266551	2.971600
H	-4.802471	-0.103352	2.333532
H	0.640511	0.759687	2.908026
H	0.167805	1.093040	5.269963
H	-2.180859	1.051893	6.115525
H	-4.027868	0.534085	4.541185

CDHF-7 in MH 9 (Heterochiral)

72

C	-1.150197	-0.283614	0.754585
C	0.184548	-0.749376	0.416591
C	-2.220773	-0.749166	-0.037048
C	-1.950955	-1.178609	-1.394570
C	-0.629502	-1.075305	-1.885785
C	0.451672	-1.107217	-0.917612
C	-2.999110	-1.534037	-2.297544
C	-2.775276	-1.635627	-3.641037
C	-1.518689	-1.257247	-4.192937
C	-0.449666	-0.917955	-3.314083
C	1.242846	-0.862872	1.410935
C	2.580431	-0.830122	0.968640
C	2.871855	-1.241120	-0.393360
C	1.790516	-1.574080	-1.256126
C	4.191351	-1.523226	-0.818550
C	4.435711	-2.215233	-1.988738
C	3.356987	-2.691320	-2.753624
C	2.063134	-2.376250	-2.388219
H	5.029136	-1.248822	-0.189449
H	5.456497	-2.436216	-2.284829
H	3.536405	-3.307132	-3.629170
H	1.238560	-2.743397	-2.983316
C	0.717758	-0.344012	-3.877693
C	0.894107	-0.214286	-5.240561
C	-0.142898	-0.653760	-6.102878
C	-1.321585	-1.143758	-5.591938
H	1.489323	0.004884	-3.208452
C	2.157729	0.370804	-5.838585
H	-0.008099	-0.572537	-7.178413
H	-2.131815	-1.432032	-6.256116
C	3.301874	0.619543	-4.853833
H	1.910997	1.308599	-6.357097
H	2.507389	-0.310599	-6.627139
C	4.595663	1.074517	-5.532352
C	5.728160	1.302018	-4.528687
H	3.499035	-0.297587	-4.285914
H	2.999567	1.375625	-4.117429
H	4.411725	1.996327	-6.100313
H	4.902374	0.319396	-6.268639
H	5.465451	2.087309	-3.811231
H	6.656098	1.600539	-5.026457
H	5.930105	0.390302	-3.955314
C	-1.439223	0.634369	1.835068
C	-3.553784	-0.626839	0.466054
H	-3.993989	-1.721698	-1.913170
H	-3.577636	-1.927817	-4.312432
C	-3.814596	0.040957	1.627013
C	-2.780165	0.747698	2.307448
C	-0.459625	1.477163	2.416301
C	-0.768848	2.313723	3.466759
C	-2.077938	2.352776	3.990258
C	-3.067031	1.591883	3.407015
H	-4.376020	-1.062426	-0.087766
H	-4.829936	0.106152	2.007462
H	0.547323	1.468511	2.020242
H	0.001902	2.949635	3.890627
H	-2.308307	3.001313	4.829741
H	-4.090740	1.645166	3.767180
C	1.001399	-1.032453	2.830482
C	2.055105	-0.752883	3.750962
C	3.349409	-0.436080	3.245105
C	3.615176	-0.542508	1.910995
C	-0.217615	-1.527581	3.356547
C	-0.417907	-1.640849	4.715430
C	0.593509	-1.264312	5.622937
C	1.813937	-0.843199	5.143057
H	4.139125	-0.182845	3.946544
H	4.628359	-0.392193	1.558542
H	-1.001447	-1.834389	2.677143
H	-1.361672	-2.027069	5.087194
H	0.419702	-1.337048	6.691999
H	2.622564	-0.598423	5.826239

CDHF-8 in MH 9 (Homochiral)

54

C	-1.284726	0.604367	0.928138
C	-0.070784	0.943975	0.221190
C	-2.357827	0.049789	0.183840
C	-2.129543	-0.394418	-1.143246
C	-0.879792	-0.333660	-1.696028
C	0.181928	0.341720	-1.035045
C	0.922610	1.862393	0.753949
C	2.260766	1.697033	0.388011
C	2.558203	1.068310	-0.888215
C	1.502865	0.459552	-1.631637
C	0.570203	3.031529	1.556838
C	3.278691	2.197780	1.309751
C	-1.456769	0.721868	2.369148
C	-3.653214	-0.084854	0.777208
C	2.932013	3.187262	2.271429
C	1.580334	3.729423	2.267238
C	-0.734869	3.570831	1.534802
C	1.241845	4.913080	2.953643
C	4.560974	1.607028	1.392472
C	3.896636	3.593435	3.214473
C	-1.044734	4.730302	2.219338
C	-0.050984	5.404099	2.944391
C	-3.865745	0.246409	2.079056
C	-2.768334	0.607862	2.922517
C	-0.377559	0.859871	3.275492
C	-0.586561	0.994670	4.632681
C	-1.892377	0.989491	5.158500
C	-2.961063	0.780120	4.313307
H	-4.465847	-0.449185	0.155340
H	-1.501001	3.076010	0.953192
H	2.013823	5.467563	3.474566
H	-2.054798	5.125499	2.178801
H	-0.287408	6.320445	3.476123
H	-4.856698	0.173917	2.517876
H	0.636620	0.838450	2.901635
H	0.265911	1.097162	5.296655
H	-2.052042	1.114258	6.224957
H	-3.971602	0.715304	4.707144
C	5.163336	3.036696	3.239124
C	5.489827	2.020405	2.331719
H	-2.952266	-0.843935	-1.691544
H	-0.716339	-0.776606	-2.669778
H	4.802503	0.773131	0.746847
H	3.630403	4.330585	3.962990
H	5.884866	3.360970	3.982402
H	6.459436	1.534699	2.379530
C	3.818383	1.218816	-1.518474
C	1.780765	-0.029639	-2.927069
C	3.035228	0.083412	-3.494303
C	4.058394	0.733020	-2.789099
H	4.596244	1.779545	-1.019889
H	0.993624	-0.496214	-3.505571
H	3.215947	-0.303530	-4.492191
H	5.032131	0.878320	-3.246292

CDHF-8 in MH 9 (Heterochiral)

54

C	-1.189873	0.448176	0.894962
C	0.160959	0.550930	0.400187
C	-2.204603	0.041405	-0.011499
C	-1.841997	-0.465002	-1.285855
C	-0.525402	-0.670531	-1.601685
C	0.506178	-0.207251	-0.743520
C	1.191639	1.427352	0.952742
C	2.539980	1.118676	0.754065
C	2.875535	-0.072144	-0.020815
C	1.897821	-0.591537	-0.917101
C	4.110390	-0.752597	0.075522
C	4.436400	-1.788983	-0.781423
C	3.526092	-2.191735	-1.768676
C	2.269674	-1.616112	-1.813946
C	0.835126	2.679297	1.617993
C	3.568221	1.999143	1.303369
H	4.805028	-0.479141	0.857976
H	5.390817	-2.295397	-0.677300
H	3.781809	-2.990476	-2.457756
H	1.540177	-2.009218	-2.511616
C	-1.567139	0.668517	2.281801
C	-3.582234	0.131384	0.367076
C	3.206439	2.979307	2.271935
C	1.803533	3.345875	2.413358
C	-0.443862	3.266835	1.491251
C	1.394129	4.447224	3.198071
C	4.923105	1.951706	0.895114
C	4.229815	3.681363	2.944248
C	-0.809327	4.371252	2.233777
C	0.104934	4.938989	3.133636
C	-3.943077	0.532330	1.615882
C	-2.949945	0.766355	2.619190
C	-0.626462	0.728046	3.337314
C	-1.018230	0.973306	4.637224
C	-2.378565	1.158862	4.949582
C	-3.326083	1.039013	3.955142
H	-4.334510	-0.124797	-0.373425
H	-1.155626	2.838216	0.800133
H	2.109765	4.948439	3.837889
H	-1.802423	4.793728	2.120056
H	-0.181999	5.789646	3.743934
H	-4.990156	0.622433	1.890334
H	0.421362	0.569738	3.121824
H	-0.270729	1.016204	5.423016
H	-2.679824	1.365369	5.971861
H	-4.383622	1.130728	4.186968
C	5.560323	3.525434	2.604136
C	5.902651	2.692529	1.529972
H	-2.627698	-0.757462	-1.976177
H	-0.270260	-1.160274	-2.533598
H	5.198817	1.344245	0.045135
H	3.972153	4.387836	3.723585
H	6.325749	4.084377	3.133280

H	6.931334	2.631151	1.188895

MH 1 (MMMMMM)

102

C	-0.484105	1.198323	-0.112367
C	0.739924	1.852744	0.302080
C	1.890121	1.076329	0.526906
C	1.954819	-0.264459	-0.017429
C	0.766170	-0.900586	-0.415793
C	-0.420369	-0.101615	-0.643860
C	3.205419	-0.965622	-0.236286
C	0.771716	-2.347560	-0.514341
C	1.986162	-3.059757	-0.661951
C	3.229358	-2.371270	-0.402074
C	2.936709	1.639628	1.358056
C	0.857297	3.292357	0.433640
C	1.909484	3.868538	1.185205
C	3.032092	3.038102	1.555637
C	-1.530706	-0.556324	-1.458691
C	-1.793719	1.798099	0.057211
C	-2.907638	1.327486	-0.678705
C	-2.802426	0.057365	-1.358947
C	-1.328709	-1.542445	-2.482037
C	-3.936496	-0.705556	-1.871784
C	-3.708181	-1.729640	-2.839466
C	-2.365808	-2.046234	-3.204453
C	-2.017573	2.785613	1.074651
C	-3.212755	3.423315	1.201733
C	-4.222630	3.247850	0.209352
C	-4.053260	2.227254	-0.774015
C	-0.438116	-3.096176	-0.323694
C	-0.477911	-4.445828	-0.491323
C	0.650823	-5.129725	-1.033238
C	1.883941	-4.424189	-1.170915
C	4.426457	-0.236012	-0.429111
C	5.624923	-0.867859	-0.554393
C	5.717240	-2.272417	-0.321758
C	4.514834	-3.030577	-0.192325
C	3.810807	0.784342	2.109685
C	4.316685	3.543166	2.030961
C	5.203076	2.659688	2.717308
C	4.851163	1.281838	2.831768
C	-0.006545	4.165507	-0.309236
C	0.026564	5.515511	-0.142950
C	0.806289	6.084757	0.907484
C	1.721393	5.250299	1.617103
H	-0.316509	-1.852075	-2.705385
H	-2.186646	-2.752295	-4.009497
H	-1.222315	2.987020	1.779851
H	-3.379215	4.128713	2.010078
H	-1.323382	-2.568149	0.004653
H	-1.389507	-5.000598	-0.291462
H	4.373494	0.840683	-0.519790
H	6.529874	-0.301567	-0.752122
H	3.599768	-0.276478	2.130406
H	5.468887	0.624182	3.435609
H	-0.664951	3.730263	-1.049058
H	-0.597569	6.162460	-0.751746
C	0.635307	7.433748	1.297047
C	2.301910	5.777518	2.797527
C	2.082816	7.083635	3.187298
C	1.271523	7.935365	2.412556
C	4.797356	4.841465	1.727971
C	6.438573	3.142461	3.208477
C	6.028602	5.276892	2.175530
C	6.845446	4.435191	2.955379
C	-4.800690	-2.457386	-3.366312
C	-5.250993	-0.576893	-1.358033
C	-6.295286	-1.332413	-1.852504
C	-6.079937	-2.257257	-2.892772
C	-5.343263	4.108603	0.145692
C	-4.944016	2.231198	-1.876245
C	-6.231662	4.038789	-0.906274
C	-6.003901	3.113247	-1.943278
H	-0.041521	8.056937	0.719265
H	2.912752	5.137461	3.418798
H	2.532755	7.450110	4.104683
H	1.120197	8.967342	2.713473
H	4.196817	5.498915	1.114844
H	7.079726	2.458215	3.757134
H	6.370810	6.273068	1.913101
H	7.803330	4.791071	3.321756
H	-4.603456	-3.208013	-4.126541
H	-5.437342	0.112470	-0.546331
H	-7.286854	-1.217317	-1.426025
H	-6.909353	-2.833154	-3.291317
H	-5.466972	4.854828	0.925522
H	-4.778369	1.543186	-2.693570
H	-7.079065	4.715503	-0.955210
H	-6.658456	3.097223	-2.809097
C	4.633375	-4.364884	0.270212
C	6.970812	-2.904591	-0.149516
C	7.051178	-4.226660	0.233160
C	5.866942	-4.948307	0.479496
C	0.545950	-6.462235	-1.496041
C	2.910157	-5.047952	-1.923390
C	1.590761	-7.064799	-2.163904
C	2.768655	-6.332467	-2.409068
H	3.738651	-4.932532	0.484785
H	7.871493	-2.312846	-0.287007
H	8.017972	-4.698097	0.379735
H	5.920134	-5.968291	0.847114
H	-0.393003	-6.988420	-1.348433
H	3.815798	-4.499035	-2.140941
H	1.494286	-8.081937	-2.530714
H	3.569918	-6.774112	-2.993252

MH 1 (MMMMMP)

102

C	-0.518092	1.042746	-0.029564

C	0.702993	1.674940	0.400492
C	1.860343	0.893428	0.569963
C	1.937063	-0.399427	-0.061157
C	0.749314	-1.019715	-0.494413
C	-0.453114	-0.225566	-0.638090
C	3.198319	-1.072810	-0.296548
C	0.779321	-2.455671	-0.701789
C	2.004295	-3.130309	-0.919900
C	3.239910	-2.456193	-0.588224
C	2.805905	1.375513	1.577642
C	0.905047	3.123349	0.457001
C	2.042580	3.645252	1.105992
C	2.785445	2.731510	1.962850
C	-1.582331	-0.638311	-1.450723
C	-1.817806	1.651198	0.170976
C	-2.949585	1.201048	-0.548078
C	-2.855299	-0.044648	-1.276475
C	-1.407896	-1.558070	-2.538943
C	-3.998544	-0.795527	-1.783816
C	-3.796682	-1.765133	-2.811466
C	-2.466027	-2.037639	-3.247747
C	-1.998164	2.640839	1.192860
C	-3.185021	3.285510	1.360233
C	-4.232902	3.109870	0.408849
C	-4.098549	2.099899	-0.591684
C	-0.411116	-3.244663	-0.557179
C	-0.428579	-4.574214	-0.847074
C	0.703135	-5.181939	-1.467682
C	1.921214	-4.442834	-1.551140
C	4.416278	-0.318263	-0.355567
C	5.625582	-0.925662	-0.500206
C	5.723590	-2.346668	-0.428331
C	4.525919	-3.124182	-0.415280
C	3.581113	0.448002	2.343057
C	3.400075	3.123753	3.210558
C	4.212477	2.183375	3.915217
C	4.297720	0.848384	3.432485
C	0.097040	3.997063	-0.337284
C	0.404183	5.318669	-0.477707
C	1.602590	5.854401	0.069366
C	2.465113	4.997044	0.818914
H	-0.401071	-1.837074	-2.819563
H	-2.309134	-2.693430	-4.098635
H	-1.173837	2.844065	1.863611
H	-3.319732	3.994655	2.171339
H	-1.299899	-2.767014	-0.167096
H	-1.325800	-5.163311	-0.683823
H	4.355050	0.761421	-0.319664
H	6.532733	-0.336480	-0.594377
H	3.569713	-0.598110	2.072984
H	4.881819	0.125573	3.994746
H	-0.754426	3.593165	-0.865764
H	-0.227619	5.962424	-1.082831
C	-4.900862	-2.481068	-3.330139
C	-5.292917	-0.708361	-1.213415
C	-6.346939	-1.453159	-1.703136
C	-6.161925	-2.323040	-2.795668
C	-5.358346	3.967361	0.399088
C	-5.030686	2.117044	-1.659845
C	-6.286833	3.906791	-0.617616
C	-6.094985	2.995635	-1.674686
H	-4.726160	-3.189334	-4.135205
H	-5.453796	-0.059895	-0.363083
H	-7.322303	-1.372654	-1.233558
H	-6.999741	-2.890341	-3.188923
H	-5.453415	4.704065	1.191854
H	-4.893914	1.443063	-2.493581
H	-7.137949	4.580525	-0.625292
H	-6.781378	2.988770	-2.515609
C	4.649184	-4.500053	-0.096038
C	6.979835	-2.983822	-0.294608
C	7.065765	-4.338837	-0.057943
C	5.883445	-5.092335	0.079081
C	0.618054	-6.466661	-2.053393
C	2.952731	-4.973680	-2.365105
C	1.666864	-6.982796	-2.784497
C	2.829671	-6.209100	-2.968495
H	3.756970	-5.095529	0.035987
H	7.876778	-2.372733	-0.342751
H	8.033787	-4.815645	0.060222
H	5.938694	-6.145650	0.335608
H	-0.308959	-7.023158	-1.947361
H	3.847031	-4.389557	-2.533883
H	1.585164	-7.962344	-3.245179
H	3.633271	-6.579567	-3.597223
C	3.752510	5.494806	1.155641
C	1.994053	7.190007	-0.196154
C	4.121179	6.788976	0.859868
C	3.225268	7.659221	0.202236
C	3.153635	4.375185	3.836423
C	4.841737	2.564170	5.126263
C	4.631405	3.810303	5.671849
C	3.754352	4.711007	5.030015
H	4.461447	4.839872	1.643619
H	1.310125	7.829088	-0.747728
H	5.114260	7.137129	1.126534
H	3.519722	8.681498	-0.013761
H	2.470973	5.073355	3.371492
H	5.473549	1.838469	5.630831
H	5.110999	4.089590	6.604800
H	3.541348	5.673600	5.484518

MH 1 (MMMMPP)

102

C	-0.369734	1.021549	0.065484
C	0.857168	1.659836	0.443608
C	2.021053	0.879277	0.591111
C	2.069044	-0.435722	0.004736
C	0.865426	-1.057160	-0.388414
C	-0.325869	-0.258283	-0.522518

C	3.315295	-1.135767	-0.238230
C	0.870278	-2.491176	-0.602391
C	2.082589	-3.190436	-0.804948
C	3.329282	-2.528243	-0.484398
C	3.003092	1.392463	1.548157
C	1.034208	3.108570	0.489891
C	2.208126	3.650319	1.050176
C	3.006658	2.762110	1.884183
C	-1.428047	-0.567049	-1.435496
C	-1.690025	1.523253	0.436245
C	-2.841607	0.942880	-0.131726
C	-2.646403	0.135057	-1.328159
C	-1.207449	-1.403073	-2.575447
C	-3.575549	0.117929	-2.436658
C	-3.351939	-0.783726	-3.522056
C	-2.153602	-1.548724	-3.546633
C	-1.834291	2.433290	1.529765
C	-3.060495	2.728600	2.050304
C	-4.227944	2.053106	1.597891
C	-4.113510	1.108051	0.532429
C	-0.346528	-3.243114	-0.500204
C	-0.391369	-4.572325	-0.790145
C	0.744860	-5.215575	-1.363846
C	1.983245	-4.507380	-1.423618
C	4.546213	-0.407854	-0.345359
C	5.742825	-1.043266	-0.476716
C	5.815150	-2.461522	-0.345540
C	4.602593	-3.213781	-0.295374
C	3.802619	0.487902	2.316044
C	3.689736	3.197192	3.082707
C	4.532974	2.277998	3.779185
C	4.578067	0.923170	3.349903
C	0.142245	3.966909	-0.225920
C	0.418950	5.291633	-0.399984
C	1.660341	5.840597	0.026104
C	2.591810	5.000666	0.710439
H	-0.256394	-1.903376	-2.685929
H	-1.963379	-2.194907	-4.398709
H	-0.948796	2.871244	1.969444
H	-3.148248	3.426911	2.877602
H	-1.236407	-2.735980	-0.151426
H	-1.310345	-5.135970	-0.660905
H	4.505026	0.673324	-0.353384
H	6.659415	-0.475520	-0.604950
H	3.764462	-0.568484	2.093274
H	5.180668	0.216287	3.912897
H	-0.753932	3.547462	-0.662002
H	-0.274936	5.927270	-0.942432
C	4.698974	-4.579772	0.071977
C	7.058955	-3.118737	-0.193775
C	7.118364	-4.465330	0.093888
C	5.921945	-5.189850	0.263427
C	0.643196	-6.506628	-1.933819
C	3.020375	-5.074963	-2.206153
C	1.697047	-7.059077	-2.629367
C	2.882236	-6.315419	-2.794950
H	3.794598	-5.151430	0.227042
H	7.968022	-2.528788	-0.270041
H	8.077169	-4.956938	0.225735
H	5.957932	-6.234139	0.557501
H	-0.300147	-7.038092	-1.844900
H	3.931646	-4.515057	-2.363787
H	1.603288	-8.043084	-3.078042
H	3.691696	-6.713413	-3.398846
C	3.898708	5.511681	0.931622
C	2.017989	7.174425	-0.291250
C	4.231193	6.802994	0.585428
C	3.275673	7.656589	-0.007278
C	3.484378	4.472099	3.675391
C	5.231084	2.699148	4.937958
C	5.059802	3.966828	5.446058
C	4.152620	4.848320	4.820182
H	4.649447	4.868085	1.370233
H	1.284746	7.801573	-0.790666
H	5.240319	7.162250	0.761705
H	3.543019	8.677062	-0.263290
H	2.779092	5.157501	3.226269
H	5.884515	1.987212	5.434536
H	5.592476	4.277610	6.339403
H	3.969064	5.828567	5.249090
C	-4.655260	1.033205	-2.560580
C	-4.270024	-0.832321	-4.600122
C	-5.517893	0.985721	-3.634014
C	-5.345593	0.024902	-4.653253
C	-5.244985	0.297482	0.248166
C	-5.476753	2.239947	2.240752
C	-6.570361	1.477516	1.897810
C	-6.439976	0.477937	0.909479
H	-4.796219	1.791745	-1.803166
H	-4.089196	-1.544119	-5.400783
H	-6.330315	1.702807	-3.699548
H	-6.038923	-0.014075	-5.487619
H	-5.160556	-0.484347	-0.494683
H	-5.545172	2.981008	3.032283
H	-7.520232	1.621669	2.403130
H	-7.284807	-0.161809	0.673985

MH 1 (MMMPPP)

102

C	-0.460297	1.169711	0.053707
C	0.765716	1.781044	0.478020
C	1.916269	0.980226	0.640581
C	1.951203	-0.325873	0.049239
C	0.744449	-0.928091	-0.366111
C	-0.425454	-0.114806	-0.529680
C	3.182879	-1.000276	-0.353424
C	0.726528	-2.388530	-0.394469
C	1.940451	-3.104414	-0.419264
C	3.145298	-2.353406	-0.746224
C	2.914262	1.466588	1.590040

C	0.967629	3.225989	0.551814
C	2.130183	3.741479	1.159632
C	2.914435	2.822747	1.974110
C	-1.515807	-0.438956	-1.446263
C	-1.783390	1.697248	0.377955
C	-2.927116	1.148499	-0.236432
C	-2.711031	0.307045	-1.405970
C	-1.302797	-1.356385	-2.522425
C	-3.598929	0.277403	-2.546600
C	-3.379262	-0.686134	-3.578455
C	-2.224786	-1.514742	-3.515097
C	-1.947266	2.610387	1.466304
C	-3.184407	2.950752	1.929095
C	-4.353250	2.318165	1.422088
C	-4.222867	1.362271	0.368218
C	-0.496514	-3.097719	-0.179063
C	-0.511171	-4.447803	0.014902
C	0.701602	-5.182604	0.127407
C	1.945121	-4.497300	-0.031892
C	4.381088	-0.247508	-0.560379
C	5.481458	-0.807217	-1.140430
C	5.437107	-2.127580	-1.667791
C	4.238680	-2.892058	-1.523800
C	3.747887	0.542569	2.294607
C	3.597936	3.211423	3.187358
C	4.458794	2.273384	3.835735
C	4.531634	0.943367	3.336548
C	0.109845	4.110688	-0.173926
C	0.399545	5.437580	-0.300404
C	1.627254	5.966591	0.185726
C	2.532413	5.100061	0.872152
H	-0.377784	-1.914634	-2.561200
H	-2.045176	-2.223056	-4.318724
H	-1.068591	3.016463	1.947785
H	-3.285134	3.652260	2.752212
H	-1.424752	-2.544675	-0.142673
H	-1.452825	-4.966736	0.169092
H	4.402226	0.794954	-0.274660
H	6.383310	-0.218403	-1.280936
H	3.733175	-0.500303	2.009988
H	5.162625	0.225394	3.852442
H	-0.771999	3.710304	-0.654414
H	-0.270509	6.092412	-0.849959
C	3.835134	5.596939	1.144838
C	1.999718	7.307828	-0.078602
C	4.183564	6.896900	0.849908
C	3.250081	7.774698	0.258068
C	3.376865	4.455308	3.837158
C	5.154353	2.651011	5.010946
C	4.964165	3.890474	5.578636
C	4.042469	4.787286	4.996920
H	4.572084	4.936629	1.581244
H	1.284468	7.953530	-0.580459
H	5.189304	7.243832	1.065804
H	3.529364	8.801378	0.042892
H	2.661456	5.150290	3.419121
H	5.821490	1.927756	5.471606
H	5.494650	4.167010	6.484442
H	3.846577	5.743684	5.471702
C	-4.628842	1.234102	-2.752350
C	-4.258112	-0.742491	-4.688389
C	-5.451307	1.176867	-3.856233
C	-5.286887	0.162019	-4.823310
C	-5.368582	0.589074	0.039847
C	-5.622765	2.555597	2.004736
C	-6.726658	1.830953	1.616117
C	-6.585896	0.818229	0.643098
H	-4.761135	2.031225	-2.033576
H	-4.083921	-1.499868	-5.447606
H	-6.225391	1.926704	-3.986700
H	-5.949230	0.116470	-5.682144
H	-5.279518	-0.204240	-0.689715
H	-5.700085	3.304709	2.787826
H	-7.693203	2.013765	2.075182
H	-7.440757	0.205766	0.373676
C	4.147519	-4.109933	-2.249604
C	6.525311	-2.655833	-2.405537
C	5.209659	-4.587844	-2.985636
C	6.424153	-3.871059	-3.044063
C	3.130429	-5.204030	0.305604
C	0.693686	-6.554631	0.480968
C	1.867380	-7.225963	0.738796
C	3.093946	-6.530335	0.676899
H	3.220379	-4.666452	-2.233959
H	7.434271	-2.065409	-2.480147
H	5.105479	-5.519177	-3.533588
H	7.260290	-4.263011	-3.614692
H	4.079391	-4.685859	0.282690
H	-0.264092	-7.059297	0.572351
H	1.850281	-8.274796	1.018126
H	4.017304	-7.040002	0.934062

MH 1 (MMPMMM)
102

C	-0.615000	1.066532	-0.250491
C	0.631404	1.786749	-0.038973
C	1.819299	1.032770	0.028916
C	1.710574	-0.405087	0.185576
C	0.590581	-1.052630	-0.371584
C	-0.525228	-0.223460	-0.800997
C	2.638073	-1.161537	0.995497
C	0.560817	-2.503126	-0.358359
C	1.724005	-3.215496	0.026751
C	2.640826	-2.575444	0.942572
C	3.089389	1.687984	-0.185469
C	0.736925	3.233779	-0.030500
C	1.991857	3.834311	0.240304
C	3.186406	3.097189	-0.104233
C	-1.592686	-0.639182	-1.679075
C	-1.939397	1.592549	-0.021261

C	-3.046459	1.129707	-0.775884
C	-2.884644	-0.065127	-1.575849
C	-1.310487	-1.543224	-2.755108
C	-3.968081	-0.816325	-2.204315
C	-3.661958	-1.734019	-3.255927
C	-2.295786	-1.996320	-3.576761
C	-2.162338	2.493245	1.071364
C	-3.384463	3.048285	1.294042
C	-4.418510	2.897707	0.321277
C	-4.229526	1.985985	-0.762597
C	-0.602132	-3.288778	-0.664063
C	-0.524073	-4.628483	-0.900918
C	0.746103	-5.276729	-0.952951
C	1.888669	-4.559068	-0.494214
C	-0.349166	4.123633	-0.333455
C	-0.286599	5.456475	-0.056595
C	0.834386	5.993447	0.644345
C	1.986147	5.169856	0.805949
H	-0.279249	-1.829866	-2.921552
H	-2.063121	-2.633844	-4.424183
H	-1.335861	2.693535	1.742287
H	-3.557510	3.682537	2.158030
H	-1.567347	-2.805973	-0.679813
H	-1.422695	-5.200644	-1.110659
H	-1.228007	3.726625	-0.818233
H	-1.115533	6.108671	-0.314254
C	4.239911	0.922630	-0.561982
C	4.418509	3.739649	-0.549857
C	5.442174	1.513315	-0.795945
C	5.567627	2.932255	-0.812406
C	3.420041	-3.310283	1.933684
C	3.503650	-0.488906	1.917217
C	4.345539	-2.598405	2.757081
C	4.368542	-1.174761	2.711355
H	4.142743	-0.148731	-0.664630
H	6.307658	0.910902	-1.056280
H	3.460891	0.588993	1.981122
H	5.033548	-0.642701	3.385456
C	3.163881	-5.151892	-0.665233
C	0.902739	-6.587744	-1.459920
C	2.154659	-7.154330	-1.578470
C	3.295517	-6.416972	-1.199850
C	3.231136	-4.685947	2.228903
C	5.146924	-3.294542	3.693758
C	4.997910	-4.649671	3.887216
C	4.000795	-5.338749	3.169629
C	6.788067	3.535463	-1.200794
C	4.523023	5.119636	-0.866047
C	6.873849	4.893790	-1.409606
C	5.714523	5.682343	-1.274915
C	0.825889	7.296765	1.193518
C	3.047001	5.653977	1.610350
C	2.999152	6.914309	2.169645
C	1.890797	7.755507	1.940445
C	-5.580178	3.703813	0.368525
C	-6.490535	3.698781	-0.666765
C	-6.243445	2.898634	-1.798933
C	-5.143288	2.065594	-1.842750
C	-5.305110	-0.791134	-1.735552
C	-4.705457	-2.437890	-3.901840
C	-6.009611	-2.329621	-3.468197
C	-6.299863	-1.526946	-2.348132
H	4.046006	-4.592003	-0.379235
H	0.017958	-7.130210	-1.781165
H	2.265662	-8.155433	-1.983394
H	4.284486	-6.843698	-1.334396
H	2.455026	-5.239541	1.723234
H	5.861005	-2.726199	4.283219
H	5.611470	-5.173863	4.613255
H	3.821759	-6.391339	3.365791
H	7.649963	2.894512	-1.364224
H	3.648740	5.749397	-0.806384
H	7.811976	5.346684	-1.714842
H	5.749869	6.741896	-1.508541
H	-0.050040	7.920545	1.038754
H	3.901443	5.013880	1.793643
H	3.818094	7.256865	2.794341
H	1.867908	8.751859	2.370851
H	-5.717666	4.361858	1.221981
H	-7.369200	4.335047	-0.628744
H	-6.914479	2.942481	-2.651068
H	-4.964122	1.477212	-2.731612
H	-5.549233	-0.199468	-0.864530
H	-4.449460	-3.101863	-4.722768
H	-6.800047	-2.888467	-3.959353
H	-7.310217	-1.492358	-1.952743

MH 1 (MMPMMP)

102

C	-0.587002	1.213740	-0.350541
C	0.665011	1.898983	-0.132471
C	1.830955	1.111506	-0.015121
C	1.676549	-0.314823	0.178557
C	0.544905	-0.945627	-0.382458
C	-0.528661	-0.101826	-0.851557
C	2.563644	-1.084633	1.023104
C	0.485333	-2.394753	-0.372378
C	1.607703	-3.132810	0.074233
C	2.510338	-2.497784	1.011070
C	3.121762	1.732729	-0.216834
C	0.801983	3.342874	-0.119488
C	2.069378	3.917834	0.139431
C	3.247507	3.140917	-0.183273
C	-1.539262	-0.417328	-1.851767
C	-1.929358	1.689699	-0.045873
C	-3.040328	1.124140	-0.705723
C	-2.749498	0.306647	-1.878245
C	-1.209391	-1.282499	-2.940362
C	-3.557699	0.279908	-3.074525
C	-3.225499	-0.635043	-4.122091

C	-2.045298	-1.422226	-4.009292
C	-2.136473	2.546426	1.079071
C	-3.392792	2.829131	1.529250
C	-4.533441	2.200463	0.955697
C	-4.356646	1.295952	-0.137662
C	-0.676867	-3.140711	-0.760572
C	-0.625959	-4.484821	-0.982537
C	0.620475	-5.176273	-0.932412
C	1.757264	-4.489279	-0.414286
C	-0.281442	4.242154	-0.394071
C	-0.191085	5.576603	-0.130254
C	0.959340	6.104461	0.527096
C	2.099216	5.262019	0.680986
H	-0.264183	-1.808951	-2.919528
H	-1.779867	-2.085640	-4.827308
H	-1.273525	2.949368	1.592849
H	-3.532168	3.483995	2.384496
H	-1.617750	-2.621030	-0.865888
H	-1.523685	-5.029418	-1.259328
H	-1.186049	3.848566	-0.833620
H	-1.019439	6.239016	-0.362958
C	4.261300	0.931974	-0.549922
C	4.492461	3.741944	-0.646339
C	5.477055	1.490521	-0.795073
C	5.628468	2.904734	-0.869510
C	3.220544	-3.232155	2.051193
C	3.431787	-0.420840	1.948529
C	4.149790	-2.535844	2.883598
C	4.240926	-1.117532	2.791183
H	4.144564	-0.140559	-0.612205
H	6.333322	0.862049	-1.022183
H	3.435208	0.659445	1.977209
H	4.909175	-0.593048	3.468022
C	3.018853	-5.129860	-0.497185
C	0.761294	-6.502271	-1.405488
C	1.996319	-7.114330	-1.435123
C	3.137479	-6.408707	-0.999841
C	2.958465	-4.588049	2.380956
C	4.885585	-3.236572	3.869542
C	4.666727	-4.576300	4.099766
C	3.664109	-5.243389	3.368571
C	6.861197	3.471151	-1.274554
C	4.622231	5.106937	-1.014884
C	6.971069	4.818353	-1.536753
C	5.825080	5.632067	-1.439341
C	0.986596	7.419012	1.049721
C	3.183976	5.742385	1.456455
C	3.170923	7.014074	1.990110
C	2.074554	7.872490	1.764901
H	3.901870	-4.595409	-0.169205
H	-0.120808	-7.019344	-1.772675
H	2.096304	-8.126583	-1.814311
H	4.117089	-6.871587	-1.065885
H	2.175760	-5.121764	1.863665
H	5.605107	-2.682483	4.465905
H	5.229784	-5.103609	4.863479
H	3.430454	-6.279884	3.591157
H	7.712902	2.809833	-1.407343
H	3.758147	5.753023	-0.983337
H	7.918258	5.242649	-1.854798
H	5.880597	6.680756	-1.714353
H	0.118981	8.055605	0.900419
H	4.028970	5.089888	1.638503
H	4.008074	7.352943	2.592273
H	2.079360	8.877631	2.174902
C	-5.480837	0.526444	-0.540408
C	-5.824345	2.392148	1.507408
C	-6.905302	1.673951	1.048580
C	-6.720120	0.712293	0.032140
C	-4.614243	1.198193	-3.316634
C	-4.023643	-0.683953	-5.291759
C	-5.080934	0.180221	-5.465309
C	-5.357429	1.147881	-4.475659
H	-5.356894	-0.229395	-1.304018
H	-5.937668	3.101775	2.322284
H	-7.888341	1.822557	1.484327
H	-7.558021	0.104696	-0.295384
H	-4.830460	1.960208	-2.580205
H	-3.765128	-1.403385	-6.063785
H	-5.680596	0.139808	-6.369244
H	-6.155421	1.867137	-4.631879

MH 1 (MMPMPM)

102

C	-0.684746	1.107548	-0.286285
C	0.553424	1.806065	0.025791
C	1.734143	1.036414	0.102631
C	1.614278	-0.404090	0.233906
C	0.484991	-1.033905	-0.319251
C	-0.576541	-0.182805	-0.832140
C	2.638652	-1.203374	0.860410
C	0.296807	-2.461175	-0.087472
C	1.367654	-3.247049	0.416995
C	2.638020	-2.602023	0.668671
C	3.006821	1.693112	-0.106290
C	0.679900	3.249796	0.116050
C	1.932828	3.810519	0.471573
C	3.125291	3.089812	0.085553
C	-1.581076	-0.602878	-1.775354
C	-2.019734	1.647786	-0.165731
C	-3.058053	1.214245	-1.027714
C	-2.859593	0.007154	-1.802001
C	-1.240798	-1.588832	-2.757379
C	-3.908554	-0.732750	-2.499088
C	-3.541659	-1.714469	-3.471455
C	-2.165646	-2.056042	-3.638453
C	-2.339515	2.525884	0.921384
C	-3.567402	3.102848	1.030681
C	-4.495892	2.998427	-0.048798
C	-4.215642	2.098363	-1.122068

C	-0.985227	-3.077821	-0.252076
C	-1.181226	-4.407457	-0.039451
C	-0.156996	-5.215687	0.524291
C	1.093850	-4.612517	0.855548
C	-0.372236	4.182707	-0.176132
C	-0.301891	5.490552	0.202061
C	0.792866	5.952011	0.992546
C	1.925011	5.098296	1.135095
H	-0.213822	-1.932131	-2.798645
H	-1.887240	-2.760398	-4.416434
H	-1.583138	2.693570	1.678390
H	-3.816689	3.720406	1.888075
H	-1.831117	-2.475294	-0.538124
H	-2.160468	-4.846240	-0.207695
H	-1.230630	3.841511	-0.733564
H	-1.106285	6.176056	-0.046670
C	4.122721	0.954574	-0.615348
C	4.363058	3.752509	-0.313697
C	5.327103	1.546293	-0.834420
C	5.487479	2.953649	-0.689151
C	3.918761	-3.278129	0.760680
C	3.674311	-0.635391	1.674513
C	4.986823	-2.654528	1.471670
C	4.776094	-1.354978	2.025627
H	3.995610	-0.093864	-0.843534
H	6.165478	0.961316	-1.201192
H	3.563816	0.388098	2.008551
H	5.542172	-0.919040	2.659577
C	6.715390	3.567934	-1.034812
C	4.498148	5.156214	-0.479649
C	6.833001	4.938402	-1.092444
C	5.696440	5.733091	-0.846994
C	0.782534	7.208902	1.640659
C	2.963996	5.501119	2.009540
C	2.913086	6.715277	2.662776
C	1.824957	7.589385	2.460110
C	-5.636603	3.832943	-0.111376
C	-6.432191	3.864092	-1.236986
C	-6.088395	3.070794	-2.348861
C	-5.010028	2.210756	-2.289834
C	-5.283502	-0.638386	-2.166763
C	-4.545881	-2.408653	-4.186718
C	-5.880457	-2.229292	-3.892784
C	-6.243656	-1.364162	-2.842812
H	7.556786	2.928627	-1.287330
H	3.643148	5.797678	-0.335174
H	7.776835	5.400499	-1.364528
H	5.753992	6.811095	-0.961629
H	-0.076719	7.859073	1.501860
H	3.803186	4.834425	2.168707
H	3.713520	6.996214	3.339980
H	1.800026	8.549783	2.965597
H	-5.848414	4.482895	0.733033
H	-7.294210	4.522300	-1.281881
H	-6.666807	3.141000	-3.264734
H	-4.753917	1.623869	-3.161244
H	-5.588321	-0.001695	-1.348923
H	-4.238363	-3.122067	-4.946153
H	-6.642005	-2.780173	-4.435793
H	-7.285138	-1.273127	-2.550576
C	1.946367	-5.356269	1.714825
C	-0.421302	-6.560268	0.882537
C	0.478930	-7.283736	1.631032
C	1.655066	-6.653089	2.083278
C	4.189966	-4.488151	0.075375
C	5.429474	-5.088933	0.151488
C	6.455585	-4.512842	0.928421
C	6.234944	-3.312970	1.571543
H	2.838146	-4.897648	2.112558
H	-1.371494	-6.994781	0.584773
H	0.265999	-8.311038	1.909779
H	2.334730	-7.183485	2.742994
H	3.408056	-4.938050	-0.524025
H	5.615742	-6.008263	-0.394748
H	7.422885	-5.000661	0.997711
H	7.029030	-2.836882	2.139941

MH 1 (MMPPMP)

102

C	-0.627568	1.482291	-0.141286
C	0.693460	2.063244	-0.077343
C	1.781657	1.186494	0.109320
C	1.502367	-0.201810	0.419646
C	0.303534	-0.771666	-0.066603
C	-0.725431	0.131361	-0.527322
C	2.315341	-0.981608	1.330733
C	0.143109	-2.211328	0.026616
C	1.232531	-3.000577	0.469060
C	2.183150	-2.389094	1.374071
C	3.126445	1.705482	0.046290
C	0.953209	3.444728	-0.455762
C	2.275952	3.957614	-0.415620
C	3.336370	3.101450	0.072259
C	-1.845000	-0.160860	-1.413329
C	-1.882281	2.127531	0.201997
C	-3.098350	1.598521	-0.277333
C	-2.997825	0.652358	-1.382355
C	-1.681094	-1.114786	-2.464617
C	-3.928707	0.594247	-2.486907
C	-3.766721	-0.416955	-3.485073
C	-2.629340	-1.270431	-3.432415
C	-1.897909	3.162234	1.187937
C	-3.072818	3.621128	1.707206
C	-4.312748	3.016010	1.353710
C	-4.326594	1.961835	0.387586
C	-1.076584	-2.902791	-0.276958
C	-1.112399	-4.255246	-0.448480
C	0.093961	-5.015843	-0.424871
C	1.286288	-4.378561	0.027607
C	-0.092714	4.275089	-0.971053

C	0.130687	5.561774	-1.354575
C	1.451929	6.082406	-1.423569
C	2.545613	5.245425	-1.046821
H	-0.772018	-1.700670	-2.498541
H	-2.489148	-2.003096	-4.221894
H	-0.953126	3.561793	1.535613
H	-3.070824	4.408877	2.454952
H	-1.990871	-2.334406	-0.359322
H	-2.051563	-4.755762	-0.664163
H	-1.088257	3.872574	-1.066571
H	-0.692420	6.176650	-1.707074
C	4.275176	0.851718	-0.050953
C	4.614006	3.565217	0.582318
C	5.534976	1.330332	0.145136
C	5.727955	2.673897	0.588958
C	2.844541	-3.124989	2.445558
C	3.164236	-0.325625	2.278841
C	3.778783	-2.441311	3.283893
C	3.913138	-1.028627	3.170265
H	4.121208	-0.192949	-0.288145
H	6.396308	0.676815	0.045697
H	3.194600	0.754187	2.294259
H	4.565724	-0.509271	3.865989
C	3.840626	5.679574	-1.437167
C	1.690028	7.361920	-1.982418
C	4.048886	6.914435	-2.014897
C	2.969814	7.788332	-2.255471
C	4.776338	4.838221	1.183165
C	6.977957	3.123481	1.075601
C	7.115667	4.385841	1.613283
C	5.994910	5.237911	1.691494
C	-4.944170	1.564697	-2.699780
C	-4.685492	-0.498531	-4.560600
C	-5.808060	1.478695	-3.769844
C	-5.700239	0.421235	-4.697980
C	-5.542123	1.250032	0.206612
C	-5.516859	3.390864	1.999026
C	-6.691689	2.715240	1.755978
C	-6.693025	1.617113	0.869209
H	4.687541	5.024780	-1.301475
H	0.833830	7.984362	-2.226959
H	5.055072	7.205015	-2.300565
H	3.144206	8.766406	-2.692867
H	3.921670	5.499810	1.250729
H	7.822081	2.440142	1.047151
H	8.075531	4.714289	1.999547
H	6.088169	6.212955	2.159301
H	-5.032946	2.396557	-2.014939
H	-4.554014	-1.290436	-5.292755
H	-6.570591	2.239639	-3.904296
H	-6.393890	0.354201	-5.530251
H	-5.558139	0.395314	-0.456602
H	-5.487757	4.209424	2.712829
H	-7.606447	3.005318	2.263311
H	-7.605237	1.049350	0.714320
C	0.143329	-6.362953	-0.854418
C	1.340620	-7.045159	-0.903312
C	2.534660	-6.393273	-0.529815
C	2.506098	-5.093411	-0.069734
C	2.531799	-4.463752	2.801490
C	4.471094	-3.143239	4.300224
C	4.206082	-4.470115	4.553907
C	3.196993	-5.119914	3.816360
H	-0.778746	-6.841602	-1.172182
H	1.370008	-8.073714	-1.249252
H	3.483093	-6.915172	-0.609401
H	3.427426	-4.599468	0.214476
H	1.741979	-4.984856	2.283185
H	5.194965	-2.598408	4.899887
H	4.736911	-4.998436	5.339703
H	2.925233	-6.143424	4.055330

MH 1 (MMPPPM)

102

C	-0.486074	1.147871	-0.194946
C	0.811827	1.778827	-0.008873
C	1.939431	0.938213	0.075537
C	1.732470	-0.497233	0.207121
C	0.549109	-1.059944	-0.310810
C	-0.500481	-0.146672	-0.736965
C	2.706770	-1.362510	0.826183
C	0.305803	-2.483770	-0.107081
C	1.365332	-3.339798	0.304176
C	2.658540	-2.750361	0.566194
C	3.255809	1.528030	0.053820
C	1.020009	3.206951	-0.221388
C	2.306492	3.780791	-0.020966
C	3.397915	2.904509	0.338660
C	-1.612614	-0.497992	-1.584765
C	-1.759128	1.786103	0.030819
C	-2.907704	1.400687	-0.703495
C	-2.854184	0.175295	-1.474233
C	-1.427390	-1.481468	-2.610151
C	-4.006461	-0.512253	-2.049623
C	-3.797095	-1.501924	-3.059306
C	-2.464483	-1.896975	-3.386152
C	-1.877875	2.761056	1.074060
C	-3.038539	3.440846	1.276929
C	-4.097944	3.334304	0.325564
C	-4.008504	2.359646	-0.715960
C	-1.012344	-3.032973	-0.211202
C	-1.256171	-4.363927	-0.072727
C	-0.235447	-5.251603	0.362370
C	1.055520	-4.722250	0.666807
C	-0.025504	4.040586	-0.733207
C	0.130453	5.382098	-0.894773
C	1.402633	5.993896	-0.732305
C	2.525899	5.174131	-0.407119
H	-0.427739	-1.869339	-2.767175
H	-2.305846	-2.601769	-4.196660

H	-1.020224	2.925096	1.715874
H	-3.138225	4.138080	2.103220
H	-1.847721	-2.377682	-0.389664
H	-2.264610	-4.747567	-0.197502
H	-0.965456	3.599031	-1.017144
H	-0.699239	5.986891	-1.249076
C	4.444320	0.793411	-0.274948
C	4.659780	3.316349	0.926359
C	5.684222	1.298929	-0.025260
C	5.819428	2.513049	0.715834
C	3.919766	-3.465696	0.636826
C	3.718972	-0.892287	1.728744
C	4.979814	-2.929002	1.425953
C	4.777938	-1.674147	2.078747
H	4.336421	-0.177296	-0.741666
H	6.574023	0.742293	-0.302400
H	3.618270	0.103555	2.140932
H	5.522325	-1.313832	2.781925
C	4.178591	-4.624103	-0.135058
C	6.207356	-3.626795	1.506481
C	5.401181	-5.261437	-0.075481
C	6.417522	-4.776101	0.772650
C	1.911601	-5.559458	1.431403
C	-0.539170	-6.607653	0.635627
C	0.364518	-7.419835	1.281800
C	1.584291	-6.868227	1.719935
C	3.806010	5.754394	-0.614559
C	1.578260	7.365319	-1.039163
C	3.958483	7.084162	-0.948785
C	2.836092	7.917481	-1.121686
C	4.764220	4.430612	1.793659
C	7.053623	2.914856	1.277609
C	7.133174	4.028901	2.087643
C	5.970671	4.775808	2.367913
H	3.400086	-5.005137	-0.785185
H	6.995828	-3.220498	2.133841
H	5.580937	-6.139066	-0.688384
H	7.370527	-5.293011	0.828008
H	2.836841	-5.167927	1.822106
H	-1.521252	-6.980262	0.358121
H	0.122456	-8.456239	1.495488
H	2.271262	-7.469634	2.307209
H	4.691528	5.145111	-0.532975
H	0.694936	7.962297	-1.248181
H	4.957411	7.482766	-1.096328
H	2.964238	8.967556	-1.365222
H	3.875531	5.011710	2.009243
H	7.934136	2.308181	1.085278
H	8.082382	4.317055	2.528611
H	6.022456	5.625201	3.041740
C	-5.181129	4.244311	0.347162
C	-4.927537	2.473743	-1.789061
C	-6.103929	4.274687	-0.676386
C	-5.947040	3.404275	-1.772342
C	-5.328069	-0.355757	-1.561695
C	-4.907108	-2.147740	-3.653163
C	-6.189331	-1.911868	-3.205507
C	-6.391063	-1.035381	-2.121797
H	-5.245157	4.950956	1.169860
H	-4.815608	1.832100	-2.651620
H	-6.920714	4.989416	-0.658813
H	-6.623981	3.469989	-2.618382
H	-5.506338	0.294410	-0.716866
H	-4.722022	-2.869389	-4.443889
H	-7.032087	-2.426641	-3.656124
H	-7.387265	-0.898969	-1.712743

MH 1 (MMPPPP)

102

C	-0.594237	1.268816	-0.191932
C	0.715036	1.864866	-0.042509
C	1.821608	0.992182	0.031962
C	1.575905	-0.431725	0.204056
C	0.366571	-0.972535	-0.282381
C	-0.659234	-0.041876	-0.698801
C	2.532414	-1.312239	0.830021
C	0.093692	-2.389500	-0.084604
C	1.121229	-3.260675	0.368037
C	2.431888	-2.706028	0.621766
C	3.152541	1.544878	-0.040033
C	0.947328	3.287675	-0.250057
C	2.258613	3.823626	-0.136300
C	3.343449	2.925681	0.190216
C	-1.747632	-0.291173	-1.627681
C	-1.873748	1.857434	0.162958
C	-3.060048	1.378932	-0.431783
C	-2.899483	0.522756	-1.600038
C	-1.565023	-1.228579	-2.691450
C	-3.778634	0.526398	-2.746874
C	-3.586814	-0.446187	-3.777377
C	-2.475377	-1.333759	-3.700992
C	-1.946856	2.778822	1.253534
C	-3.148978	3.186829	1.750923
C	-4.367141	2.633500	1.262217
C	-4.326204	1.682808	0.194887
C	-1.227944	-2.913937	-0.248918
C	-1.506526	-4.235936	-0.087766
C	-0.529457	-5.128562	0.430916
C	0.762582	-4.620668	0.765116
C	-0.114702	4.153803	-0.663224
C	0.071202	5.489043	-0.847211
C	1.374803	6.054691	-0.813740
C	2.492207	5.201421	-0.564053
H	-0.674209	-1.844627	-2.691671
H	-2.325202	-2.052083	-4.501702
H	-1.023414	3.134581	1.693723
H	-3.189808	3.889245	2.578343
H	-2.034043	-2.246133	-0.503580
H	-2.513024	-4.606000	-0.260526
H	-1.094230	3.742072	-0.840977

H	-0.766759	6.123465	-1.121369
C	4.307279	0.762434	-0.379394
C	4.640701	3.324897	0.706661
C	5.569451	1.239777	-0.195870
C	5.768250	2.480191	0.484351
C	3.664555	-3.467797	0.717967
C	3.573258	-0.844429	1.701018
C	4.751300	-2.940363	1.476341
C	4.605835	-1.650593	2.073141
H	4.154870	-0.221794	-0.803121
H	6.431784	0.645743	-0.482234
H	3.514560	0.170068	2.073631
H	5.370890	-1.289760	2.753578
C	3.872923	-4.668353	-0.003572
C	5.950562	-3.682613	1.582088
C	5.069089	-5.351515	0.078987
C	6.109623	-4.871236	0.900116
C	1.570985	-5.448604	1.589154
C	-0.877876	-6.465114	0.744114
C	-0.019798	-7.272708	1.455133
C	1.198517	-6.735962	1.915935
C	3.771784	5.729873	-0.882567
C	1.573243	7.412592	-1.163871
C	3.942732	7.045957	-1.258710
C	2.839064	7.915577	-1.360736
C	4.813518	4.470781	1.520574
C	7.036130	2.868869	0.976040
C	7.181450	4.013724	1.731936
C	6.052633	4.804831	2.027871
C	-4.767988	1.522386	-2.960666
C	-4.454162	-0.462985	-4.897138
C	-5.580512	1.500004	-4.073519
C	-5.444404	0.482691	-5.041795
C	-5.530807	1.001559	-0.123469
C	-5.605584	2.952181	1.871435
C	-6.766513	2.315154	1.494296
C	-6.717899	1.310633	0.504561
H	3.077717	-5.045417	-0.635193
H	6.758792	-3.280355	2.186486
H	5.209431	-6.261529	-0.495814
H	7.041322	-5.423438	0.974121
H	2.493267	-5.064077	1.994790
H	-1.857893	-6.825118	0.443644
H	-0.296871	-8.293371	1.699751
H	1.847710	-7.331746	2.550010
H	4.638549	5.088832	-0.854711
H	0.697882	8.038542	-1.313030
H	4.939538	7.405638	-1.494232
H	2.983665	8.955104	-1.637925
H	3.951697	5.085362	1.750888
H	7.890193	2.228470	0.774022
H	8.156301	4.292655	2.119662
H	6.156103	5.679804	2.661854
H	-4.876162	2.322033	-2.240446
H	-4.303257	-1.225610	-5.656119
H	-6.323914	2.279309	-4.209721
H	-6.097762	0.465377	-5.908470
H	-5.511971	0.214874	-0.865510
H	-5.615219	3.694370	2.664837
H	-7.708598	2.561181	1.974139
H	-7.620273	0.767431	0.241563

MH 2 (MMMMMM)

138

C	-0.448713	1.194060	-0.184035
C	0.768543	1.846969	0.241886
C	1.920246	1.072397	0.472996
C	1.986436	-0.270913	-0.056622
C	0.800801	-0.907489	-0.467153
C	-0.383517	-0.112708	-0.701617
C	3.243734	-0.977819	-0.246620
C	0.805212	-2.358293	-0.575003
C	2.023904	-3.062622	-0.730227
C	3.260993	-2.387153	-0.384633
C	2.974260	1.642456	1.298024
C	0.875747	3.291070	0.382041
C	1.913861	3.861475	1.158138
C	3.072398	3.044786	1.469183
C	-1.496052	-0.584469	-1.511713
C	-1.759644	1.808429	-0.040121
C	-2.858940	1.340143	-0.800245
C	-2.770475	0.023487	-1.404054
C	-1.302779	-1.557201	-2.543134
C	-3.905516	-0.721691	-1.912557
C	-3.682367	-1.632102	-2.972645
C	-2.345959	-1.982472	-3.314947
C	-2.014212	2.784465	0.974757
C	-3.251586	3.343553	1.120056
C	-4.256239	3.130836	0.134498
C	-4.005801	2.219231	-0.918467
C	-0.388162	-3.120771	-0.370386
C	-0.375791	-4.483865	-0.451498
C	0.770412	-5.163235	-0.952106
C	1.944659	-4.422692	-1.226368
C	4.468491	-0.268707	-0.457384
C	5.643217	-0.935611	-0.657419
C	5.715966	-2.341425	-0.447044
C	4.527945	-3.058706	-0.170199
C	3.835428	0.810058	2.080943
C	4.336399	3.567220	1.950300
C	5.131911	2.732608	2.770694
C	4.809439	1.349935	2.871279
C	0.044501	4.174011	-0.377467
C	0.162180	5.529253	-0.257980
C	0.959616	6.092479	0.777813
C	1.741846	5.235486	1.587812
H	-0.299261	-1.899403	-2.756644
H	-2.172325	-2.641738	-4.159926
H	-1.229106	3.021450	1.679872
H	-3.462064	4.004656	1.955168

H	-1.292050	-2.606111	-0.073930
H	-1.260270	-5.058502	-0.194085
H	4.441006	0.809888	-0.532239
H	6.546588	-0.392834	-0.918347
H	3.656120	-0.256368	2.098552
H	5.388734	0.719342	3.538770
H	-0.632143	3.753674	-1.109040
H	-0.399177	6.190078	-0.911400
C	0.997884	7.505102	0.992946
C	2.299027	5.778748	2.809499
C	2.359172	7.194147	2.973706
C	1.731624	8.040958	2.008621
C	4.848049	4.881815	1.621601
C	6.226894	3.289166	3.501820
C	5.914038	5.424093	2.398451
C	6.558140	4.605119	3.376568
C	-4.792852	-2.166385	-3.696953
C	-5.249827	-0.611668	-1.384561
C	-6.339184	-1.118880	-2.152652
C	-6.073337	-1.860234	-3.345139
C	-5.515207	3.803745	0.208376
C	-4.896754	2.240426	-2.060338
C	-6.462653	3.628204	-0.755277
C	-6.162926	2.885406	-1.938768
H	0.442295	8.143273	0.311767
C	2.728501	4.969291	3.888490
C	2.985450	7.735871	4.121191
H	1.793494	9.117111	2.142100
C	4.386279	5.635328	0.515789
H	6.776223	2.639497	4.177183
C	6.357890	6.743841	2.146110
H	7.362470	5.034582	3.966781
H	-4.588824	-2.807572	-4.549711
C	-5.534489	-0.083172	-0.102556
C	-7.663431	-0.933388	-1.689605
H	-6.913217	-2.232593	-3.924535
H	-5.705613	4.443770	1.065148
C	-4.544642	1.708428	-3.324029
H	-7.434407	4.106432	-0.673192
C	-7.078103	2.831450	-3.016712
C	4.653988	-4.410253	0.335314
C	6.962135	-3.036125	-0.534834
C	7.037477	-4.375705	-0.296186
C	5.900333	-5.088958	0.194147
C	0.757485	-6.577774	-1.157423
C	2.982442	-5.062017	-2.008873
C	1.842035	-7.222398	-1.672541
C	2.961999	-6.480076	-2.159259
C	3.614651	-5.074437	1.029980
H	7.845827	-2.471280	-0.817647
H	7.977712	-4.908118	-0.406820
C	6.006892	-6.440467	0.599672
H	-0.133652	-7.129667	-0.872404
C	3.987252	-4.338882	-2.695422
H	1.844618	-8.302600	-1.786127
C	4.021417	-7.123000	-2.842373
C	-7.912343	-0.333416	-0.473966
C	-6.832449	0.062140	0.340374
C	-5.443208	1.692330	-4.370144
C	-6.736922	2.226668	-4.207310
C	5.035106	-6.393897	-3.425937
C	4.994613	-4.986066	-3.379823
C	4.945661	-7.088443	1.194333
C	3.750939	-6.383495	1.442130
C	3.310368	5.520957	5.010597
C	3.474481	6.916581	5.115838
C	4.854235	6.909453	0.271548
C	5.824562	7.486610	1.114824
H	2.590592	3.898042	3.833059
H	3.052150	8.816333	4.213438
H	3.657243	5.199105	-0.153469
H	7.147758	7.152833	2.769966
H	-4.716607	0.205503	0.543501
H	-8.482605	-1.296362	-2.304063
H	-3.548725	1.313729	-3.472435
H	-8.050967	3.299272	-2.894091
H	2.699076	-4.541186	1.246905
H	6.954190	-6.951947	0.453480
H	3.958933	-3.257765	-2.687620
H	4.006778	-8.206707	-2.918032
H	-8.932521	-0.198049	-0.128481
H	-7.019659	0.479569	1.324470
H	-5.147388	1.269066	-5.324651
H	-7.446821	2.192982	-5.027894
H	5.841657	-6.898217	-3.948992
H	5.756591	-4.404585	-3.888754
H	5.039882	-8.124955	1.502899
H	2.932885	-6.869208	1.964257
H	3.638471	4.873867	5.817673
H	3.951459	7.343801	5.992437
H	4.474178	7.466467	-0.578763
H	6.174330	8.497485	0.929783

MH 2 (MMMMMP)

138

C	-0.505489	1.063041	0.013302
C	0.714105	1.697285	0.435854
C	1.866078	0.909768	0.633026
C	1.940746	-0.389596	0.021289
C	0.753733	-1.011698	-0.415608
C	-0.446370	-0.220148	-0.567269
C	3.206205	-1.069650	-0.193753
C	0.782301	-2.452679	-0.620275
C	2.010903	-3.121373	-0.844598
C	3.241681	-2.461369	-0.445913
C	2.806654	1.401784	1.640832
C	0.922477	3.145730	0.467543
C	2.077268	3.664681	1.098717
C	2.764402	2.764206	2.019394
C	-1.584869	-0.654526	-1.363603

C	-1.804196	1.687357	0.197704
C	-2.929141	1.238146	-0.533385
C	-2.857757	-0.060674	-1.179380
C	-1.429502	-1.573007	-2.449544
C	-4.008442	-0.793606	-1.668408
C	-3.825502	-1.660442	-2.772059
C	-2.502839	-1.977509	-3.190874
C	-2.006035	2.664039	1.221650
C	-3.233918	3.227337	1.422935
C	-4.283217	3.016386	0.484931
C	-4.078128	2.119664	-0.591179
C	-0.390823	-3.254055	-0.451369
C	-0.356405	-4.605489	-0.645671
C	0.790850	-5.219212	-1.222327
C	1.948923	-4.437626	-1.447838
C	4.423913	-0.328876	-0.301192
C	5.611315	-0.960161	-0.540117
C	5.696287	-2.379199	-0.473034
C	4.514511	-3.133775	-0.277519
C	3.625558	0.510542	2.392074
C	3.387344	3.190942	3.241828
C	4.341064	2.325833	3.837210
C	4.425047	0.979307	3.398619
C	0.101613	4.031925	-0.287478
C	0.372227	5.372652	-0.323210
C	1.561961	5.897617	0.243837
C	2.502244	5.010210	0.827673
H	-0.433287	-1.891329	-2.724963
H	-2.361075	-2.595658	-4.072096
H	-1.185094	2.898795	1.886074
H	-3.406147	3.888227	2.267069
H	-1.296978	-2.783493	-0.094892
H	-1.225142	-5.216017	-0.419156
H	4.380986	0.751528	-0.262678
H	6.514120	-0.385983	-0.725222
H	3.621861	-0.544787	2.160107
H	5.097967	0.303466	3.918380
H	-0.747844	3.643472	-0.830728
H	-0.299475	6.049576	-0.843139
C	-4.962663	-2.178693	-3.465746
C	-5.330059	-0.709659	-1.081511
C	-6.447854	-1.199655	-1.819385
C	-6.228667	-1.897967	-3.046838
C	-5.540715	3.682174	0.625556
C	-5.016153	2.161848	-1.694708
C	-6.529672	3.516952	-0.297081
C	-6.278482	2.797861	-1.506521
H	-4.791665	-2.786061	-4.349948
C	-5.564009	-0.221195	0.226344
C	-7.752487	-1.038454	-1.295662
H	-7.090322	-2.257681	-3.601722
H	-5.695047	4.307884	1.499988
C	-4.713505	1.662313	-2.984006
H	-7.498990	3.988498	-0.163691
C	-7.237814	2.762445	-2.546046
C	4.655625	-4.523676	0.106769
C	6.951443	-3.048760	-0.616729
C	7.041168	-4.403837	-0.505620
C	5.910276	-5.172362	-0.089182
C	0.798158	-6.611412	-1.545926
C	2.991540	-4.993136	-2.285941
C	1.886955	-7.192154	-2.124152
C	2.991316	-6.393769	-2.554631
C	3.623673	-5.257655	0.739004
H	7.830151	-2.449710	-0.838041
H	7.988171	-4.912885	-0.659808
C	6.031811	-6.553279	0.194589
H	-0.080492	-7.200432	-1.298985
C	3.983046	-4.200009	-2.911835
H	1.905173	-8.258740	-2.328593
C	4.055912	-6.960812	-3.294493
C	3.826797	5.530205	1.128819
C	1.814379	7.305590	0.233080
C	4.017557	6.943103	1.176266
C	2.964145	7.814191	0.753791
C	3.119230	4.459089	3.901744
C	5.208057	2.812369	4.865719
C	5.116683	4.095813	5.308700
C	4.047264	4.940181	4.872411
C	4.962804	4.709999	1.324356
H	1.048210	7.960956	-0.171182
C	5.269551	7.467188	1.573508
H	3.130006	8.886855	0.794664
C	1.951573	5.225594	3.677041
H	5.964093	2.139425	5.260065
H	5.811248	4.479494	6.050424
C	3.860583	6.219789	5.444893
C	-6.843175	-0.098001	0.726992
C	-7.953805	-0.477417	-0.052905
C	-5.654486	1.665462	-3.992399
C	-6.943276	2.187022	-3.763437
C	4.996335	-4.772167	-3.652112
C	5.056098	-6.170202	-3.818251
C	3.774624	-6.596337	1.034656
C	4.977134	-7.263278	0.727049
C	2.755508	6.983196	5.133240
C	1.775479	6.460289	4.268814
C	6.333582	6.634332	1.847895
C	6.183822	5.244464	1.683757
H	-4.721616	0.054408	0.846133
H	-8.594591	-1.388063	-1.886411
H	-3.722179	1.278544	-3.182927
H	-8.206815	3.222129	-2.372696
H	2.702602	-4.755524	1.001818
H	6.985319	-7.038888	0.006631
H	3.940000	-3.123910	-2.812040
H	4.056572	-8.034374	-3.461285
H	4.878002	3.642209	1.181665
H	5.383168	8.545960	1.634594
H	1.174981	4.834792	3.035243

H	4.597106	6.580337	6.157589
H	-6.991544	0.288814	1.730057
H	-8.959388	-0.360148	0.338882
H	-5.395934	1.267146	-4.968183
H	-7.686519	2.167748	-4.554416
H	5.747709	-4.138682	-4.112452
H	5.866874	-6.616607	-4.385534
H	2.962028	-7.135091	1.511193
H	5.082777	-8.321921	0.942791
H	2.622673	7.964013	5.579098
H	0.871409	7.026027	4.067358
H	7.290099	7.048003	2.151843
H	7.032697	4.585238	1.834813

MH 2 (MMMMPP)

138

C	-0.351890	1.035873	0.051932
C	0.879510	1.665609	0.422678
C	2.032422	0.869712	0.598177
C	2.068179	-0.451392	0.030250
C	0.860555	-1.061361	-0.372715
C	-0.317982	-0.251225	-0.527483
C	3.311370	-1.172175	-0.188273
C	0.848356	-2.501076	-0.572681
C	2.056268	-3.210022	-0.776134
C	3.305246	-2.572569	-0.392976
C	3.011510	1.383749	1.558477
C	1.077294	3.112425	0.440699
C	2.267399	3.641231	0.990936
C	3.001670	2.758794	1.893327
C	-1.416255	-0.556330	-1.447114
C	-1.668588	1.553022	0.414645
C	-2.823100	0.961534	-0.147490
C	-2.615771	0.190796	-1.370223
C	-1.242195	-1.452517	-2.540927
C	-3.551466	0.162869	-2.461397
C	-3.439569	-0.890039	-3.405140
C	-2.255684	-1.670900	-3.433415
C	-1.824988	2.504947	1.461316
C	-3.072726	2.892296	1.868432
C	-4.236970	2.241528	1.383851
C	-4.103103	1.161375	0.473183
C	-0.359661	-3.251573	-0.430637
C	-0.370552	-4.606601	-0.602823
C	0.770627	-5.272325	-1.131419
C	1.958689	-4.535019	-1.353824
C	4.548346	-0.472587	-0.340328
C	5.715579	-1.146994	-0.560757
C	5.760938	-2.563652	-0.434654
C	4.558365	-3.275566	-0.207435
C	3.847533	0.507138	2.308446
C	3.691369	3.217616	3.068412
C	4.666527	2.362335	3.642295
C	4.705094	0.998504	3.254234
C	0.193320	3.986834	-0.252182
C	0.456614	5.327749	-0.324600
C	1.689050	5.858902	0.136665
C	2.669878	4.982023	0.668706
H	-0.303924	-1.974237	-2.661481
H	-2.138811	-2.411624	-4.219176
H	-0.948268	2.931920	1.928379
H	-3.185442	3.669192	2.619123
H	-1.257663	-2.736594	-0.116180
H	-1.267413	-5.182030	-0.394217
H	4.536401	0.609226	-0.345852
H	6.632569	-0.606918	-0.776251
H	3.812679	-0.556296	2.121403
H	5.391007	0.330229	3.766756
H	-0.698416	3.586238	-0.714159
H	-0.255756	5.999461	-0.794862
C	4.658849	-4.656259	0.220514
C	6.995556	-3.274297	-0.556264
C	7.046171	-4.626524	-0.396603
C	5.893911	-5.347257	0.045895
C	0.739039	-6.672770	-1.417515
C	2.992311	-5.144372	-2.166119
C	1.818411	-7.303193	-1.959813
C	2.952769	-6.551076	-2.396286
C	3.604838	-5.340432	0.871906
H	7.890703	-2.709645	-0.800922
H	7.977712	-5.167993	-0.533555
C	5.975045	-6.721459	0.373150
H	-0.162195	-7.226730	-1.170785
C	4.010939	-4.398192	-2.805654
H	1.806427	-8.375163	-2.134309
C	4.007430	-7.169907	-3.108175
C	4.010942	5.506873	0.870869
C	1.940832	7.265845	0.078918
C	4.205112	6.919960	0.872664
C	3.125255	7.783199	0.504603
C	3.468924	4.507537	3.702879
C	5.595543	2.874438	4.601988
C	5.543002	4.174140	5.001887
C	4.456210	5.013291	4.599777
C	5.155810	4.688425	1.014503
H	1.148087	7.914393	-0.282886
C	5.481046	7.450421	1.173452
H	3.293878	8.856192	0.509913
C	2.291830	5.272456	3.527616
H	6.365540	2.206516	4.977398
H	6.283049	4.576131	5.687822
C	4.311286	6.312553	5.139221
C	-4.617066	1.133372	-2.657576
C	-4.510286	-1.154797	-4.316049
C	-5.702744	0.799104	-3.520404
C	-5.635094	-0.388820	-4.314929
C	-5.277017	0.343069	0.214114
C	-5.536353	2.673948	1.797207
C	-6.658106	2.046980	1.349657
C	-6.560972	0.843781	0.581793

C	-4.612064	2.426598	-2.084065
H	-4.415643	-2.004975	-4.985516
C	-6.801306	1.682090	-3.636601
H	-6.470318	-0.624895	-4.967952
C	-5.215127	-0.957509	-0.338980
H	-5.605262	3.538062	2.451820
H	-7.644798	2.410465	1.621855
C	-7.721453	0.110881	0.241281
C	-6.360383	-1.667680	-0.638439
C	-7.629492	-1.116178	-0.380797
C	-6.807396	2.897876	-2.986251
C	-5.682076	3.285649	-2.234939
C	5.013476	-5.020319	-3.519983
C	5.034870	-6.423681	-3.644365
C	3.716253	-6.673324	1.208603
C	4.899599	-7.383765	0.925186
C	3.192450	7.072398	4.870975
C	2.157723	6.526853	4.088106
C	6.398060	5.228421	1.280613
C	6.559852	6.621689	1.398157
H	2.697906	-4.804272	1.116178
H	6.914115	-7.240250	0.201910
H	3.997236	-3.318862	-2.738214
H	3.978082	-8.247306	-3.244803
H	5.059816	3.617490	0.906595
H	5.599490	8.530043	1.200789
H	1.474295	4.864114	2.950891
H	5.091674	6.691368	5.793345
H	-3.748835	2.755288	-1.523196
H	-7.633096	1.392466	-4.272704
H	-4.251644	-1.410088	-0.525189
H	-8.690979	0.525820	0.502435
H	-6.277167	-2.659728	-1.070761
H	-8.527273	-1.669816	-0.637497
H	-7.655927	3.567929	-3.083479
H	-5.649056	4.267614	-1.773852
H	5.786199	-4.422092	-3.991918
H	5.837547	-6.909373	-4.190480
H	2.887502	-7.173565	1.699140
H	4.974316	-8.437775	1.173998
H	3.091779	8.068236	5.291216
H	1.244521	7.090515	3.925958
H	7.254130	4.570987	1.394220
H	7.534663	7.040252	1.628308

MH 2 (MMMPPP)

138

C	-0.388857	1.212260	0.054224
C	0.841246	1.812466	0.476681
C	1.981815	0.995732	0.654528
C	2.007888	-0.306998	0.059530
C	0.793218	-0.900107	-0.355606
C	-0.362679	-0.073193	-0.534476
C	3.233068	-0.992919	-0.344791
C	0.759259	-2.360884	-0.369372
C	1.974006	-3.085047	-0.362511
C	3.170634	-2.344985	-0.754556
C	2.972413	1.470470	1.618153
C	1.063800	3.255272	0.539505
C	2.244805	3.751630	1.138565
C	2.963960	2.828512	2.012652
C	-1.447081	-0.380041	-1.464582
C	-1.709341	1.747344	0.378606
C	-2.855844	1.187759	-0.231624
C	-2.626277	0.400254	-1.440084
C	-1.272251	-1.324535	-2.515483
C	-3.523943	0.374154	-2.562289
C	-3.413450	-0.705732	-3.475896
C	-2.263065	-1.535516	-3.435107
C	-1.884616	2.691926	1.429359
C	-3.136159	3.115996	1.783901
C	-4.297158	2.505705	1.242421
C	-4.154747	1.422484	0.337238
C	-0.463025	-3.071840	-0.203222
C	-0.469901	-4.437778	-0.125161
C	0.740610	-5.172314	-0.031926
C	1.975527	-4.474689	0.004522
C	4.455480	-0.282265	-0.511264
C	5.573595	-0.918290	-0.977279
C	5.508560	-2.247435	-1.469984
C	4.257753	-2.916423	-1.501252
C	3.825717	0.562316	2.306759
C	3.652424	3.229510	3.208965
C	4.630297	2.348710	3.739011
C	4.684773	1.009463	3.273164
C	0.211053	4.163650	-0.149452
C	0.482806	5.504525	-0.158285
C	1.702343	6.008304	0.363742
C	2.663935	5.102116	0.880673
H	-0.352708	-1.889832	-2.575201
H	-2.152189	-2.308789	-4.189823
H	-1.017351	3.087491	1.939303
H	-3.257904	3.890407	2.535766
H	-1.394215	-2.524900	-0.153994
H	-1.411249	-4.977104	-0.071979
H	4.501144	0.766018	-0.251029
H	6.520330	-0.388674	-1.032326
H	3.803041	-0.487596	2.050209
H	5.383892	0.323314	3.742491
H	-0.667125	3.788695	-0.656316
H	-0.210555	6.199331	-0.623401
C	4.004037	5.608347	1.131219
C	1.962180	7.414951	0.377631
C	4.205114	7.018450	1.206357
C	3.137805	7.904809	0.857025
C	3.426921	4.486752	3.904197
C	5.553427	2.813480	4.728044
C	5.493991	4.089940	5.196055
C	4.407978	4.947011	4.831641
C	5.142763	4.777564	1.250753

H	1.181878	8.084177	0.026599
C	5.477635	7.525835	1.557404
H	3.311237	8.975315	0.917834
C	2.252722	5.261463	3.755104
H	6.324881	2.129990	5.071064
H	6.229395	4.457307	5.905969
C	4.259100	6.217787	5.434080
C	-4.551603	1.371497	-2.815415
C	-4.457659	-0.954901	-4.421374
C	-5.613806	1.054142	-3.712860
C	-5.554452	-0.151505	-4.480811
C	-5.338934	0.634921	0.033467
C	-5.600269	2.977845	1.596469
C	-6.718978	2.384458	1.098176
C	-6.622964	1.174996	0.340014
C	-4.530121	2.672533	-2.260471
H	-4.367663	-1.823083	-5.067996
C	-6.679543	1.967490	-3.885920
H	-6.371364	-0.374284	-5.161094
C	-5.290038	-0.673105	-0.503071
H	-5.672506	3.844550	2.247289
H	-7.705645	2.778423	1.324078
C	-7.787734	0.473010	-0.047867
C	4.167561	-4.145879	-2.272707
C	6.689760	-2.913791	-1.925041
C	5.370599	-4.815269	-2.645519
C	6.632609	-4.180819	-2.418289
C	3.161986	-5.220023	0.393947
C	0.722713	-6.601835	0.018313
C	1.877760	-7.312816	0.129246
C	3.121609	-6.645428	0.362241
C	2.945416	-4.693952	-2.727821
H	7.639150	-2.393740	-1.834650
C	5.300537	-6.067449	-3.299026
H	7.537418	-4.705215	-2.711695
C	4.349403	-4.609048	0.860744
H	-0.235433	-7.106174	-0.068650
H	1.866761	-8.398783	0.111719
C	4.297024	-7.384547	0.630677
C	5.477572	-5.351958	1.145113
C	5.466818	-6.751319	0.993604
C	2.901592	-5.904321	-3.390170
C	4.086147	-6.619943	-3.647038
C	-7.703474	-0.762062	-0.655011
C	-6.440699	-1.353132	-0.848193
C	-5.567481	3.559883	-2.465524
C	-6.673591	3.193589	-3.255232
C	3.142845	6.990929	5.194610
C	2.114993	6.486812	4.375663
C	6.548299	6.680739	1.758849
C	6.382626	5.296362	1.565634
H	5.044108	3.714436	1.083844
H	5.600699	8.602020	1.641238
H	1.441017	4.883797	3.149846
H	5.034453	6.563255	6.112190

H	-3.680267	2.983674	-1.669854
H	-7.495069	1.691858	-4.548713
H	-4.332714	-1.155883	-0.638560
H	-8.755580	0.917915	0.165869
H	2.025838	-4.151020	-2.561997
H	6.228726	-6.575906	-3.544552
H	4.373583	-3.538525	1.006663
H	4.252511	-8.468308	0.568802
H	6.375101	-4.850397	1.492708
H	6.362924	-7.329054	1.197633
H	1.945904	-6.300771	-3.717930
H	4.044595	-7.581620	-4.148897
H	-8.604447	-1.291957	-0.948045
H	-6.367038	-2.352095	-1.266039
H	-5.523534	4.547399	-2.017397
H	-7.497044	3.886827	-3.395800
H	3.039125	7.964683	5.663074
H	1.204336	7.059734	4.232803
H	7.520683	7.081578	2.027564
H	7.233863	4.629510	1.658636

MH 2 (MMPMMM)
138

C	-0.610212	1.066701	-0.234131
C	0.630731	1.785936	-0.003617
C	1.820422	1.030470	0.041280
C	1.710333	-0.406054	0.190093
C	0.599538	-1.050111	-0.392407
C	-0.516131	-0.216677	-0.805940
C	2.625495	-1.166069	1.011941
C	0.587726	-2.505560	-0.425073
C	1.754950	-3.212415	-0.026426
C	2.597061	-2.581025	0.973556
C	3.089750	1.684777	-0.185050
C	0.735112	3.236876	0.055359
C	2.001569	3.825460	0.321177
C	3.173218	3.097014	-0.130826
C	-1.585049	-0.625137	-1.692574
C	-1.939479	1.591390	-0.003165
C	-3.033791	1.151823	-0.789923
C	-2.882394	-0.069182	-1.560190
C	-1.312039	-1.475664	-2.807882
C	-3.965251	-0.792772	-2.197687
C	-3.665716	-1.551164	-3.355635
C	-2.305387	-1.826683	-3.677193
C	-2.195400	2.440704	1.117102
C	-3.456224	2.901297	1.369731
C	-4.483010	2.742174	0.394963
C	-4.219950	1.985719	-0.773082
C	-0.548871	-3.301251	-0.769975
C	-0.440958	-4.651119	-0.957006
C	0.827747	-5.292000	-0.934374
C	1.962858	-4.547969	-0.531751
C	-0.342275	4.140373	-0.203212
C	-0.222869	5.478892	0.047552

C	0.933898	6.001192	0.687834
C	2.044257	5.148113	0.896530
H	-0.288169	-1.781547	-2.984729
H	-2.078803	-2.382231	-4.582057
H	-1.380599	2.656988	1.797160
H	-3.671616	3.456094	2.277780
H	-1.519713	-2.836744	-0.842337
H	-1.326861	-5.243363	-1.165409
H	-1.253030	3.767820	-0.645106
H	-1.034512	6.153891	-0.206646
C	4.260009	0.936689	-0.518278
C	4.387760	3.744765	-0.577016
C	5.472739	1.552390	-0.628304
C	5.572317	2.971308	-0.630813
C	3.354743	-3.323904	1.957313
C	3.528412	-0.518259	1.909341
C	4.413937	-2.659206	2.621079
C	4.457819	-1.237591	2.602651
H	4.185873	-0.134723	-0.638023
H	6.374154	0.967148	-0.785647
H	3.500875	0.558110	2.001065
H	5.204492	-0.732049	3.208225
C	3.267165	-5.174483	-0.624708
C	0.948633	-6.686207	-1.221084
C	2.150782	-7.318937	-1.125875
C	3.343880	-6.580529	-0.854260
C	3.096475	-4.701174	2.336795
C	5.401159	-3.414215	3.328519
C	5.311551	-4.770283	3.419913
C	4.130815	-5.445045	2.978297
C	6.845126	3.616751	-0.723047
C	4.482090	5.130423	-0.999669
C	6.936650	4.972811	-0.812610
C	5.761020	5.762129	-1.010936
C	1.017457	7.384822	1.032857
C	3.171866	5.655634	1.653715
C	3.247019	7.053647	1.928561
C	2.152854	7.903344	1.577446
C	-5.770150	3.337052	0.578634
C	-6.728221	3.253040	-0.387190
C	-6.418819	2.697149	-1.666808
C	-5.131122	2.126260	-1.889867
C	-5.325725	-0.822655	-1.702347
C	-4.728822	-2.031612	-4.182269
C	-6.032516	-1.837329	-3.835254
C	-6.367166	-1.280119	-2.562581
C	4.482534	-4.454171	-0.537701
H	0.050036	-7.237101	-1.483651
H	2.230900	-8.388263	-1.298838
C	4.605490	-7.220714	-0.852466
C	1.838141	-5.328570	2.178561
H	6.240110	-2.879899	3.765157
H	6.091614	-5.349212	3.905811
C	3.935709	-6.819812	3.250049
H	7.737596	2.998412	-0.689316
C	3.379007	5.873124	-1.483096
H	7.903835	5.466991	-0.822825
C	5.854909	7.142287	-1.307952
H	0.163546	8.020923	0.818109
C	4.193684	4.826170	2.174720
C	4.386628	7.576319	2.584016
H	2.229724	8.964588	1.795727
H	-5.970237	3.847392	1.516465
H	-7.717129	3.672646	-0.226888
C	-7.345471	2.761877	-2.734197
C	-4.776167	1.793282	-3.219080
C	-5.666686	-0.490331	-0.369314
H	-4.470013	-2.544874	-5.103998
H	-6.836163	-2.169618	-4.485982
C	-7.708994	-1.234063	-2.115897
C	5.403761	6.748988	3.008735
C	5.287996	5.357110	2.825522
C	4.740026	7.865260	-1.674744
C	3.500439	7.209223	-1.805482
C	-6.979263	-0.482935	0.053738
C	-8.017045	-0.823280	-0.836700
C	-5.686312	1.894241	-4.250234
C	-6.996725	2.349273	-4.002313
C	5.767843	-6.499540	-0.684378
C	5.702239	-5.098378	-0.555100
C	2.727846	-7.429473	2.986189
C	1.657828	-6.661230	2.487215
H	4.454758	-3.376275	-0.456820
H	4.640225	-8.296167	-1.002670
H	0.997104	-4.747761	1.825822
H	4.749506	-7.379091	3.703128
H	2.426719	5.379510	-1.619396
H	6.832684	7.614580	-1.272808
H	4.114742	3.753777	2.061131
H	4.436414	8.647202	2.760357
H	-8.333717	3.168560	-2.538426
H	-3.768636	1.461177	-3.429073
H	-4.880216	-0.245799	0.331548
H	-8.492927	-1.554314	-2.796510
H	6.274472	7.162145	3.508310
H	6.060081	4.694702	3.204022
H	4.823542	8.922825	-1.905002
H	2.633752	7.753354	-2.166464
H	-7.210501	-0.216943	1.080137
H	-9.050107	-0.795509	-0.504369
H	-5.387096	1.624877	-5.258020
H	-7.715320	2.407053	-4.813924
H	6.729905	-7.002379	-0.682166
H	6.616123	-4.518197	-0.475358
H	2.586890	-8.483837	3.202725
H	0.682223	-7.115426	2.347801

MH 2 (MMPMMP)

138

C	-0.603365	1.194210	-0.296794

C	0.643120	1.878073	-0.052324	C	1.882069	-7.287657	-1.022072
C	1.812232	1.088965	0.037007	C	3.091950	-6.594892	-0.707011
C	1.656009	-0.340019	0.201807	C	2.793925	-4.654756	2.446270
C	0.536064	-0.958175	-0.398842	C	5.116775	-3.452233	3.501236
C	-0.531433	-0.101507	-0.854378	C	4.962152	-4.800741	3.613861
C	2.525440	-1.126342	1.050231	C	3.769365	-5.430912	3.139682
C	0.485902	-2.410705	-0.441044	C	6.902141	3.562633	-0.684873
C	1.607473	-3.154868	0.010640	C	4.571890	5.113950	-1.028317
C	2.437774	-2.539578	1.032807	C	7.020589	4.914119	-0.805459
C	3.102230	1.714013	-0.160769	C	5.862828	5.720676	-1.037377
C	0.776732	3.324404	0.016861	C	1.129707	7.475903	0.939944
C	2.050895	3.888971	0.288185	C	3.248078	5.717573	1.600021
C	3.213072	3.125428	-0.134980	C	3.349892	7.118641	1.848397
C	-1.525719	-0.376023	-1.882470	C	2.274084	7.982921	1.476044
C	-1.951664	1.643625	0.021430	C	4.303046	-4.512019	-0.372141
C	-3.053907	1.074293	-0.661581	H	-0.193628	-7.123691	-1.481942
C	-2.731175	0.366992	-1.900162	H	1.924757	-8.361965	-1.176355
C	-1.219974	-1.229312	-2.978849	C	4.324679	-7.285911	-0.642490
C	-3.542106	0.381465	-3.084917	C	1.516685	-5.230604	2.248417
C	-3.302953	-0.618184	-4.065389	H	5.962945	-2.947669	3.958836
C	-2.119717	-1.398730	-3.995738	H	5.696709	-5.404313	4.139084
C	-2.189821	2.490602	1.139249	C	3.503709	-6.790626	3.426732
C	-3.468397	2.823623	1.495167	H	7.782222	2.928826	-0.623802
C	-4.586285	2.216994	0.865503	C	3.489048	5.867517	-1.539859
C	-4.377597	1.220211	-0.124765	H	7.997051	5.389708	-0.814273
C	-0.659018	-3.155703	-0.857050	C	5.987132	7.091808	-1.363465
C	-0.597652	-4.510679	-1.029564	H	0.288188	8.123986	0.712397
C	0.638557	-5.204070	-0.922240	C	4.248361	4.877631	2.145118
C	1.783691	-4.502643	-0.472110	C	4.495843	7.630883	2.500854
C	-0.293523	4.235768	-0.233860	H	2.371176	9.046519	1.673770
C	-0.152774	5.575254	0.001711	C	-5.524410	0.423404	-0.533788
C	1.019509	6.087428	0.621300	C	-5.915719	2.609474	1.219237
C	2.114950	5.217985	0.845063	C	-6.997768	2.020741	0.641489
H	-0.273860	-1.754593	-2.992343	C	-6.835780	0.886994	-0.215709
H	-1.912445	-2.101719	-4.797325	C	-4.601075	1.344624	-3.346084
H	-1.347706	2.880944	1.695793	C	-4.253425	-0.838843	-5.111551
H	-3.645020	3.523596	2.306668	C	-5.380933	-0.083104	-5.205399
H	-1.597626	-2.643528	-1.004538	C	-5.566145	1.052659	-4.355624
H	-1.491778	-5.068171	-1.292208	C	-5.414511	-0.828927	-1.182383
H	-1.217934	3.863922	-0.648795	H	-6.038015	3.413552	1.939291
H	-0.957810	6.260324	-0.246974	H	-8.004308	2.356692	0.873350
C	4.264034	0.936887	-0.453298	C	-7.963560	0.179393	-0.692310
C	4.445536	3.739523	-0.577389	C	-4.697532	2.596056	-2.693601
C	5.490104	1.527274	-0.557546	H	-4.066153	-1.648098	-5.811561
C	5.616238	2.943227	-0.595471	H	-6.125749	-0.286968	-5.969227
C	3.126538	-3.296667	2.054699	C	-6.659138	1.927788	-4.553746
C	3.429188	-0.503378	1.963712	C	1.266431	-6.548496	2.571604
C	4.191031	-2.667549	2.744627	C	2.281464	-7.352564	3.125709
C	4.302023	-1.250529	2.700323	C	5.507878	-6.609923	-0.435888
H	4.171729	-0.135553	-0.548741	C	5.494914	-5.205444	-0.329703
H	6.382766	0.921555	-0.684433	C	-6.771666	3.098600	-3.834250
H	3.447607	0.574663	2.035842	C	-5.758375	3.449233	-2.922745
H	5.051327	-0.767426	3.320803	C	-6.530028	-1.517947	-1.614458
C	3.063815	-5.183755	-0.499812	C	-7.818694	-0.993750	-1.402143
C	0.713914	-6.606843	-1.183311	C	5.348852	5.398752	2.793619

C	5.492628	6.791271	2.949279
C	4.890951	7.828127	-1.759032
C	3.640181	7.193760	-1.889418
H	4.316442	-3.432737	-0.308252
H	4.321241	-8.364072	-0.776263
H	0.716181	-4.621026	1.852638
H	4.274214	-7.376057	3.920623
H	2.528663	5.389900	-1.676158
H	6.973497	7.545765	-1.326862
H	4.147453	3.805062	2.052904
H	4.566580	8.703784	2.656462
H	-4.437542	-1.263861	-1.334798
H	-8.952280	0.568090	-0.464888
H	-3.919392	2.898732	-2.008193
H	-7.399056	1.669340	-5.306145
H	0.278096	-6.962822	2.401061
H	2.086293	-8.395687	3.354278
H	6.447433	-7.151369	-0.386139
H	6.427876	-4.661626	-0.220391
H	-7.614815	3.763046	-3.995546
H	-5.804086	4.398567	-2.398610
H	-6.407955	-2.472932	-2.115586
H	-8.691919	-1.528731	-1.762258
H	6.103983	4.728175	3.191380
H	6.368385	7.196284	3.446689
H	4.997955	8.878521	-2.011344
H	2.788395	7.746646	-2.271949

MH 2 (MMPMPM)
138

C	-0.697624	1.099037	-0.272666
C	0.543947	1.787728	0.038851
C	1.725038	1.011929	0.066030
C	1.600807	-0.427911	0.182070
C	0.463842	-1.045435	-0.371880
C	-0.599593	-0.179920	-0.851772
C	2.631846	-1.234870	0.794166
C	0.276542	-2.480682	-0.174405
C	1.332140	-3.258533	0.377602
C	2.626402	-2.633831	0.587746
C	2.996253	1.664456	-0.171298
C	0.675169	3.231558	0.184390
C	1.949686	3.775471	0.505292
C	3.105895	3.067192	-0.013135
C	-1.617855	-0.573941	-1.796780
C	-2.033293	1.642867	-0.123689
C	-3.073013	1.245699	-1.002389
C	-2.902160	0.023632	-1.767865
C	-1.294376	-1.483620	-2.849350
C	-3.957473	-0.674072	-2.475899
C	-3.595587	-1.469860	-3.590735
C	-2.228633	-1.821627	-3.786153
C	-2.364738	2.461451	0.999382
C	-3.629908	2.952136	1.158170
C	-4.572082	2.852805	0.094337
C	-4.234182	2.111480	-1.064118
C	-0.962113	-3.138406	-0.446507
C	-1.075101	-4.497547	-0.369552
C	-0.050635	-5.281733	0.221256
C	1.063839	-4.620652	0.787918
C	-0.373791	4.178865	-0.027954
C	-0.234226	5.489244	0.337417
C	0.918704	5.932542	1.040449
C	2.013344	5.045790	1.184072
H	-0.276119	-1.847348	-2.920775
H	-1.958139	-2.423327	-4.648554
H	-1.605888	2.634515	1.752626
H	-3.907283	3.485955	2.061940
H	-1.822247	-2.565995	-0.750028
H	-1.992718	-4.986479	-0.683814
H	-1.279774	3.866429	-0.521780
H	-1.027252	6.199547	0.123918
C	4.133558	0.934981	-0.634770
C	4.319393	3.732294	-0.434612
C	5.353657	1.537619	-0.737391
C	5.485841	2.946117	-0.596321
C	3.879422	-3.341881	0.706591
C	3.655399	-0.687020	1.626236
C	4.890294	-2.768564	1.517287
C	4.707826	-1.455026	2.035914
H	4.032459	-0.114777	-0.869424
H	6.233024	0.954770	-0.995540
H	3.572158	0.342788	1.947521
H	5.451247	-1.051797	2.716886
C	6.769453	3.573639	-0.660788
C	4.432498	5.148717	-0.734565
C	6.887078	4.930017	-0.621551
C	5.723375	5.755724	-0.716867
C	1.019803	7.279069	1.505745
C	3.149184	5.471627	1.978541
C	3.241207	6.839010	2.375684
C	2.158809	7.730636	2.100069
C	-5.850512	3.486795	0.182007
C	-6.725868	3.452392	-0.862128
C	-6.330560	2.903820	-2.121015
C	-5.048476	2.291968	-2.247767
C	-5.353789	-0.651495	-2.090169
C	-4.606128	-1.921370	-4.496242
C	-5.924602	-1.666800	-4.262861
C	-6.339368	-1.079508	-3.027690
H	7.649019	2.938160	-0.710812
C	3.336673	5.950426	-1.132212
H	7.863703	5.405240	-0.609219
C	5.838790	7.154998	-0.891327
H	0.177034	7.944613	1.342322
C	4.164092	4.588493	2.418375
C	4.386776	7.288272	3.073650
H	2.248980	8.767152	2.412025
H	-6.112732	3.986683	1.110114
H	-7.710338	3.903206	-0.776737

C	-7.165886	3.010830	-3.258043
C	-4.601626	1.954790	-3.547945
C	-5.786896	-0.301640	-0.788927
H	-4.293634	-2.462246	-5.384948
H	-6.684917	-1.976480	-4.973991
C	-7.709956	-0.980909	-2.690086
C	1.836829	-5.361035	1.770087
C	-0.157625	-6.706185	0.299547
C	0.771729	-7.439007	0.972982
C	1.757088	-6.785117	1.777929
C	4.152257	-4.608483	0.057111
C	5.289422	-5.365638	0.467286
C	6.210971	-4.804455	1.404176
C	6.047308	-3.532178	1.862284
C	2.604017	-4.741419	2.784570
H	-0.984686	-7.187743	-0.214231
H	0.724906	-8.524077	0.985819
C	2.588386	-7.525449	2.650947
C	3.364151	-5.121329	-1.000837
C	5.513771	-6.645994	-0.091344
H	7.070125	-5.396852	1.705299
H	6.780776	-3.082441	2.525417
C	-8.103354	-0.550966	-1.440877
C	-7.127758	-0.245112	-0.471277
C	-5.423034	2.095434	-4.646736
C	-6.731788	2.596454	-4.498918
C	5.265299	5.047368	3.111165
C	5.395513	6.415425	3.420137
C	3.478411	7.307484	-1.338297
C	4.731951	7.927606	-1.171515
C	3.612972	-6.365285	-1.542625
C	4.680241	-7.151468	-1.065568
C	3.376422	-5.484020	3.654154
C	3.406756	-6.888870	3.559657
H	2.373723	5.487388	-1.297453
H	6.826196	7.604609	-0.834214
H	4.074978	3.531860	2.206977
H	4.448572	8.338497	3.344922
H	-8.152196	3.449970	-3.136778
H	-3.593326	1.586977	-3.680979
H	-5.050131	-0.084588	-0.027798
H	-8.448436	-1.278155	-3.429420
H	2.571209	-3.665710	2.890502
H	2.543050	-8.610292	2.613746
H	2.551203	-4.525568	-1.392532
H	6.370213	-7.218528	0.253714
H	-9.157825	-0.483491	-1.191964
H	-7.429523	0.033007	0.533317
H	-5.054693	1.822208	-5.630335
H	-7.380892	2.686563	-5.364352
H	6.031804	4.345764	3.424929
H	6.271163	6.772724	3.953043
H	2.616755	7.896123	-1.635941
H	4.832024	8.999789	-1.308859
H	2.984062	-6.735621	-2.345986
H	4.861200	-8.136158	-1.485166
H	3.959473	-4.978605	4.417154
H	4.036935	-7.466447	4.228774

MH 2 (MMPPMP)

138

C	-0.602024	1.416255	-0.099056
C	0.711028	2.013352	-0.036310
C	1.810866	1.145321	0.128634
C	1.544813	-0.247677	0.426388
C	0.356196	-0.825229	-0.081012
C	-0.678718	0.074773	-0.530833
C	2.359312	-1.026315	1.338464
C	0.214153	-2.272044	-0.020114
C	1.310590	-3.053316	0.432480
C	2.194817	-2.431581	1.403888
C	3.154270	1.674287	0.057774
C	0.951470	3.405231	-0.396666
C	2.280363	3.908014	-0.419561
C	3.352741	3.074442	0.097315
C	-1.786460	-0.198893	-1.437887
C	-1.866576	2.024437	0.272952
C	-3.074818	1.477139	-0.220973
C	-2.938150	0.625584	-1.401631
C	-1.647768	-1.149906	-2.486740
C	-3.871647	0.593362	-2.493322
C	-3.803025	-0.501772	-3.394834
C	-2.661929	-1.345298	-3.384175
C	-1.914323	3.042710	1.265475
C	-3.118158	3.524345	1.701398
C	-4.339232	2.926096	1.290843
C	-4.315603	1.799958	0.424959
C	-0.985906	-2.977634	-0.336943
C	-0.999078	-4.340985	-0.445072
C	0.204773	-5.091430	-0.358662
C	1.400027	-4.430784	0.017607
C	-0.104353	4.275859	-0.802391
C	0.124646	5.601696	-1.039272
C	1.448408	6.105184	-1.134485
C	2.529202	5.198639	-1.025639
H	-0.737923	-1.731761	-2.555281
H	-2.581506	-2.121575	-4.139581
H	-0.985764	3.442015	1.654642
H	-3.155079	4.344538	2.412451
H	-1.905393	-2.427548	-0.463670
H	-1.931385	-4.863176	-0.638334
H	-1.109324	3.895988	-0.885626
H	-0.705356	6.272635	-1.241534
C	4.304827	0.840444	-0.088688
C	4.633799	3.564836	0.549911
C	5.563336	1.362156	0.005939
C	5.757813	2.713783	0.409204
C	2.850342	-3.170110	2.460307
C	3.270993	-0.400698	2.242543
C	3.942912	-2.557525	3.120602

C	4.104676	-1.148789	3.022492
H	4.163457	-0.210601	-0.303137
H	6.433270	0.736409	-0.169227
H	3.324959	0.677461	2.281876
H	4.861645	-0.667010	3.634512
C	3.807442	5.630283	-1.563670
C	1.697109	7.491917	-1.383469
C	4.045677	7.025497	-1.738547
C	2.964436	7.948839	-1.581704
C	4.833908	4.879720	1.125901
C	7.074409	3.231242	0.608744
C	7.271614	4.532526	0.959733
C	6.162276	5.381820	1.259819
C	-4.889016	1.604456	-2.738587
C	-4.877948	-0.749640	-4.305894
C	-5.980358	1.289900	-3.602224
C	-5.963287	0.069804	-4.348981
C	-5.554029	1.060224	0.239362
C	-5.588759	3.463684	1.732871
C	-6.768169	2.897190	1.358909
C	-6.784664	1.661863	0.638345
C	4.807214	4.732857	-2.007191
H	0.850986	8.173096	-1.383730
C	5.323470	7.468139	-2.154020
H	3.161186	9.008761	-1.714235
C	3.772626	5.684222	1.605342
H	7.917901	2.566926	0.443852
H	8.275606	4.933031	1.066185
C	6.365644	6.700332	1.730881
C	-4.827039	2.918458	-2.218215
H	-4.818879	-1.629125	-4.940713
C	-7.031594	2.220879	-3.769689
H	-6.801922	-0.150594	-5.003081
C	-5.605857	-0.259450	-0.267448
H	-5.572420	4.356858	2.350887
H	-7.715620	3.338070	1.655045
C	-8.005848	0.998820	0.375749
C	0.199045	-6.506367	-0.555968
C	1.335418	-7.239929	-0.399309
C	2.588547	-6.599657	-0.148956
C	2.640995	-5.181123	-0.006698
C	2.458901	-4.491351	2.919137
C	4.839232	-3.347456	3.906874
C	4.629227	-4.682413	4.076502
C	3.405775	-5.279466	3.638331
H	-0.743428	-6.987749	-0.800853
H	1.317648	-8.320800	-0.505080
C	3.783896	-7.353183	-0.081989
C	3.916994	-4.573133	0.065751
C	1.152917	-5.013555	2.766231
H	5.708071	-2.859594	4.339396
H	5.341184	-5.294238	4.622895
C	3.081469	-6.610888	3.990174
C	5.302601	7.488794	2.115225
C	3.997157	6.960404	2.078387
C	6.033597	5.186672	-2.447848
C	6.314030	6.566469	-2.480315
C	-5.850375	3.822971	-2.418995
C	-6.984604	3.461375	-3.169613
C	-6.808461	-0.899086	-0.491311
C	-8.024850	-0.253267	-0.201139
C	5.070734	-5.328076	0.113578
C	5.006255	-6.734340	0.068052
C	1.830162	-7.127728	3.730068
C	0.845628	-6.301849	3.153698
H	4.599901	3.671549	-2.015547
H	5.498087	8.536730	-2.243014
H	2.765864	5.289657	1.601895
H	7.383377	7.074273	1.799635
H	-3.955083	3.228422	-1.660989
H	-7.868753	1.947057	-4.405743
H	-4.684883	-0.784368	-0.475522
H	-8.932542	1.489047	0.660832
H	3.719544	-8.434392	-0.166799
H	3.989560	-3.494507	0.084108
H	0.375463	-4.384500	2.355285
H	3.830459	-7.208574	4.502188
H	5.470991	8.499613	2.473426
H	3.161124	7.556772	2.429738
H	6.782590	4.472748	-2.774725
H	7.289660	6.916610	-2.802760
H	-5.772591	4.820545	-1.998553
H	-7.796879	4.168384	-3.306533
H	-6.811339	-1.909264	-0.888268
H	-8.968446	-0.752715	-0.397547
H	6.033706	-4.831688	0.180286
H	5.916485	-7.323479	0.120988
H	1.589836	-8.149025	4.008720
H	-0.163972	-6.675270	3.016483

MH 2 (MMPPPM)

138

C	-0.480006	1.127317	-0.217047
C	0.819175	1.752528	-0.027862
C	1.942340	0.903067	0.047282
C	1.724730	-0.528819	0.181184
C	0.542878	-1.084843	-0.350722
C	-0.497077	-0.161818	-0.776347
C	2.697027	-1.393231	0.813621
C	0.291459	-2.512571	-0.162698
C	1.324004	-3.352306	0.343352
C	2.640125	-2.785620	0.569576
C	3.270961	1.474584	0.020400
C	1.033430	3.184775	-0.227010
C	2.345695	3.726121	-0.116248
C	3.431206	2.854047	0.290669
C	-1.607970	-0.494834	-1.639649
C	-1.756169	1.764520	0.018761
C	-2.894069	1.406796	-0.746236
C	-2.853750	0.166059	-1.500622

C	-1.422275	-1.409027	-2.721180
C	-4.000691	-0.482548	-2.104758
C	-3.780216	-1.296880	-3.242920
C	-2.453358	-1.703461	-3.566842
C	-1.908519	2.675320	1.108315
C	-3.117888	3.255403	1.365044
C	-4.175115	3.151588	0.415160
C	-4.001219	2.342338	-0.734590
C	-0.983643	-3.108980	-0.407889
C	-1.150601	-4.464369	-0.383845
C	-0.141579	-5.312925	0.140581
C	1.006660	-4.717072	0.711299
C	-0.019839	4.077151	-0.595807
C	0.169011	5.429724	-0.615446
C	1.472442	5.984016	-0.531534
C	2.576883	5.101478	-0.508236
H	-0.432057	-1.817775	-2.884889
H	-2.290238	-2.312809	-4.450577
H	-1.057081	2.849952	1.755589
H	-3.265059	3.863504	2.252433
H	-1.831746	-2.492501	-0.649753
H	-2.098551	-4.901629	-0.683914
H	-0.996125	3.692562	-0.834090
H	-0.672569	6.094600	-0.787283
C	4.445084	0.730105	-0.311758
C	4.697999	3.293245	0.828630
C	5.687998	1.266665	-0.130228
C	5.845134	2.513689	0.539467
C	3.865490	-3.541655	0.687573
C	3.699355	-0.925915	1.719235
C	4.874059	-3.036737	1.544943
C	4.708789	-1.749095	2.130497
H	4.335633	-0.258673	-0.737227
H	6.573223	0.718178	-0.437053
H	3.632435	0.085144	2.098697
H	5.434073	-1.405775	2.861731
C	4.108202	-4.789620	-0.006572
C	6.002772	-3.847642	1.873898
C	5.218366	-5.595161	0.385571
C	6.140845	-5.101081	1.358375
C	1.755028	-5.521525	1.661483
C	-0.300399	-6.734555	0.162990
C	0.609740	-7.527069	0.794015
C	1.623627	-6.941082	1.615740
C	3.856915	5.647407	-0.924815
C	1.682870	7.399065	-0.541732
C	4.056951	7.058293	-0.862117
C	2.941554	7.915511	-0.601990
C	4.858384	4.490461	1.627994
C	7.145710	3.015344	0.850161
C	7.305940	4.230975	1.443875
C	6.172782	4.987126	1.874690
C	3.314912	-5.239270	-1.089252
H	6.735488	-3.450353	2.570510
C	5.413303	-6.857728	-0.222505

H	6.978941	-5.729837	1.644828
C	2.540155	-4.968646	2.700349
H	-1.151890	-7.164253	-0.356827
H	0.523651	-8.609408	0.764434
C	2.428577	-7.743335	2.458190
C	4.892688	4.862254	-1.483459
H	0.814128	8.047752	-0.475554
C	5.331999	7.595475	-1.156982
H	3.108472	8.987606	-0.552026
C	3.770222	5.180507	2.214056
H	8.007696	2.414800	0.573922
H	8.298400	4.626591	1.639591
C	6.338134	6.204172	2.577067
C	-5.402436	3.860904	0.602769
C	-4.915986	2.535343	-1.841108
C	-6.382053	3.835091	-0.344500
C	-6.144942	3.222160	-1.613505
C	-5.352798	-0.387668	-1.593659
C	-4.890219	-1.708462	-4.044838
C	-6.168648	-1.396079	-3.690364
C	-6.439534	-0.779828	-2.429775
H	-5.539847	4.411831	1.528845
C	-4.613742	2.142298	-3.166868
H	-7.327737	4.343308	-0.179950
C	-7.080728	3.341901	-2.668018
C	-5.647844	0.003925	-0.265846
H	-4.686801	-2.267063	-4.954016
H	-7.005317	-1.677422	-4.323222
C	-7.767057	-0.612573	-1.969611
C	-6.949718	0.128804	0.171791
C	-8.023552	-0.146656	-0.697938
C	-5.528886	2.298515	-4.186722
C	-6.790709	2.870904	-3.930587
C	6.115583	5.413940	-1.806655
C	6.356872	6.785773	-1.598380
C	3.957861	6.357487	2.908384
C	5.251036	6.892805	3.070295
C	3.534205	-6.467657	-1.677104
C	4.576105	-7.300640	-1.223601
C	3.284037	-5.770983	3.541295
C	3.267106	-7.171227	3.391142
H	2.520801	-4.606934	-1.462025
H	6.249520	-7.467720	0.107714
H	2.542619	-3.897454	2.848884
H	2.344968	-8.823681	2.379477
H	4.715578	3.813149	-1.677193
H	5.476800	8.668106	-1.063028
H	2.771970	4.776724	2.114593
H	7.345875	6.579151	2.732749
H	-3.642271	1.719801	-3.383980
H	-8.026315	3.837992	-2.467775
H	-4.835090	0.199582	0.420122
H	-8.583444	-0.884489	-2.632867
H	-7.144619	0.437088	1.193885
H	-9.046388	-0.026271	-0.354819

H	-5.270764	1.981637	-5.192040
H	-7.515270	2.972087	-4.732579
H	6.892410	4.783915	-2.227501
H	7.329821	7.208832	-1.828214
H	3.102102	6.869383	3.337075
H	5.390490	7.827064	3.605230
H	2.901982	-6.789490	-2.498599
H	4.734297	-8.272484	-1.680698
H	3.881578	-5.316248	4.324662
H	3.875915	-7.795840	4.037395

MH 2 (MMPPPP)

138

C	-0.557293	1.244086	-0.150517
C	0.751266	1.834933	0.015861
C	1.857450	0.958119	0.069198
C	1.605953	-0.467777	0.201786
C	0.399809	-0.989997	-0.315835
C	-0.613939	-0.042999	-0.721742
C	2.559482	-1.366499	0.815501
C	0.114671	-2.411692	-0.158116
C	1.115751	-3.282400	0.353312
C	2.450134	-2.757911	0.579702
C	3.196044	1.505444	0.021663
C	0.984331	3.264481	-0.155709
C	2.308503	3.778717	-0.093132
C	3.389612	2.884035	0.277952
C	-1.684728	-0.240810	-1.683116
C	-1.842520	1.803617	0.230220
C	-3.023595	1.327825	-0.389112
C	-2.828985	0.591943	-1.636744
C	-1.519885	-1.142060	-2.772165
C	-3.705914	0.654624	-2.773127
C	-3.606692	-0.374887	-3.746378
C	-2.490911	-1.253890	-3.728650
C	-1.942973	2.695586	1.334624
C	-3.166042	3.144835	1.749938
C	-4.365361	2.621410	1.197109
C	-4.298514	1.596201	0.216558
C	-1.170431	-2.964621	-0.443158
C	-1.381374	-4.314024	-0.421383
C	-0.413713	-5.191877	0.133225
C	0.747557	-4.634911	0.718003
C	-0.074032	4.176385	-0.450303
C	0.139358	5.525385	-0.472725
C	1.457031	6.052849	-0.463160
C	2.546264	5.150898	-0.491094
H	-0.627667	-1.754440	-2.816773
H	-2.395912	-1.988722	-4.522802
H	-1.036277	3.031879	1.822329
H	-3.238651	3.876711	2.549169
H	-1.983143	-2.316227	-0.723491
H	-2.332050	-4.724338	-0.749821
H	-1.071058	3.805202	-0.616690
H	-0.696734	6.209545	-0.586260
C	4.347536	0.734925	-0.330457
C	4.680215	3.303869	0.773773
C	5.604824	1.249575	-0.190060
C	5.804396	2.502216	0.457085
C	3.646826	-3.558901	0.698723
C	3.588534	-0.930275	1.706796
C	4.677740	-3.087677	1.548357
C	4.568190	-1.787806	2.119382
H	4.208286	-0.255701	-0.742369
H	6.470487	0.680963	-0.515609
H	3.563209	0.085529	2.078156
H	5.310995	-1.465832	2.842744
C	3.840357	-4.819306	0.011246
C	5.773075	-3.941045	1.882843
C	4.916560	-5.666844	0.409454
C	5.858682	-5.203761	1.378694
C	1.460230	-5.462890	1.675143
C	-0.623327	-6.606865	0.160244
C	0.250106	-7.428761	0.805295
C	1.277504	-6.876752	1.633561
C	3.818079	5.674409	-0.958004
C	1.692389	7.463868	-0.484745
C	4.044621	7.081579	-0.906115
C	2.955924	7.958137	-0.601646
C	4.886442	4.503377	1.559431
C	7.122537	2.985631	0.719172
C	7.321916	4.204795	1.293470
C	6.215859	4.982096	1.755956
C	-4.696152	1.697454	-2.989755
C	-4.631465	-0.529919	-4.732144
C	-5.739273	1.473396	-3.937115
C	-5.697046	0.315485	-4.775528
C	-5.528856	0.894753	-0.113991
C	-5.634797	3.129546	1.616861
C	-6.793912	2.626090	1.111881
C	-6.776719	1.471504	0.268029
C	3.032547	-5.241717	-1.071694
H	6.522746	-3.567684	2.574627
C	5.060306	-6.940149	-0.190492
H	6.670431	-5.864032	1.670034
C	2.261686	-4.934889	2.714521
H	-1.483108	-7.008639	-0.368230
H	0.124824	-8.507350	0.779342
C	2.047732	-7.704689	2.483603
C	4.819166	4.869772	-1.551170
H	0.839396	8.128239	-0.380132
C	5.316388	7.596247	-1.251086
H	3.143248	9.027235	-0.560867
C	3.830359	5.212167	2.180773
H	7.965040	2.368073	0.421207
H	8.326461	4.586653	1.451087
C	6.423638	6.201947	2.442054
C	-4.651637	2.960320	-2.353726
H	-4.552618	-1.361245	-5.426881
C	-6.767144	2.433180	-4.086705

H	-6.499182	0.163481	-5.491907
C	-5.560344	-0.370577	-0.745939
H	-5.648364	3.951202	2.327273
H	-7.753774	3.049242	1.393541
C	-7.984823	0.855287	-0.133010
C	-5.650937	3.894929	-2.535797
C	-6.741242	3.617722	-3.381697
C	-6.751965	-0.968605	-1.103215
C	-7.978467	-0.334902	-0.828892
C	6.310040	6.767913	-1.727788
C	6.038412	5.399570	-1.921229
C	4.059531	6.391088	2.859174
C	5.364939	6.909655	2.968901
C	4.207578	-7.354203	-1.190837
C	3.202517	-6.481303	-1.652319
C	2.971880	-5.760666	3.561807
C	2.902682	-7.160001	3.418012
H	2.267138	-4.578673	-1.451097
H	5.870303	-7.581878	0.144867
H	2.304331	-3.863862	2.857815
H	1.924808	-8.781528	2.408690
H	4.617742	3.823006	-1.733299
H	5.482967	8.666426	-1.165815
H	2.823895	4.820936	2.122236
H	7.441562	6.563369	2.558592
H	-3.812228	3.205994	-1.719643
H	-7.568606	2.227569	-4.790792
H	-4.633091	-0.888201	-0.944173
H	-8.923268	1.328756	0.141776
H	-5.588346	4.851221	-2.026307
H	-7.535316	4.347453	-3.505511
H	-6.738737	-1.936979	-1.593193
H	-8.912190	-0.799241	-1.130454
H	7.280671	7.173705	-1.995427
H	6.788669	4.755062	-2.367554
H	3.227390	6.917373	3.315994
H	5.536828	7.845698	3.491123
H	4.326548	-8.334437	-1.641865
H	2.560253	-6.781037	-2.474377
H	3.583404	-5.325120	4.345272
H	3.484865	-7.803997	4.069743

MH 4 (MMMMMM)

156

C	-1.093686	2.160962	1.550723
C	-2.341715	1.554857	1.318895
C	-2.424259	0.120472	1.171953
C	-1.251770	-0.588907	0.855250
C	0.037059	0.019762	1.087929
C	0.102280	1.417857	1.228816
C	-1.405151	-1.892911	0.223865
C	-3.646240	-0.643877	1.406507
C	-3.675685	-2.041098	1.117782
C	-2.491568	-2.694481	0.582863
C	-3.495283	2.429876	1.157793
C	-3.552133	3.616658	1.892998
C	-2.298884	4.200275	2.345080
C	-1.077827	3.481946	2.173159
C	1.381354	2.061121	0.958865
C	2.560038	1.387023	1.287565
C	2.518696	-0.064763	1.362484
C	1.268932	-0.745639	1.258272
C	-2.379398	-4.128009	0.328083
C	-1.582022	-4.560397	-0.749937
C	-0.769542	-3.593787	-1.472868
C	-0.591290	-2.297962	-0.916765
C	1.479052	3.282762	0.168030
C	2.753073	3.878749	-0.036523
C	3.885769	3.393125	0.736963
C	3.765510	2.192181	1.463694
C	0.380109	3.795491	-0.559321
C	0.522727	4.856967	-1.427799
C	1.777602	5.461557	-1.632697
C	2.866312	4.954387	-0.941862
C	-4.867085	4.218585	2.092272
C	-5.858705	4.011652	1.112340
C	-5.605940	3.082103	0.022188
C	-4.485330	2.215392	0.109529
C	-6.469406	2.981011	-1.091282
C	-6.270995	2.055991	-2.101994
C	-5.146713	1.209821	-2.008398
C	-4.278863	1.291484	-0.942365
C	-4.790502	-0.070515	2.012488
C	-4.917899	-2.716977	1.205090
C	-2.229295	5.539559	2.802129
C	0.102695	4.048026	2.712854
C	3.705140	-0.839272	1.367317
C	-2.927520	-5.126820	1.226339
C	-1.563501	-5.944803	-1.093849
H	-0.590640	3.331049	-0.457957
H	-0.343122	5.220606	-1.974429
C	1.936814	6.576613	-2.636049
H	-7.305827	3.661863	-1.167040
C	-7.189527	1.927351	-3.299275
H	-4.950362	0.493483	-2.801844
H	-3.405585	0.654517	-0.930197
C	0.237519	-1.385069	-1.609159
C	0.877338	-1.736658	-2.778930
C	0.715247	-3.019576	-3.335760
C	-0.111036	-3.916298	-2.677797
C	1.266667	-2.151458	1.428267
H	3.838164	5.398131	-1.126073
H	0.353507	-0.378255	-1.233381
H	1.501762	-1.006780	-3.286973
H	-0.262307	-4.892157	-3.125194
C	-1.043041	6.101038	3.226211
C	0.125293	5.323424	3.238268
C	2.436806	-2.876065	1.522791
C	3.672319	-2.216703	1.429898
C	-5.963561	-0.779067	2.170796

C	-6.045892	-2.100509	1.705019
H	-4.746397	0.946839	2.372170
H	-4.981704	-3.741014	0.865803
H	-3.130216	6.136253	2.813180
H	1.012101	3.465687	2.725624
H	4.659632	-0.335207	1.315313
H	0.324985	-2.675649	1.497073
H	-1.023866	7.131413	3.566930
H	1.050754	5.722938	3.640367
H	2.393564	-3.952937	1.650285
H	4.599941	-2.780298	1.427916
H	-6.819944	-0.303614	2.638063
H	-6.983003	-2.645939	1.754819
C	-2.907308	-6.502040	0.839469
C	-2.250473	-6.872644	-0.366311
C	-3.381601	-4.826383	2.538501
H	-0.979419	-6.272551	-1.944421
C	-3.461444	-7.484844	1.697223
H	-2.239423	-7.919277	-0.656581
C	-5.241296	4.889426	3.323364
C	-6.489506	5.581019	3.391863
C	-7.386268	5.501762	2.290750
C	-7.104974	4.697382	1.225078
C	-4.482356	4.796599	4.520657
C	-6.855764	6.260846	4.580248
H	-8.333323	6.030665	2.347311
H	-7.854231	4.567646	0.454494
C	4.769940	1.886777	2.465094
C	5.989384	2.631072	2.482442
C	6.144396	3.727325	1.589760
C	5.108011	4.126426	0.796815
C	4.559299	0.957804	3.519056
C	6.988751	2.326374	3.440117
H	7.074093	4.288771	1.605983
H	5.213933	5.030191	0.210269
C	-8.374584	2.891355	-3.357901
C	-9.245178	2.681597	-4.599912
C	-10.433723	3.642655	-4.662507
C	2.041494	6.057140	-4.080628
C	2.189305	7.176486	-5.113783
C	2.293976	6.652897	-6.547568
H	2.831684	7.164361	-2.398946
H	1.083046	7.262374	-2.564084
H	-7.567594	0.895745	-3.334345
H	-6.589208	2.043767	-4.212682
H	-8.010117	3.927185	-3.344063
H	-8.993957	2.770512	-2.459159
H	-9.607828	1.645262	-4.616192
H	-8.626642	2.801163	-5.499316
H	-11.085469	3.520541	-3.790205
H	-11.040037	3.473792	-5.557819
H	-10.097085	4.685240	-4.679110
H	1.151712	5.456692	-4.311410
H	2.895947	5.371297	-4.150754
H	3.078390	7.774932	-4.873789
H	1.332887	7.859088	-5.031691
H	3.160753	5.992802	-6.663476
H	2.398575	7.470624	-7.267556
H	1.402982	6.077613	-6.822633
C	1.363000	-3.371675	-4.651760
C	0.572168	-2.835947	-5.858075
C	1.222458	-3.172558	-7.201969
C	0.430473	-2.637743	-8.396778
H	1.458814	-4.460575	-4.738777
H	2.381338	-2.963759	-4.682596
H	0.464870	-1.747477	-5.762100
H	-0.446349	-3.244511	-5.825221
H	1.330180	-4.262091	-7.288672
H	2.241797	-2.764304	-7.222258
H	-0.582291	-3.055321	-8.416671
H	0.914363	-2.890443	-9.345458
H	0.336004	-1.547159	-8.350110
C	-6.060008	6.206131	5.701790
C	-4.876772	5.437895	5.674810
C	6.776047	1.369692	4.406281
C	5.533314	0.703461	4.459988
C	-3.884063	-5.805104	3.368196
C	-3.956205	-7.146436	2.936039
H	-3.318547	-3.809494	2.900540
H	-3.457908	-8.519788	1.366493
H	-3.582084	4.197867	4.531697
H	-7.797839	6.801922	4.595881
H	3.608260	0.448851	3.593817
H	7.917714	2.889248	3.413620
H	-6.354805	6.720964	6.610872
H	-4.275433	5.339636	6.573243
H	7.543468	1.151475	5.142373
H	5.335385	-0.008072	5.255585
H	-4.215945	-5.540518	4.367336
H	-4.368237	-7.908260	3.590440

MH 4 (MMMMMP)

156

C	-1.048486	2.095460	1.325891
C	-2.294485	1.478139	1.093001
C	-2.364989	0.047260	0.889049
C	-1.166723	-0.639981	0.611361
C	0.093617	0.009807	0.818059
C	0.151268	1.412905	0.906805
C	-1.214171	-1.992544	0.083680
C	-3.574634	-0.754173	1.064309
C	-3.536437	-2.160200	0.810132
C	-2.290912	-2.810549	0.429802
C	-3.446869	2.370041	0.998335
C	-3.499672	3.506489	1.811097
C	-2.244529	4.057411	2.301981
C	-1.028503	3.360993	2.046117
C	1.382472	2.045117	0.430428
C	2.558221	1.284738	0.375194
C	2.555817	-0.014992	1.032746

C	1.323412	-0.705710	1.136410	H	-3.056934	5.950552	2.950967
C	-2.098447	-4.253090	0.340435	H	1.082115	3.305008	2.490121
C	-1.150290	-4.755584	-0.576772	H	4.639932	-0.027668	1.583231
C	-0.276640	-3.835977	-1.289600	H	0.367801	-2.508628	1.851139
C	-0.254943	-2.471355	-0.900823	H	-0.927426	6.884375	3.707974
C	1.401000	3.390009	-0.131061	H	1.154380	5.495316	3.583768
C	2.656427	4.032741	-0.322555	H	2.420597	-3.515381	2.732745
C	3.866144	3.227335	-0.355048	H	4.570822	-2.247448	2.646201
C	3.750057	1.826885	-0.244064	H	-6.847196	-0.510822	2.037374
C	0.235125	4.100555	-0.494413	H	-6.866731	-2.865053	1.166941
C	0.283900	5.427527	-0.862637	C	-2.619659	-6.587713	1.021271
C	1.509925	6.120838	-0.898790	C	-1.807993	-7.045282	-0.053447
C	2.670531	5.405787	-0.656299	C	6.070688	1.599270	-1.090688
C	-4.810530	4.108341	2.035819	C	6.225214	3.005745	-0.922482
C	-5.791174	3.985048	1.031348	C	-3.337009	-4.780518	2.480506
C	-5.536353	3.127047	-0.115104	H	-0.365935	-6.554386	-1.513647
C	-4.428759	2.240248	-0.071121	H	5.273087	4.867835	-0.603575
C	-6.386711	3.110189	-1.242857	C	4.617184	-0.354858	-1.166951
C	-6.190251	2.246744	-2.307176	C	-3.248302	-7.506342	1.898143
C	-5.082731	1.375408	-2.252237	H	-1.737130	-8.113179	-0.238924
C	-4.226630	1.376550	-1.173451	C	7.119978	0.802643	-1.611309
C	-4.783290	-0.217905	1.573143	H	7.198242	3.451667	-1.107625
C	-4.759477	-2.875144	0.800262	C	-5.191427	4.698439	3.304935
C	-2.159355	5.355551	2.862613	C	-6.430960	5.401471	3.406356
C	0.173498	3.884183	2.576743	C	-7.313696	5.412633	2.291085
C	3.711738	-0.584119	1.608242	C	-7.028915	4.680460	1.175380
C	-2.723479	-5.184151	1.264609	C	-4.448370	4.512111	4.501216
C	-1.055313	-6.166140	-0.775161	C	-6.803051	6.001742	4.635047
C	5.148088	3.792384	-0.630907	H	-8.253939	5.950371	2.372443
C	4.808409	1.001906	-0.794511	H	-7.768997	4.616264	0.387777
H	-0.720712	3.596841	-0.479339	C	2.439971	-5.356796	-4.367662
H	-0.634899	5.946442	-1.120934	C	3.461100	-5.716832	-5.450361
C	1.555372	7.574129	-1.301005	C	3.486659	-7.212503	-5.770308
H	-7.211527	3.807952	-1.284968	C	-8.259924	3.199747	-3.534548
C	-7.094947	2.209933	-3.521324	C	-9.117885	3.085902	-4.797729
H	-4.890098	0.704546	-3.085368	C	-10.285480	4.074111	-4.814370
H	-3.365665	0.722848	-1.186847	C	1.455198	7.769017	-2.823807
C	0.593193	-1.591322	-1.618372	C	1.482230	9.239979	-3.245449
C	1.430599	-2.046591	-2.611308	C	1.383637	9.426623	-4.760794
C	1.461008	-3.411386	-2.968885	H	2.485341	8.029177	-0.939678
C	0.591951	-4.269764	-2.318852	H	0.732696	8.115290	-0.816472
C	1.307238	-1.982801	1.742402	H	-7.492543	1.190301	-3.624610
H	3.618394	5.918312	-0.775553	H	-6.480281	2.370199	-4.418362
H	0.567023	-0.531303	-1.407004	H	3.430223	-3.540221	-3.770510
H	2.067210	-1.340795	-3.138363	H	2.189133	-3.305181	-4.973957
C	2.417345	-3.861052	-4.053433	H	1.442603	-5.682456	-4.691884
H	0.574313	-5.308754	-2.616109	H	2.666930	-5.922060	-3.453905
C	-0.957129	5.883976	3.287718	H	4.459140	-5.391641	-5.127823
C	0.214946	5.116990	3.193864	H	3.236624	-5.148324	-6.362578
C	2.462096	-2.541611	2.254973	H	3.739964	-7.801286	-4.881627
C	3.671794	-1.830058	2.203893	H	4.223596	-7.444775	-6.545326
C	-5.941868	-0.963394	1.645718	H	2.508881	-7.556188	-6.125817
C	-5.946046	-2.290723	1.189825	H	-7.875099	4.224918	-3.451628
H	-4.803798	0.801192	1.926559	H	-8.893768	3.036212	-2.652799
H	-4.756025	-3.907736	0.479887	H	-9.501433	2.060527	-4.883027

H	-8.485104	3.248002	-5.680394
H	-10.951030	3.912029	-3.959165
H	-10.883285	3.974178	-5.725605
H	-9.927200	5.108253	-4.761913
H	0.531980	7.298304	-3.186448
H	2.279982	7.228555	-3.306459
H	2.405918	9.705202	-2.875800
H	0.656775	9.771786	-2.753548
H	2.215028	8.930436	-5.273655
H	1.404775	10.485348	-5.037723
H	0.453716	8.997913	-5.150539
C	6.918032	-0.527011	-1.909006
C	5.642715	-1.097465	-1.712785
C	-6.022194	5.857659	5.759230
C	-4.848490	5.077189	5.692643
C	-3.909892	-5.695581	3.337273
C	-3.897804	-7.072625	3.031418
H	-3.341445	-3.733937	2.750687
H	3.644088	-0.810616	-1.044991
H	-3.177095	-8.566721	1.672295
H	8.080856	1.272292	-1.802929
H	-3.555975	3.901676	4.479722
H	-7.737902	6.553797	4.677657
H	7.724665	-1.125292	-2.321282
H	5.460639	-2.129158	-1.998575
H	-6.321797	6.311813	6.698585
H	-4.259789	4.908662	6.589009
H	-4.363482	-5.350824	4.261295
H	-4.366413	-7.784669	3.703561

MH 4 (MMMMPP)

156

C	-0.956691	2.139357	1.067712
C	-2.207680	1.608624	0.690150
C	-2.338230	0.171580	0.612031
C	-1.167255	-0.601669	0.466323
C	0.115070	-0.022488	0.759882
C	0.236643	1.377556	0.847944
C	-1.287288	-1.973585	-0.003232
C	-3.600807	-0.536288	0.783425
C	-3.655807	-1.949421	0.603167
C	-2.441913	-2.696784	0.306849
C	-3.216200	2.595956	0.294797
C	-3.134558	3.902083	0.796584
C	-2.155787	4.157707	1.846165
C	-0.987316	3.357073	1.865818
C	1.545783	1.973235	0.613088
C	2.688300	1.173844	0.732952
C	2.527090	-0.150000	1.322377
C	1.263867	-0.792978	1.226805
C	-2.357701	-4.152412	0.270006
C	-1.418374	-4.755334	-0.591978
C	-0.443280	-3.927997	-1.285599
C	-0.315729	-2.560926	-0.916775
C	1.693166	3.342604	0.139211
C	2.973214	3.957146	0.225785
C	4.148595	3.115017	0.378493
C	3.986673	1.713424	0.379331
C	0.635979	4.098019	-0.418609
C	0.783267	5.436884	-0.711262
C	2.000724	6.100559	-0.457487
C	3.077093	5.345083	-0.024569
C	-4.047755	4.923698	0.323657
C	-5.252256	4.512275	-0.285798
C	-5.328626	3.178336	-0.856177
C	-4.218276	2.303351	-0.718631
C	-6.406220	2.787360	-1.686305
C	-6.375443	1.626998	-2.439392
C	-5.197782	0.849628	-2.407045
C	-4.153264	1.180087	-1.574481
C	-4.777389	0.124765	1.210477
C	-4.930481	-2.567816	0.576859
C	-2.332411	5.130371	2.851713
C	0.017364	3.632926	2.819457
C	3.561108	-0.779673	2.049780
C	-3.089575	-5.002118	1.193232
C	-1.426988	-6.174059	-0.748113
C	5.469985	3.655940	0.387576
C	5.116700	0.890993	-0.006453
H	-0.309288	3.617925	-0.631238
H	-0.049206	5.987698	-1.139429
C	2.139060	7.572598	-0.757299
C	-3.773083	6.347370	0.401959
C	-6.310051	5.459925	-0.435212
H	-7.264479	3.439271	-1.771591
C	-7.523058	1.181491	-3.321121
H	-5.118077	-0.026074	-3.045405
H	-3.265987	0.562392	-1.571625
C	0.652675	-1.778755	-1.591347
C	1.488714	-2.324068	-2.541972
C	1.392281	-3.684672	-2.893867
C	0.420250	-4.451012	-2.272864
C	1.116687	-2.079435	1.797435
H	4.037451	5.837772	0.076833
H	0.723844	-0.720534	-1.383023
H	2.217480	-1.690470	-3.040574
C	2.272531	-4.259625	-3.975525
H	0.323600	-5.487614	-2.574154
C	-1.345881	5.372714	3.789369
C	-0.151020	4.635213	3.755767
C	2.161278	-2.696237	2.457134
C	3.390811	-2.034346	2.600516
C	-5.993287	-0.523057	1.268861
C	-6.080747	-1.870563	0.883235
H	-4.722885	1.165855	1.492398
H	-4.999144	-3.613260	0.310383
H	-3.255779	5.694775	2.881575
H	0.906851	3.016326	2.844736
H	4.499777	-0.258657	2.184210
H	0.152786	-2.568077	1.767066

H	-1.502555	6.124031	4.556881
H	0.625203	4.820058	4.491687
H	2.011162	-3.675301	2.901087
H	4.199037	-2.493868	3.160610
H	-6.877735	0.017648	1.589952
H	-7.042276	-2.373097	0.849503
C	-3.087962	-6.416496	0.993687
C	-2.274450	-6.969332	-0.033847
C	-6.133427	6.778940	-0.132897
C	-4.843503	7.273733	0.215013
C	6.423613	1.465409	-0.014781
C	6.568568	2.855295	0.262859
C	-3.715090	-4.513323	2.371193
H	-0.739933	-6.636385	-1.445023
H	5.606610	4.724179	0.502212
C	4.977100	-0.437966	-0.486003
C	-2.463620	6.878333	0.538973
H	-7.277881	5.125395	-0.787858
C	-3.820525	-7.254969	1.870332
H	-2.280674	-8.044811	-0.185828
H	-6.955948	7.481664	-0.230239
C	-4.594348	8.665286	0.305694
C	7.539913	0.666349	-0.363613
H	7.567101	3.281526	0.296194
C	1.762079	-3.928944	-5.388863
C	2.654501	-4.491752	-6.497513
C	2.140714	-4.161246	-7.900209
C	-8.735072	2.110742	-3.386888
C	-9.842094	1.573557	-4.298179
C	-11.058399	2.498855	-4.365806
C	2.289220	7.862906	-2.260299
C	2.395079	9.356847	-2.575107
C	2.543816	9.640791	-4.071061
H	3.004205	7.980340	-0.221195
H	1.256633	8.106188	-0.379876
H	-7.852894	0.190145	-2.978841
H	-7.139314	1.020761	-4.338321
H	2.337657	-5.348251	-3.861354
H	3.293071	-3.871076	-3.864996
H	1.682740	-2.839211	-5.496364
H	0.741765	-4.319076	-5.498607
H	2.733070	-5.580959	-6.380444
H	3.672860	-4.099072	-6.375430
H	1.136499	-4.569902	-8.058237
H	2.794576	-4.573429	-8.675153
H	2.083139	-3.077897	-8.053239
H	-8.422709	3.101250	-3.743682
H	-9.140986	2.260646	-2.377540
H	-10.152578	0.581750	-3.943584
H	-9.437511	1.422233	-5.307715
H	-11.501176	2.640817	-3.373620
H	-11.834797	2.094310	-5.022516
H	-10.780479	3.487688	-4.747295
H	1.433101	7.433785	-2.796983
H	3.177079	7.338620	-2.637190
H	3.249976	9.781587	-2.031933
H	1.504265	9.870233	-2.188923
H	3.443703	9.163850	-4.474970
H	2.616668	10.714425	-4.271034
H	1.685972	9.253924	-4.632100
C	7.375576	-0.639893	-0.768197
C	6.076216	-1.182057	-0.858847
C	-2.240923	8.238523	0.580365
C	-3.317470	9.145766	0.495271
C	-4.390733	-5.353354	3.229820
C	-4.475957	-6.736178	2.963744
H	-3.646730	-3.461293	2.610229
H	3.989078	-0.868813	-0.580973
H	-1.619086	6.206771	0.592295
H	-3.825303	-8.324289	1.677943
H	-5.430361	9.349221	0.187864
H	8.528338	1.116657	-0.336237
H	8.236163	-1.240337	-1.046318
H	5.936388	-2.191761	-1.232391
H	-1.224850	8.610002	0.675204
H	-3.135373	10.214660	0.548331
H	-4.850387	-4.945530	4.124698
H	-5.024567	-7.387979	3.636482

MH 4 (MMMPPP)

156

C	-0.927984	2.329502	0.912335
C	-2.198226	1.797767	0.605198
C	-2.318083	0.375291	0.455174
C	-1.170639	-0.393627	0.167449
C	0.114303	0.164751	0.479694
C	0.250070	1.561653	0.624543
C	-1.368515	-1.671356	-0.509319
C	-3.509873	-0.372135	0.834310
C	-3.598577	-1.744993	0.489433
C	-2.622395	-2.291425	-0.445548
C	-3.286166	2.743153	0.375597
C	-3.181390	4.042536	0.886976
C	-2.097884	4.313223	1.824249
C	-0.920125	3.527452	1.741445
C	1.569130	2.138486	0.384639
C	2.699758	1.317478	0.472924
C	2.522342	-0.017836	1.030662
C	1.251299	-0.636086	0.913815
C	-2.876686	-3.488586	-1.221344
C	-1.771716	-4.259443	-1.641625
C	-0.464990	-3.627883	-1.725252
C	-0.334426	-2.265523	-1.343340
C	1.735556	3.516273	-0.056009
C	3.029180	4.103741	0.017951
C	4.191152	3.235989	0.122257
C	4.001907	1.837868	0.106123
C	0.678227	4.310191	-0.557021
C	0.841001	5.656468	-0.802497
C	2.078935	6.287402	-0.563938

C	3.152466	5.497046	-0.190071
C	-4.184111	5.033804	0.552810
C	-5.453080	4.583844	0.129565
C	-5.578038	3.241134	-0.413484
C	-4.426045	2.410941	-0.465971
C	-6.764855	2.802002	-1.048857
C	-6.812444	1.646110	-1.807923
C	-5.609999	0.930452	-1.995790
C	-4.455347	1.302868	-1.345364
C	-4.469659	0.173702	1.716854
C	-4.586560	-2.539993	1.110097
C	-2.199277	5.281658	2.846523
C	0.147864	3.802212	2.625591
C	3.544665	-0.691561	1.733728
C	5.522684	3.751376	0.103612
C	5.106856	0.999018	-0.317178
H	-0.281475	3.853981	-0.756177
H	0.005515	6.238034	-1.182708
C	2.239665	7.765936	-0.817016
C	-3.937232	6.462908	0.588136
C	-6.544007	5.505623	0.124280
H	-7.654952	3.411176	-0.974120
C	-8.073923	1.143194	-2.477451
H	-5.595997	0.074211	-2.665129
H	-3.550279	0.737608	-1.519789
C	0.802021	-1.560467	-1.805250
C	1.834761	-2.203133	-2.449793
C	1.795562	-3.596461	-2.674426
C	0.636456	-4.273163	-2.338051
C	1.077635	-1.942362	1.425151
H	4.124141	5.968933	-0.098219
H	0.861089	-0.490897	-1.658013
H	2.692344	-1.629984	-2.792428
C	2.995580	-4.267905	-3.308179
H	0.554900	-5.321226	-2.590922
C	-1.145813	5.528411	3.705362
C	0.045701	4.795492	3.579372
C	2.111952	-2.603569	2.057280
C	3.350882	-1.965335	2.231887
C	-5.462878	-0.613508	2.264982
C	-5.510165	-1.987156	1.975847
H	-4.393968	1.214494	2.002445
H	-4.612322	-3.602086	0.903576
H	-3.123416	5.834086	2.956969
H	1.039867	3.191332	2.583294
H	4.493393	-0.193592	1.886234
H	0.104300	-2.410298	1.360697
H	-1.248851	6.274744	4.486715
H	0.873179	4.976869	4.258054
H	1.949381	-3.599871	2.456291
H	4.150274	-2.461328	2.773302
H	-6.178568	-0.174935	2.953243
H	-6.257517	-2.619453	2.444716
C	-6.358495	6.831980	0.387549
C	-5.045715	7.361460	0.549520

C	6.423331	1.549673	-0.355456
C	6.602077	2.932632	-0.063456
H	5.683269	4.815112	0.228330
C	4.931179	-0.321625	-0.808130
C	-2.633701	7.024019	0.544996
H	-7.543221	5.147049	-0.089847
H	-7.203661	7.514188	0.399555
C	-4.820145	8.758995	0.601761
C	7.514394	0.735989	-0.748342
H	7.608884	3.340155	-0.053372
C	-4.204195	-3.911747	-1.624877
C	-1.996798	-5.594128	-2.097379
C	-3.258376	-6.088713	-2.261267
C	-4.393906	-5.242277	-2.105831
C	-5.315550	-3.029473	-1.673920
H	-1.149501	-6.232478	-2.315264
H	-3.409661	-7.113390	-2.588385
C	-5.688444	-5.674836	-2.484999
C	2.853575	-5.753841	-3.637239
C	4.117367	-6.341063	-4.271667
C	3.981715	-7.828783	-4.600814
C	-9.318773	2.019049	-2.336915
C	-10.540342	1.426089	-3.044502
C	-11.789931	2.297318	-2.904701
C	2.354811	8.104397	-2.313075
C	2.486763	9.605424	-2.580903
C	2.601216	9.936367	-4.070206
H	3.128023	8.137074	-0.292403
H	1.380315	8.304300	-0.395655
H	-8.297193	0.142689	-2.078758
H	-7.863595	0.986275	-3.544545
H	3.856562	-4.133869	-2.636912
H	3.258150	-3.720575	-4.224166
H	2.004867	-5.901457	-4.318289
H	2.616606	-6.313882	-2.722903
H	4.966468	-6.189855	-3.591880
H	4.356505	-5.781503	-5.185689
H	3.774164	-8.414666	-3.698456
H	4.896322	-8.225099	-5.052596
H	3.159529	-8.003373	-5.303700
H	-9.114759	3.018362	-2.743964
H	-9.552768	2.160854	-1.273449
H	-10.741632	0.425231	-2.639890
H	-10.307835	1.282772	-4.108046
H	-12.062413	2.429384	-1.851658
H	-12.648451	1.853398	-3.418078
H	-11.625209	3.293513	-3.330123
H	1.474782	7.712685	-2.839678
H	3.219604	7.573735	-2.732248
H	3.365858	9.992225	-2.048146
H	1.619206	10.125554	-2.152947
H	3.478313	9.453189	-4.514746
H	2.693775	11.014227	-4.236413
H	1.719622	9.588105	-4.619610
C	-2.440663	8.389052	0.555057

C	-3.541055	9.269780	0.614986
C	7.315618	-0.561112	-1.166779
C	6.004822	-1.079608	-1.224380
C	-6.557149	-3.463924	-2.086648
C	-6.758140	-4.807300	-2.467630
H	3.935258	-0.737319	-0.874204
H	-1.774167	6.370823	0.486377
H	-5.680309	9.422799	0.598505
H	8.511066	1.168582	-0.743622
H	-5.184772	-1.991874	-1.399292
H	-5.813799	-6.700117	-2.822068
H	-1.430900	8.786161	0.510175
H	-3.379048	10.342886	0.641016
H	8.157025	-1.171702	-1.479351
H	5.835082	-2.081916	-1.606279
H	-7.383808	-2.760726	-2.124450
H	-7.743395	-5.145033	-2.773734

MH 4 (MMPMMM)

156

C	-0.845113	1.935870	1.189889
C	-2.150011	1.425909	1.043237
C	-2.304279	-0.001383	0.857971
C	-1.141312	-0.745173	0.547795
C	-0.009317	-0.029093	0.010169
C	0.184331	1.310164	0.388628
C	-1.118285	-2.174001	0.769579
C	-3.549364	-0.738949	1.021793
C	-3.561480	-2.137039	0.721081
C	-2.321001	-2.889542	0.822947
C	-3.217785	2.414457	1.135311
C	-3.100929	3.458440	2.060332
C	-1.768933	3.820101	2.528725
C	-0.646491	3.065398	2.074778
C	1.370713	2.011311	-0.080775
C	2.473922	1.240262	-0.477628
C	2.183910	-0.031412	-1.117439
C	0.898463	-0.613033	-0.956834
C	-2.272943	-4.342494	0.941552
C	-1.085266	-5.000350	0.549802
C	0.170909	-4.270480	0.611554
C	0.136616	-2.890508	0.931267
C	1.326071	-2.273543	1.371893
C	2.527972	-2.947567	1.351062
C	2.605081	-4.270256	0.868462
C	1.425608	-4.917465	0.538779
C	1.453915	3.447556	-0.297009
C	2.722926	4.051007	-0.507565
C	3.921015	3.232432	-0.393604
C	3.800185	1.827766	-0.367792
C	0.298117	4.243807	-0.468348
C	0.387767	5.586606	-0.764339
C	1.640989	6.210782	-0.920146
C	2.779082	5.429240	-0.805966
C	-4.326665	4.170463	2.413284
C	-5.352339	4.267615	1.451106
C	-5.256490	3.497217	0.220700
C	-4.262673	2.489231	0.125487
C	-6.155973	3.674795	-0.853931
C	-6.116517	2.885582	-1.991306
C	-5.125206	1.884634	-2.067640
C	-4.222035	1.696567	-1.045299
C	-4.785046	-0.147421	1.380514
C	-4.798243	-2.732372	0.379188
C	-1.507582	5.007528	3.255475
C	0.641789	3.396381	2.554560
C	3.068171	-0.594001	-2.070435
C	0.537046	-1.713501	-1.764670
H	1.292878	-1.249157	1.721016
H	3.426973	-2.452765	1.705249
C	3.941826	-4.965722	0.786689
H	1.475872	-5.964595	0.261916
C	5.216237	3.823880	-0.320860
C	4.975571	1.025454	-0.082490
H	-0.678453	3.785881	-0.394046
H	-0.521006	6.168212	-0.892305
C	1.727695	7.670916	-1.289493
H	3.739926	5.899511	-0.982612
H	-6.893020	4.463637	-0.793764
C	-7.075038	3.058354	-3.151372
H	-5.062114	1.264003	-2.957595
H	-3.451122	0.945113	-1.151270
C	1.425302	-2.258485	-2.668323
C	2.704737	-1.694589	-2.818715
C	-5.992925	-2.068686	0.571151
C	-5.984446	-0.797719	1.169790
C	0.852344	4.510043	3.339836
C	-0.228797	5.352677	3.645737
C	6.257834	1.654500	-0.041087
C	6.344281	3.065556	-0.196674
H	-4.796812	0.845537	1.801038
H	-4.802861	-3.742328	-0.007280
H	-2.326364	5.667030	3.502972
H	1.476308	2.758885	2.293201
H	4.030731	-0.127785	-2.234866
H	-0.459064	-2.127333	-1.672328
H	5.309077	4.902237	-0.350916
C	4.915477	-0.350876	0.263314
H	1.126346	-3.105595	-3.277363
H	3.397013	-2.100703	-3.549665
H	-6.929641	-2.547653	0.304731
H	-6.915299	-0.303968	1.429555
H	1.851056	4.743077	3.694594
H	-0.067120	6.271852	4.200071
C	7.415714	0.880875	0.224420
H	7.322175	3.536797	-0.156758
C	-1.129062	-6.395940	0.256352
C	-3.341699	-5.165920	1.474700
C	-3.353439	-6.565727	1.188234
C	-2.251194	-7.137456	0.491081

H	-0.256012	-6.873232	-0.172301
C	-4.317195	-4.675330	2.381574
C	-4.411652	-7.373743	1.672505
H	-2.281566	-8.192041	0.232927
C	-4.589211	4.660609	3.752010
C	-6.486474	5.090314	1.722411
C	-6.638361	5.730525	2.917964
C	-5.727568	5.491941	3.984092
C	-3.836006	4.254276	4.885775
H	-7.254979	5.200571	0.967735
H	-7.499700	6.367640	3.096811
C	-5.980309	5.993587	5.285208
C	4.559226	-5.263842	2.162713
C	5.940812	-5.915856	2.069894
C	6.555349	-6.207360	3.440193
C	-8.112895	4.173055	-3.019612
C	-9.035957	4.267750	-4.237678
C	-10.076231	5.382050	-4.110174
C	1.492111	7.915259	-2.790176
C	1.561570	9.394349	-3.177136
C	1.327852	9.630582	-4.670598
H	4.637582	-4.337761	0.213138
H	3.836998	-5.901477	0.225243
H	2.711846	8.066034	-1.010480
H	0.986783	8.240790	-0.714301
H	-7.595772	2.104017	-3.314251
H	-6.487904	3.224038	-4.065695
H	3.877471	-5.912706	2.727538
H	4.634432	-4.331460	2.737355
H	6.610621	-5.260020	1.496992
H	5.863991	-6.847547	1.493631
H	6.672351	-5.286963	4.023016
H	7.542194	-6.671607	3.347678
H	5.920055	-6.885689	4.020315
H	-7.604975	5.136149	-2.876861
H	-8.720056	4.008424	-2.119455
H	-9.543138	3.304661	-4.382705
H	-8.429820	4.430609	-5.138695
H	-10.716286	5.225363	-3.234735
H	-10.723068	5.430331	-4.991623
H	-9.594562	6.359513	-3.995681
H	0.513079	7.505662	-3.071482
H	2.234578	7.345433	-3.364043
H	2.541516	9.797892	-2.888800
H	0.818980	9.955393	-2.594035
H	2.076446	9.105546	-5.274224
H	1.381992	10.694320	-4.922787
H	0.341922	9.264244	-4.977505
C	6.053372	-1.076435	0.541829
C	7.324005	-0.464496	0.500073
C	-5.308468	-5.493091	2.880216
C	-5.383301	-6.848260	2.494648
C	-4.122907	4.727651	6.147659
C	-5.186943	5.632243	6.350324
H	3.956841	-0.843447	0.315912
H	8.380071	1.381398	0.229215
H	-4.266093	-3.644559	2.704783
H	-4.417452	-8.428872	1.413360
H	-3.027911	3.546947	4.756607
H	-6.836669	6.646502	5.429643
H	5.966217	-2.126711	0.805489
H	8.216965	-1.045146	0.709528
H	-6.031158	-5.090162	3.582884
H	-6.180472	-7.479204	2.875255
H	-3.531223	4.390857	6.993175
H	-5.396340	6.012373	7.345341

MH 4 (MMPMMP)

156

C	-0.957768	2.277931	1.448459
C	-2.272065	1.793907	1.278744
C	-2.429633	0.357880	1.257681
C	-1.288196	-0.434876	0.986972
C	-0.117161	0.222530	0.453769
C	0.098960	1.574733	0.779978
C	-1.331379	-1.859328	1.250022
C	-3.684203	-0.322738	1.530707
C	-3.776665	-1.721214	1.262733
C	-2.563714	-2.524865	1.325085
C	-3.287664	2.833771	1.125005
C	-3.081982	4.086677	1.719695
C	-1.934980	4.220552	2.612507
C	-0.806550	3.400667	2.353290
C	1.344459	2.219328	0.400699
C	2.441108	1.400888	0.092535
C	2.137478	0.128837	-0.545543
C	0.827755	-0.413639	-0.441037
C	-2.570198	-3.978917	1.447967
C	-1.411995	-4.682534	1.046157
C	-0.128181	-4.005961	1.122208
C	-0.111068	-2.625963	1.435648
C	1.093586	-2.061817	1.906245
C	2.260362	-2.793209	1.924274
C	2.294943	-4.119244	1.441310
C	1.097312	-4.711422	1.077434
C	1.496026	3.650898	0.196693
C	2.799744	4.207086	0.095211
C	3.953100	3.339154	0.281433
C	3.778077	1.938140	0.288509
C	0.389417	4.487485	-0.084691
C	0.552719	5.826699	-0.366178
C	1.835578	6.409391	-0.387362
C	2.930098	5.586853	-0.178995
C	-4.023973	5.163291	1.483707
C	-5.314470	4.834084	1.014376
C	-5.512392	3.565489	0.333734
C	-4.426653	2.653467	0.242872
C	-6.696891	3.273523	-0.379852
C	-6.800573	2.180579	-1.224634
C	-5.665088	1.365138	-1.406102

C	-4.510693	1.597882	-0.691605
C	-4.855584	0.340968	1.965350
C	-5.062248	-2.251718	1.001931
C	-1.897302	5.093432	3.718097
C	0.361299	3.563366	3.129013
C	3.050743	-0.479253	-1.441062
C	0.477580	-1.524039	-1.240086
H	1.096344	-1.036990	2.256753
H	3.169543	-2.346070	2.315162
H	1.091925	-5.758144	0.804582
C	5.263696	3.880055	0.433923
C	4.905928	1.092191	0.637010
H	-0.604475	4.062895	-0.121104
H	-0.317950	6.437279	-0.588311
C	1.999771	7.872057	-0.718345
H	3.918086	6.025403	-0.260780
C	-3.700264	6.568396	1.655032
C	-6.354665	5.807063	1.110823
H	-7.545034	3.945334	-0.309996
C	-8.060471	1.918963	-2.012235
H	-5.707272	0.535535	-2.106143
H	-3.657286	0.948388	-0.832347
C	1.394808	-2.114423	-2.084113
C	2.694857	-1.587282	-2.182388
C	-6.205806	-1.522894	1.264847
C	-6.097512	-0.249829	1.850308
C	0.391182	4.467074	4.174463
C	-0.753803	5.218999	4.486597
C	-4.759866	7.523847	1.718591
C	-6.100009	7.085543	1.515081
C	6.206619	1.671983	0.752219
C	6.354037	3.079621	0.613472
H	-4.778788	1.346931	2.351179
H	-5.147541	-3.262391	0.627571
H	-2.777726	5.674916	3.961194
H	1.219752	2.933755	2.927909
H	4.033628	-0.043965	-1.565753
H	-0.533615	-1.907450	-1.188001
H	5.399522	4.953967	0.417172
C	4.776318	-0.281257	0.973714
C	-2.370448	7.065292	1.640146
H	-7.371316	5.522253	0.868899
H	1.104048	-2.968283	-2.687655
H	3.411343	-2.028002	-2.868623
H	-7.180776	-1.955544	1.064274
H	-6.984748	0.293341	2.158656
H	1.289105	4.565801	4.776257
H	-0.746736	5.894169	5.336403
C	-4.460209	8.895770	1.905242
H	-6.907589	7.805914	1.608105
C	7.319324	0.854566	1.073060
H	7.345027	3.513627	0.710782
C	-1.514278	-6.071960	0.737609
C	-3.659028	-4.766599	1.995516
C	-3.731129	-6.161897	1.695312
C	-2.663560	-6.769820	0.974518
H	-0.664701	-6.577988	0.294485
C	-4.590335	-4.250997	2.934129
C	-4.809839	-6.933197	2.193770
H	-2.740646	-7.818884	0.703979
C	-7.975590	2.443261	-3.456052
C	-9.241018	2.167408	-4.271432
C	-9.151415	2.692750	-5.705577
C	1.866231	8.158748	-2.223820
C	2.003179	9.643959	-2.566677
C	1.869719	9.923677	-4.064895
H	2.977383	8.224349	-0.368415
H	1.243872	8.456585	-0.177380
H	-8.915396	2.386056	-1.508836
H	-8.261344	0.840153	-2.035126
H	-7.109352	1.987726	-3.953557
H	-7.776977	3.522700	-3.431228
H	-10.103377	2.621809	-3.765343
H	-9.432789	1.086089	-4.285714
H	-8.991156	3.776506	-5.718662
H	-10.066786	2.483824	-6.268107
H	-8.316171	2.230083	-6.242901
H	0.894841	7.787551	-2.575648
H	2.625971	7.579880	-2.765042
H	2.974570	10.010060	-2.207952
H	1.241984	10.212701	-2.015906
H	2.638737	9.390867	-4.635095
H	1.970484	10.991110	-4.285002
H	0.894418	9.595611	-4.441070
C	3.628687	-4.832927	1.378639
C	3.600688	-6.299283	0.948621
C	4.995957	-6.928638	0.908231
C	4.974954	-8.395385	0.474455
H	4.113995	-4.756108	2.361887
H	4.278747	-4.273623	0.689950
H	3.138868	-6.383401	-0.044070
H	2.964195	-6.873586	1.634847
H	5.459325	-6.844201	1.900203
H	5.632876	-6.351079	0.225078
H	4.370234	-9.000143	1.159348
H	5.982352	-8.822466	0.453391
H	4.545528	-8.503623	-0.527770
C	7.163286	-0.487879	1.333533
C	5.870054	-1.050878	1.306094
C	-5.600964	-5.035501	3.447546
C	-5.740197	-6.380676	3.045674
C	-2.105349	8.411652	1.776734
C	-3.156110	9.337265	1.945237
H	3.798588	-0.737288	0.976540
H	-1.546599	6.381627	1.495450
H	-5.284513	9.599648	1.980325
H	8.299786	1.318924	1.132655
H	-4.488689	-3.228032	3.269869
H	-4.863183	-7.984049	1.922887
H	8.021721	-1.102923	1.584911

H 5.729072 -2.098035 1.557844
H -6.288224 -4.614288 4.174636
H -6.552564 -6.984700 3.437670
H -1.076461 8.757728 1.747692
H -2.937699 10.392999 2.072685

MH 4 (MMPMPM)
156
C -0.790286 2.491244 1.225791
C -2.070536 1.903799 1.186341
C -2.164597 0.468505 1.293405
C -0.999654 -0.290722 1.074994
C 0.141497 0.352704 0.478948
C 0.291843 1.746518 0.617282
C -0.997801 -1.710640 1.367468
C -3.340526 -0.227252 1.807957
C -3.402663 -1.647859 1.684326
C -2.214686 -2.394708 1.292006
C -3.214772 2.799181 1.126822
C -3.148902 4.030911 1.787205
C -1.834713 4.577163 2.099646
C -0.667649 3.797425 1.846747
C 1.514915 2.362662 0.117908
C 2.650219 1.548509 -0.005419
C 2.425653 0.176748 -0.426207
C 1.124713 -0.376242 -0.302379
C -2.175869 -3.815493 0.985433
C -1.007007 -4.544461 1.299398
C 0.156160 -3.851658 1.833936
C 0.181411 -2.434955 1.799191
C 1.310214 -1.767987 2.332259
C 2.388045 -2.468468 2.823095
C 2.399888 -3.880621 2.822359
C 1.279864 -4.540824 2.347141
C 1.626665 3.724488 -0.377812
C 2.913339 4.294083 -0.577807
C 4.089589 3.537587 -0.173826
C 3.958027 2.166318 0.125061
C 0.495684 4.451590 -0.813239
C 0.619542 5.708288 -1.364863
C 1.884991 6.307135 -1.523150
C 3.003893 5.584161 -1.141972
C -4.412451 4.700255 2.079869
C -5.511577 4.474294 1.225426
C -5.437629 3.425858 0.219702
C -4.346690 2.518757 0.257530
C -6.449876 3.240017 -0.748798
C -6.428018 2.192271 -1.653260
C -5.335594 1.300851 -1.604762
C -4.323574 1.463899 -0.685472
C -4.389084 0.431669 2.488468
C -4.534342 -2.325607 2.199672
C -1.649744 5.922901 2.503121
C 0.584646 4.296249 2.277021
C 3.399662 -0.531691 -1.171326
C 0.829771 -1.585395 -0.968990
H 1.312933 -0.686982 2.379336
H 3.235230 -1.926941 3.236810
H 1.259742 -5.621103 2.386090
C 5.373983 4.149566 -0.085146
C 5.085933 1.451873 0.691612
H -0.485056 4.003123 -0.731457
H -0.269666 6.239955 -1.692196
C 2.013056 7.663138 -2.171852
H 3.979560 6.025182 -1.313695
H -7.267570 3.945620 -0.793415
C -7.508687 1.973000 -2.691627
H -5.281555 0.484638 -2.320633
H -3.479406 0.788630 -0.707086
C 1.796744 -2.261321 -1.685211
C 3.096708 -1.734517 -1.776257
C -5.567179 -1.644858 2.811203
C -5.488330 -0.252816 2.967496
C 0.720552 5.576756 2.769310
C -0.401592 6.420442 2.818175
C 6.361321 2.094454 0.740241
C 6.475001 3.446198 0.311121
H -4.320647 1.494923 2.665750
H -4.564155 -3.405679 2.153644
H -2.508395 6.575892 2.565762
H 1.453062 3.654829 2.212639
H 4.382346 -0.097437 -1.301071
H -0.176909 -1.981057 -0.925009
H 5.478961 5.200444 -0.324643
C 4.975296 0.172328 1.300596
H 1.544764 -3.187448 -2.191768
H 3.855598 -2.251975 -2.354873
H -6.414800 -2.194823 3.208080
H -6.272827 0.287314 3.487797
H 1.694369 5.934063 3.087995
H -0.296696 7.456838 3.123222
C 7.477928 1.406222 1.277311
H 7.447246 3.928344 0.358505
C -4.631318 5.472534 3.289355
C -6.695510 5.253992 1.394664
C -6.821383 6.156483 2.409968
C -5.822880 6.249309 3.418895
C -3.776140 5.399463 4.421120
H -7.521595 5.122235 0.707667
H -7.722244 6.755547 2.506777
C -6.035482 7.027054 4.584257
C -3.235337 -4.475482 0.244992
C -3.187586 -5.892466 0.069510
C -2.054086 -6.611552 0.540127
C -0.988548 -5.954397 1.082686
C -4.277914 -3.772197 -0.415559
C -4.226076 -6.552794 -0.632790
H -2.023755 -7.688446 0.401343
H -0.103728 -6.520216 1.345205
C -8.655112 2.984191 -2.708117

C	-9.697858	2.677604	-3.786728
C	-10.847556	3.686518	-3.805900
C	1.930435	7.592714	-3.706603
C	2.042622	8.962893	-4.379157
C	1.961419	8.885311	-5.905003
H	2.965407	8.123883	-1.883264
H	1.219804	8.325061	-1.801490
H	-7.924634	0.965414	-2.549326
H	-7.037696	1.951221	-3.684565
H	-8.255221	3.993956	-2.870460
H	-9.146694	3.001346	-1.726286
H	-10.096787	1.667078	-3.626996
H	-9.207296	2.658924	-4.768961
H	-11.374676	3.701429	-2.845434
H	-11.579011	3.447076	-4.583922
H	-10.478662	4.700703	-3.995343
H	0.983534	7.116944	-3.993731
H	2.725243	6.931235	-4.075389
H	2.989586	9.434303	-4.083650
H	1.246518	9.617608	-3.999960
H	2.765609	8.261684	-6.310930
H	2.043718	9.876047	-6.362961
H	1.010460	8.446481	-6.226786
C	3.621192	-4.599165	3.356918
C	3.569522	-6.126864	3.360062
C	4.851548	-6.759382	3.908592
C	4.805594	-8.288413	3.913449
H	3.814049	-4.245934	4.379793
H	4.493674	-4.274569	2.771501
H	3.395063	-6.492414	2.339372
H	2.714679	-6.466535	3.959828
H	5.027841	-6.392637	4.928495
H	5.707156	-6.418191	3.310741
H	3.977787	-8.655734	4.530154
H	5.731695	-8.716737	4.309127
H	4.662049	-8.681465	2.900789
C	-5.146859	6.986421	5.634609
C	-4.023743	6.135700	5.559465
C	6.072842	-0.465906	1.836445
C	7.345597	0.142397	1.806146
C	-5.262005	-4.435499	-1.116548
C	-5.256334	-5.843252	-1.206828
H	4.011886	-0.314490	1.354538
H	8.440538	1.910092	1.283520
H	-2.919964	4.739511	4.395226
H	-6.936178	7.631734	4.644321
H	-4.297081	-2.691841	-0.378324
H	-4.176498	-7.633695	-0.731166
H	-5.324997	7.575923	6.528566
H	-3.353171	6.049141	6.408723
H	5.950452	-1.443324	2.293856
H	8.206377	-0.372710	2.221048
H	-6.041657	-3.865386	-1.612256
H	-6.042722	-6.357834	-1.750128

MH 4 (MMPMPP)

156

C	-0.779744	2.452319	0.772859
C	-2.066951	1.887934	0.637696
C	-2.140970	0.450377	0.648647
C	-0.981103	-0.290505	0.338857
C	0.171957	0.409861	-0.163169
C	0.322279	1.776512	0.149862
C	-1.006558	-1.737269	0.457981
C	-3.301273	-0.280006	1.139636
C	-3.398518	-1.673372	0.864851
C	-2.241235	-2.385128	0.339087
C	-3.176898	2.827454	0.526855
C	-3.026266	4.122809	1.040454
C	-1.857441	4.381635	1.874367
C	-0.689929	3.609939	1.644681
C	1.575135	2.444063	-0.165710
C	2.708133	1.642900	-0.365816
C	2.485807	0.356312	-1.007556
C	1.183127	-0.209065	-0.999415
C	-2.247095	-3.756626	-0.140786
C	-1.086155	-4.541817	0.036158
C	0.103493	-3.946673	0.626659
C	0.159470	-2.537961	0.777018
C	1.305100	-1.971630	1.385618
C	2.371489	-2.752685	1.767647
C	2.356682	-4.151150	1.572108
C	1.218966	-4.719682	1.025806
C	1.719050	3.872571	-0.382250
C	3.015603	4.450575	-0.394834
C	4.165150	3.609483	-0.095535
C	4.011268	2.206305	-0.066421
C	0.621162	4.687893	-0.749228
C	0.784611	6.024002	-1.037144
C	2.058031	6.630683	-0.981668
C	3.146318	5.829116	-0.683196
C	-4.057127	5.114725	0.813245
C	-5.356646	4.668857	0.488852
C	-5.530441	3.328771	-0.047007
C	-4.392733	2.491391	-0.192985
C	-6.768975	2.890221	-0.573708
C	-6.890532	1.717927	-1.298961
C	-5.717812	0.976039	-1.559467
C	-4.507318	1.354827	-1.026239
C	-4.302824	0.329437	1.925996
C	-4.527302	-2.379785	1.346879
C	-1.854464	5.319702	2.928299
C	0.464826	3.859478	2.419089
C	3.477998	-0.235893	-1.825469
C	0.911319	-1.317930	-1.829521
H	1.328461	-0.907841	1.581756
H	3.230123	-2.288975	2.246881
C	3.569307	-4.957511	1.987901
H	1.176082	-5.794880	0.920103
C	5.451308	4.172812	0.154272

C	5.110435	1.383392	0.405062
H	-0.361067	4.244701	-0.843436
H	-0.077882	6.617232	-1.329888
C	2.174096	8.111304	-1.277850
H	4.134172	6.268527	-0.697915
C	-3.809800	6.544157	0.854169
C	-6.444649	5.588971	0.586410
H	-7.644605	3.509893	-0.439161
C	-8.208492	1.221488	-1.855458
H	-5.773993	0.093211	-2.191284
H	-3.625319	0.769399	-1.245778
C	1.898325	-1.884303	-2.610467
C	3.195193	-1.342203	-2.601042
C	-5.519508	-1.744451	2.066198
C	-5.402060	-0.378310	2.368435
C	0.455754	4.816434	3.415021
C	-0.717948	5.540702	3.683200
C	-4.918836	7.440383	0.922453
C	-6.239800	6.910894	0.857011
C	6.388097	1.985677	0.619128
C	6.530087	3.391445	0.451498
H	-4.197892	1.370906	2.196730
H	-4.587898	-3.447463	1.184047
H	-2.761710	5.868250	3.147246
H	1.349224	3.256071	2.257488
H	4.461878	0.213206	-1.867734
H	-0.094100	-1.718815	-1.859569
H	5.576700	5.247709	0.119256
C	4.964551	0.017764	0.770308
C	-2.515513	7.110415	0.712613
H	-7.457789	5.231860	0.449430
H	1.665049	-2.733577	-3.244602
H	3.969279	-1.769735	-3.230685
H	-6.367118	-2.315143	2.432865
H	-6.157689	0.117651	2.969176
H	1.346650	4.979469	4.013277
H	-0.740278	6.262410	4.493619
C	-4.691952	8.837424	0.981881
H	-7.081907	7.590724	0.949521
C	7.476594	1.194378	1.063626
H	7.503803	3.841469	0.622682
C	-1.105304	-5.910587	-0.364937
C	-3.348371	-4.295972	-0.917069
C	-3.337360	-5.678324	-1.277723
C	-2.202826	-6.471675	-0.950235
H	-0.224455	-6.521121	-0.211433
C	-4.401109	-3.495736	-1.436565
C	-4.415541	-6.223829	-2.017895
H	-2.200441	-7.520919	-1.231250
C	3.484631	-6.471085	1.791727
C	4.759886	-7.195540	2.231514
C	4.680972	-8.710638	2.035694
C	-9.428189	2.113150	-1.622931
C	-10.710297	1.527076	-2.220666
C	-11.934551	2.414512	-1.988804
C	3.582227	8.705505	-1.253988
C	3.594249	10.206513	-1.556272
C	5.000989	10.807029	-1.532641
H	3.783868	-4.745370	3.044860
H	4.440459	-4.576217	1.435532
H	1.546549	8.655747	-0.557269
H	1.719283	8.307535	-2.258949
H	-8.407990	0.227826	-1.429166
H	-8.089276	1.052409	-2.934875
H	3.288571	-6.695964	0.734910
H	2.630709	-6.869540	2.355537
H	4.957714	-6.970195	3.287861
H	5.614613	-6.795336	1.670093
H	3.853609	-9.139795	2.611717
H	5.602730	-9.205594	2.356741
H	4.515650	-8.963685	0.982500
H	-9.248400	3.105789	-2.056817
H	-9.572003	2.268887	-0.545457
H	-10.888516	0.533018	-1.789460
H	-10.567774	1.369872	-3.298058
H	-12.118022	2.561010	-0.918542
H	-12.837231	1.975346	-2.424690
H	-11.794547	3.404117	-2.437819
H	4.215251	8.185194	-1.985035
H	4.039934	8.532107	-0.270939
H	2.958999	10.726945	-0.827205
H	3.135279	10.381120	-2.538398
H	5.469750	10.674063	-0.551219
H	4.983621	11.879218	-1.751271
H	5.647432	10.325499	-2.274769
C	7.312502	-0.141952	1.349856
C	6.033911	-0.724407	1.223280
C	-5.425396	-4.045605	-2.176932
C	-5.452244	-5.429026	-2.451484
C	-2.324374	8.475709	0.731472
C	-3.417017	9.351993	0.899048
H	3.995835	-0.455462	0.701315
H	-1.662110	6.462930	0.568543
H	-5.550677	9.498354	1.061305
H	8.442327	1.673928	1.197416
H	-4.397566	-2.429642	-1.256772
H	-4.392530	-7.283034	-2.258827
H	8.151589	-0.737112	1.696439
H	5.883639	-1.765762	1.492690
H	-6.212029	-3.403247	-2.560977
H	-6.269359	-5.855340	-3.025031
H	-1.322285	8.875739	0.608759
H	-3.255155	10.424966	0.931284

MH 4 (MMPPMM)

156

C	-0.956956	2.012026	0.790703
C	-2.254400	1.472068	0.746718
C	-2.376206	0.027777	0.737293
C	-1.181565	-0.715147	0.549960

C	-0.092331	-0.062154	-0.152531	H	-3.596369	0.777974	-1.350511
C	0.038991	1.329789	-0.011293	C	1.870282	-2.902833	-1.621886
C	-1.090404	-2.047245	1.110275	C	3.145301	-2.307926	-1.627775
C	-3.593611	-0.723761	1.013715	C	-5.948716	-2.264730	0.785590
C	-3.527463	-2.152459	1.019341	C	-6.027992	-0.896460	1.092971
C	-2.258811	-2.788653	1.327624	C	0.810064	4.797427	2.572306
C	-3.324915	2.463229	0.772026	C	-0.267543	5.656865	2.848002
C	-3.186610	3.595659	1.585081	H	-4.959549	0.909260	1.397167
C	-1.844535	4.003907	1.982134	H	-4.647926	-3.951185	0.630665
C	-0.735115	3.215743	1.557742	H	-2.373496	5.938293	2.791318
C	1.062276	2.035401	-0.763987	H	1.398994	2.948466	1.678064
C	2.142647	1.325436	-1.313027	H	4.242693	-0.484572	-1.517402
C	2.132048	-0.127497	-1.224971	H	-0.195257	-2.622195	-1.162012
C	0.907515	-0.784386	-0.910655	H	1.754393	-3.958028	-1.845662
C	-2.154520	-4.156395	1.819625	H	4.030495	-2.910215	-1.806682
C	-0.926348	-4.831471	1.643366	H	-6.848246	-2.843648	0.602608
C	0.291968	-4.046410	1.529790	H	-6.992515	-0.412652	1.209297
C	0.188774	-2.635590	1.474415	H	1.823230	5.081996	2.836695
C	1.344776	-1.869276	1.736355	H	-0.089394	6.631409	3.292016
C	2.577168	-2.466249	1.873173	C	-0.899295	-6.255575	1.727243
C	2.721539	-3.865925	1.762042	C	-3.209114	-4.865339	2.520017
C	1.575457	-4.631479	1.629632	C	-3.150298	-6.290230	2.610139
C	1.008935	3.476075	-0.962858	C	-1.997192	-6.966320	2.119416
C	2.231737	4.173176	-1.113137	H	0.008325	-6.784361	1.462135
C	3.400021	3.427182	-1.551186	C	-4.241784	-4.206964	3.237662
C	3.231316	2.077037	-1.931713	C	-4.193023	-6.997982	3.257493
C	-0.188877	4.221976	-0.984308	H	-1.972246	-8.051859	2.147343
C	-0.169326	5.598402	-0.951206	C	-4.638529	4.977677	3.165267
C	1.051352	6.304817	-0.895364	C	-6.565550	5.213621	1.131524
C	2.227907	5.584057	-1.018870	C	-6.694791	5.972394	2.258469
C	-4.402341	4.349104	1.881141	C	-5.767712	5.837397	3.328793
C	-5.441010	4.358872	0.928160	C	-3.866302	4.686565	4.321420
C	-5.366412	3.470956	-0.221603	H	-7.344966	5.252568	0.380918
C	-4.381767	2.449486	-0.227645	H	-7.549551	6.630541	2.385078
C	-6.274423	3.551332	-1.300810	C	-5.994247	6.472960	4.575038
C	-6.251463	2.655130	-2.356534	C	4.134395	1.561319	-2.942327
C	-5.270246	1.641136	-2.343005	C	5.380370	2.223638	-3.168956
C	-4.359415	1.544366	-1.314791	C	5.647946	3.444302	-2.485870
C	-4.873500	-0.145578	1.198466	C	4.659792	4.059431	-1.771815
C	-4.714383	-2.881942	0.780887	C	3.786821	0.502898	-3.822002
C	-1.561411	5.261408	2.570230	C	6.285702	1.708120	-4.129152
C	0.573151	3.601890	1.929558	H	6.613528	3.922919	-2.621228
C	3.266765	-0.944312	-1.452540	H	4.835941	5.045154	-1.357716
C	0.775111	-2.151999	-1.251733	C	4.210658	-5.980960	1.702031
H	1.261392	-0.792521	1.802012	C	5.656597	-6.483701	1.731484
H	3.456036	-1.852022	2.047786	C	5.760122	-8.005498	1.615370
C	4.112575	-4.460021	1.816323	C	2.360772	8.532825	-0.768376
H	1.662838	-5.709417	1.640890	C	2.222984	10.046510	-0.583586
H	-1.138736	3.702936	-1.009865	C	3.568282	10.774225	-0.610372
H	-1.106065	6.148356	-0.945008	C	-8.257613	3.843526	-3.481712
C	1.014555	7.809766	-0.737828	C	-9.191060	3.824339	-4.695319
H	3.165321	6.118906	-1.092274	C	-10.234835	4.942162	-4.660852
H	-7.004302	4.349109	-1.311372	H	4.599507	-4.136982	2.747178
C	-7.217039	2.724274	-3.521491	H	4.703104	-4.003529	1.008833
H	-5.221841	0.935957	-3.168563	H	0.510784	8.034494	0.213428

H	0.362334	8.230214	-1.515904
H	-7.735806	1.758117	-3.597565
H	-6.635544	2.811110	-4.450247
H	3.732388	-6.311330	0.770202
H	3.648917	-6.451406	2.519961
H	6.136038	-6.151471	2.661801
H	6.219497	-6.012396	0.914818
H	5.232419	-8.500234	2.438354
H	6.801822	-8.340262	1.638235
H	5.316009	-8.360270	0.678585
H	2.868478	8.330065	-1.720811
H	3.012426	8.131826	0.019426
H	1.712807	10.249312	0.367457
H	1.570543	10.448837	-1.369839
H	4.227761	10.412702	0.186547
H	3.444228	11.853329	-0.476516
H	4.084293	10.613253	-1.563462
H	-7.752263	4.817172	-3.431645
H	-8.856181	3.759325	-2.564823
H	-9.695520	2.850407	-4.747879
H	-8.593009	3.906607	-5.612542
H	-10.866879	4.863786	-3.769232
H	-10.889200	4.907599	-5.537371
H	-9.756344	5.927644	-4.640227
C	-5.183463	6.218598	5.657888
C	-4.127470	5.290908	5.531930
C	5.943804	0.632326	-4.917895
C	4.664421	0.052260	-4.784629
C	-5.218305	-4.916969	3.902807
C	-5.220175	-6.327743	3.883430
H	-4.247461	-3.126556	3.283071
H	-4.143242	-8.083057	3.283744
H	-3.063932	3.964347	4.251616
H	-6.844308	7.143526	4.665903
H	2.804075	0.057485	-3.746865
H	7.241801	2.207626	-4.259278
H	-5.372994	6.701405	6.611510
H	-3.521946	5.040298	6.397350
H	6.636952	0.256325	-5.663975
H	4.362321	-0.749866	-5.450814
H	-5.986205	-4.382752	4.453583
H	-6.005737	-6.878178	4.391752

MH 4 (MMPPMP)

156

C	-0.873331	2.010427	0.626011
C	-2.193673	1.520502	0.604963
C	-2.345515	0.083095	0.647677
C	-1.200919	-0.708615	0.369994
C	-0.100347	-0.082573	-0.341556
C	0.102883	1.297864	-0.153995
C	-1.163124	-2.073149	0.863687
C	-3.566841	-0.605134	1.040531
C	-3.605942	-2.029483	0.950864
C	-2.359713	-2.762623	1.104680
C	-3.215609	2.565766	0.568213
C	-2.966827	3.796287	1.195298
C	-1.727387	3.901422	1.962381
C	-0.625860	3.118853	1.525609
C	1.200261	1.973199	-0.818162
C	2.249712	1.226840	-1.379886
C	2.131906	-0.225249	-1.395301
C	0.861900	-0.818075	-1.133212
C	-2.305468	-4.166369	1.495624
C	-1.126925	-4.889170	1.204763
C	0.126175	-4.161002	1.106143
C	0.093805	-2.746589	1.144893
C	1.292985	-2.057054	1.425408
C	2.495014	-2.723006	1.496653
C	2.565171	-4.118357	1.293671
C	1.379380	-4.815572	1.137324
C	1.238253	3.423521	-0.933844
C	2.505627	4.052876	-1.020059
C	3.646846	3.252975	-1.433100
C	3.409745	1.942483	-1.905404
C	0.084995	4.234181	-0.990366
C	0.179195	5.610271	-0.977316
C	1.432910	6.246760	-0.875000
C	2.573383	5.461165	-0.923850
C	-3.945471	4.864323	1.104421
C	-5.259294	4.536234	0.695986
C	-5.484194	3.313378	-0.053561
C	-4.407200	2.410262	-0.240304
C	-6.705870	3.058647	-0.722064
C	-6.859327	2.000684	-1.601176
C	-5.738862	1.180181	-1.857936
C	-4.549169	1.379046	-1.196676
C	-4.758644	0.045649	1.439022
C	-4.866764	-2.649607	0.795746
C	-1.568812	4.706088	3.107217
C	0.629611	3.277402	2.150179
C	3.209175	-1.104494	-1.667593
C	0.640877	-2.150027	-1.558325
H	1.265429	-0.983897	1.562273
H	3.407627	-2.166557	1.690610
C	3.924921	-4.782708	1.280439
H	1.409975	-5.895336	1.080649
H	-0.890995	3.769906	-1.058660
H	-0.722371	6.211345	-1.040959
C	1.512942	7.752583	-0.822800
H	3.537826	5.956074	-0.958644
H	-7.533387	3.739620	-0.579441
C	-8.158862	1.706946	-2.321519
H	-5.822979	0.375368	-2.583158
H	-3.708721	0.727900	-1.398996
C	1.684152	-2.947474	-1.976484
C	2.996072	-2.440872	-1.937909
C	-6.038628	-1.946885	0.997800
C	-5.973779	-0.610660	1.427178
C	0.774832	4.128851	3.229403

C	-0.338733	4.823213	3.730971
H	-4.726607	1.086016	1.720888
H	-4.906238	-3.704723	0.560783
H	-2.423019	5.247353	3.494094
H	1.467366	2.683244	1.804780
H	4.216253	-0.714160	-1.688676
H	-0.359974	-2.557386	-1.496179
H	1.499445	-3.975610	-2.269094
H	3.840254	-3.089049	-2.151306
H	-6.996609	-2.443371	0.880623
H	-6.876203	-0.073990	1.701383
H	1.741390	4.226405	3.713688
H	-0.238658	5.448837	4.612226
C	-1.176744	-6.314689	1.168015
C	-3.353105	-4.875298	2.207433
C	-3.373534	-6.303997	2.175676
C	-2.292792	-6.997096	1.559163
H	-0.315031	-6.864197	0.808006
C	-4.289969	-4.227712	3.054421
C	-4.413192	-7.007259	2.832649
H	-2.331183	-8.080581	1.493295
C	-3.652645	6.264853	1.368764
C	-4.732845	7.175825	1.580954
C	-6.071320	6.713020	1.430768
C	-6.316432	5.464444	0.940066
C	-2.344401	6.812363	1.313139
C	-4.464020	8.537576	1.863473
H	-6.889896	7.395841	1.639028
H	-7.339195	5.163110	0.749789
C	4.323823	1.438218	-2.912915
C	5.620487	2.026198	-3.036863
C	5.936146	3.174987	-2.255980
C	4.955157	3.809923	-1.549756
C	3.946569	0.475515	-3.885101
C	6.533269	1.518367	-3.993934
H	6.937963	3.590876	-2.310837
H	5.172669	4.750755	-1.057784
C	3.943906	-6.295340	1.062488
C	5.362807	-6.869535	1.027109
C	5.387179	-8.383170	0.807037
C	-9.325858	2.653419	-2.040417
C	-10.596859	2.265786	-2.801266
C	-11.767843	3.210012	-2.523146
C	1.234647	8.415127	-2.182374
C	1.267008	9.943962	-2.121026
C	0.987528	10.600125	-3.474488
H	4.443331	-4.548095	2.220741
H	4.524205	-4.302172	0.493515
H	2.503099	8.058808	-0.465237
H	0.785607	8.129740	-0.091798
H	-8.464436	0.680904	-2.071482
H	-7.962678	1.692098	-3.402821
H	3.432630	-6.537651	0.121158
H	3.373968	-6.792045	1.858904
H	5.875205	-6.625397	1.967101
H	5.934000	-6.372033	0.231996
H	4.850174	-8.906166	1.606182
H	6.410871	-8.769577	0.785000
H	4.908773	-8.651194	-0.141640
H	-9.043298	3.679676	-2.310271
H	-9.540196	2.665917	-0.963378
H	-10.879470	1.239007	-2.533508
H	-10.383057	2.250948	-3.878203
H	-12.022367	3.218911	-1.457423
H	-12.663580	2.912973	-3.077309
H	-11.522241	4.238004	-2.812010
H	0.255158	8.083593	-2.550591
H	1.971195	8.054863	-2.912331
H	2.246654	10.271009	-1.747326
H	0.529547	10.289744	-1.384159
H	1.728689	10.294133	-4.221105
H	1.014721	11.692127	-3.405109
H	0.000361	10.313897	-3.853813
C	-2.111264	8.151486	1.547667
C	-3.175005	9.022480	1.858963
C	6.156419	0.530577	-4.876499
C	4.835602	0.035714	-4.842406
C	-5.260933	-4.938694	3.726922
C	-5.352920	-6.339376	3.585837
H	-4.223328	-3.157546	3.194296
H	-4.427887	-8.091554	2.764325
H	-1.511241	6.174568	1.061768
H	-5.303662	9.201502	2.049831
H	2.933411	0.096837	-3.884675
H	7.526075	1.956724	-4.045609
H	-1.096760	8.534087	1.487728
H	-2.981254	10.071148	2.061989
H	6.856856	0.161871	-5.619372
H	4.510995	-0.690845	-5.580753
H	-5.953128	-4.414021	4.378107
H	-6.134949	-6.888475	4.101031

MH 4 (MMPPPM)

156

C	-0.865690	2.154451	0.538299
C	-2.160511	1.637437	0.707402
C	-2.317554	0.200992	0.689530
C	-1.235094	-0.577701	0.227041
C	-0.198711	0.066546	-0.556264
C	-0.007265	1.453448	-0.395530
C	-1.230996	-2.001426	0.508023
C	-3.427926	-0.489399	1.340083
C	-3.570524	-1.896920	1.141826
C	-2.464527	-2.656667	0.579620
C	-3.217113	2.602502	0.971478
C	-2.923325	3.740149	1.733717
C	-1.533263	4.165725	1.836559
C	-0.512698	3.362988	1.250934
C	0.937424	2.146148	-1.258493
C	1.946950	1.422547	-1.911995

C	1.919662	-0.029473	-1.824440
C	0.703642	-0.661070	-1.430840
C	-2.493777	-4.077368	0.263351
C	-1.319232	-4.835793	0.456191
C	-0.093970	-4.171623	0.876494
C	-0.033955	-2.756692	0.824973
C	1.163839	-2.116696	1.217488
C	2.268114	-2.844216	1.597692
C	2.236941	-4.255291	1.626597
C	1.054659	-4.888080	1.281381
C	0.871323	3.584732	-1.472463
C	2.074617	4.270352	-1.773846
C	3.189992	3.507810	-2.309334
C	2.974587	2.153747	-2.648068
C	-0.317305	4.336401	-1.364216
C	-0.292026	5.714598	-1.367798
C	0.927448	6.411581	-1.486622
C	2.081420	5.681616	-1.722412
C	-4.049779	4.453633	2.327452
C	-5.298973	4.409824	1.674538
C	-5.498480	3.490670	0.564771
C	-4.506275	2.509952	0.304388
C	-6.675454	3.498128	-0.217074
C	-6.908131	2.567878	-1.215736
C	-5.914028	1.596822	-1.458905
C	-4.747049	1.573484	-0.728311
C	-4.334574	0.153389	2.213471
C	-4.657182	-2.564472	1.758704
C	-1.158813	5.442028	2.326258
C	0.839789	3.744916	1.412447
C	3.012508	-0.865842	-2.155096
C	0.502576	-2.003248	-1.833448
H	1.206769	-1.035603	1.227265
H	3.176200	-2.325484	1.893266
C	3.483357	-5.005337	2.048342
H	1.005955	-5.966692	1.340206
H	-1.262724	3.820906	-1.252678
H	-1.218943	6.269599	-1.256561
C	0.946189	7.919501	-1.454562
H	3.000048	6.222773	-1.921378
H	-7.418119	4.262981	-0.037748
C	-8.166406	2.556133	-2.058741
H	-6.063734	0.868336	-2.251592
H	-3.987773	0.842343	-0.969827
C	1.545982	-2.769626	-2.310723
C	2.838460	-2.218944	-2.368396
C	-5.563877	-1.890442	2.550497
C	-5.390307	-0.520083	2.794660
C	1.179525	4.954832	1.976765
C	0.162433	5.836847	2.382415
H	-4.187521	1.193580	2.460762
H	-4.744471	-3.636450	1.649651
H	-1.926638	6.128046	2.653642
H	1.614630	3.076326	1.060002
H	3.992445	-0.426616	-2.280057

H	-0.476575	-2.448343	-1.719968
H	1.374681	-3.805902	-2.582876
H	3.689763	-2.839729	-2.628651
H	-6.377487	-2.433929	3.020752
H	-6.063945	0.010073	3.460239
H	2.223166	5.234425	2.076531
H	0.412122	6.824969	2.756049
C	-3.977080	5.090042	3.629470
C	-6.359981	5.240175	2.146413
C	-6.221678	6.018558	3.258401
C	-5.052992	5.923175	4.063710
C	-2.938344	4.828820	4.562417
H	-7.304610	5.247214	1.617714
H	-7.034633	6.659288	3.587595
C	-4.981237	6.569297	5.322903
C	-1.354285	-6.243080	0.226566
C	-3.638588	-4.705178	-0.367157
C	-3.641561	-6.120832	-0.560673
C	-2.483278	-6.870192	-0.214639
H	-0.460101	-6.830230	0.393952
C	-4.729330	-3.970752	-0.904600
C	-4.762343	-6.748824	-1.158598
H	-2.491556	-7.945624	-0.367199
C	3.769980	1.615079	-3.734906
C	4.995314	2.260311	-4.088547
C	5.338549	3.487621	-3.452720
C	4.427767	4.124541	-2.659251
C	3.327298	0.552650	-4.565567
C	5.799489	1.722611	-5.123659
H	6.291819	3.953090	-3.685817
H	4.650686	5.114226	-2.278402
C	3.395631	-6.531543	2.047354
C	4.708366	-7.198930	2.466659
C	4.625330	-8.726434	2.467597
C	-9.195998	3.647499	-1.765591
C	-10.430806	3.550759	-2.665853
C	-11.464858	4.640287	-2.376345
C	0.452169	8.550674	-2.767175
C	0.446980	10.080760	-2.734228
C	-0.044744	10.702855	-4.042645
H	3.772824	-4.665301	3.052819
H	4.308956	-4.695714	1.391883
H	1.963313	8.271591	-1.245002
H	0.316329	8.272380	-0.627582
H	-8.647243	1.573856	-1.947357
H	-7.876097	2.614887	-3.117263
H	3.115130	-6.884414	1.045892
H	2.594499	-6.856930	2.724218
H	4.990767	-6.844536	3.466868
H	5.510912	-6.873734	1.791374
H	3.851832	-9.078594	3.159136
H	5.574312	-9.180093	2.769840
H	4.375311	-9.108066	1.471352
H	-8.733926	4.635569	-1.892561
H	-9.510336	3.586704	-0.715142

H	-10.892249	2.562136	-2.541366
H	-10.117518	3.609747	-3.716631
H	-11.818305	4.582959	-1.340786
H	-12.336792	4.550599	-3.031601
H	-11.037532	5.638336	-2.524122
H	-0.559376	8.183655	-2.984888
H	1.085670	8.198527	-3.591752
H	1.460537	10.441127	-2.512534
H	-0.184785	10.421641	-1.903013
H	0.588985	10.402061	-4.884265
H	-0.039581	11.796370	-3.994966
H	-1.067704	10.383252	-4.270384
C	-3.922533	6.346702	6.173891
C	-2.909003	5.440227	5.797091
C	4.103897	0.080871	-5.602111
C	5.371830	0.642277	-5.862811
C	-5.796278	-4.603208	-1.505368
C	-5.830517	-6.009573	-1.613329
H	-2.160894	4.123054	4.303688
H	-5.799958	7.220383	5.616653
H	-4.719347	-2.890780	-0.850283
H	-4.748317	-7.829150	-1.273559
H	2.350220	0.121901	-4.394299
H	6.744109	2.209121	-5.350683
H	-3.882954	6.836520	7.141838
H	-2.104030	5.211238	6.488459
H	3.729709	-0.723618	-6.227585
H	5.986429	0.249164	-6.666669
H	-6.611544	-4.010489	-1.908642
H	-6.680626	-6.499820	-2.077395

MH 4 (MMPPPP)

156

C	-0.832942	2.454529	0.362963
C	-2.133223	1.916752	0.319735
C	-2.235170	0.479566	0.358995
C	-1.092343	-0.290583	0.052113
C	0.050700	0.366682	-0.550874
C	0.206022	1.753679	-0.345468
C	-1.131516	-1.720139	0.317291
C	-3.395971	-0.210041	0.909895
C	-3.525335	-1.611768	0.691822
C	-2.369013	-2.368807	0.235510
C	-3.226746	2.880942	0.305023
C	-3.032607	4.148332	0.872672
C	-1.791121	4.360660	1.611429
C	-0.653564	3.597703	1.237277
C	1.345362	2.446462	-0.918228
C	2.470381	1.720785	-1.342757
C	2.388323	0.266282	-1.345537
C	1.104179	-0.346722	-1.247366
C	-2.372887	-3.782966	-0.104483
C	-1.229667	-4.553910	0.196960
C	-0.060566	-3.908297	0.775366
C	0.007560	-2.492551	0.773914
C	1.140180	-1.870643	1.348142
C	2.186592	-2.614038	1.842616
C	2.157139	-4.025246	1.810759
C	1.029251	-4.641939	1.296392
C	1.356210	3.893250	-1.065750
C	2.605283	4.559156	-1.032225
C	3.807245	3.786569	-1.296783
C	3.662292	2.457265	-1.754163
C	0.193101	4.669849	-1.254696
C	0.249695	6.046809	-1.256460
C	1.467288	6.724959	-1.032313
C	2.626651	5.971299	-0.953284
C	-4.089240	5.140964	0.796030
C	-5.405044	4.700012	0.529928
C	-5.607743	3.404241	-0.096216
C	-4.482372	2.581311	-0.356919
C	-6.873363	2.997347	-0.582226
C	-7.036568	1.865346	-1.361279
C	-5.883440	1.131443	-1.715673
C	-4.644497	1.482548	-1.231366
C	-4.365886	0.441087	1.702845
C	-4.671969	-2.272746	1.196976
C	-1.679207	5.244560	2.705424
C	0.577389	3.824922	1.891991
C	3.505598	-0.594044	-1.473396
C	0.961093	-1.667826	-1.733635
H	1.177184	-0.790929	1.405900
H	3.046012	-2.108703	2.275065
C	3.343942	-4.792724	2.355055
H	0.974507	-5.721814	1.311613
H	-0.757689	4.175014	-1.411322
H	-0.655181	6.621602	-1.429458
C	1.451772	8.235138	-0.925768
H	3.579962	6.479448	-0.896372
C	-3.865743	6.569208	0.938596
C	-6.496781	5.584620	0.784894
H	-7.738379	3.610544	-0.371880
C	-8.383413	1.403523	-1.877211
H	-5.978883	0.276914	-2.380576
H	-3.777200	0.902698	-1.517144
C	2.063340	-2.446070	-2.020344
C	3.349775	-1.930961	-1.782530
C	-5.642100	-1.591002	1.903554
C	-5.478682	-0.223938	2.176639
C	0.673276	4.743121	2.918751
C	-0.469519	5.441314	3.344815
C	-4.981054	7.432730	1.161738
C	-6.294667	6.883275	1.150499
H	-4.227200	1.481847	1.956993
H	-4.760212	-3.343193	1.071868
H	-2.558924	5.775395	3.045757
H	1.439665	3.233745	1.609726
H	4.500198	-0.186538	-1.361486
H	-0.031754	-2.087024	-1.831404
C	-2.596753	7.177183	0.754030

H	-7.510853	5.214870	0.697973
H	1.931898	-3.466619	-2.364315
H	4.223511	-2.566607	-1.884644
H	-6.503305	-2.127785	2.289365
H	-6.208731	0.307390	2.778702
H	1.623296	4.892122	3.422312
H	-0.409046	6.124761	4.185885
C	-4.774415	8.825260	1.320100
H	-7.137828	7.533937	1.363831
C	-1.244957	-5.954715	-0.070508
C	-3.450464	-4.389222	-0.861837
C	-3.438114	-5.800041	-1.087961
C	-2.323505	-6.563894	-0.643587
H	-0.375773	-6.551436	0.176269
C	-4.480018	-3.636869	-1.488273
C	-4.494646	-6.407948	-1.810624
H	-2.319311	-7.635174	-0.823190
C	4.699659	1.954899	-2.635779
C	5.104806	4.379970	-1.279953
C	6.177573	3.756291	-1.849274
C	5.985816	2.577909	-2.626451
C	4.463131	0.958848	-3.618615
H	5.239491	5.339301	-0.794167
H	7.167423	4.200696	-1.800960
C	7.016859	2.073499	-3.456727
C	3.254498	-6.317834	2.301702
C	4.505855	-7.002657	2.858163
C	4.421206	-8.529211	2.806977
C	-9.587320	2.276565	-1.523468
C	-10.901420	1.724607	-2.082985
C	-12.109701	2.594151	-1.730756
C	2.810407	8.935962	-0.934269
C	2.687560	10.459820	-0.849185
C	4.043886	11.167174	-0.847555
H	3.514448	-4.481437	3.395403
H	4.241368	-4.469490	1.808409
H	0.921494	8.505662	-0.000555
H	0.833874	8.636653	-1.740344
H	-8.561490	0.384467	-1.504994
H	-8.322595	1.303691	-2.970059
H	3.096622	-6.642360	1.264452
H	2.376737	-6.657146	2.867587
H	4.665517	-6.676909	3.894575
H	5.384778	-6.662998	2.294239
H	3.569086	-8.896323	3.389783
H	5.325913	-8.995439	3.209311
H	4.293188	-8.882462	1.777663
H	-9.431491	3.294065	-1.905999
H	-9.670343	2.366633	-0.432178
H	-11.056658	0.706221	-1.702691
H	-10.819275	1.632308	-3.174097
H	-12.232894	2.676363	-0.645091
H	-13.035875	2.179812	-2.140898
H	-11.993772	3.609003	-2.127212
H	3.358761	8.665016	-1.846252
H	3.417097	8.578523	-0.091782
H	2.131258	10.727095	0.059241
H	2.084110	10.821263	-1.692330
H	4.653898	10.847690	0.004758
H	3.930009	12.253955	-0.787249
H	4.607232	10.940609	-1.759564
C	6.771389	1.053211	-4.348642
C	5.468815	0.521701	-4.454082
C	-3.519076	9.375541	1.186174
C	-2.427580	8.540694	0.869071
C	-5.483151	-4.249780	-2.207603
C	-5.509898	-5.652948	-2.352487
H	-1.746217	6.562562	0.500868
H	-5.636600	9.456559	1.516651
H	-4.473701	-2.558512	-1.407167
H	-4.472138	-7.485352	-1.949306
H	3.466826	0.552315	-3.727165
H	7.996898	2.539793	-3.405874
H	7.562915	0.686698	-4.994843
H	5.251030	-0.231397	-5.204959
H	-3.374234	10.446033	1.293727
H	-1.444685	8.972179	0.705204
H	-6.252605	-3.643762	-2.676091
H	-6.310262	-6.127921	-2.911252

MH 8 (1st)

246

C	-0.600234	2.424113	0.929588
C	-1.889944	1.871629	0.781134
C	-1.980980	0.437256	0.714670
C	-0.829112	-0.295131	0.361810
C	0.335127	0.412905	-0.098604
C	0.493759	1.761078	0.278328
C	-0.867094	-1.739683	0.397800
C	-3.147871	-0.308286	1.165749
C	-3.252354	-1.688253	0.827623
C	-2.107171	-2.377023	0.250235
C	-2.997806	2.809129	0.720657
C	-2.847574	4.087164	1.278484
C	-1.647646	4.358538	2.057403
C	-0.491267	3.573984	1.808537
C	1.743310	2.432735	-0.014909
C	2.878721	1.639313	-0.246750
C	2.651942	0.382101	-0.941103
C	1.345262	-0.176555	-0.958335
C	-2.116963	-3.700029	-0.348630
C	-0.927882	-4.459854	-0.331001
C	0.211415	-3.954393	0.422583
C	0.285898	-2.566329	0.684688
C	1.410758	-2.072251	1.389801
C	2.418391	-2.915366	1.806676
C	2.335862	-4.309126	1.588973
C	1.218940	-4.797832	0.936992
C	1.890921	3.861833	-0.207882
C	3.191258	4.423623	-0.183712

C	4.310394	3.584191	0.225315	H	1.823879	-2.607288	-3.308145
C	4.174222	2.185346	0.107545	H	4.140082	-1.673610	-3.226844
C	0.812178	4.684890	-0.616548	H	-6.222375	-2.391700	2.365986
C	1.008096	6.005551	-0.959849	H	-5.999850	0.011560	3.019805
C	2.302784	6.570229	-0.963977	H	1.615742	4.966327	4.104211
C	3.364080	5.756388	-0.614095	H	-0.431098	6.317317	4.575370
C	-3.926993	5.043687	1.168278	C	-4.790085	8.646623	0.525563
C	-5.223552	4.548740	0.948660	C	7.604714	1.090873	1.338719
C	-5.346455	3.331385	0.173974	C	7.960467	3.980707	0.064558
C	-4.201263	2.519543	-0.036829	C	-0.924449	-5.766515	-0.921719
C	-6.538447	3.034288	-0.529579	C	-3.232028	-4.188093	-1.139313
C	-6.632489	1.969051	-1.403660	C	-3.263043	-5.544117	-1.558570
C	-5.469222	1.202846	-1.644068	C	-2.149708	-6.393709	-1.201850
C	-4.287577	1.482039	-0.994245	C	0.267476	-6.498696	-1.338379
C	-4.148621	0.267147	1.979778	C	-4.268823	-3.302433	-1.574458
C	-4.383765	-2.412534	1.275614	C	-4.297533	-5.970881	-2.490888
C	-1.601614	5.333776	3.077956	C	-2.189870	-7.841156	-1.018519
C	0.681032	3.822094	2.558000	C	3.360761	-6.662367	1.535687
C	3.647911	-0.188745	-1.769882	C	4.570243	-7.527159	1.893448
C	1.072076	-1.248563	-1.836443	C	4.440309	-8.947822	1.337824
H	1.461290	-1.018642	1.633216	C	-9.177147	2.319142	-1.585118
H	3.269255	-2.505440	2.345555	C	-10.466058	1.814580	-2.237799
C	3.444712	-5.227141	2.063361	C	-11.711092	2.525002	-1.702780
H	1.111367	-5.862097	0.805834	C	3.913590	8.566030	-1.086323
C	5.587097	4.133148	0.579989	C	4.055609	10.067197	-1.341365
C	5.272061	1.342551	0.544010	C	5.476465	10.562533	-1.059180
H	-0.178977	4.258561	-0.698474	H	3.467491	-5.239176	3.162570
H	0.156979	6.608019	-1.267838	H	4.405022	-4.787960	1.757890
C	2.507118	8.021234	-1.351207	H	1.776564	8.628209	-0.797728
H	4.365926	6.149436	-0.667893	H	2.252819	8.157447	-2.412132
C	-3.752746	6.484125	1.085888	H	-8.063182	0.534267	-2.052326
C	-6.327779	5.353357	1.432260	H	-7.813910	1.836577	-3.190109
H	-7.391226	3.680090	-0.381761	H	3.249314	-6.639868	0.444707
C	-7.922476	1.622151	-2.117078	H	2.454986	-7.145959	1.923983
H	-5.504939	0.390858	-2.366082	H	4.692467	-7.563731	2.984185
H	-3.405859	0.898388	-1.222253	H	5.481978	-7.058604	1.499122
C	2.061311	-1.791539	-2.632236	H	3.561594	-9.452596	1.755317
C	3.363922	-1.263439	-2.588362	H	5.318771	-9.557384	1.571623
C	-5.372956	-1.808058	2.025055	H	4.320172	-8.930042	0.248634
C	-5.249559	-0.457304	2.390487	H	-9.097461	3.401992	-1.746622
C	0.710984	4.807208	3.525556	H	-9.239504	2.178490	-0.498098
C	-0.441694	5.566021	3.792110	H	-10.557540	0.733143	-2.069529
C	-4.895346	7.316037	1.088811	H	-10.402390	1.948867	-3.325944
C	-6.140340	6.736952	1.572515	H	-11.804306	2.389977	-0.619442
C	6.561554	1.905025	0.730166	H	-12.624828	2.143134	-2.168783
C	6.729726	3.320145	0.488300	H	-11.662236	3.602428	-1.895788
H	-4.037588	1.292712	2.304126	H	4.639924	8.032131	-1.712830
H	-4.447342	-3.471124	1.059647	H	4.194122	8.353088	-0.047391
H	-2.496182	5.900651	3.304063	H	3.342038	10.612875	-0.709450
H	1.551243	3.198043	2.398223	H	3.781204	10.295693	-2.379838
H	4.635477	0.254708	-1.785624	H	5.766104	10.345569	-0.024710
H	0.063119	-1.637342	-1.895728	H	5.569329	11.641551	-1.217323
C	5.809947	5.476326	1.105989	H	6.201659	10.064530	-1.712883
C	5.061069	-0.036310	0.867484	C	7.372954	-0.303706	1.543527
C	-2.478267	7.046417	0.753979	C	6.078335	-0.837655	1.285980

C	-5.321400	-3.740950	-2.317444
C	-5.368170	-5.078453	-2.803122
C	-2.350559	8.360787	0.415717
C	-3.496355	9.190285	0.255178
H	4.069943	-0.454961	0.777873
H	-1.604371	6.411620	0.743350
C	-5.915409	9.410446	0.115849
C	8.814344	1.624351	1.856026
H	-4.213517	-2.257744	-1.306031
C	8.388726	-1.117824	2.099707
H	5.892899	-1.886728	1.499762
H	-6.106215	-3.050883	-2.614121
C	-6.403520	-5.489476	-3.676794
H	-1.371696	8.768912	0.178968
C	-3.376298	10.504070	-0.259700
C	9.780782	0.815399	2.417190
C	9.585203	-0.576432	2.514284
C	-4.492607	11.245505	-0.576547
C	-5.772038	10.673926	-0.416352
H	-6.905323	8.984257	0.201012
H	8.982520	2.690956	1.828717
H	8.189999	-2.179166	2.220244
H	-7.216298	-4.796123	-3.874514
H	-2.380160	10.902998	-0.430312
H	10.692817	1.262108	2.800691
H	10.355435	-1.207942	2.945873
H	-4.390287	12.247735	-0.980848
H	-6.653803	11.226757	-0.725000
C	-7.545274	4.751087	1.955309
C	-7.177599	7.536386	2.194248
C	-8.651996	5.583230	2.307418
C	-8.487318	7.026763	2.306111
C	-9.565284	7.967892	2.545772
C	-7.632116	3.362418	2.221928
C	-6.891262	8.821251	2.760474
C	-9.250897	9.266526	3.053963
C	-7.881964	9.626418	3.233559
C	-9.800858	4.964530	2.865587
C	-10.919160	7.704894	2.216131
H	-5.857913	9.134707	2.829521
C	-10.291620	10.182337	3.331222
H	-7.644668	10.578869	3.697921
C	-9.878292	3.599349	3.050064
C	-8.778427	2.788148	2.729940
H	-6.773480	2.734296	2.033622
H	-10.610033	5.581176	3.228123
H	-10.768940	3.165773	3.494201
H	-8.808980	1.719561	2.919026
C	-11.914173	8.631599	2.461127
C	-11.606287	9.870604	3.054499
H	-11.172216	6.782390	1.709637
H	-10.029946	11.153649	3.741695
H	-12.937628	8.406764	2.177347
H	-12.395029	10.587577	3.259667
C	7.088575	6.079675	0.962956
C	8.085212	5.381827	0.159695
C	4.839647	6.103681	1.919054
C	8.960596	3.234632	-0.633308
C	7.352440	7.274064	1.669641
C	9.155810	6.050981	-0.539983
C	10.048329	3.847913	-1.180867
C	10.176328	5.265355	-1.164473
H	8.838225	2.164145	-0.731447
C	9.211635	7.458675	-0.728312
H	10.805421	3.262217	-1.694270
C	11.254916	5.904751	-1.821387
C	6.381519	7.881161	2.447118
C	5.112193	7.294201	2.566752
H	3.877491	5.625562	2.054970
H	8.350318	7.692471	1.645551
H	6.616905	8.789904	2.992171
H	4.355534	7.750359	3.197351
C	10.261301	8.054956	-1.395586
C	11.312490	7.277137	-1.922516
H	8.393303	8.075213	-0.382106
H	12.027566	5.285283	-2.268315
H	10.266811	9.132257	-1.530216
H	12.141154	7.756145	-2.434494
C	0.215193	-7.914500	-1.447136
C	-0.994418	-8.588122	-0.989674
C	1.404082	-5.811500	-1.820544
C	-3.421498	-8.476292	-0.667429
C	1.295604	-8.582317	-2.065580
C	-1.015276	-9.946893	-0.503197
C	-4.249626	-7.193189	-3.211621
C	-5.257577	-7.563195	-4.077855
C	-6.365935	-6.720494	-4.292804
H	-3.398217	-7.847797	-3.097795
H	-5.183804	-8.507014	-4.609083
H	-7.161286	-7.024098	-4.966281
C	2.464435	-6.495065	-2.385774
C	2.407442	-7.891554	-2.515765
H	1.428480	-4.730088	-1.768489
H	1.227491	-9.648318	-2.240018
H	3.321016	-5.944498	-2.761951
H	3.215740	-8.428561	-3.002199
C	-2.275368	-10.569187	-0.231621
C	-3.467193	-9.798196	-0.336288
C	0.156978	-10.685388	-0.186021
C	-2.316771	-11.913806	0.208878
C	0.088081	-11.986491	0.266731
C	-1.158347	-12.622640	0.438207
H	-4.328031	-7.885605	-0.658992
H	-4.414728	-10.267033	-0.086925
H	1.123799	-10.205951	-0.256935
H	-3.287056	-12.369092	0.387064
H	1.003324	-12.517781	0.509237
H	-1.202460	-13.651188	0.782305

MH 8 (2nd)

246

C	-0.614990	2.285225	1.339258
C	-1.907822	1.724181	1.247410
C	-1.981585	0.294443	1.092699
C	-0.848565	-0.389118	0.604818
C	0.267637	0.368947	0.109029
C	0.438683	1.689071	0.569616
C	-0.857005	-1.833477	0.548111
C	-3.094648	-0.507683	1.581779
C	-3.199948	-1.865554	1.162159
C	-2.091947	-2.489592	0.453600
C	-3.024657	2.652975	1.326700
C	-2.837410	3.901846	1.936815
C	-1.601043	4.107239	2.682921
C	-0.456685	3.361033	2.299677
C	1.664097	2.393362	0.251077
C	2.785642	1.630902	-0.114961
C	2.519687	0.429590	-0.889752
C	1.219949	-0.143603	-0.858649
C	-2.122688	-3.772255	-0.227141
C	-0.920828	-4.501729	-0.350154
C	0.262643	-4.018493	0.347732
C	0.329784	-2.649067	0.695337
C	1.493270	-2.175743	1.350392
C	2.544772	-3.019915	1.636256
C	2.474133	-4.397617	1.330246
C	1.321927	-4.869243	0.729238
C	1.795263	3.834393	0.167681
C	3.093023	4.402762	0.158033
C	4.238196	3.541937	0.425332
C	4.099368	2.156605	0.200270
C	0.690965	4.679423	-0.105246
C	0.861942	6.025287	-0.350457
C	2.152160	6.599072	-0.386514
C	3.235144	5.767723	-0.170369
C	-3.910857	4.880914	1.931509
C	-5.224041	4.405548	1.775973
C	-5.414131	3.180578	1.007353
C	-4.291250	2.384394	0.666345
C	-6.653553	2.899883	0.389897
C	-6.825496	1.873979	-0.518170
C	-5.688357	1.123040	-0.886394
C	-4.457469	1.387546	-0.323998
C	-4.039353	-0.009633	2.505496
C	-4.277401	-2.644404	1.648927
C	-1.507421	4.963119	3.802681
C	0.756219	3.549544	3.000632
C	3.459803	-0.064240	-1.826046
C	0.895528	-1.154294	-1.790237
H	1.540936	-1.139786	1.660969
H	3.424113	-2.626025	2.140121
C	3.632997	-5.317274	1.659686
H	1.225550	-5.924849	0.534665
C	5.532900	4.070833	0.743227
C	5.226062	1.290286	0.494867
H	-0.301824	4.252439	-0.161778
H	-0.007250	6.644965	-0.557092
C	2.328506	8.078522	-0.664536
H	4.230579	6.171793	-0.252998
C	-3.703258	6.303414	2.084002
C	-6.321895	5.258000	2.137315
H	-7.489270	3.540801	0.608784
C	-8.181429	1.590003	-1.134212
H	-5.776931	0.345627	-1.641203
H	-3.594920	0.825241	-0.654582
C	1.831310	-1.623643	-2.690713
C	3.128387	-1.080172	-2.699954
C	-5.214745	-2.113480	2.512122
C	-5.089510	-0.785934	2.952863
C	0.832524	4.429059	4.062614
C	-0.312498	5.131560	4.475714
C	-4.802415	7.169845	2.315197
C	-6.142943	6.651610	2.173533
C	6.522814	1.846826	0.645068
C	6.670251	3.278621	0.511760
H	-3.924667	0.997302	2.882626
H	-4.337522	-3.688219	1.369403
H	-2.392700	5.484640	4.143271
H	1.619977	2.955612	2.729658
H	4.440131	0.392579	-1.876755
H	-0.111074	-1.553513	-1.805404
C	5.783797	5.367844	1.363813
C	5.041238	-0.112288	0.715831
C	-2.414264	6.890493	1.847071
H	1.555028	-2.392548	-3.405634
H	3.861142	-1.431362	-3.419716
H	-6.022845	-2.738144	2.879755
H	-5.799386	-0.376137	3.664507
H	1.767453	4.541535	4.603007
H	-0.267553	5.789862	5.337461
C	7.605657	0.994391	1.116477
C	7.868790	3.983191	0.067120
C	-0.936470	-5.767211	-1.024197
C	-3.286037	-4.237721	-0.959810
C	-3.321515	-5.564403	-1.464208
C	-2.166510	-6.406370	-1.250841
C	0.234633	-6.440355	-1.577064
C	-4.372677	-3.353729	-1.253506
C	-4.416615	-5.956930	-2.340635
C	-2.162322	-7.863161	-1.160300
C	3.538907	-6.715890	1.042923
C	4.788006	-7.572785	1.253124
C	4.645131	-8.956026	0.612735
C	-9.318615	2.479177	-0.622341
C	-10.687927	2.150275	-1.218359
C	-11.783044	3.070131	-0.670846
C	3.747213	8.610154	-0.441338
C	3.870189	10.128190	-0.579788
C	5.304571	10.608516	-0.343815
H	3.737236	-5.401722	2.750976

H	4.559237	-4.835116	1.316021
H	1.631279	8.632503	-0.020063
H	2.009718	8.299439	-1.693285
H	-8.433957	0.536551	-0.947012
H	-8.107610	1.681967	-2.227004
H	3.347392	-6.623703	-0.033180
H	2.673683	-7.246239	1.461466
H	4.990635	-7.678850	2.327211
H	5.659069	-7.057005	0.827050
H	3.809432	-9.508686	1.057044
H	5.550044	-9.558319	0.740422
H	4.444844	-8.868568	-0.461178
H	-9.086623	3.530163	-0.838343
H	-9.377765	2.400672	0.471168
H	-10.941318	1.103666	-1.002918
H	-10.644301	2.235901	-2.312218
H	-11.858718	2.979685	0.418898
H	-12.762893	2.832266	-1.096547
H	-11.563883	4.119889	-0.897362
H	4.435262	8.134963	-1.152599
H	4.091248	8.314610	0.557528
H	3.195117	10.615035	0.136830
H	3.532780	10.439445	-1.577372
H	5.656822	10.309056	0.649839
H	5.385597	11.697505	-0.417630
H	5.989751	10.171038	-1.078934
C	7.392569	-0.414386	1.218961
C	6.085979	-0.936516	1.000970
C	-5.470929	-3.770833	-1.940633
C	-5.527095	-5.074688	-2.509633
H	4.048225	-0.531186	0.655317
H	-1.614741	6.260259	1.483170
C	8.844013	1.493955	1.598480
H	-4.318713	-2.327756	-0.920499
C	8.444855	-1.262687	1.639638
H	5.917959	-2.001219	1.138267
H	-6.291308	-3.084098	-2.129008
C	-6.618614	-5.455373	-3.326877
C	9.847369	0.649964	2.027746
C	9.663501	-0.746827	2.020826
H	9.006259	2.560609	1.649428
H	8.258048	-2.332092	1.683827
H	-7.458943	-4.772362	-3.415092
H	10.780479	1.071502	2.388359
H	10.462134	-1.405001	2.348430
C	-7.613496	4.746302	2.577930
C	-7.325437	7.453124	1.827006
C	-8.747763	5.598495	2.559817
C	-8.615050	6.884519	1.890032
C	-9.727835	7.546972	1.249706
C	-7.715355	3.479589	3.196083
C	-7.170672	8.732646	1.206585
C	-9.544957	8.864555	0.724869
C	-8.240894	9.430436	0.729114
C	-9.917948	5.178614	3.229461

C	-10.983954	6.923607	1.013140
H	-6.180148	9.147390	1.095046
C	-10.633209	9.545600	0.127611
H	-8.094563	10.406809	0.276079
C	-9.998284	3.931568	3.824359
C	-8.893073	3.066886	3.790131
H	-6.843000	2.838234	3.224533
H	-10.751921	5.863435	3.314686
H	-10.905985	3.637532	4.341922
H	-8.944922	2.094104	4.268911
C	-12.019785	7.598523	0.401481
C	-11.858951	8.935234	-0.017708
H	-11.121923	5.883644	1.274333
H	-10.470980	10.554641	-0.241190
H	-12.961353	7.086882	0.226930
H	-12.684368	9.463261	-0.484792
C	7.047999	5.993017	1.189823
C	7.993803	5.372857	0.269855
C	4.865094	5.916757	2.286189
C	8.825182	3.306483	-0.752194
C	7.351451	7.128045	1.974245
C	9.014730	6.107209	-0.438650
C	9.873338	3.972762	-1.314796
C	9.996488	5.385159	-1.189261
H	8.700905	2.246686	-0.930831
C	9.052506	7.526072	-0.513349
H	10.598350	3.438380	-1.921927
C	11.028343	6.086464	-1.857965
C	6.429837	7.659913	2.859383
C	5.173518	7.052714	3.011165
H	3.915854	5.419686	2.443393
H	8.343821	7.556734	1.921183
H	6.695817	8.523163	3.461353
H	4.457127	7.448058	3.724434
C	10.054929	8.185001	-1.194210
C	11.073642	7.463004	-1.848874
H	8.255316	8.104276	-0.066346
H	11.773469	5.513097	-2.402493
H	10.047273	9.269719	-1.239625
H	11.865988	7.990208	-2.370886
C	0.203530	-7.847231	-1.773857
C	-0.952874	-8.579369	-1.271018
C	1.316331	-5.694860	-2.097398
C	-3.349512	-8.550991	-0.758945
C	1.246838	-8.446396	-2.514299
C	-0.907514	-9.966202	-0.873595
C	-4.399186	-7.128301	-3.142764
C	-5.463680	-7.467844	-3.951942
C	-6.603111	-6.642465	-4.024643
H	-3.527610	-7.766022	-3.138497
H	-5.411629	-8.372950	-4.549035
H	-7.442039	-6.922620	-4.653969
C	2.344110	-6.313655	-2.784317
C	2.306258	-7.700045	-3.000732
H	1.322219	-4.618685	-1.976600

H	1.187812	-9.500604	-2.752281
H	3.157745	-5.718491	-3.186997
H	3.085804	-8.184084	-3.580700
C	-2.129484	-10.636920	-0.548385
C	-3.341747	-9.891963	-0.512108
C	0.301032	-10.692502	-0.694443
C	-2.108452	-12.007308	-0.194313
C	0.294692	-12.020966	-0.323299
C	-0.921112	-12.698884	-0.099841
H	-4.264817	-7.985687	-0.643107
H	-4.257133	-10.400007	-0.222662
H	1.249233	-10.184584	-0.806704
H	-3.052300	-12.497973	0.026798
H	1.236915	-12.542505	-0.185587
H	-0.916857	-13.747932	0.178989
C	-4.508887	8.518545	2.777916
C	-2.187594	8.219718	2.029653
C	-3.198458	9.052746	2.596597
C	-2.911558	10.369342	3.025576
C	-5.440840	9.297566	3.508083
C	-5.127042	10.567484	3.948998
C	-3.860590	11.123855	3.682787
H	-1.214063	8.646228	1.805839
H	-1.914072	10.764062	2.853448
H	-6.412636	8.881012	3.738741
H	-5.859917	11.134612	4.514286
H	-3.625208	12.128450	4.020125

MH 8 (3rd)
246

C	-0.916691	1.754429	1.494940
C	-2.156147	1.091307	1.386697
C	-2.161215	-0.329711	1.146381
C	-0.969698	-0.927716	0.695992
C	0.104424	-0.089130	0.238131
C	0.174287	1.241069	0.695816
C	-0.871293	-2.366719	0.622329
C	-3.259841	-1.213561	1.526918
C	-3.245500	-2.563728	1.061224
C	-2.048183	-3.101736	0.430782
C	-3.356933	1.873287	1.601005
C	-3.331540	2.939727	2.510499
C	-2.041255	3.530909	2.833277
C	-0.842598	2.913978	2.366907
C	1.328618	2.036452	0.315466
C	2.512911	1.358568	-0.019595
C	2.358513	0.107745	-0.740094
C	1.099186	-0.547823	-0.716724
C	-1.939615	-4.372708	-0.265366
C	-0.684773	-5.017581	-0.304543
C	0.412683	-4.461718	0.474720
C	0.361815	-3.094229	0.831164
C	1.453194	-2.541225	1.544742
C	2.545149	-3.310535	1.885358
C	2.585509	-4.689976	1.581000

C	1.503967	-5.238837	0.917286
C	1.328545	3.471690	0.106251
C	2.568461	4.150820	0.013130
C	3.789707	3.428322	0.344778
C	3.775377	2.023623	0.242307
C	0.147313	4.187319	-0.203243
C	0.193138	5.512384	-0.580896
C	1.424939	6.188926	-0.717021
C	2.582964	5.482431	-0.449042
C	-4.610015	3.369934	3.062731
C	-5.790051	3.123865	2.332468
C	-5.682368	2.478682	1.032495
C	-4.522862	1.715053	0.750341
C	-6.668200	2.613501	0.033483
C	-6.620499	1.912768	-1.157638
C	-5.503731	1.080040	-1.387392
C	-4.472555	1.005742	-0.474665
C	-4.301275	-0.820097	2.398245
C	-4.301509	-3.427267	1.441008
C	-1.928063	4.780158	3.492763
C	0.395402	3.413241	2.839075
C	3.350584	-0.348651	-1.642024
C	0.855373	-1.586771	-1.642147
H	1.412519	-1.504319	1.853452
H	3.369074	-2.857102	2.431259
C	3.784758	-5.528666	1.975779
H	1.492412	-6.297773	0.717512
C	5.030418	4.097160	0.608308
C	4.971885	1.286867	0.599426
H	-0.805390	3.675108	-0.170464
H	-0.732424	6.034923	-0.807804
C	1.462269	7.642126	-1.145966
H	3.537325	5.962534	-0.592420
H	-7.492580	3.282794	0.216699
C	-7.723792	2.022165	-2.191052
H	-5.434155	0.523700	-2.318698
H	-3.590767	0.427289	-0.716774
C	1.837130	-2.014524	-2.514134
C	3.101800	-1.399918	-2.501001
C	-5.333702	-2.993003	2.247474
C	-5.326509	-1.679334	2.741671
C	0.471065	4.575957	3.576945
C	-0.700801	5.304522	3.843672
C	6.212767	1.967340	0.704880
C	6.234907	3.389858	0.450375
H	-4.282987	0.167628	2.835449
H	-4.268510	-4.462373	1.127731
H	-2.827818	5.331482	3.728490
H	1.300593	2.864930	2.616387
H	4.300685	0.168330	-1.684437
H	-0.124999	-2.044815	-1.677279
C	5.160624	5.463223	1.106361
C	4.911758	-0.107190	0.921990
H	1.620890	-2.807301	-3.223556
H	3.871437	-1.721175	-3.195863

H	-6.121655	-3.682665	2.533514	C	8.545159	1.900329	1.691686
H	-6.104932	-1.343113	3.419483	H	-4.158788	-3.066239	-1.128730
H	1.434192	4.930935	3.930570	C	8.394955	-0.869772	1.945243
H	-0.648771	6.263610	4.349102	H	5.952560	-1.872267	1.485799
C	7.362316	1.254823	1.245156	H	-5.981597	-3.939678	-2.486694
C	7.372660	4.153961	-0.052723	C	-6.067156	-6.322201	-3.702687
C	-0.565509	-6.274546	-0.984825	C	9.615376	1.186333	2.189893
C	-3.009402	-4.907238	-1.088065	C	9.557762	-0.217702	2.290882
C	-2.919734	-6.229830	-1.596363	H	8.609859	2.978139	1.659273
C	-1.729451	-6.994133	-1.300803	H	8.304976	-1.945173	2.071359
C	0.684292	-6.860557	-1.458938	H	-6.940215	-5.693493	-3.854031
C	-4.123645	-4.092203	-1.464479	H	10.502699	1.717072	2.520667
C	-3.918187	-6.687459	-2.552934	H	10.408493	-0.773694	2.672291
C	-1.631453	-8.447857	-1.215759	C	6.368873	6.176872	0.882304
C	3.819663	-6.929953	1.358913	C	7.375413	5.561537	0.025764
C	5.111115	-7.700000	1.638045	C	4.186432	6.014333	1.968516
C	5.097733	-9.088421	0.993034	C	8.392501	3.493220	-0.805950
C	-8.975929	2.768381	-1.721949	C	6.565190	7.399544	1.562231
C	-10.128295	2.737788	-2.726998	C	8.337446	6.316238	-0.741671
C	-11.359256	3.484864	-2.207063	C	9.386102	4.195364	-1.421365
C	2.824720	8.323983	-0.988960	C	9.386392	5.618615	-1.421046
C	2.803302	9.825843	-1.276742	H	8.361545	2.415185	-0.891096
C	4.184896	10.461281	-1.100005	C	8.253820	7.720774	-0.942800
H	3.834668	-5.606649	3.071389	H	10.160941	3.674215	-1.976219
H	4.693192	-4.984050	1.681743	C	10.361979	6.344773	-2.145360
H	0.714886	8.191582	-0.556965	C	5.590448	7.928985	2.389958
H	1.127644	7.725520	-2.190042	C	4.387486	7.233645	2.588006
H	-8.005477	1.005599	-2.499447	H	3.279147	5.456297	2.162317
H	-7.328061	2.501011	-3.098249	H	7.518506	7.904600	1.475658
H	3.680791	-6.849995	0.273859	H	5.774503	8.862681	2.912098
H	2.971301	-7.518483	1.731731	H	3.629648	7.630331	3.256378
H	5.262148	-7.794222	2.721692	C	9.203865	8.401358	-1.675525
H	5.966695	-7.125126	1.258845	C	10.288694	7.715440	-2.258839
H	4.278773	-9.697659	1.392258	H	7.404471	8.264950	-0.552827
H	6.033314	-9.627945	1.170582	H	11.159942	5.792162	-2.633589
H	4.950433	-9.012463	-0.090294	H	9.103282	9.472995	-1.817688
H	-8.723100	3.814719	-1.506252	H	11.038899	8.261123	-2.822480
H	-9.320271	2.336780	-0.774412	C	0.764367	-8.264483	-1.662511
H	-10.394203	1.694391	-2.943420	C	-0.370710	-9.078502	-1.243776
H	-9.802890	3.174314	-3.680685	C	1.743243	-6.037344	-1.903284
H	-11.698864	3.059902	-1.255680	C	-2.793748	-9.218463	-0.901702
H	-12.192169	3.437806	-2.915558	C	1.894510	-8.783959	-2.332340
H	-11.129926	4.541789	-2.029574	C	-0.257872	-10.462230	-0.849416
H	3.555314	7.848363	-1.656155	C	-3.765889	-7.851484	-3.351490
H	3.194698	8.161705	0.031018	C	-4.742756	-8.254282	-4.238445
H	2.083585	10.314637	-0.606508	C	-5.923771	-7.502703	-4.396998
H	2.439707	10.003859	-2.297563	H	-2.858310	-8.432598	-3.281834
H	4.562148	10.296182	-0.084333	H	-4.588328	-9.151517	-4.829690
H	4.161815	11.540484	-1.280985	H	-6.693486	-7.832758	-5.087605
H	4.910271	10.020765	-1.793571	C	2.855019	-6.577772	-2.522186
C	7.275424	-0.155346	1.455311	C	2.929118	-7.961790	-2.744398
C	6.023910	-0.809583	1.270816	H	1.665511	-4.964319	-1.779639
C	-5.136872	-4.574342	-2.234732	H	1.925526	-9.837861	-2.576925
C	-5.066721	-5.876744	-2.805459	H	3.649665	-5.924119	-2.868069
H	3.960239	-0.616845	0.886888	H	3.777637	-8.385876	-3.272140

C	-1.450521	-11.217529	-0.612748
C	-2.710844	-10.557728	-0.659243
C	0.982495	-11.105671	-0.589435
C	-1.359826	-12.586166	-0.262886
C	1.041821	-12.434497	-0.224399
C	-0.137107	-13.195668	-0.088400
H	-3.751673	-8.717948	-0.849382
H	-3.607028	-11.129724	-0.436791
H	1.898917	-10.533077	-0.633799
H	-2.280701	-13.142090	-0.109859
H	2.005857	-12.891656	-0.023442
H	-0.080046	-14.244244	0.186361
C	-4.753532	3.864815	4.420624
C	-7.037137	3.633079	2.831653
C	-7.018310	4.606830	3.843914
C	-5.947644	4.523177	4.811010
C	-3.745673	3.609371	5.402930
C	-6.131828	4.885875	6.209634
C	-5.044503	4.719321	7.121511
C	-3.859256	4.065259	6.680040
H	-2.877223	3.032198	5.120370
H	-3.071336	3.871547	7.402046
C	-8.366486	3.181344	2.431954
C	-8.045938	5.638251	3.770235
C	-9.475832	4.058247	2.577832
C	-9.211034	5.426928	3.005171
C	-8.597417	1.829003	2.095052
C	-9.878997	1.353093	1.889155
C	-10.775535	3.535987	2.394595
C	-10.977929	2.212637	2.042843
C	-7.375451	5.291728	6.761375
C	-5.190424	5.099100	8.477497
C	-7.783924	6.936939	4.306719
C	-10.061306	6.546376	2.680192
H	-7.754166	1.152740	2.028613
H	-10.032816	0.306323	1.646571
H	-11.631374	4.168982	2.590321
H	-11.988462	1.833686	1.926562
C	-8.653338	7.969765	4.114558
C	-9.804197	7.815250	3.291076
H	-6.871401	7.093498	4.867318
C	-11.097690	6.488540	1.709409
H	-8.445674	8.946577	4.541787
C	-10.651048	8.914900	3.012929
C	-6.391412	5.571311	8.957987
C	-7.500196	5.636130	8.091549
H	-8.255282	5.318914	6.136108
H	-4.337209	4.981267	9.139712
H	-6.494555	5.854964	10.000697
H	-8.468644	5.944135	8.473311
C	-11.892493	7.582870	1.438773
C	-11.690983	8.802485	2.116833
H	-11.240227	5.582240	1.136557
H	-10.447072	9.861397	3.505914
H	-12.668811	7.506899	0.683594
H	-12.329763	9.654602	1.906792

MH 8 (4th)

246

C	-0.600007	2.395561	0.953275
C	-1.890168	1.841000	0.814890
C	-1.981951	0.406598	0.742598
C	-0.828861	-0.324516	0.390343
C	0.333640	0.383163	-0.074666
C	0.490304	1.732943	0.295466
C	-0.862723	-1.768943	0.429298
C	-3.151031	-0.341690	1.184136
C	-3.250146	-1.722549	0.845791
C	-2.099931	-2.410127	0.276997
C	-2.998120	2.778558	0.760569
C	-2.844395	4.058741	1.312510
C	-1.634840	4.343586	2.070075
C	-0.482427	3.553720	1.820079
C	1.734686	2.408124	-0.011094
C	2.872692	1.618379	-0.241402
C	2.648345	0.354356	-0.924274
C	1.343138	-0.208276	-0.934381
C	-2.102268	-3.734983	-0.318387
C	-0.910877	-4.490965	-0.291977
C	0.223329	-3.979513	0.465139
C	0.292359	-2.590354	0.722505
C	1.413907	-2.089814	1.428294
C	2.423122	-2.928111	1.851048
C	2.345159	-4.323085	1.639428
C	1.231608	-4.817872	0.986250
C	1.873190	3.835778	-0.219684
C	3.170361	4.404799	-0.208222
C	4.295719	3.575643	0.204676
C	4.166904	2.174928	0.101468
C	0.787615	4.647990	-0.631789
C	0.974693	5.965578	-0.991384
C	2.266147	6.537501	-1.007903
C	3.333736	5.733716	-0.653858
C	-3.927982	5.010240	1.214020
C	-5.227258	4.513815	1.009268
C	-5.337808	3.323474	0.188617
C	-4.196534	2.502260	-0.010563
C	-6.496064	3.087606	-0.591530
C	-6.571920	2.058151	-1.509106
C	-5.423345	1.258520	-1.706401
C	-4.267645	1.487752	-0.993654
C	-4.160033	0.231256	1.989707
C	-4.382414	-2.449633	1.286815
C	-1.573859	5.340424	3.068892
C	0.698316	3.811701	2.552738
C	3.643911	-0.219049	-1.751763
C	1.070793	-1.285718	-1.806043
H	1.460805	-1.034970	1.667106
H	3.271693	-2.513331	2.389850
C	3.455278	-5.235809	2.121008

H	1.127614	-5.882969	0.858944	C	3.864098	8.541661	-1.156535
C	5.570438	4.135301	0.549198	C	3.996145	10.040978	-1.427324
C	5.271152	1.342667	0.541663	C	5.414642	10.547878	-1.153960
H	-0.201637	4.215203	-0.703162	H	3.473967	-5.244124	3.220331
H	0.118924	6.559533	-1.303021	H	4.415243	-4.794414	1.817695
C	2.460347	7.985345	-1.411857	H	1.727525	8.593877	-0.863105
H	4.333093	6.131879	-0.716250	H	2.202615	8.108219	-2.473595
C	-3.760621	6.449032	1.095223	H	-8.049182	0.709159	-2.234214
C	-6.323414	5.317377	1.521134	H	-7.616087	1.955165	-3.379323
H	-7.333838	3.758142	-0.472861	H	3.270187	-6.654484	0.506402
C	-7.825231	1.782362	-2.313862	H	2.472644	-7.158509	1.984652
H	-5.445142	0.468108	-2.452467	H	4.707995	-7.565040	3.053702
H	-3.390241	0.889643	-1.199725	H	5.500702	-7.062059	1.569620
C	2.059554	-1.830807	-2.601067	H	3.587805	-9.461876	1.827335
C	3.360957	-1.299481	-2.563038	H	5.345928	-9.561113	1.649760
C	-5.378573	-1.847576	2.028833	H	4.349505	-8.941592	0.321447
C	-5.261951	-0.496055	2.393151	H	-8.887918	3.651244	-2.071125
C	0.742229	4.815578	3.500266	H	-9.230121	2.467344	-0.824073
C	-0.404497	5.584561	3.763389	H	-10.512453	1.090816	-2.482663
C	-4.906791	7.272997	1.110928	H	-10.170168	2.272593	-3.734445
C	-6.125698	6.700578	1.665399	H	-11.739578	2.859820	-1.150236
C	6.558101	1.914286	0.717699	H	-12.458163	2.642126	-2.754179
C	6.717372	3.327935	0.461649	H	-11.415796	4.034322	-2.427923
H	-4.054894	1.257122	2.314697	H	4.592062	8.005869	-1.779514
H	-4.441192	-3.508638	1.071696	H	4.148688	8.341102	-0.116250
H	-2.464274	5.913867	3.294838	H	3.280856	10.588701	-0.799135
H	1.565226	3.182528	2.395037	H	3.717703	10.257140	-2.467368
H	4.630131	0.227130	-1.772852	H	5.708232	10.343375	-0.118073
H	0.062782	-1.677459	-1.861216	H	5.500458	11.625756	-1.323437
C	5.787011	5.484873	1.061226	H	6.141222	10.047614	-1.804384
C	5.069185	-0.034251	0.878942	C	7.384973	-0.281930	1.549151
C	-2.502159	7.007181	0.700720	C	6.092541	-0.825730	1.301340
H	1.822474	-2.650911	-3.271797	C	-5.294115	-3.792210	-2.306979
H	4.136777	-1.711525	-3.200729	C	-5.334521	-5.131779	-2.787460
H	-6.228381	-2.433513	2.364829	C	-2.401005	8.305239	0.295048
H	-6.018446	-0.028637	3.015949	C	-3.559615	9.119701	0.147148
H	1.653838	4.982810	4.065750	H	4.080064	-0.459307	0.796982
H	-0.382106	6.352638	4.529946	H	-1.622662	6.379496	0.691633
C	-4.834276	8.583998	0.506487	C	-5.986584	9.336150	0.155166
C	7.608014	1.111927	1.330285	C	8.816315	1.657214	1.838293
C	7.942943	3.991453	0.027607	H	-4.196200	-2.302144	-1.294571
C	-0.900033	-5.799512	-0.878501	C	8.407332	-1.084903	2.109454
C	-3.211056	-4.229224	-1.114088	H	5.913942	-1.873729	1.525904
C	-3.235709	-5.586888	-1.528517	H	-6.078657	-3.105306	-2.611480
C	-2.121719	-6.431710	-1.162708	C	-6.363546	-5.548952	-3.665691
C	0.296315	-6.528939	-1.287265	H	-1.435839	8.709902	0.002623
C	-4.247378	-3.348034	-1.559104	C	-3.476764	10.412904	-0.424305
C	-4.263490	-6.020166	-2.465250	C	9.789323	0.859179	2.403714
C	-2.157953	-7.878658	-0.974815	C	9.602104	-0.532764	2.514666
C	3.378033	-6.673051	1.597801	C	-4.613409	11.140790	-0.697674
C	4.589281	-7.532477	1.962452	C	-5.880035	10.580257	-0.427906
C	4.466122	-8.955395	1.411075	H	-6.966934	8.915881	0.336826
C	-9.063881	2.580768	-1.902638	H	8.978227	2.724460	1.800316
C	-10.328990	2.160157	-2.653603	H	8.215135	-2.146168	2.240763
C	-11.556636	2.966888	-2.225356	H	-7.176893	-4.858487	-3.871025

H	-2.494705	10.808350	-0.668692
H	10.700072	1.314729	2.779796
H	10.377443	-1.155729	2.949515
H	-4.539311	12.127084	-1.144993
H	-6.779884	11.127763	-0.690514
C	-7.586564	4.740617	1.951390
C	-7.062076	7.479142	2.456835
C	-8.646835	5.605733	2.358679
C	-8.319775	6.945311	2.817164
C	-9.168998	7.696547	3.730692
C	-7.836529	3.345131	1.969439
C	-6.678541	8.766456	2.951464
C	-8.805637	9.038049	4.073536
C	-7.537617	9.539748	3.667881
C	-9.964147	5.081310	2.331279
H	-5.684363	9.131844	2.740952
H	-7.237561	10.529084	4.001001
C	-10.202684	3.729420	2.182371
C	-9.115285	2.842336	2.097837
H	-7.012851	2.658408	1.837996
H	-10.800686	5.762089	2.409674
H	-11.221861	3.356509	2.160037
H	-9.277715	1.769186	2.088596
C	7.061772	6.094025	0.908574
C	8.059993	5.394107	0.109053
C	4.815395	6.114683	1.870848
C	8.945063	3.244453	-0.666396
C	7.320844	7.296698	1.602877
C	9.124687	6.062598	-0.600241
C	10.027629	3.858610	-1.223120
C	10.147716	5.276831	-1.220432
H	8.828400	2.172424	-0.754078
C	9.172068	7.468737	-0.802018
H	10.786404	3.272354	-1.733389
C	11.220727	5.915996	-1.886637
C	6.348650	7.905912	2.377111
C	5.082986	7.313052	2.506026
H	3.856318	5.632574	2.014296
H	8.316264	7.720469	1.571904
H	6.580413	8.821248	2.912599
H	4.325469	7.771113	3.134215
C	10.216368	8.064540	-1.478082
C	11.270316	7.287663	-2.000866
H	8.351351	8.083947	-0.459128
H	11.995492	5.296645	-2.330052
H	10.215431	9.140536	-1.622870
H	12.094757	7.766432	-2.519837
C	0.249316	-7.945247	-1.391797
C	-0.960165	-8.621494	-0.937847
C	1.432654	-5.839363	-1.766645
C	-3.389151	-8.516721	-0.627581
C	1.334752	-8.611318	-2.003283
C	-0.978843	-9.978778	-0.447195
C	-4.208110	-7.245171	-3.180863
C	-5.209899	-7.621243	-4.051615
C	-6.319127	-6.782301	-4.276545
H	-3.355714	-7.897102	-3.059455
H	-5.130492	-8.566966	-4.578628
H	-7.109648	-7.090648	-4.953547
C	2.497765	-6.521074	-2.325137
C	2.446162	-7.918162	-2.450854
H	1.453110	-4.757717	-1.717991
H	1.271069	-9.678103	-2.174578
H	3.354050	-5.968732	-2.699387
H	3.258416	-8.453935	-2.932030
C	-2.238155	-10.604368	-0.179585
C	-3.432030	-9.837695	-0.292367
C	0.194340	-10.712393	-0.122248
C	-2.277192	-11.947727	0.264958
C	0.127601	-12.012293	0.334260
C	-1.117525	-12.652001	0.501926
H	-4.297657	-7.928999	-0.625484
H	-4.379176	-10.308905	-0.046018
H	1.159891	-10.229975	-0.190191
H	-3.246794	-12.405669	0.440027
H	1.043435	-12.539809	0.582696
H	-1.159866	-13.679608	0.849043
C	-10.298980	7.159603	4.405706
C	-9.647721	9.818563	4.900498
C	-11.091088	7.931399	5.232147
C	-10.791628	9.288620	5.454412
H	-10.526501	6.108327	4.318139
H	-9.351835	10.841706	5.115132
H	-11.940604	7.475858	5.731716
H	-11.428319	9.893801	6.092063

MH 8 (5th)

246

C	-1.074489	2.481856	1.362743
C	-2.359272	1.897299	1.343535
C	-2.458942	0.461332	1.335183
C	-1.340786	-0.291722	0.930528
C	-0.039471	0.313225	0.941918
C	0.063035	1.715731	0.933948
C	-1.570715	-1.629199	0.425351
C	-3.565305	-0.288954	1.917762
C	-3.632785	-1.700956	1.735602
C	-2.720455	-2.335260	0.792562
C	-3.496540	2.797310	1.213091
C	-3.352292	4.146862	1.564259
C	-2.116455	4.565544	2.206584
C	-0.952669	3.778445	2.011782
C	1.275087	2.298547	0.379978
C	2.436154	1.517606	0.286156
C	2.435826	0.199066	0.903168
C	1.191050	-0.436135	1.148970
C	-2.859606	-3.708680	0.345540
C	-1.687663	-4.400073	-0.016474
C	-0.632869	-3.634410	-0.648043
C	-0.668447	-2.220599	-0.544258

C	1.281415	3.614648	-0.239487	C	-5.316556	-3.653045	-0.113590
C	2.531771	4.251469	-0.458041	C	-5.225214	-6.374180	-0.856215
C	3.753000	3.484005	-0.318845	C	-2.923575	-7.919869	0.586801
C	3.628311	2.084662	-0.307719	C	4.629204	1.333224	-1.044703
C	0.114120	4.285102	-0.676430	C	5.096525	4.026707	-0.301822
C	0.166600	5.561829	-1.191114	C	6.146213	3.240481	-0.802656
C	1.398600	6.239363	-1.333378	C	5.814669	1.982955	-1.456838
C	2.554039	5.558618	-1.002845	C	4.323350	0.034367	-1.564273
C	-4.462250	5.056824	1.380514	C	5.414235	5.268104	0.388590
C	-5.755574	4.511611	1.333760	C	7.513174	3.698136	-0.649955
C	-5.891301	3.191894	0.752253	C	6.570181	1.421628	-2.559171
C	-4.734936	2.387073	0.569224	C	-9.791953	1.895123	-0.629693
C	-7.117264	2.779453	0.176327	C	-11.103012	1.287235	-1.133250
C	-7.232180	1.617426	-0.561004	C	-12.332996	2.043085	-0.626479
C	-6.059173	0.866610	-0.799685	C	2.758047	8.386864	-1.750074
C	-4.848823	1.250312	-0.265524	C	2.676027	9.858872	-2.160448
C	-4.453530	0.318222	2.835637	C	4.023331	10.575203	-2.048491
C	-4.539809	-2.442630	2.526118	H	0.651787	8.230811	-1.323265
C	-2.042567	5.698230	3.047795	H	1.090236	7.657231	-2.914059
C	0.256311	4.187902	2.620260	H	-8.662106	0.082104	-0.914171
C	3.624045	-0.450835	1.309302	H	-8.513804	1.216991	-2.233763
H	-0.842173	3.783471	-0.616075	H	-9.746223	2.947631	-0.938285
H	-0.752511	6.048989	-1.506714	H	-9.790673	1.900614	0.468209
C	1.417717	7.657260	-1.864492	H	-11.159639	0.236828	-0.817583
H	3.514300	6.019288	-1.181290	H	-11.102721	1.275228	-2.231409
C	-4.334179	6.467887	1.058736	H	-12.363835	2.052313	0.468737
C	-6.847361	5.363346	1.761799	H	-13.261722	1.588128	-0.984768
H	-7.979353	3.416802	0.304852	H	-12.319758	3.085736	-0.962901
C	-8.553403	1.152788	-1.136826	H	3.508398	7.884525	-2.374336
H	-6.109289	-0.018230	-1.429265	H	3.125471	8.315901	-0.717950
H	-3.965392	0.672292	-0.499562	H	1.933857	10.368834	-1.531611
C	-5.404837	-1.822071	3.405967	H	2.302973	9.931257	-3.190830
C	-5.368245	-0.426174	3.553196	H	4.406589	10.531487	-1.022961
C	0.314225	5.327563	3.397711	H	3.944434	11.629123	-2.332909
C	-0.847870	6.084650	3.623246	H	4.771767	10.108426	-2.698417
C	-5.495896	7.271313	1.015644	C	6.271202	0.098109	-3.007583
C	-6.687827	6.754820	1.672771	C	5.144593	-0.579970	-2.462171
H	-4.380224	1.382410	3.013838	C	-4.211102	9.010820	-0.204581
H	-4.546327	-3.521540	2.444196	C	-3.034914	8.234334	-0.003223
H	-2.943190	6.266515	3.243570	C	-6.444094	-4.254274	-0.588353
H	1.138954	3.571823	2.509241	C	-6.427233	-5.615535	-1.005609
H	4.571664	0.054798	1.175323	H	-2.215611	6.372927	0.570535
C	-3.102411	6.989102	0.547102	C	-6.638767	9.170124	-0.194127
C	-8.013424	4.830766	2.450618	H	-5.317888	-2.588841	0.077325
H	-6.089808	-2.418082	4.000702	C	-5.166311	-7.635966	-1.505849
H	-6.025749	0.067426	4.262242	H	3.407802	-0.453303	-1.262347
H	1.251050	5.611326	3.867358	C	7.529167	2.158064	-3.304382
H	-0.815326	6.957236	4.267911	C	7.028568	-0.482784	-4.053499
C	-5.467079	8.497587	0.244512	H	4.897226	-1.566182	-2.841845
C	-7.701068	7.625149	2.235828	C	-4.163352	10.227946	-0.926882
C	-1.675335	-5.831464	0.200659	H	-2.087380	8.614243	-0.375818
C	-4.113341	-4.397523	0.102306	H	-7.350817	-3.674523	-0.736526
C	-4.099973	-5.786826	-0.159422	C	-7.548613	-6.191021	-1.651454
C	-2.897583	-6.521380	0.207570	C	6.726215	5.823584	0.279702
C	-0.467452	-6.527431	0.620016	C	7.774442	5.054651	-0.369163

C	4.484064	5.896011	1.253010
C	8.616717	2.786380	-0.715563
C	7.012898	7.005434	1.011174
C	9.112307	5.555907	-0.622641
C	10.189546	4.624055	-0.744558
C	9.904786	3.226800	-0.695107
C	8.229217	1.582581	-4.343365
C	8.005945	0.237311	-4.703509
H	7.699540	3.199134	-3.068141
H	6.798447	-1.501706	-4.352854
C	9.403309	6.925018	-0.849742
C	11.505913	5.096454	-0.951188
H	8.950652	2.177269	-4.894978
H	8.575157	-0.212370	-5.511063
C	6.061995	7.629949	1.791934
C	4.783206	7.065737	1.919609
C	11.762557	6.442496	-1.107447
C	10.693391	7.358184	-1.088357
H	3.515976	5.441453	1.406270
H	8.022387	7.389095	1.021707
H	8.591520	7.639565	-0.894307
H	12.311844	4.370574	-1.014239
H	6.325054	8.524307	2.348199
H	4.043736	7.519475	2.572077
H	12.775999	6.791503	-1.278834
H	10.878654	8.410711	-1.279136
C	-9.114632	5.691108	2.748632
C	-8.986696	7.119642	2.516861
C	-8.049797	3.496811	2.925938
C	-9.146037	2.990009	3.591802
C	-10.240675	3.825538	3.863335
C	-10.208386	5.148180	3.471786
C	-7.409616	8.986347	2.575973
C	-8.385857	9.840211	2.989941
C	-9.754210	9.436660	2.965935
C	-10.069444	8.070772	2.685576
C	-5.318848	10.893253	-1.271156
C	-6.567696	10.335339	-0.927279
H	-7.608369	8.748195	0.030838
H	-3.193008	10.609431	-1.232606
H	-7.190581	2.859525	2.774252
H	-9.138778	1.961713	3.939596
H	-11.089089	3.451919	4.428232
H	-11.006209	5.801295	3.793273
C	-10.796497	10.369023	3.173372
C	-11.435458	7.740790	2.496133
H	-5.272546	11.820155	-1.834268
H	-7.483803	10.819068	-1.251552
C	-12.434636	8.679617	2.666784
C	-12.118627	9.999417	3.041260
H	-10.532135	11.395646	3.411200
H	-11.698930	6.747967	2.154946
H	-13.468930	8.399011	2.493480
H	-12.909464	10.727889	3.190105
C	-0.477486	-7.950983	0.739781
C	-1.730488	-8.671586	0.591750
C	-4.132605	-8.550930	1.027221
C	-4.188595	-9.889074	1.271336
C	-3.073483	-10.725123	0.966156
C	-1.837537	-10.118604	0.581519
C	-6.261014	-8.160409	-2.158904
C	-7.478190	-7.449260	-2.206509
H	-4.236754	-8.188075	-1.508345
H	-8.458981	-5.602872	-1.727733
C	-3.183335	-12.134155	1.000152
C	-0.805396	-10.974436	0.119895
H	-6.178635	-9.124830	-2.650333
H	-8.340051	-7.877397	-2.708775
C	0.695036	-5.823464	1.022230
C	0.695003	-8.587728	1.224534
C	1.831863	-6.476619	1.449165
C	1.832065	-7.876726	1.549141
H	0.692612	-4.743735	1.008882
H	0.677485	-9.650223	1.418042
H	2.704478	-5.901757	1.744264
H	2.703325	-8.399805	1.931006
C	-0.950507	-12.348380	0.126845
C	-2.136985	-12.939244	0.601331
H	-4.124562	-12.571820	1.321014
H	0.093928	-10.542290	-0.299638
H	-0.148573	-12.973908	-0.252836
H	-2.238231	-14.019831	0.619635
C	1.186049	-1.711122	1.761978
C	2.363189	-2.347211	2.101695
C	3.595377	-1.709456	1.876937
C	0.173278	-1.459152	-1.384183
C	0.337272	-4.231932	-1.488939
C	1.241659	-3.472912	-2.208834
C	1.113751	-2.066891	-2.185372
H	0.240345	-2.177324	2.004801
H	2.328418	-3.319186	2.584412
H	4.521887	-2.187836	2.178151
H	0.084003	-0.380243	-1.390225
H	0.372676	-5.311607	-1.567348
C	2.405477	-4.101906	-2.929430
H	1.765964	-1.460525	-2.807870
C	3.647195	-4.116144	-2.015627
C	4.935321	-4.530069	-2.729224
C	6.164423	-4.431323	-1.823157
H	2.634424	-3.533436	-3.839730
H	2.164504	-5.124389	-3.241599
H	3.453894	-4.785004	-1.166849
H	3.777456	-3.117644	-1.579341
H	5.079203	-3.890910	-3.612051
H	4.831896	-5.553367	-3.113162
H	6.306806	-3.403465	-1.471499
H	7.076512	-4.736481	-2.345564
H	6.054580	-5.070794	-0.940358
H	-5.006067	-7.933383	1.189552
H	-5.101129	-10.344169	1.644567

H	-6.382335	9.321897	2.521520
H	-8.141883	10.856445	3.284638
H	8.406301	1.725754	-0.757093
H	10.729800	2.520838	-0.702651

MH 9 (1st)

264

C	-1.076422	2.467639	1.438125
C	-2.363999	1.888715	1.421174
C	-2.469991	0.453112	1.397706
C	-1.357015	-0.299856	0.978652
C	-0.053226	0.300145	0.985845
C	0.055113	1.702365	0.991701
C	-1.594194	-1.631873	0.463465
C	-3.576615	-0.298885	1.977551
C	-3.649197	-1.709530	1.784211
C	-2.742046	-2.339418	0.833005
C	-3.497675	2.795560	1.307969
C	-3.345187	4.140010	1.674708
C	-2.103306	4.545505	2.313976
C	-0.944259	3.756092	2.100921
C	1.264337	2.286338	0.432455
C	2.419512	1.499892	0.315418
C	2.420382	0.174570	0.917600
C	1.175682	-0.456897	1.172437
C	-2.880702	-3.712004	0.383043
C	-1.708929	-4.401720	0.012279
C	-0.663850	-3.629683	-0.632336
C	-0.700581	-2.216718	-0.518552
C	1.272214	3.609362	-0.173652
C	2.523779	4.239930	-0.404804
C	3.744505	3.465024	-0.282442
C	3.608188	2.065783	-0.285658
C	0.103340	4.289582	-0.591132
C	0.155651	5.568322	-1.101448
C	1.389956	6.236396	-1.262315
C	2.545069	5.545984	-0.950815
C	-4.452338	5.057104	1.511954
C	-5.749811	4.519119	1.465657
C	-5.892236	3.209877	0.857923
C	-4.741126	2.400257	0.663374
C	-7.116908	2.817852	0.266736
C	-7.239859	1.667549	-0.487635
C	-6.073639	0.908318	-0.730180
C	-4.862015	1.275689	-0.186860
C	-4.459701	0.303509	2.903587
C	-4.555425	-2.453941	2.573076
C	-2.018240	5.666568	3.169450
C	0.271167	4.153428	2.704501
C	3.610095	-0.486046	1.301405
H	-0.854980	3.793329	-0.520607
H	-0.765100	6.062362	-1.401249
C	1.415855	7.655620	-1.790309
H	3.504133	5.999472	-1.148844
C	-4.320657	6.472465	1.220235
C	-6.835970	5.365107	1.908688
H	-7.973003	3.462099	0.395077
C	-8.565330	1.227207	-1.073479
H	-6.128633	0.032148	-1.371346
H	-3.982343	0.694388	-0.426755
C	-5.415081	-1.837969	3.461226
C	-5.373991	-0.443430	3.618941
C	0.339602	5.282640	3.496195
C	-0.817884	6.040691	3.741106
C	-5.473724	7.288151	1.227820
C	-6.660423	6.761902	1.885295
H	-4.382371	1.365960	3.089927
H	-4.564638	-3.532109	2.484262
H	-2.915348	6.234746	3.380676
H	1.150497	3.535564	2.578150
H	4.558346	0.016686	1.161780
C	-3.093036	7.000116	0.702222
C	-8.039450	4.826235	2.518260
H	-6.099117	-2.436688	4.054364
H	-6.027249	0.046571	4.334357
H	1.281087	5.557064	3.961990
H	-0.777089	6.904199	4.397461
C	-5.445563	8.552639	0.521097
C	-7.686743	7.614763	2.446878
C	-1.688129	-5.830533	0.224571
C	-4.132071	-4.406718	0.156395
C	-4.119640	-5.800807	-0.073853
C	-2.911009	-6.526154	0.287906
C	-0.463513	-6.540109	0.552681
C	-5.341536	-3.670008	-0.058352
C	-5.260799	-6.416340	-0.718221
C	-2.911460	-7.928596	0.645420
C	4.597015	1.314901	-1.035676
C	5.088228	3.999986	-0.280073
C	6.139954	3.196463	-0.761753
C	5.795356	1.950328	-1.429310
C	4.276404	0.026148	-1.573928
C	5.415391	5.270850	0.343594
C	7.503993	3.665227	-0.637464
C	6.569844	1.381770	-2.514539
C	-9.792323	1.988579	-0.565957
C	-11.115119	1.406685	-1.068718
C	-12.327446	2.187262	-0.556404
C	2.771686	8.361154	-1.711281
C	2.708111	9.837276	-2.108986
C	4.074256	10.522003	-2.030299
H	0.675494	8.239738	-1.225219
H	1.058236	7.664731	-2.829886
H	-8.692944	0.157362	-0.856758
H	-8.519827	1.296549	-2.169821
H	-9.728077	3.041038	-0.871533
H	-9.791753	1.991364	0.531776
H	-11.192005	0.357277	-0.754027
H	-11.118391	1.396215	-2.166867
H	-12.349292	2.201980	0.539042

H	-13.267376	1.748866	-0.906004
H	-12.296629	3.228224	-0.897035
H	3.494285	7.848710	-2.359692
H	3.167700	8.277321	-0.690946
H	1.996128	10.358049	-1.454631
H	2.307063	9.926590	-3.127426
H	4.488762	10.453861	-1.018270
H	4.011016	11.581461	-2.297518
H	4.790835	10.047642	-2.710028
C	6.257472	0.067052	-2.975338
C	5.102242	-0.591875	-2.463960
C	-4.192516	9.070263	0.073037
C	-3.023159	8.266565	0.204882
C	-6.475277	-4.288000	-0.493205
C	-6.466735	-5.665116	-0.860437
H	-2.213689	6.372861	0.682396
C	-6.612681	9.280213	0.174570
H	-5.341143	-2.601003	0.105070
C	-5.227955	-7.713549	-1.290666
H	3.348740	-0.448941	-1.288867
C	7.584987	2.089642	-3.207227
C	7.042012	-0.531744	-3.989735
H	4.845224	-1.569719	-2.858314
C	-4.138832	10.331023	-0.568114
H	-2.079544	8.652048	-0.171933
H	-7.386927	-3.715661	-0.640001
C	-7.609758	-6.270157	-1.435937
C	6.703315	5.843441	0.115443
C	7.731172	5.041190	-0.517150
C	4.527604	5.930522	1.226554
C	8.656839	2.771546	-0.550769
C	7.004720	7.087000	0.728794
C	9.010243	5.564523	-0.983886
C	10.115407	4.682725	-1.141268
C	9.963491	3.286563	-0.751889
C	8.318675	1.495914	-4.212078
C	8.071000	0.160357	-4.589193
C	11.069734	2.430940	-0.575994
C	10.903936	1.107838	-0.208657
C	9.614312	0.606057	0.019339
C	8.515011	1.427298	-0.143492
H	7.778196	3.123702	-2.957898
H	6.797784	-1.543485	-4.302067
C	9.142299	6.896177	-1.440108
C	11.314856	5.183004	-1.684813
H	9.088172	2.068253	-4.720489
H	8.665677	-0.304955	-5.369082
H	12.072776	2.822268	-0.700361
H	11.771397	0.469325	-0.074114
H	9.476177	-0.421836	0.339399
H	7.525952	1.039886	0.061512
C	6.089866	7.738639	1.530770
C	4.841684	7.149250	1.792054
C	11.433557	6.503909	-2.081259
C	10.331584	7.362643	-1.972593
H	3.581649	5.465610	1.467533
H	7.999795	7.497927	0.630072
H	8.278579	7.548348	-1.424183
H	12.153576	4.512282	-1.830421
H	6.359669	8.679336	2.000618
H	4.137814	7.630132	2.464061
H	12.366554	6.861137	-2.505657
H	10.397739	8.385599	-2.329640
C	-9.159183	5.688453	2.720765
C	-8.991471	7.117146	2.545391
C	-8.119703	3.491287	2.981231
C	-9.277406	2.986075	3.536250
C	-10.396805	3.820127	3.694759
C	-10.327687	5.145112	3.314843
C	-7.419319	8.944537	2.990587
C	-8.495489	9.839259	3.223928
C	-9.838134	9.443308	2.818534
C	-10.090889	8.075485	2.520638
C	-5.288530	11.045194	-0.823719
C	-6.538034	10.493990	-0.474482
H	-7.585105	8.867658	0.404824
H	-3.169836	10.712517	-0.878132
H	-7.250522	2.853079	2.901513
H	-9.307123	1.956936	3.880151
H	-11.299153	3.441090	4.164397
H	-11.156420	5.799644	3.546387
C	-6.125286	9.318663	3.413027
C	-8.230867	11.082554	3.833028
C	-10.888724	10.373589	2.695122
C	-11.374992	7.715923	2.050745
H	-5.237726	12.009318	-1.320000
H	-7.452333	11.022768	-0.724744
C	-5.889213	10.544653	4.005230
C	-6.948791	11.440619	4.208588
C	-12.389968	8.649887	1.936530
C	-12.150672	9.987941	2.277039
H	-5.306991	8.622609	3.285692
H	-9.050388	11.760798	4.040583
H	-10.698139	11.421255	2.896649
H	-11.551770	6.700391	1.720573
H	-4.885174	10.803338	4.326262
H	-6.770177	12.401458	4.681016
H	-13.360854	8.347060	1.557082
H	-12.939957	10.726962	2.181682
C	-0.472036	-7.967443	0.559509
C	-1.736714	-8.668422	0.461882
C	-4.049679	-8.600410	1.270057
C	-4.101982	-10.018030	1.298881
C	-3.058858	-10.773544	0.617204
C	-1.861323	-10.104812	0.240448
C	-6.346665	-8.272553	-1.870122
C	-7.561805	-7.559437	-1.918216
H	-4.301674	-8.271258	-1.291356
H	-8.521905	-5.684682	-1.511813
C	-5.040990	-7.873965	1.964838

C	-5.160857	-10.649518	1.982191
C	-3.204207	-12.139727	0.306218
C	-0.888249	-10.827792	-0.487147
H	-6.285303	-9.267577	-2.299469
H	-8.442572	-8.013167	-2.361818
C	0.718911	-5.867511	0.945911
C	0.727152	-8.645587	0.901269
C	1.875578	-6.554580	1.250463
C	1.881178	-7.959256	1.220258
H	0.716620	-4.789208	1.016698
H	0.717784	-9.723226	0.988916
H	2.765416	-6.006889	1.545643
H	2.775514	-8.509092	1.496523
C	-6.071465	-8.514783	2.625851
C	-6.140217	-9.915446	2.626256
C	-1.055583	-12.169802	-0.781253
C	-2.216414	-12.836919	-0.367741
H	-4.980249	-6.794405	1.994626
H	-5.193700	-11.731662	2.032588
H	-4.123771	-12.650148	0.567820
H	-0.019890	-10.307173	-0.869856
H	-6.815994	-7.930543	3.157275
H	-6.943071	-10.424125	3.150634
H	-0.298379	-12.694176	-1.355693
H	-2.357864	-13.887118	-0.602892
C	1.171692	-1.737875	1.772949
C	2.349639	-2.383649	2.090852
C	3.582237	-1.750186	1.856385
C	0.128778	-1.446726	-1.362619
C	0.292600	-4.217351	-1.494974
C	1.186560	-3.450922	-2.220701
C	1.059413	-2.045722	-2.181893
H	0.226563	-2.201405	2.023083
H	2.316420	-3.360382	2.564038
H	4.509953	-2.236468	2.140846
H	0.037555	-0.367937	-1.357754
H	0.326742	-5.295423	-1.588551
C	2.340101	-4.074390	-2.962467
H	1.702487	-1.433118	-2.807732
C	3.589148	-4.112058	-2.059133
C	4.869881	-4.517937	-2.790563
C	6.107440	-4.435048	-1.894333
H	2.564951	-3.491445	-3.864546
H	2.090266	-5.089865	-3.290281
H	3.399215	-4.796499	-1.222116
H	3.727398	-3.122735	-1.604890
H	5.007416	-3.865957	-3.664936
H	4.760890	-5.535136	-3.188893
H	6.256282	-3.412308	-1.530621
H	7.013875	-4.735910	-2.428953
H	6.003491	-5.085911	-1.019114

MH 9 (2nd)

264

C	-0.626048	2.413877	0.924939
C	-1.918248	1.862012	0.793627
C	-2.010650	0.426645	0.737726
C	-0.858832	-0.308495	0.389413
C	0.300389	0.394010	-0.091866
C	0.459862	1.746925	0.264881
C	-0.890950	-1.752461	0.450561
C	-3.177608	-0.316972	1.193153
C	-3.279574	-1.700888	0.868374
C	-2.128049	-2.396844	0.311969
C	-3.023096	2.804268	0.737137
C	-2.862876	4.084594	1.287434
C	-1.655391	4.355689	2.054856
C	-0.503937	3.567569	1.796749
C	1.703732	2.418950	-0.051480
C	2.839213	1.626084	-0.283549
C	2.611345	0.354232	-0.950990
C	1.305659	-0.207215	-0.949704
C	-2.131038	-3.731280	-0.258192
C	-0.939591	-4.484734	-0.231997
C	0.207124	-3.958512	0.492573
C	0.268762	-2.567045	0.741813
C	1.397257	-2.053993	1.427120
C	2.425755	-2.879788	1.825940
C	2.364711	-4.274737	1.607354
C	1.241133	-4.784586	0.983855
C	1.844082	3.847094	-0.250918
C	3.141265	4.417230	-0.231547
C	4.268628	3.580606	0.154345
C	4.135511	2.181814	0.046780
C	0.757392	4.666026	-0.646365
C	0.941091	5.993079	-0.969607
C	2.230193	6.571122	-0.962742
C	3.301022	5.760663	-0.635258
C	-3.937560	5.046547	1.181235
C	-5.239648	4.559645	0.969909
C	-5.370033	3.342685	0.192369
C	-4.231827	2.519903	-0.014765
C	-6.558999	3.059578	-0.520875
C	-6.661981	1.993403	-1.392890
C	-5.508251	1.210514	-1.622595
C	-4.326483	1.479416	-0.968246
C	-4.182875	0.265119	1.996513
C	-4.415913	-2.419356	1.312418
C	-1.595500	5.334730	3.070964
C	0.677885	3.816531	2.530958
C	3.603821	-0.232361	-1.772568
C	1.029181	-1.295546	-1.806295
H	1.436655	-0.998577	1.664809
H	3.280216	-2.455559	2.347674
C	3.504723	-5.168971	2.051174
H	1.150603	-5.850927	0.851218
C	5.550766	4.135312	0.487142
C	5.243346	1.352042	0.483231
H	-0.230818	4.232742	-0.728787
H	0.084363	6.593454	-1.265605

C	2.414954	8.033348	-1.314546	H	1.643044	8.610654	-0.786411
H	4.299631	6.164917	-0.679546	H	2.208652	8.183879	-2.383998
C	-3.756306	6.484671	1.102695	H	-8.122411	0.581813	-2.027401
C	-6.337425	5.369332	1.450911	H	-7.832423	1.856165	-3.187126
H	-7.403236	3.718013	-0.385170	H	3.357901	-6.520675	0.375527
C	-7.954278	1.664550	-2.111275	H	2.597725	-7.122486	1.836381
H	-5.550000	0.395538	-2.340858	H	4.858185	-7.477771	2.870648
H	-3.450197	0.885616	-1.190769	H	5.614861	-6.882854	1.402020
C	2.015082	-1.852977	-2.596395	H	3.807483	-9.370213	1.578433
C	3.317438	-1.323487	-2.568295	H	5.566367	-9.386890	1.384175
C	-5.410518	-1.807787	2.048980	H	4.533274	-8.759603	0.088597
C	-5.287431	-0.454937	2.405561	H	-9.085729	3.479929	-1.781773
C	0.721623	4.806552	3.492939	H	-9.265659	2.284627	-0.511428
C	-0.426780	5.568178	3.770184	H	-10.616293	0.845557	-2.056789
C	-4.890785	7.325450	1.140342	H	-10.425914	2.030737	-3.337504
C	-6.133459	6.750353	1.632588	H	-11.811071	2.572512	-0.640454
C	6.534231	1.921782	0.619098	H	-12.644097	2.307773	-2.180777
C	6.697133	3.332209	0.331771	H	-11.641722	3.747212	-1.947942
H	-4.073296	1.293080	2.313337	H	4.560831	8.117445	-1.582501
H	-4.479124	-3.479619	1.105355	H	4.035082	8.371183	0.070853
H	-2.486624	5.903389	3.305809	H	3.140634	10.625010	-0.560011
H	1.544961	3.190034	2.363648	H	3.655098	10.371583	-2.219578
H	4.590721	0.211721	-1.802031	H	5.544222	10.417690	0.213072
H	0.020567	-1.686980	-1.852628	H	5.349261	11.739916	-0.949971
C	5.771501	5.437989	1.098053	H	6.053927	10.200115	-1.463876
C	5.040098	-0.015050	0.858134	C	7.369881	-0.256467	1.482972
C	-2.482922	7.043706	0.756584	C	6.067265	-0.798614	1.285265
H	1.775051	-2.682105	-3.254844	C	-5.302478	-3.828644	-2.276198
H	4.091239	-1.745793	-3.201738	C	-5.355810	-5.188433	-2.697352
H	-6.263677	-2.387471	2.387351	C	-2.351374	8.364537	0.449134
H	-6.041542	0.019532	3.025989	C	-3.492016	9.212030	0.343802
H	1.633922	4.967041	4.059278	H	4.047455	-0.436455	0.800350
H	-0.405558	6.322770	4.550081	H	-1.615364	6.401445	0.714708
C	-4.785064	8.673673	0.620817	C	-5.908762	9.475047	0.294040
C	7.597641	1.127745	1.218329	C	8.840892	1.668889	1.631534
C	7.915745	4.017304	-0.088484	H	-4.204333	-2.313963	-1.296972
C	-0.935710	-5.805554	-0.795213	C	8.407770	-1.055324	2.018910
C	-3.240163	-4.242806	-1.042478	H	5.889531	-1.839622	1.541044
C	-3.272743	-5.613345	-1.405404	H	-6.076598	-3.146777	-2.616610
C	-2.165707	-6.457106	-1.004613	C	-6.392570	-5.636371	-3.550277
C	0.239413	-6.501078	-1.299352	H	-1.374036	8.770894	0.202933
C	-4.262804	-3.367500	-1.529162	C	-3.366190	10.547194	-0.109004
C	-4.302311	-6.076859	-2.325058	C	9.832652	0.874619	2.168544
C	-2.171450	-7.904049	-0.810921	C	9.630119	-0.508263	2.341228
C	3.478623	-6.583548	1.464274	C	-4.479687	11.322162	-0.347063
C	4.727726	-7.406722	1.782559	C	-5.762274	10.764011	-0.171906
C	4.659498	-8.810557	1.176048	H	-6.903317	9.062554	0.392253
C	-9.194184	2.402683	-1.600372	H	9.018776	2.729927	1.540109
C	-10.494304	1.920322	-2.246989	H	8.209712	-2.109439	2.192569
C	-11.718807	2.676708	-1.727344	H	-7.190031	-4.942276	-3.800852
C	3.792197	8.609378	-0.972433	H	-2.369426	10.940105	-0.289765
C	3.896248	10.121433	-1.177838	H	10.773000	1.326190	2.468613
C	5.287387	10.654275	-0.825756	H	10.419425	-1.128962	2.753720
H	3.522925	-5.226575	3.149108	H	-4.373339	12.343668	-0.698669
H	4.448775	-4.681349	1.770240	H	-6.644744	11.348254	-0.412916

C	-7.590229	4.787310	1.901249
C	-7.181124	7.548663	2.233729
C	-8.705332	5.645687	2.141689
C	-8.498765	7.079726	2.173122
C	-9.575298	8.063438	2.203580
C	-7.726906	3.405921	2.177284
C	-6.928939	8.784361	2.971837
C	-9.318944	9.371036	2.701628
C	-8.002043	9.670090	3.249510
C	-9.922902	5.061071	2.576920
C	-10.828788	7.806804	1.602051
C	-5.662535	9.060193	3.531425
C	-10.340060	10.339053	2.633565
C	-7.758925	10.811704	4.039703
C	-10.042735	3.700027	2.771686
C	-8.929772	2.864050	2.580982
H	-6.866138	2.760014	2.072823
H	-10.754019	5.701881	2.836624
H	-10.982860	3.287259	3.124163
H	-9.002158	1.799937	2.783013
C	-11.815039	8.776184	1.546178
C	-11.575584	10.049128	2.080872
H	-11.000486	6.850116	1.125730
H	-10.144799	11.344309	2.988372
H	-12.761667	8.554764	1.063295
H	-12.341789	10.816469	2.032571
C	7.074080	6.006733	1.061631
C	8.071495	5.376147	0.206173
C	4.785034	6.028002	1.922720
C	8.908097	3.345620	-0.926008
C	7.349194	7.108376	1.903602
C	9.209033	6.112058	-0.323134
C	10.151106	3.984056	-1.190063
C	10.304400	5.401762	-0.889962
C	8.677570	2.073305	-1.497143
C	9.251901	7.527593	-0.340179
C	11.158452	3.253395	-1.859022
C	11.456728	6.125360	-1.266965
C	6.366871	7.675713	2.694939
C	5.069607	7.138816	2.693181
H	3.803464	5.575316	1.978646
H	8.361173	7.486642	1.964336
H	6.611376	8.512525	3.341612
H	4.300248	7.563616	3.330389
C	10.376278	8.214274	-0.756420
C	11.507941	7.503934	-1.182488
H	8.372227	8.085868	-0.050168
H	12.315262	5.599642	-1.665694
H	10.375658	9.299751	-0.761672
H	12.405363	8.032703	-1.487944
C	0.173452	-7.905284	-1.510951
C	-0.982791	-8.621079	-0.985966
C	1.346744	-5.778794	-1.802977
C	-3.358259	-8.592156	-0.306469
C	1.194192	-8.517442	-2.273666
C	-0.930694	-10.037916	-0.661504
C	-4.283398	-7.351660	-2.944971
C	-5.295748	-7.761592	-3.787460
C	-6.379446	-6.909612	-4.075357
H	-3.452940	-8.018923	-2.770392
H	-5.246878	-8.747692	-4.238505
H	-7.178426	-7.243415	-4.730059
C	2.356230	-6.414821	-2.499289
C	2.273096	-7.794021	-2.749100
H	1.381360	-4.704926	-1.673160
H	1.105648	-9.564973	-2.530176
H	3.187534	-5.836091	-2.889858
H	3.034217	-8.288800	-3.344203
C	-2.140637	-10.754938	-0.442952
C	-3.383090	-10.013571	-0.269606
C	0.293546	-10.732819	-0.505672
C	-2.074772	-12.154545	-0.268573
C	0.330996	-12.097198	-0.291158
C	-0.866427	-12.823382	-0.216108
C	-4.495602	-7.892631	0.157462
C	-4.593974	-10.660489	0.064174
H	1.221526	-10.177731	-0.523450
H	-2.986656	-12.723599	-0.137787
H	1.285786	-12.599548	-0.171469
H	-0.848178	-13.899181	-0.072591
C	9.665795	1.395190	-2.181759
C	10.934293	1.974454	-2.329751
C	-5.719018	-9.947982	0.430475
C	-5.655296	-8.549675	0.515951
C	-5.447032	10.187692	4.300670
C	-6.501210	11.078545	4.549517
H	-4.850745	8.364017	3.368206
H	-8.578305	11.479150	4.280146
H	7.704608	1.615895	-1.393489
H	12.124951	3.708552	-2.035563
H	-4.459823	-6.815561	0.230739
H	-4.643883	-11.742143	0.061295
H	9.456918	0.415958	-2.600473
H	11.726687	1.438571	-2.842847
H	-6.634580	-10.472858	0.684623
H	-6.513002	-7.980865	0.860411
H	-4.464531	10.371332	4.723798
H	-6.339319	11.961333	5.160071

MH 9 (3rd)
264

C	-1.009338	2.595527	1.675703
C	-2.276895	1.977308	1.627511
C	-2.343468	0.542112	1.631105
C	-1.202520	-0.194113	1.259667
C	0.081338	0.442882	1.306075
C	0.156061	1.849169	1.290266
C	-1.400340	-1.534605	0.742003
C	-3.452956	-0.218054	2.190976
C	-3.508197	-1.623909	1.984946

C	-2.574927	-2.232208	1.046354	C	-3.186486	7.048754	0.790398
C	-3.436271	2.839266	1.456305	C	-8.085571	4.692815	2.503135
C	-3.346020	4.194216	1.801254	H	-6.011818	-2.392044	4.179262
C	-2.138783	4.655412	2.470087	H	-5.963937	0.087570	4.486462
C	-0.945764	3.902345	2.313743	H	1.149419	5.816360	4.212488
C	1.377666	2.445829	0.767187	H	-0.967304	7.098622	4.549203
C	2.555277	1.688471	0.734607	C	-5.596117	8.496840	0.518069
C	2.552945	0.408439	1.433752	C	-7.850453	7.492428	2.394907
C	1.317012	-0.270761	1.592194	C	-1.654797	-5.727429	0.246156
C	-2.752654	-3.570806	0.524265	C	-4.029236	-4.185058	0.213200
C	-1.602538	-4.286836	0.142873	C	-4.073362	-5.557941	-0.115100
C	-0.511856	-3.518295	-0.427062	C	-2.910945	-6.363816	0.227583
C	-0.483292	-2.113318	-0.225899	C	-0.474981	-6.520465	0.544433
C	1.381758	3.757909	0.139252	C	-5.195488	-3.377389	0.012152
C	2.626956	4.411388	-0.053554	C	-5.219166	-6.072395	-0.836035
C	3.861164	3.644206	0.063940	C	-2.990707	-7.785912	0.483351
C	3.757011	2.243070	0.134021	C	4.852986	1.436111	-0.355979
C	0.212962	4.406980	-0.325126	C	5.158240	4.236102	-0.135213
C	0.248807	5.688250	-0.830193	C	6.172877	3.478878	-0.748182
C	1.470190	6.388213	-0.935810	C	6.093011	2.039097	-0.679932
C	2.629659	5.722788	-0.587042	C	4.676024	0.044953	-0.667831
C	-4.485157	5.061375	1.590718	C	5.551329	5.522042	0.425490
C	-5.757690	4.468066	1.516802	C	7.173013	4.238648	-1.501946
C	-5.829635	3.144271	0.928166	C	7.249735	1.173280	-0.853354
C	-4.640745	2.381658	0.780641	C	-9.634891	1.714011	-0.558884
C	-7.020096	2.687247	0.313395	C	-10.916831	1.057827	-1.075768
C	-7.072534	1.517276	-0.418790	C	-12.176321	1.788222	-0.604928
C	-5.867685	0.807109	-0.618854	C	2.852093	8.497682	-1.458225
C	-4.688176	1.238995	-0.052989	C	2.794919	9.956984	-1.914629
C	-4.362436	0.371810	3.098795	C	4.171776	10.624812	-1.906277
C	-4.429359	-2.385192	2.738806	H	0.783365	8.402763	-0.860020
C	-2.121363	5.794986	3.304605	H	1.074263	7.819450	-2.481117
C	0.232783	4.353593	2.950690	H	-8.443072	-0.067230	-0.789516
C	3.713774	-0.151715	2.012243	H	-8.295089	1.053854	-2.121135
H	-0.732627	3.883332	-0.293795	H	-9.615061	2.761841	-0.885710
H	-0.671860	6.156259	-1.169588	H	-9.657786	1.739403	0.538292
C	1.485947	7.808427	-1.461955	H	-10.949194	0.012567	-0.740136
H	3.578366	6.204310	-0.759251	H	-10.896240	1.024664	-2.173279
C	-4.405413	6.476595	1.280834	H	-12.222064	1.824288	0.489281
C	-6.890062	5.273652	1.916832	H	-13.086165	1.297133	-0.963944
H	-7.907093	3.294687	0.407163	H	-12.188971	2.822250	-0.967135
C	-8.361896	1.003406	-1.024868	H	3.544348	7.948405	-2.109626
H	-5.866755	-0.082407	-1.243982	H	3.282864	8.450668	-0.449332
H	-3.777133	0.694558	-0.260859	H	2.109521	10.515297	-1.262907
C	-5.315709	-1.783563	3.610651	H	2.365213	10.007971	-2.923926
C	-5.288410	-0.389955	3.783370	H	4.609566	10.607356	-0.901900
C	0.234747	5.499481	3.721109	H	4.115799	11.668903	-2.229681
C	-0.955411	6.221453	3.910019	H	4.865249	10.103560	-2.575599
C	-5.591603	7.242561	1.243168	C	7.048441	-0.218227	-1.085257
C	-6.773347	6.676154	1.875622	C	5.717739	-0.735097	-1.066384
H	-4.299069	1.434148	3.293120	C	-4.353425	9.061026	0.098326
H	-4.430042	-3.462357	2.633016	C	-3.155448	8.310001	0.276155
H	-3.043968	6.335812	3.473381	C	-6.340703	-3.908498	-0.500540
H	1.136715	3.764861	2.866148	C	-6.383750	-5.255957	-0.962555
H	4.642826	0.401616	1.968974	H	-2.281337	6.459056	0.806534

C	-6.782308	9.169066	0.127350
H	-5.154587	-2.323153	0.247761
C	-5.226357	-7.326894	-1.497693
H	3.683154	-0.380303	-0.612735
H	5.554343	-1.776192	-1.321773
C	-4.334299	10.313912	-0.560016
H	-2.218375	8.730303	-0.079023
H	-7.218765	-3.282888	-0.635520
C	-7.532884	-5.763772	-1.614739
C	6.802697	6.089620	0.053130
C	7.474938	5.548068	-1.121352
C	4.845117	6.080255	1.516986
C	7.342128	7.111557	0.866037
C	6.635829	7.637047	1.933091
C	5.362244	7.136156	2.242426
H	3.902522	5.639582	1.814777
H	8.346522	7.464103	0.673101
H	7.080864	8.416166	2.543946
H	4.805558	7.536277	3.084039
C	-9.246407	5.508822	2.660534
C	-9.135020	6.940553	2.465890
C	-8.120917	3.363395	2.987267
C	-9.270880	2.817383	3.519302
C	-10.429240	3.604316	3.632517
C	-10.406643	4.924798	3.231806
C	-7.655409	8.840489	2.924094
C	-8.774900	9.691478	3.113046
C	-10.087526	9.231913	2.677576
C	-10.273204	7.850080	2.395376
C	-5.505169	10.974769	-0.859219
C	-6.740005	10.375750	-0.537626
H	-7.742721	8.718644	0.336120
H	-3.373646	10.732113	-0.847837
H	-7.223217	2.762269	2.942631
H	-9.265755	1.793673	3.880255
H	-11.327232	3.193983	4.083859
H	-11.268914	5.546311	3.429406
C	-6.390924	9.276256	3.375948
C	-8.580715	10.954114	3.708721
C	-11.173038	10.114002	2.510300
C	-11.527126	7.428330	1.896576
H	-5.480594	11.933201	-1.368352
H	-7.668079	10.861587	-0.821920
C	-6.224079	10.520114	3.954282
C	-7.326164	11.372674	4.113541
C	-12.577486	8.315773	1.739419
C	-12.405181	9.667937	2.064332
H	-5.540514	8.614112	3.282660
H	-9.433875	11.599568	3.882529
H	-11.033112	11.171869	2.699735
H	-11.651017	6.401195	1.578393
H	-5.241431	10.826597	4.298582
H	-7.202045	12.347395	4.574864
H	-13.523572	7.965588	1.338668
H	-13.222337	10.370675	1.935362
C	-0.555149	-7.942903	0.449273
C	-1.849843	-8.569978	0.274416
C	-4.177996	-8.440132	1.030489
C	-4.301286	-9.851697	0.956855
C	-3.278796	-10.609987	0.247528
C	-2.039822	-9.978893	-0.052500
C	-6.348980	-7.789862	-2.149789
C	-7.527716	-7.017424	-2.185217
H	-4.327340	-7.927363	-1.509342
H	-8.413888	-5.130942	-1.677472
C	-5.151186	-7.713975	1.750693
C	-5.408574	-10.474220	1.567824
C	-3.483499	-11.941532	-0.164094
C	-1.085099	-10.697749	-0.807963
H	-6.318549	-8.754608	-2.646301
H	-8.412582	-7.396370	-2.687041
C	0.730936	-5.938729	1.004656
C	0.598695	-8.704691	0.768331
C	1.842400	-6.705464	1.286514
C	1.777119	-8.102840	1.160343
H	0.780464	-4.868635	1.150675
H	0.532530	-9.783852	0.780212
H	2.751731	-6.227965	1.638006
H	2.634943	-8.716158	1.418241
C	-6.230018	-8.345691	2.339394
C	-6.367817	-9.737621	2.238738
C	-1.311059	-12.005201	-1.201614
C	-2.514080	-12.639298	-0.863633
H	-5.038274	-6.643578	1.858221
H	-5.496433	-11.554120	1.540209
H	-4.433922	-12.421123	0.038944
H	-0.182117	-10.196725	-1.132169
H	-6.959349	-7.762531	2.892643
H	-7.209005	-10.239826	2.706177
H	-0.565924	-12.525922	-1.794855
H	-2.701270	-13.661609	-1.176794
C	1.307799	-1.532570	2.231316
C	2.470042	-2.090344	2.726449
C	3.680723	-1.383362	2.636675
C	0.392029	-1.340544	-1.023395
C	0.393418	-4.096041	-1.351555
C	1.286029	-3.332895	-2.080846
C	1.256183	-1.929878	-1.919335
H	0.365294	-2.041987	2.382869
H	2.433291	-3.051525	3.230074
H	4.586323	-1.790764	3.074678
H	0.364634	-0.261386	-0.950317
H	0.354454	-5.164658	-1.522073
C	2.257779	-3.965745	-3.047229
H	1.902180	-1.303724	-2.528187
C	3.724834	-3.710749	-2.663958
C	4.722314	-4.368587	-3.620511
C	6.178883	-4.053110	-3.271744
H	2.085172	-3.572241	-4.058442
H	2.074487	-5.045063	-3.097045

H 3.895356 -4.071873 -1.641145
H 3.906291 -2.629068 -2.640192
H 4.511580 -4.037176 -4.646002
H 4.567128 -5.455473 -3.614022
H 6.374633 -2.977043 -3.332453
H 6.873803 -4.555639 -3.951759
H 6.418467 -4.377358 -2.252234
C 7.754333 3.701789 -2.730778
C 8.435292 6.325959 -1.893881
C 8.581943 1.637347 -0.744071
C 8.160765 -1.061902 -1.308278
C 9.446582 -0.565748 -1.253570
C 9.654580 0.791615 -0.942105
C 9.260901 5.669119 -2.850567
C 8.897129 4.329550 -3.293775
C 7.205516 2.586654 -3.404420
C 8.567157 7.729238 -1.759961
H 8.764253 2.672434 -0.488364
H 7.981474 -2.115209 -1.503464
H 10.294887 -1.221902 -1.422022
H 10.664762 1.177698 -0.849581
C 10.309553 6.396268 -3.453940
C 9.552236 3.697851 -4.374910
C 7.839198 2.011539 -4.486765
C 9.052428 2.544684 -4.947574
C 9.569816 8.429815 -2.403807
C 10.483311 7.747443 -3.219800
H 6.268610 2.170716 -3.062165
H 7.849042 8.273162 -1.162327
H 10.978998 5.898952 -4.144875
H 10.446651 4.140842 -4.795041
H 7.397203 1.149165 -4.975219
H 9.574054 2.082493 -5.779843
H 9.641536 9.505905 -2.280466
H 11.294127 8.282446 -3.704192

MH 9 (4th(1))
264
C -1.228618 2.808791 -0.167460
C -2.513633 2.256722 -0.354225
C -2.651532 1.033370 -1.099117
C -1.539851 0.176605 -1.199673
C -0.284778 0.622916 -0.659225
C -0.083822 2.013218 -0.523449
C -1.707363 -1.136576 -1.804357
C -3.887229 0.624571 -1.746900
C -4.105957 -0.764237 -1.956687
C -2.992967 -1.689850 -1.802390
C -3.668261 2.965372 0.151817
C -3.590575 4.358935 0.278723
C -2.290374 4.896427 0.654996
C -1.132286 4.070755 0.556599
C 1.273891 2.492488 -0.727274
C 2.352823 1.620696 -0.520155
C 2.069974 0.305136 0.038500
C 0.764465 -0.228229 -0.123184
C -3.124827 -3.130787 -1.815688
C -2.084715 -3.912253 -2.358443
C -0.771296 -3.314778 -2.522503
C -0.618091 -1.913167 -2.343651
C 1.539419 3.806049 -1.282714
C 2.852729 4.333466 -1.179634
C 3.927773 3.482753 -0.708890
C 3.703209 2.094444 -0.737952
C 0.557538 4.582633 -1.940591
C 0.839433 5.847016 -2.408581
C 2.134223 6.399157 -2.281689
C 3.117705 5.621083 -1.702593
C -4.824353 5.114014 0.158579
C -4.861415 2.307730 0.638503
C -2.189312 6.125993 1.350092
C 0.043674 4.476993 1.225351
C 3.023404 -0.441061 0.766017
H -0.434101 4.172465 -2.079142
H 0.059304 6.423952 -2.898692
C 2.411641 7.798646 -2.790281
H 4.130496 5.991629 -1.654214
C 0.118436 5.685408 1.888930
C -1.005517 6.525700 1.937289
H -3.073981 6.738710 1.462503
H 0.898495 3.815645 1.235712
H 4.002776 -0.014476 0.941212
H 1.036195 5.965672 2.396967
H -0.962719 7.467265 2.475856
C 4.824254 1.263767 -1.138439
C 5.239013 3.936694 -0.301040
C 6.332042 3.058252 -0.428111
C 6.124654 1.814685 -1.153774
C 4.604819 -0.023821 -1.726173
C 5.442200 5.201853 0.385225
C 7.620670 3.447258 0.104567
C 7.155237 1.165645 -1.939479
C 3.747944 8.402698 -2.350987
C 3.907335 9.871050 -2.750562
C 5.251971 10.452819 -2.308975
H 1.595212 8.450639 -2.450030
H 2.350531 7.806961 -3.888154
H 4.576073 7.825506 -2.782412
H 3.849100 8.310060 -1.261934
H 3.089375 10.456879 -2.310201
H 3.800625 9.969635 -3.839142
H 5.375113 10.373605 -1.223037
H 5.344627 11.508599 -2.582150
H 6.084823 9.912793 -2.772986
C 6.917678 -0.146645 -2.448999
C 5.622298 -0.722167 -2.303830
H 3.607092 -0.435929 -1.728927
C 8.375316 1.792571 -2.299438
C 7.936289 -0.823308 -3.161324
H 5.436575 -1.701737 -2.734160

C	6.773035	5.689545	0.557874
C	7.890137	4.810007	0.276557
C	4.373675	5.936171	0.953046
C	8.630566	2.489008	0.548362
C	6.954516	6.928435	1.225685
C	9.282064	5.242350	0.219588
C	10.322173	4.291537	0.414622
C	9.967499	2.918514	0.750832
C	9.342910	1.124114	-3.018696
C	9.140053	-0.209409	-3.428337
C	10.909022	2.003027	1.263021
C	10.552503	0.703232	1.573621
C	9.224188	0.286927	1.402673
C	8.283036	1.168154	0.905736
H	8.546521	2.823252	-2.021442
H	7.737373	-1.831398	-3.514739
C	9.634139	6.550600	-0.184671
C	11.660339	4.703390	0.259558
H	10.265020	1.635416	-3.276445
H	9.914670	-0.734253	-3.978676
H	11.926009	2.329484	1.447023
H	11.294055	0.017595	1.971480
H	8.927552	-0.721132	1.674521
H	7.254940	0.847273	0.804001
C	5.884111	7.654606	1.706964
C	4.580071	7.147662	1.580130
C	11.980791	6.003298	-0.092036
C	10.957089	6.929636	-0.331873
H	3.370710	5.536811	0.897896
H	7.957247	7.275907	1.432033
H	8.851484	7.253933	-0.438431
H	12.457486	3.979544	0.382674
H	6.059323	8.589332	2.230447
H	3.738100	7.687040	2.002801
H	13.020095	6.291792	-0.213820
H	11.196008	7.936575	-0.659430
C	0.475703	-1.505391	0.408754
C	1.441679	-2.236686	1.070405
C	2.725873	-1.697612	1.257416
C	0.599034	-1.314812	-2.753903
C	0.407466	-4.081368	-2.725685
C	1.634215	-3.490554	-2.958410
C	1.687485	-2.082524	-3.085738
H	-0.528790	-1.898659	0.321876
H	1.194167	-3.212492	1.475895
H	3.477456	-2.254756	1.807548
H	0.668022	-0.235192	-2.794933
H	0.342467	-5.161354	-2.684883
C	2.921431	-4.272684	-3.003631
H	2.611050	-1.606024	-3.401299
C	3.744401	-4.034521	-1.722207
C	5.166384	-4.594508	-1.776871
C	5.980937	-4.227844	-0.534092
H	3.515384	-3.954465	-3.871353
H	2.720752	-5.342127	-3.131857
H	3.203891	-4.459424	-0.866857
H	3.792372	-2.957175	-1.526067
H	5.672315	-4.205827	-2.672056
H	5.133713	-5.685149	-1.898927
H	6.058627	-3.140013	-0.430084
H	6.995904	-4.634742	-0.581232
H	5.506839	-4.614926	0.374819
C	-6.050606	4.452710	0.374280
C	-6.030060	3.077307	0.848371
C	-7.113917	2.494528	1.539778
C	-7.118155	1.167938	1.928987
C	-8.323186	0.537291	2.597064
C	-9.617281	1.349529	2.487814
C	-10.852701	0.615320	3.011173
C	-12.121028	1.462682	2.882497
H	-8.098981	0.344270	3.656191
H	-8.481777	-0.453090	2.148116
H	-9.781785	1.627210	1.439104
H	-9.509717	2.293314	3.037897
H	-10.699372	0.332898	4.061301
H	-10.978744	-0.324634	2.457280
H	-12.036711	2.385580	3.467490
H	-13.006360	0.923720	3.234068
H	-12.292935	1.751219	1.839295
C	-4.850215	0.962907	1.082880
C	-5.954281	0.403331	1.688088
C	-4.862852	1.542890	-2.189176
C	-5.332106	-1.181385	-2.526816
C	-6.051042	1.112205	-2.747987
C	-6.295743	-0.263557	-2.897075
C	-4.151056	-3.811731	-1.039759
C	-2.422312	-5.270127	-2.745530
C	-4.865050	6.462461	-0.375755
C	-7.274527	5.195437	0.261508
H	-7.972233	3.110173	1.757097
H	-3.947795	0.375857	0.969596
H	-5.914839	-0.631835	2.018400
H	-4.669567	2.604183	-2.093432
H	-5.494256	-2.236350	-2.710830
H	-6.782472	1.838705	-3.088177
H	-7.223051	-0.607106	-3.344741
C	-7.222603	6.601001	0.317691
C	-6.060310	7.220097	-0.285256
C	-3.739930	7.013096	-1.068280
C	-8.579636	4.624827	-0.039061
C	-8.352939	7.264679	0.960562
C	-6.136654	8.503884	-0.968231
C	-3.765965	8.270585	-1.587605
C	-4.957289	9.051201	-1.557474
H	-2.849362	6.411492	-1.180255
H	-2.891877	8.672959	-2.091660
C	-9.599239	6.629099	0.975895
C	-9.746008	5.399330	0.207250
C	-8.691753	3.428272	-0.785388
C	-8.172456	8.510534	1.703441

C	-10.702156	7.203151	1.730580
C	-10.967800	4.971721	-0.360878
C	-7.344748	9.221854	-1.154471
C	-5.000798	10.320228	-2.182492
C	-6.181065	11.023494	-2.279806
C	-7.367120	10.449727	-1.782573
H	-8.276699	8.798037	-0.811968
H	-4.082701	10.718111	-2.605922
H	-6.204562	11.995459	-2.762671
H	-8.312200	10.970270	-1.901242
C	-10.605434	8.538977	2.212293
C	-9.311574	9.208949	2.190300
C	-6.894177	9.053662	1.966297
C	-6.739126	10.289769	2.560486
C	-7.869995	11.034332	2.925737
C	-9.127939	10.488761	2.760004
C	-11.865451	6.461501	2.050963
C	-11.739200	9.122551	2.818680
H	-6.014332	8.489938	1.692899
H	-5.742731	10.680425	2.740615
H	-7.759866	12.018830	3.369558
H	-9.986517	11.052639	3.102554
C	-9.910509	3.008373	-1.282089
C	-11.056712	3.793878	-1.080702
H	-7.800122	2.854723	-1.001937
H	-11.842004	5.602880	-0.269472
H	-9.967684	2.094726	-1.865655
H	-12.005710	3.498613	-1.517130
C	-12.899076	8.402257	3.033300
C	-12.945566	7.044478	2.685345
H	-11.897582	5.405012	1.822092
H	-11.698301	10.149808	3.158568
H	-13.751372	8.876594	3.509611
H	-13.820948	6.447366	2.920864
C	-4.170811	-5.223490	-1.024764
C	-3.506562	-5.911459	-2.117986
C	-4.981722	-3.102036	-0.110792
C	-1.800090	-5.939749	-3.877106
C	-4.794527	-5.908205	0.087619
C	-3.899616	-7.238140	-2.549094
C	-1.965692	-7.350116	-4.022283
C	-2.956813	-8.030067	-3.213361
C	-1.117776	-5.233144	-4.895019
C	-5.251811	-7.773290	-2.404135
C	-1.350808	-7.992737	-5.127603
C	-3.083794	-9.478456	-3.111690
C	-5.746287	-3.761835	0.804695
C	-5.646233	-5.173831	0.965297
C	-6.314884	-5.840844	2.019390
C	-4.550957	-7.270325	0.397236
C	-6.107497	-7.182941	2.250369
C	-5.191307	-7.892637	1.447137
H	-4.994047	-2.022357	-0.122690
H	-6.386015	-3.203080	1.482531
H	-6.977170	-5.264727	2.659696
H	-3.835695	-7.829058	-0.191191
H	-6.620504	-7.685533	3.064380
H	-4.981186	-8.936266	1.658753
C	-5.472189	-9.169869	-2.524393
C	-4.325410	-10.048562	-2.715849
C	-6.370471	-6.921416	-2.276165
C	-7.654991	-7.426632	-2.214410
C	-7.868798	-8.810602	-2.292426
C	-6.791602	-9.661825	-2.459057
C	-1.961782	-10.328496	-3.240251
C	-4.403039	-11.440817	-2.516913
H	-6.217732	-5.850879	-2.243548
H	-8.496673	-6.747666	-2.121428
H	-8.876435	-9.212103	-2.250439
H	-6.971498	-10.724394	-2.574569
C	-0.527301	-5.884916	-5.958031
C	-0.629254	-7.281524	-6.064882
C	-3.298706	-12.257930	-2.688053
C	-2.064803	-11.694054	-3.039543
H	-1.073430	-4.154296	-4.846202
H	-1.521991	-9.048417	-5.287465
H	-0.991655	-9.894568	-3.446664
H	-5.338200	-11.880567	-2.190167
H	-0.014128	-5.312535	-6.724189
H	-0.191748	-7.798522	-6.913312
H	-3.383866	-13.326886	-2.519714
H	-1.182799	-12.320439	-3.129066

MH 9 (4th(2))

264

C	-1.737078	2.613083	-2.109054
C	-2.961639	2.029561	-1.718443
C	-3.107208	0.613201	-1.933673
C	-1.944996	-0.158530	-2.135643
C	-0.663258	0.436456	-1.849233
C	-0.542410	1.837299	-1.934021
C	-2.073038	-1.554847	-2.512926
C	-4.396071	-0.048387	-2.091738
C	-4.449817	-1.470114	-2.002265
C	-3.215602	-2.239773	-2.090452
C	-3.943197	2.931279	-1.136737
C	-3.890405	4.298286	-1.447844
C	-2.951279	4.733491	-2.471898
C	-1.811662	3.925481	-2.721456
C	0.768204	2.447197	-1.813018
C	1.895558	1.640044	-2.038037
C	1.788128	0.248870	-1.626993
C	0.502101	-0.320857	-1.426159
C	-3.132346	-3.678536	-1.937692
C	-2.187699	-4.396902	-2.698384
C	-1.056697	-3.684816	-3.265800
C	-1.049712	-2.265342	-3.238974
C	1.009486	3.800450	-1.350619
C	2.288706	4.379396	-1.550985
C	3.271363	3.658939	-2.347939

C	3.105151	2.271509	-2.523805
C	1.660690	6.305914	-0.170740
C	2.589784	5.603614	-0.914289
C	-4.830432	5.207522	-0.830084
C	-4.872316	2.491929	-0.112018
C	2.928680	-0.489044	-1.227533
C	1.981910	7.655538	0.438654
H	3.586204	6.001062	-1.018472
H	3.908923	-0.044459	-1.338881
C	3.281901	8.294542	-0.058143
C	3.501520	9.720931	0.448081
C	4.811500	10.318612	-0.071613
H	2.010602	7.566768	1.534139
H	1.143781	8.334014	0.225066
H	3.286934	8.299100	-1.155508
H	4.136859	7.676951	0.246342
H	3.498983	9.729296	1.546183
H	2.658258	10.351539	0.135831
H	5.672083	9.731723	0.269029
H	4.952030	11.348284	0.271759
H	4.830970	10.321968	-1.167403
C	0.404859	-1.578922	-0.791567
C	1.532082	-2.287206	-0.428544
C	2.806493	-1.740418	-0.658467
C	-0.051196	-1.583064	-3.976382
C	0.117340	-4.341111	-3.723123
C	1.162261	-3.650295	-4.305107
C	1.010686	-2.260266	-4.521249
H	-0.574458	-1.989260	-0.581933
H	1.430134	-3.254169	0.053274
H	3.696394	-2.283435	-0.356323
H	-0.127836	-0.510024	-4.097538
H	0.200260	-5.413325	-3.597770
C	2.477747	-4.296734	-4.656351
H	1.763937	-1.721381	-5.088635
C	3.584646	-3.853367	-3.679051
C	5.000133	-4.213305	-4.132943
C	6.072042	-3.642896	-3.201154
H	2.766893	-4.003002	-5.674977
H	2.385445	-5.388377	-4.657331
H	3.382527	-4.284418	-2.690315
H	3.521393	-2.768522	-3.539344
H	5.159523	-3.826272	-5.149490
H	5.104161	-5.303924	-4.205839
H	6.011743	-2.549742	-3.165386
H	7.079943	-3.911956	-3.532386
H	5.944678	-4.017264	-2.179124
C	-6.028758	4.674005	-0.323307
C	-5.975597	3.325913	0.207693
C	-6.899197	2.893268	1.188629
C	-6.780343	1.675627	1.829706
C	-7.802837	1.195986	2.839031
C	-9.100202	2.006635	2.893381
C	-10.160034	1.390939	3.809232
C	-11.444583	2.221284	3.854165
H	-7.345099	1.171918	3.838507
H	-8.044064	0.149551	2.605899
H	-9.510576	2.106101	1.880162
H	-8.885629	3.027662	3.234819
H	-9.752493	1.283842	4.823404
H	-10.389903	0.373846	3.464493
H	-11.248162	3.227775	4.240139
H	-12.202045	1.759427	4.495116
H	-11.873638	2.333162	2.852114
C	-4.718304	1.284211	0.607689
C	-5.651893	0.877666	1.534916
C	-5.587581	0.656688	-2.364509
C	-5.711012	-2.106619	-2.087795
C	-6.806684	0.011875	-2.440848
C	-6.871047	-1.380967	-2.277481
C	-3.783151	-4.380336	-0.841977
C	-2.434382	-5.816970	-2.872967
C	-4.559207	6.602061	-0.530275
C	-7.177606	5.551393	-0.272358
H	-7.710441	3.554818	1.451313
H	-3.847138	0.669238	0.425965
H	-5.510885	-0.062920	2.061294
H	-5.540759	1.724775	-2.526201
H	-5.757325	-3.187592	-2.050418
H	-7.706699	0.581798	-2.650240
H	-7.823357	-1.896995	-2.349586
C	-6.967620	6.942906	-0.237549
C	-5.605884	7.420854	-0.051361
C	-3.213151	7.091753	-0.486917
C	-8.536737	5.057565	-0.413232
C	-8.105765	7.834059	-0.318291
H	-2.408558	6.463217	-0.838918
C	-9.364378	7.348817	0.056081
C	-9.625653	5.935055	-0.125892
C	-8.823351	3.756689	-0.891383
C	-8.026717	9.194831	-0.844998
C	-10.345929	8.307795	0.550609
C	-10.946684	5.435669	-0.264615
C	-10.186593	9.690885	0.258615
C	-9.086032	10.106772	-0.601410
C	-11.366947	7.923824	1.450080
C	-11.086624	10.615350	0.824020
C	-10.118447	3.292988	-0.996660
C	-11.191184	4.138916	-0.668767
H	-8.006547	3.111935	-1.184707
H	-11.782908	6.106648	-0.125545
H	-10.304759	2.289068	-1.365294
H	-12.214247	3.794554	-0.783652
C	-12.107528	10.208560	1.665639
C	-12.237498	8.852403	1.993595
H	-11.433771	6.889976	1.763572
H	-10.957273	11.673029	0.626202
H	-12.781630	10.942969	2.095115
H	-13.001277	8.529630	2.694143
C	-3.615089	-5.777379	-0.724839

C	-3.206400	-6.501106	-1.915927
C	-4.379628	-3.663955	0.246804
C	-2.066520	-6.530576	-4.085900
C	-3.795055	-6.403742	0.567446
C	-3.529239	-7.898446	-2.122630
C	-2.085273	-7.958062	-4.081281
C	-2.716837	-8.651542	-2.977099
C	-1.783589	-5.864314	-5.301341
C	-4.704904	-8.553395	-1.552846
C	-1.723493	-8.642488	-5.270205
C	-2.623240	-10.087572	-2.745356
C	-4.747620	-4.297451	1.396291
C	-4.433494	-5.670259	1.611335
C	-4.673849	-6.288702	2.861661
C	-3.304727	-7.697154	0.880088
C	-4.244308	-7.573929	3.108659
C	-3.525461	-8.269005	2.114458
H	-4.521836	-2.596268	0.162577
H	-5.213471	-3.736985	2.202016
H	-5.183129	-5.717435	3.632878
H	-2.730397	-8.240305	0.141931
H	-4.428539	-8.038715	4.072209
H	-3.135016	-9.260253	2.322055
C	-4.769872	-9.970278	-1.511017
C	-3.620653	-10.740439	-1.969266
C	-5.840667	-7.813164	-1.158520
C	-6.979952	-8.439617	-0.690876
C	-7.028832	-9.839128	-0.612842
C	-5.943096	-10.586868	-1.030931
C	-1.482362	-10.825444	-3.135353
C	-3.460617	-12.106137	-1.663776
H	-5.822586	-6.734695	-1.241831
H	-7.840373	-7.845329	-0.400054
H	-7.923751	-10.336414	-0.252099
H	-6.011102	-11.668523	-1.018813
C	-1.430830	-6.556220	-6.441578
C	-1.383200	-7.959638	-6.420307
C	-2.353070	-12.817611	-2.092232
C	-1.349275	-12.167066	-2.822412
H	-1.862725	-4.787081	-5.340494
H	-1.797874	-9.720778	-5.302762
H	-0.672222	-10.316414	-3.641854
H	-4.202674	-12.605160	-1.051410
H	-1.223452	-6.014547	-7.358953
H	-1.135265	-8.512244	-7.321248
H	-2.250175	-13.867219	-1.835334
H	-0.454526	-12.704315	-3.120911
C	-3.140711	5.898955	-3.245767
C	-0.872216	4.341124	-3.691502
C	-1.060414	5.507665	-4.406917
C	-2.208939	6.288242	-4.189366
C	0.083344	4.495801	-0.532716
C	4.486471	4.276050	-2.805599
C	4.067212	1.567305	-3.351196
C	0.389863	5.719925	0.020909

H	-4.035237	6.490500	-3.094921
H	-0.018193	3.709509	-3.902429
H	-0.337644	5.798857	-5.162860
H	-2.377076	7.185789	-4.776087
H	-0.870669	4.039394	-0.305023
H	-0.346832	6.223547	0.641762
C	5.607301	3.460273	-3.055082
C	5.334614	2.147772	-3.604111
C	4.641967	5.681667	-3.147722
C	3.729186	0.325539	-3.976465
C	6.910192	4.036999	-2.738909
C	6.235500	1.495438	-4.544525
C	5.950437	6.229782	-3.245951
C	7.065405	5.427240	-2.759304
C	8.011061	3.197674	-2.269409
C	6.104145	7.491192	-3.865063
C	8.313857	6.028224	-2.316250
C	9.308445	3.760612	-2.121733
C	9.464534	5.209426	-2.138327
C	4.621576	-0.351525	-4.749154
C	5.898711	0.203496	-5.050218
C	3.540432	6.440017	-3.608622
C	3.717697	7.694873	-4.158404
C	5.012257	8.219149	-4.302113
H	2.741453	-0.083107	-3.825544
C	7.413201	2.099407	-5.051477
C	7.835269	1.832801	-1.946428
H	7.100393	7.874848	-4.041587
C	8.421735	7.405798	-2.005775
C	10.390109	2.899520	-1.830666
C	10.698365	5.831426	-1.848082
H	4.349060	-1.301673	-5.198429
C	6.791162	-0.476322	-5.912549
H	2.548333	6.009336	-3.563246
H	2.858577	8.250990	-4.520305
H	5.162559	9.178977	-4.786223
C	8.260039	1.426084	-5.907565
C	7.966876	0.112641	-6.322651
C	8.903388	1.019456	-1.626054
C	10.201623	1.549509	-1.607659
C	9.634003	7.983223	-1.680022
C	10.793531	7.195224	-1.644842
H	7.655061	3.114543	-4.774554
H	6.840535	1.411889	-1.953830
H	7.528595	8.015441	-1.992147
H	11.391899	3.302853	-1.751711
H	11.592864	5.229797	-1.746162
H	6.515622	-1.467813	-6.261345
H	9.156158	1.920911	-6.268535
H	8.645847	-0.415828	-6.984527
H	8.733564	-0.026646	-1.392163
H	11.051778	0.912946	-1.383288
H	9.682307	9.041408	-1.442958
H	11.755151	7.643161	-1.414833
C	-6.978296	9.590086	-1.703812

C	-9.036702	11.385326	-1.192724
C	-5.277282	8.636788	0.664636
C	-2.924894	8.323016	0.020363
C	-3.934132	9.120227	0.633572
C	-6.949476	10.850416	-2.269668
C	-7.982090	11.761619	-2.004632
H	-6.192391	8.883996	-1.936339
H	-9.854761	12.078597	-1.035078
C	-6.205989	9.344097	1.470239
H	-1.898344	8.679433	0.031733
C	-3.606835	10.336238	1.279903
H	-6.134348	11.125128	-2.931615
H	-7.968617	12.749926	-2.453378
C	-4.554028	11.035733	1.994474
C	-5.857638	10.512541	2.113190
H	-7.204299	8.950645	1.602084
H	-2.582397	10.693545	1.221048
H	-4.292540	11.965148	2.490685
H	-6.594261	11.026783	2.722393
